# Genomic evolution and complexity of the Anaphase-promoting Complex (APC) in land plants

**DOI:** 10.1186/1471-2229-10-254

**Published:** 2010-11-18

**Authors:** Marcelo de F Lima, Núbia B Eloy, Camila Pegoraro, Rauan Sagit, Cristian Rojas, Thiago Bretz, Lívia Vargas, Arne Elofsson, Antonio Costa de Oliveira, Adriana S Hemerly, Paulo CG Ferreira

**Affiliations:** 1 Laboratório de Biologia Molecular de Plantas, Instituto de Bioquímica Médica, CCS, Cidade Universitária - Ilha do Fundão, CEP 21941-590, Rio de Janeiro, RJ, Brasil; 2 Centro de Genômica e Fitomelhoramento, Departamento de Fitotecnia, Faculdade de Agronomia Eliseu Maciel, Universidade Federal de Pelotas, Campus Universitário s/n - Capão do Leão, CEP 90001-970, Pelotas, RS, Brasil; 3 Stockholm Bioinformatics Center, Center for Biomembrane Research, Department of Biochemistry and Biophysics, Stockholm University, 106, 91,Stockholm, Sweden

## Abstract

**Background:**

The orderly progression through mitosis is regulated by the Anaphase-Promoting Complex (APC), a large multiprotein E_3 _ubiquitin ligase that targets key cell-cycle regulators for destruction by the 26 S proteasome. The APC is composed of at least 11 subunits and associates with additional regulatory activators during mitosis and interphase cycles. Despite extensive research on APC and activator functions in the cell cycle, only a few components have been functionally characterized in plants.

**Results:**

Here, we describe an in-depth search for APC subunits and activator genes in the Arabidopsis, rice and poplar genomes. Also, searches in other genomes that are not completely sequenced were performed. Phylogenetic analyses indicate that some APC subunits and activator genes have experienced gene duplication events in plants, in contrast to animals. Expression patterns of paralog subunits and activators in rice could indicate that this duplication, rather than complete redundancy, could reflect initial specialization steps. The absence of subunit APC7 from the genome of some green algae species and as well as from early metazoan lineages, could mean that APC7 is not required for APC function in unicellular organisms and it may be a result of duplication of another tetratricopeptide (TPR) subunit. Analyses of TPR evolution suggest that duplications of subunits started from the central domains.

**Conclusions:**

The increased complexity of the APC gene structure, tied to the diversification of expression paths, suggests that land plants developed sophisticated mechanisms of APC regulation to cope with the sedentary life style and its associated environmental exposures.

## Background

Cell proliferation is controlled by an universally conserved molecular machinery in which the key players are cyclin-dependent kinases (CDK) and cyclins (reviewed in [1]). Eukaryotes have therefore evolved elaborate mechanisms for CDK regulation. An irreversible mechanism of CDK down-regulation is destruction of cyclin subunits [2,3]. At the G_1_- to S-phase and metaphase to anaphase transitions, CDKs are irreversibly inactivated by ubiquitin-mediated proteolysis of cognate cyclins [4]. Degradation of protein substrates through the ubiquitin-proteasome pathway involves the activity of different E3 ligases, among them the anaphase-promoting complex (APC). The APC was first identified based on its role in facilitating the multiubiquitination and targeting of A- and B-type cyclins for proteasome-mediated destruction during mitosis [5-8]. APC is a multiprotein complex conserved from plants to man and contains at least 11 core subunits [9]. The APC is regulated in part by two associated proteins, CDC20 and CCS52/CDH1, that can both activate the APC with proper timing and provide substrate specificity [10,11]. The APC is activated at metaphase/anaphase transition by the CDC20 protein and later in telophase and G_1 _by the CDH1 protein. Substrates that have a destruction box (D-box), KEN-box or A-box motifs are recognized and ubiquitinated by the APC [12-14].

The APC has important functions in mitosis, meiosis, G_1_-S-phase and in post-mitotic differentiated cells [15,16]. The mitosis-specific activator CDC20 is itself an APC substrate. Other targets of APC/C degradation are: Cyclins A and B; protein kinases Plk1, CDC5, Aurora A and B; regulators of DNA replication Geminin, CDC6; and the anaphase inhibitor Securin (reviewed in [17]). The proteolytic events triggered by the APC are required to release sister chromatides cohesion during anaphase, to control the exit from mitosis and to prevent premature entry into S-phase [6,18,19].

Although the role of the APC in controlling cell-cycle progression has been extensively investigated, has recent work shown the unexpected presence of APC subunits in differentiated tissues, in particular in the nervous system of vertebrates (reviewed in [20]). In plants, the expression of several APC subunits has been detected in differentiated tissues of Arabidopsis [21,22]. In addition, while both CDC27a and CDC27b genes are essential during gametogenesis, the CDC27b subunit has been implicated also in post-embryogenic differentiation at meristem levels [23]. Besides, it has been shown that reduced levels of APC6 and APC10 subunits in Arabidopsis lead to plants with several defects in vascular development [24]. These results indicate that, while the overall structure of the APC is conserved among eukaryotes, this E3 ligase may have assumed specialized functions in the diverse kingdoms. Gene duplication and retention in plants has been extensive and gene families are generally larger in plants than in animals [25]. Nevertheless, almost all studies on the APC function in plants have been carried out in the model plant Arabidopsis thaliana. Comparative genomic analyses can provide valuable insights into the organization of cell cycle machinery and the evolution of these protein complexes.

In this article, we describe thorough searches for the predicted sequences of the APC subunits and the activators CDC20/CCS52 in plant sequence databases. Our results indicate that land plants and green algae orthologs have an ancient evolutionary origin. We present phylogenetic analysis of TPR subunits and activators, and their orthologs from other plants. An evolutionary analysis of Arabidopsis TPR domain suggests that tandem regions could have been created from duplication of internal sequence domains. Overall, our data support the proposal that APC subunits and activators have been conserved in the course of evolution. However, while other eukaryotes like fungi and metazoans have only one copy of each APC subunit and its regulators, gene duplication of different subunits have occurred in Arabidopsis, rice and poplar, and in other plant genomes as well. An attractive proposal is that gene duplication of APC subunits may result in the formation of APC subcomplexes that evolved to assume restricted specialized roles during plant development. Indeed, in Arabidopsis, duplication of the CDC27 subunit led to substantial sequence divergence and specialization [23]. Here we show that the two copies of rice CDC23 and APC11 subunits and the activators genes are differentially expressed in plant tissues or when grown under either dark or light-mediated developmental programs, suggesting the duplicated genes could be assuming new functions in the plant.

## Results and Discussion

### Identification of APC subunits and activator genes

In order to carry out the cross-species comparison of the APC and activators, we have searched for homologous sequences in the Genomic Research (TIGR) Rice genome database and DOE Join Genome Institute (JGI) Poplar genome database. The sequences of the Arabidopsis APC subunits have been published [21,26,27]. Even after exhaustive data mining, some rice and poplar sequences were only found in the EST database Gene Index DFCI. Subsequently, the SMART motif identification tool was used to identify predicted domains in all candidate proteins [28]. Using this strategy, we report the APC/activators in the dicotyledonous poplar and the monocotyledon rice plants (Table 1 and Additional file 1). All APC subunits and activators were found, and they contained the predicted conserved domains, providing one of many examples for the evolutionary conservation of the eukaryotic cell-cycle machinery [29,30].

**Table 1:** APC subunits and activators in Arabidopsis, rice and poplar genomes.

Gene Description	*Arabidopsis thaliana*	*Oryza sativa*	*Populus trichocarpa*	Protein Motifs
**APC Subunits**	**Access number**			

APC1	At5g05560	TC286185^a^	Pt765590	Rpn1/2 repeats

APC2	At2g04660	LOC_Os04g40830	Pt832637	Cullin domain

APC3/CDC27a	At3g16320			TPR repeats

APC3/CDC27b	At2g20000	LOC_Os06g41750	Pt835890	TPR repeats

			Pt278795	TPR repeats

APC4	At4g21530	LOC_Os02g54490	Pt817758	WD-40 repeats

APC5	At1g06590	LOC_Os12g43120	Pt592813	TPR repeats

APC6/CDC16	At1g78770	LOC_Os03g13370	Pt585761	TPR repeats

APC7	At2g39090	LOC_Os05g05720	Pt828004	TPR repeats

APC8/CDC23	At3g48150	LOC_Os02g43920	Pt834319	TPR repeats

		LOC_Os06g46540		TPR repeats

APC10	At2g18290	LOC_Os05g50360	Pt796785	Doc domain

APC11	At3g05870	LOC_Os03g19059	Pt292476	RING-H2 domain

		LOC_Os07g22840		RING-H2 domain

CDC26	TC308166^a^	TC356501^a^	TC118342^a^	-

APC13	At1g73177	TC311476^a^	Pt647861	

			Pt660762	-

**Activators**				

CDC20_1	At4g33260	LOC_Os09g06680	Pt571123	WD-40 repeats

CDC20_2	At4g33270	LOC_Os04g51110	Pt272847	WD-40 repeats

CDC20_3	At5g26900	LOC_Os02g47180	Pt738273	WD-40 repeats

CDC20_4	At5g27080		Pt256238	WD-40 repeats

CDC20_5	At5g27570		Pt257786	WD-40 repeats

CDC20_6	At5g27945			WD-40 repeats

CCS52A1	At4g22910	LOC_Os03g03150	Pt415429	WD-40 repeats

CCS52A1_2			Pt180625	WD-40 repeats

CCS52A2	At4g11920			WD-40 repeats

CCS52B	At5g13840	LOC_Os01g74146	Pt820353	WD-40 repeats

CCS52B_2			Pt833809	WD-40 repeats

^a^Gene index project access number http://compbio.dfci.harvard.edu/tgi/

Two CDC27 homologs have been identified in Arabidopsis *AtCDC27a *and *AtCDC27b *[31]. In poplar, two homologs of CDC27 were also identified: *PtCDC27_1 *and *PtCDC27_2*. However, a careful inspection of the *PtCDC27_2 *deposited sequence revealed a stop codon TAG at nucleotide position 1471-1473. This fragment was amplified and sequenced and the presence of the stop codon was discarded. The expression of both genes in leaves was confirmed by quantitative PCR (see Additional file 2). We also found two homologs of CDC27 in the bryophyte *Physcomitrella patens *genome [32]. However, only one copy of CDC27 was found in the rice genome as well as other monocot plants. On the other hand, the rice genome has two *OsCDC23 *and *OsAPC11 *subunits [33]. We examined other complete genomes and found two CDC23 (*VvCDC23_1 and VvCDC23_2*) and APC11 (*VvAPC11_1 and VvAPC11_2*) genes in grapevine [34]. Two homologs of *PtAPC13 *were found in the poplar and grapevine genomes, a feature that has not been reported in any other eukaryotic species so far.

The structures of many predicted genes were considerably misannotated. Comparing EST databases from plant species and using bioinformatics tools, it was possible to identify the correct gene sequences. The genomic sequence of *OsAPC1 *was not found in the TIGR database, but a partial CDS was identified in the Gene Index database (see Additional file 3). Most likely, the largest subunit of the APC had come apart by misannotation. Diverse exon-intron structures needed correction in the genomics sequences. *OsAPC4 *may contain an intron in the genomic ORF and *OsCDC23_2 *may have a fragment of an exon involved in the formation of TPR domain erroneously annotated as an intron (see Additional file 4). The 3'region of predicted *PtAPC4 *and the 5'-3'regions of *PtAPC5 *were incomplete. However, we found fragments from 3'regions of both genes and fragments from the 5'region of *PtAPC5 *in the EST database (see Additional file 4). Interestingly, *OsAPC5*, *OsCDC16 *and *OsAPC11_2 *have a divergent 5'region compared to Arabidopsis and poplar genes (see Additional files 4 and 5). The first methionine of *OsCDC16 *and *OsAPC11_2 *are upstream from a consensus start codon in CDC16 *and *APC11 genes, but only for CDC16 there is EST support. The opposite occurs with *OsAPC5*, where the first methionine is downstream from the consensus start codon. The APC genes *AtCDC26*, *OsCDC26*, *PtCDC26 *and *OsAPC13 *were identified in EST databases.

Arabidopsis contains three CCS52 genes, *AtCCS52A1*, *AtCCS52A2 *and *AtCCS52B*; and five CDC20 genes [27]. In addition, we found another CDC20 homolog in the Arabidopsis genome. This *AtCDC20_6 *(At5g27945) showed major differences from conserved structures of other CDC20; the CDS does not have the C-box sequence element (consensus DR(F/Y)IPxR) that was first identified in the N-terminal region of CDC20, although it is conserved in all known APC/C co-activators (this was confirmed by re-sequencing - see Additional file 6), and therefore *AtCDC20_6 *could be a pseudogene. The rice genome has three predicted CDC20 genes compared to five in Poplar and Arabidopsis. Rice has two CCS52 genes compared to four and three genes in Poplar and Arabidopsis, respectively (Table 1). Exon-intron organization of activators was analyzed and we found a number of annotation errors. *AtCDC20_5*, *PtCDC20_5*, *PtCCS52A1 *and *PtCCS52A1_2 *have errors in the 5' region. We found that only *PtCDC20_5 *is without a C-box. *OsCDC20_1*, *AtCDC20_4*, *PtCDC20_2*, *PtCDC20_4 *and *PtCDC20_5 *had mistakes in the 3' region. This region contains the IR-tail sequence element (consensus IR) that occupies the C terminus of APC activators and the APC subunit APC10. Comparing with EST databases, all sequences were deduced and the IR-tail was identified (see Additional file 7). In *PtCCS52A1_2 *and *OsCDC20_2 *genes, introns were incorrectly included. The opposite happened in the *OsCDC20_1 *gene, where one exon was absent. Alternative splicing variants were found in the Arabidopsis, rice and poplar APC/activator genes (data not shown) and could represent another layer of complexity in the organization and function of the APC. We selected only a single variant for further analysis.

Duplication of cell-cycle machinery components is rare in metazoans but it is a widespread phenomenon in plants [35-38].It has been proposed that duplication, followed by sequence divergence of promoter and/or coding regions, leads to novel and specialized functions unique to the plant kingdom [23,39]. Still, additional file 8 shows that duplications of individual subunits of the APC are not present in all plants; on the contrary, they may be restricted to one or to a small group of phylogenetically related plant species and may be involved in the organization of developmental events unique to this group.

### Chromosomal location of genes for APC subunits and activator

The close similarity and presence of putative subunits and paralogs prompted us to investigate the genome distribution of plant APC/activators. APC genes were found in the five Arabidopsis chromosomes, 10 of the 12 rice chromosomes and 10 of the 19 poplar chromosomes (and seven in scaffolds) (see Additional file 9).

Only one APC subunit is duplicated in the Arabidopsis genome, *AtCDC27a *and *AtCDC27b*, and they are located on different chromosomes, 3 and 2 respectively; they share 47% identity and 64% protein similarity. A duplication event is predicted to have occurred in this case according to the tool Paralogons in Arabidopsis [40]. Arabidopsis CDC20 and CCS52 genes are located on chromosomes 4 and 5. *AtCCS52A1 *and *AtCCS52A2 *appear to be the result of a recent duplication and *AtCDC20_1 *and *AtCDC20_2 *are present in tandem on chromosome 4 [27].

The 19 rice APC and activator genes are distributed among all chromosomes, except chromosomes 10 and 11. Based on the sequence consensus, rice genome, segmental duplication information from the TIGR database was used to identify paralog genes. Each pair of paralogs located in the corresponding segmental duplication regions share high sequence similarity. Two subunits - CDC23 and APC11 *- *are duplicated. *OsCDC23_1 *and *OsCDC23_2 *are located on chromosomes 2 and 6, respectively and share 92% identity. *OsAPC11_1 and OsAPC11_2 *are located on chromosome 3 and 7, respectively and share 97% identify. No evidences for recent duplication events in rice activators were found.

*In-silico *chromosome mapping revealed that poplar APC/activators are scattered throughout the genome. Paralogs are also located on different chromosomes or scaffolds. It is possible, however, that some of the apparently closely related genes are in fact alleles from unassembled haplotypes, which are potential artifacts from shotgun assembly of this highly heterozygous genome. However, the apparent co-orthologs are divergent at the nucleotide level, as well as in the flanking gene order, and they are identified in the syntenic blocks which argue against the classification of the scaffold as a haplotype. Duplications of two APC subunits were identified; CDC27 and APC13. *PtCDC27_1 *is located on linkage group VIII and *PtCDC27_2 *on scaffold_211. Both are very similar to each other - 88% identity and 85% similarity in their amino-acid sequences, respectively. *PtAPC13_1 *is 95% identical to *PtAPC13_2 *and they are located on linkage groups IV and XI, respectively. Poplar CCS52 genes are closely related, suggesting a recent genomic duplication event. Linkage groups III and I and VIII and X share large megabase-blocks in complete colinearity. *PtCDC20_1 *and *PtCDC20_3 *are located in chromosomal regions that might represent paralog segments - linkage groups XIII and XIX. Other CDC20 genes are located on linkage group XVI and scaffold_1538. The scattered distribution of APC/activators is in good agreement with previous studies that described large-scale duplication events in poplar [41].

### Phylogenetic analysis of APC TPRs subunits and activators proteins

**Figure 1:**
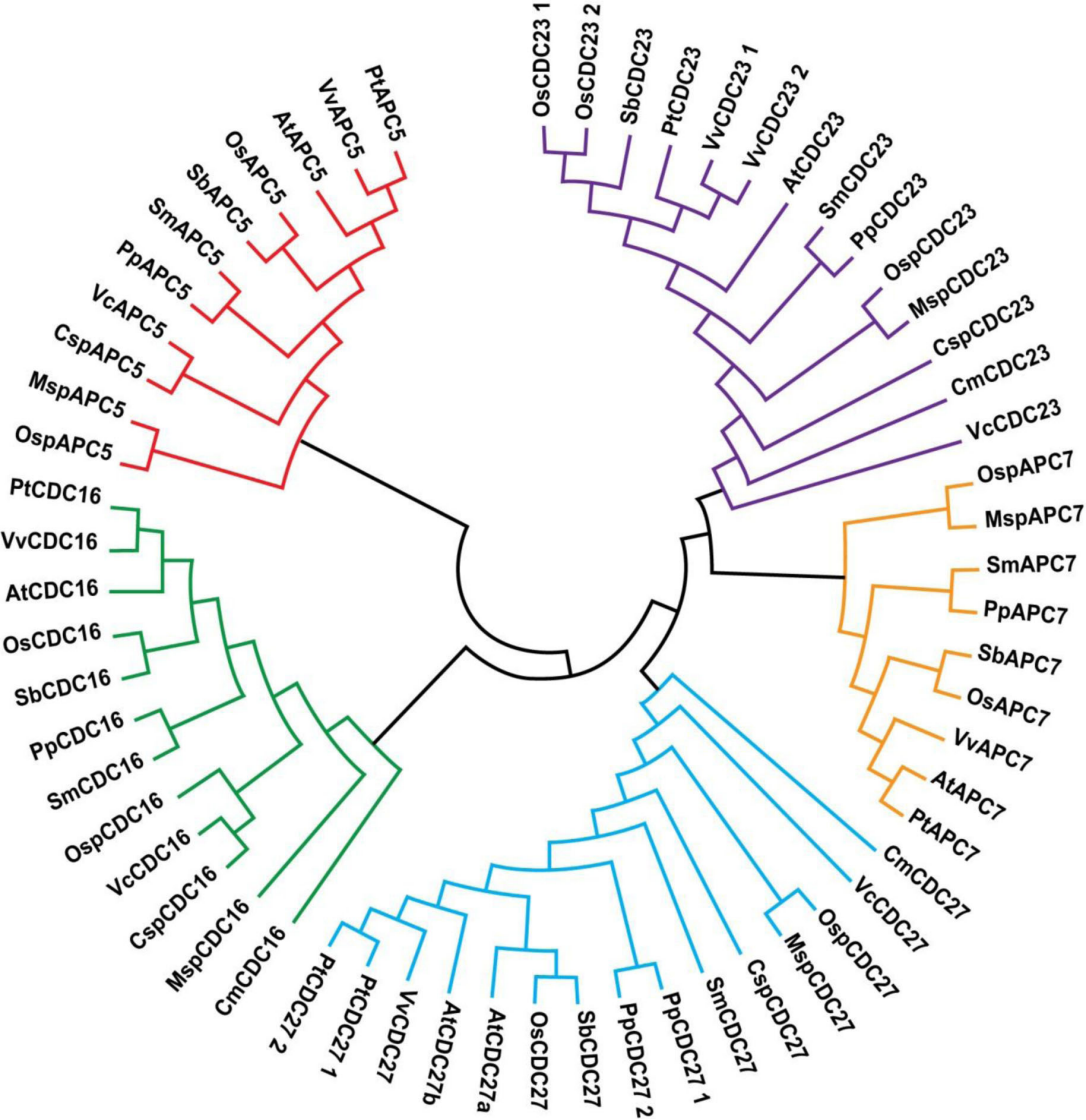
**Phylogenetic relationships of APC TPR proteins from diverse species**. Protein sequences were aligned with ClustalW using the Gonnet scoring matrix in MEGA4. The unrooted tree was generated using the MEGA4 program by the Neighbor-Joining method. Branches with less than 50% bootstrapping support were condensed. The abbreviations of species names are as follows: At, *Arabidopsis thaliana*; Pt, *Populus trichocarpa*; Os, *Oryza sativa*; Vv, *Vitis vinifera*; Sb, *Sorghum bicolor*; Pp, *Physcomitrella patens*; Sm, *Selaginella moellendorffii*; Msp, *Micromonas sp*; Osp, *Ostreococcus sp*; Csp, *Chlorella sp*; Vc, *Volvox carteri*; Cm, *Cyanidioschyzon merolae*.

An important question is why the APC is composed of many different subunits, while most E3 ligases are composed of one to three subunits. Most of the APC subunits are conserved in all eukaryotes and remain tightly associated throughout the cell cycle [42,43]. It seems probable that the complexity of multiprotein domains is related to the intricate topology of APC [44,45]. One important domain in the APC subunits is TPR, which consists of 3-16 tandem repeats of 34 amino-acid residues. However, individual TPR domains can be dispersed in the protein sequence [46]. TPRs appear to act as versatile protein-protein interaction domains and it has been hypothesized that the TPR-containing proteins in the APC form a scaffold on which the other subunits assemble [47]. Five APC subunits contain TPR domains: CDC27, APC5, CDC16, APC7 and CDC23. TPR-containing sequences of primitive red algae *Cyanidioschyzon merolae*; green algae *Volvox carteri*, *Chlorella sp*., *Micromonas *sp and *Ostreococcus sp*; the bryophyte *Physcomitrella patens*; the licophyte *Selaginella moellendorffii*; the land plants *Vitis vinifera *and *Sorghum bicolor*; and the poplar, rice and Arabidopsis sequences were used to gain insight into the evolutionary relationship between land plants and algae. A phylogenetic tree was constructed with the MEGA4 neighbor-joining method, employing multiple alignments of 15 CDC27, 11 APC5, 12 CDC16, 9 APC7 and 14 CDC23 genes, with bootstrap analysis of 2,000 replicates to ensure statistical reliability (Figure 1 and Additional file 10). Subsequently, the 61 APC subunits were divided into five phylogenetic groups of ortholog genes. The cladogram obtained shows the *OsCDC23 *genes in the same branch, located in the same group as *S*. *bicolor*. Grapevine CDC23 genes are in the same group as poplar. The cladogram also shows that the poplar and grapevine genes are in the same branches in every group, and this could reveal slow evolutionary rates in woody when compared to herbaceous plants. On the other hand, while Arabidopsis CDC27a is in the same group of CDC27b, it is in a separate branch, and sequence divergence and evolution may reflect the accelerated life cycle of this plant. Interestingly, the APC5 and CDC16 clades do not contain duplicated genes. We have not identified an APC5 sequence in red algae *C*. *merolae*; however, this subunit sequence is not so well conserved among different organisms, and it is possible that the similarity is too low to be identified by BLAST comparison.

The APC7 gene is absent in green algae *Chlorella sp *and *V*. *carteri*, as well as in the red algae *C. merolae*, but it is present in green algae *Ostreococcus sp *and *Micromonas sp*. Chlorophytes (e.g., *V. carteri*) and Trebouxiophytes (e.g., *Chlorella sp*) have apparently lost the APC7 gene, but the Prasinophytes (e.g., *Ostreococcus sp*, *Micromonas sp*) and land plants retained this TPR subunit. These data also suggest that the primitive ancestor of Chlorophyceae and Trebouxiophyceae classes lost APC7 gene during green algae evolution (see Additional file 11). The APC7 gene is also not found in the yeasts, and it could mean that it is not required for APC function in unicellular organisms and it could be a recent duplication of another TPR subunit.

**Figure 2:**
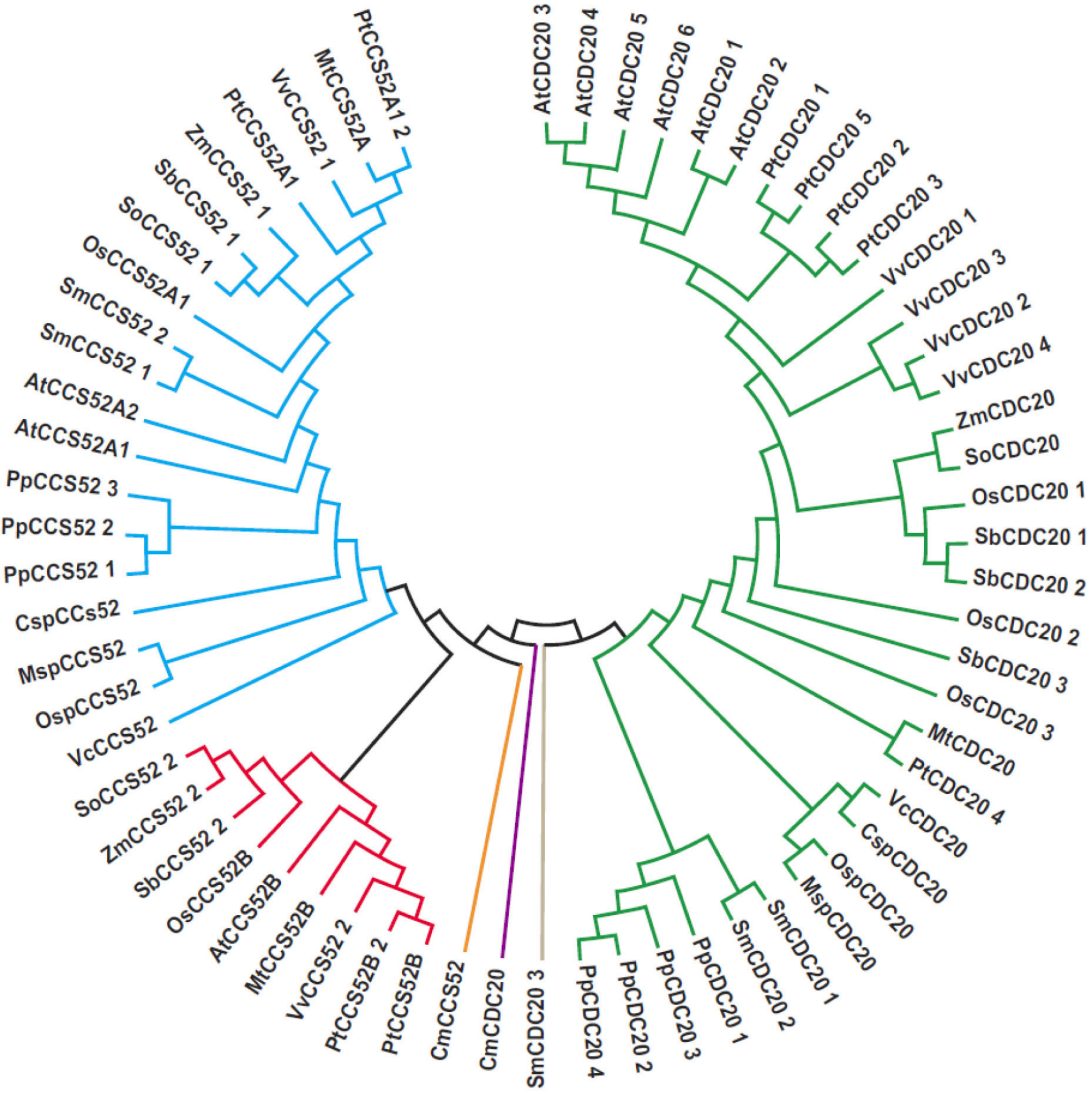
**Phylogenetic relationships of CDC20 and CCS52 proteins from diverse species**. Protein sequences were aligned with ClustalW using the Gonnet scoring matrix in MEGA4. The unrooted tree was generated using the MEGA4 program by the Neighbor-Joining method. Branches with less than 50% bootstrapping support were condensed. The abbreviations of species names are as follows: At, *Arabidopsis thaliana; *Pt, *Populus trichocarpa; *Os, *Oryza sativa; *Vv, *Vitis vinifera; *Zm, *Zea mays; *So, *Saccharum officinarum; *Sb, *Sorghum bicolor; *Mt, *Medicago truncatula; *Pp, *Physcomitrella patens; *Sm, *Selaginella moellendorffii; *Msp, *Micromonas sp; *Osp, *Ostreococcus sp; *Csp, *Chlorella sp; *Vc, *Volvox carteri; *Cm, *Cyanidioschyzon merolae*.

The Arabidopsis genome codes for six CDC20 genes and three CCS52 genes. In addition, CCS52 genes have been divided into two types (A and B) on the basis of functional and sequence analysis [27]. To gain an understanding of the evolutionary relationship between activators from land plants and algae, phylogenetic analysis was performed for activators from plants and algae genomes (Figure 2 and Additional file 10). We used EST sequences from *Zea mays*, *Medicago truncatula *and *Saccharum officinarum *to better estimate divergence between monocot and dicot sequences. The obtained phylogenetic tree reveals three major class groups. Three genes, *CmCDC20*, *CmCCS52 *and *SmCDC20_3*, are isolated from the others and thus need to be treated as nonconserved sequences. One clade groups all CDC20 sequences (except *CmCDC20 *and *SmCDC20_3*), while two other clades included the CCS52A and CCS52B sequences (except *CmCCS52*). The CDC20 clade is divided into two subfamilies: one with *S*. *moellendorffii *and *P*. *patens *and another with algae and land plants. The number of CDC20 copies varies according to species. Arabidopsis has six copies, rice three, poplar five, grapevine four, sorghum three, *S*. *moellendorffii *three and *P*. *patens *four. The CCS52A clade contains more duplications than the CCS52B clade. All algae CCS52 genes, two copies of *PtCCS52A*, *AtCCS52A and SmCCS52*, and three copies of *PpCCS52 *genes are grouped into the CCS52A branch.

Curiously, only one copy of the CDC20 and CCS52 (possibly a primitive A-type) genes was found in algae genomes. The absence of CCS52B type genes in algae, *P. patens *and *S*. *moellendorffii *is interesting because these species are at the base of plant evolution where divergence into A-type and B-type has not occurred yet. Like Arabidopsis, poplar has two copies of CCS52A; however poplar has an additional copy of CCS52B. Analyses of poplar gene evolution must take into account the most significant event in the recent evolution of the genus: a genome-wide duplication event at approximately 65Mya and is still detectable over approximately 92% of the genome [41]. Based on the age estimates of duplicate genes and homology microsynteny analyses, CDC20 and CCS52 gene pairs are represented within segmental duplication regions associated with the recent salicoid duplication event.

### Comparison of Arabidopsis, rice and poplar TPRs proteins and determination of orthologous relationships

Progress in comparative genomics allows assessing the impact of gene and genome evolution on the appearance of novel biological functions and their effect on organismal complexity [48]. The importance of gene duplication in supplying raw genetic material for biological evolution has been recognized for decades and is still extensively studied. Gene duplication generates functional redundancy, which allows two identical genes resulting from the duplication to accumulate mutations with relaxed selection pressure [49,50]. Differentiated or novel function may be achieved after a period of evolution. Segmental duplication (tandem duplication of a genomic segment) is the most prevalent way to generate redundant genes [51]. Segmental duplication can also happen on a smaller scale, resulting in duplicated exons, rather than an entire gene [52]. These segmental duplication blocks can create protein fragments (referred to as domain) that have structure and function [53].

**Figure 3:**
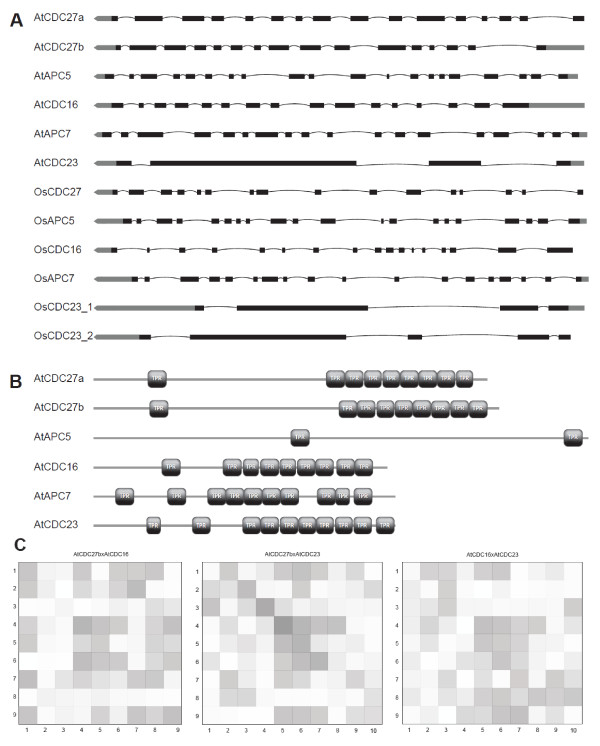
**Analysis of Arabidopsis TPR subunits.** A, Diagram of exon-intron structure of Arabidopsis and rice TPR subunit genes. Exons and introns are represented by black boxes and lines, respectively. Thick gray lines correspond to the untranslated regions. B, Schematic representation of TPR domains on Arabidopsis TPR subunit sequences. TPR domains and full-length proteins sequences are represented by black boxes and gray lines, respectively. C, Pattern of internal domain duplications in Arabidopsis TPR subunits. The intensity of shading reflects the alignment score, with a dark shading for higher scores. The numbers on each axis indicate the domains in N-to-C terminal orientation within the repeat.

The TPR domain consists of a 34-residue repeat that adopts a helix-turn-helix conformation, which is associated with protein-protein interactions [54]. The subunits CDC27, APC5, CDC16, APC7 and CDC23 have TPR domains, but the total number of TPR repeats and the position in each sequence is variable. Exon shuffling may be responsible for internal duplications in repeats and we decided to investigate the exon-intron organization of TPR genes in Arabidopsis, rice and poplar (Figure 3A) [55]. The number and position of exons and introns in the genomic sequences are remarkably conserved in some of the APC subunit genes. The CDC27 gene has two homologs in Arabidopsis and poplar, and only one in rice. Interestingly, the CDC27 gene maintains 16 exons in monocot and dicots. The APC5 and APC7 genes have one copy in all genomes and also maintain 20 and 18 exons respectively, in the three genomes. On the other hand, the CDC23 gene has two homologs in rice and only one in Arabidopsis and poplar, but the most significant information is the lower number of exons when compared with other TPR-containing genes. The *OsCDC23_2 *has 5 exons (exon-intron annotation in Additional file 12) and *AtCDC23*, *PtCDC23 *and *OsCDC23_1 *have 4 exons. Examining the CDC16 gene we identified 15 exons in poplar, 16 exons in Arabidopsis and 17 exons in rice. We next compared the exon organization of all TPR genes from Arabidopsis using domain prediction. Nine TPR repeat sequences were identified in *AtCDC27a *and *AtCDC16 *sequences, two in *AtAPC5 *and ten in the *AtAPC7 *and *AtCDC23 *sequences (Figure 3B). *AtCDC27b *has the same number of TPR domains as *AtCDC27a*. Tandem arrangement of TPR domains occurs predominantly at the C-terminal of *AtCDC27a*, *AtCDC27b*, *AtCDC16*, *AtAPC7 *and *AtCDC23 *sequences. *AtAPC5 *has only two TPR domains, one in the middle of the sequence and another at the C-terminal. The results show that TPR tandem repeat topology on protein sequences is at least partially related with the exon organization of TPR subunit genes.

In the APC subunits, the tandem arrangement of TPR domains suggests that the elements evolved by duplication followed by primary sequence divergence. Because TPR are conserved structure repeat elements which are often divergent at the primary sequence level, TPR domain sequences can diverge a great deal over time and have poor consensus motifs. An approach to investigate the evolution of TPR domains in the subunits is to identify patterns of TPR duplication from the alignments. Distinct patterns of repetition can often be distinguished, and we tested whether the Arabidopsis TPR proteins have defined internal repetition blocks. The sequences of TPR proteins were aligned to each other or in pairs using the methodology described in [56]. The pairwise sequence similarities between all repeating domains in a protein were examined using the Smith-Waterman algorithm to identify patterns of duplication from the alignments [57]. The alignment scores between the domains were displayed in a matrix (Figure 3C and in Additional file 13). Comparing the proteins to themselves, there are similarities among domains 4-6 in *AtCDC27; *and among domains 5-9 in *AtCDC16*. *AtAPC7 *has a pattern for domains 1-2, *AtCDC23 *has patterns of similarities for domains 6-8; and no pattern was found to for *AtAPC5*. Comparing the proteins in pairs, we can observe a diagonal match between *AtCDC27 *domains 2-6 and *AtCDC23 *domains 3-7. There are also indications of similarities for the pairs *AtCDC27 *and *AtCDC16*, *AtCDC16 *and *AtCDC23*. Although there are no unambiguous duplication patterns for any of the proteins, the results suggest that TPR domains in the APC genes have evolved by duplication of the central elements, or that higher constraints are imposed to changes in the primary sequence of these repeats.

### Expression analysis of APC subunits and activators genes

**Figure 4:**
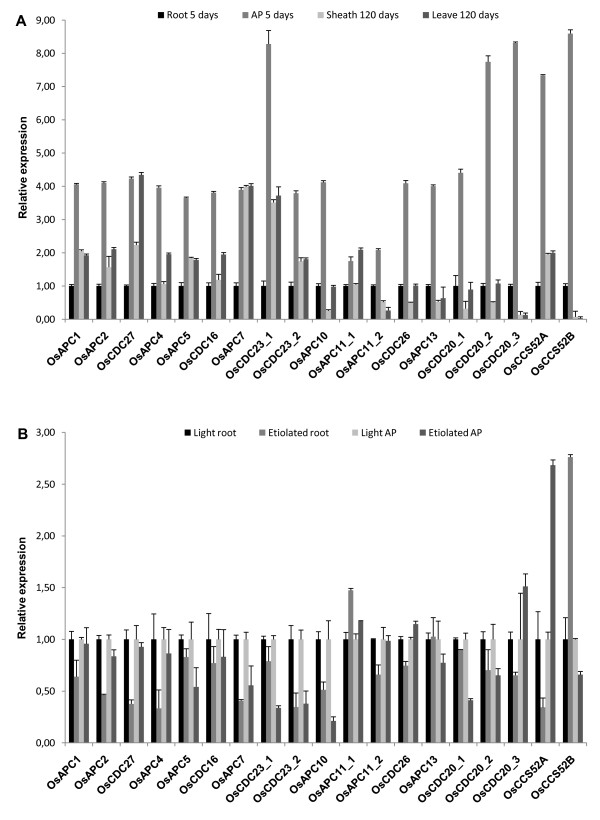
**Relative expression profile of rice APC subunits and activators**. The transcript level is represented as a ratio of absolute value of the studied gene to the absolute value of Os28 S gene. Color codes of the bars are indicated at right figure. A, APC subunits and activator genes expression profile in different tissues. Expression levels obtained are normalized to root. B, Expression pattern of APC subunits and activators in darkness stress. Etiolated root and aerial part are normalized to corresponding control light root and aerial part respectively. Error bars indicate mean ± SD of three independent experiments. Data are representative of two independent biological replicates.

Gene duplication can be a source of innovation for the increased developmental complexity of plants. Expression patterns can provide important clues for gene function under specific conditions. We examined the expression of the APC subunit and activators genes in 5-day old rice roots and shoots, and in sheath and blade of mature tissues (Figure 4A). As expected, there are higher levels of gene expression in tissues with high proliferation rates, although there is more mRNA in shoots than in roots. However, a complex pattern is observed when their expression is examined in mature blade. In general, there is a general decrease in expression for several genes, as expected. However, the degree of reduction is variable. The mRNA levels of *OsAPC1*, *OsAPC2*, *OsAPC4*, *OsAPC5*, *OsCDC16*, *OsCDC23_2*, *OsAPC10*, *OsAPC11_2*, *OsCDC26*, and *OsAPC13 *are reduced in both sheath and blade compared to aerial part. On the other hand, *OsAPC11_2 *and *OsCDC27 *mRNA levels are reduced only in the sheath, but not in the blade. Finally, there is no reduction of *OsAPC7 *mRNA levels in both sheath and blade. In contrast, expression levels of activator genes are markedly reduced in mature leaves. However, although CCS52A mRNA levels are lower in both sheath and blade, the reduction is less pronounced. In Medicago, the CCS52A gene has been implicated in control of endoreduplication in nodules [58]. Recently, it has been shown that the CCS52A gene is important for maintenance of meristem activity in roots of Arabidopsis [59]. Therefore, it is possible that the CCS52A could form a specialized complex with some of the APC subunits and assume a unique function in mature leaves.

Growth response to dark is part of an integrated developmental change throughout all the plant organs [60]. In Arabidopsis, hypocotyl cells undergo up to two rounds of endoreduplication in light-grown seedlings, whereas an additional round can be observed specifically during dark-grown development [61,62]. The endoreduplication regulatory mechanism is thought to be common to the G_1_-S transition of the mitotic cell cycle [63]. G_1_-S transition is important for CDK inactivation by cyclin proteolysis, mediated by APC^CDH1^, which then maintains cyclin instability in G_1 _and enables a new round of DNA replication by allowing the assembly of pre-replication complex (pre-RCs) [64]. Almost all rice APC subunits have similar expression patterns, both in roots and shoots (Figure 4B). However, *OsAPC11_1 *and *OsCDC26 *mRNA levels increased in darkness. Interestingly, the two *OsCDC23 *genes are differentially expressed in etiolated roots. While *OsCDC23_1 *mRNA levels did not change in roots grown in the dark or light, *OsCDC23*_2 mRNA levels in roots were much lower when rice plants were grown in the dark. Similarly, in etiolated roots *OsAPC11_1 *mRNA levels increased while *OsAPC11_2 *levels decreased. These results could indicate that, following gene duplication, the APC11 and CDC23 genes are assuming new functions in rice.

The activators *OsCCS52A *and *OsCCS52B *exhibited high levels of mRNA in darkness and *OsCDC20_3 *showed a lower level. One hypothesis for the high mRNA levels of CCS52 genes is that the APC^CCS52 ^acts as a negative regulator of CYCA2;3/CDKA;1 complex in Arabidopsis [65]. The CYCA2;3/CDKA;1complex acts as a negative regulator of endocycle and the APC activation by CCS52 may increase CYCA2;3 degradation and consequently the progression of endocycle in darkness [65].

**Figure 5:**
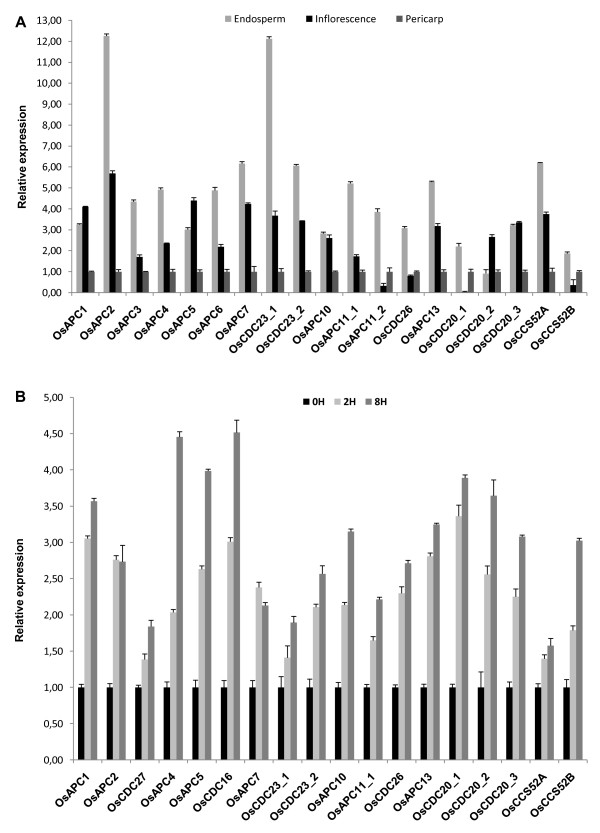
**Gene expression analysis of APC subunits and activators in rice**. The transcript level is represented as a ratio of absolute value of the studied gene to the absolute value of Os28 S gene. Color codes of the bars are indicated at right figure. A, APC subunits and activator genes expression profile in endosperm, inflorescence and pericarp. Expression levels obtained are normalized to pericarp. B, Expression pattern of APC subunits and activators in auxin treatment (NAA). Seedlings treated are normalized to control 0 hour. Error bars indicate mean ± SD of three independent experiments. Data are representative of two independent biological replicates.

To investigate the expression of the APC genes and activators during rice seed development, we examined the expression of the APC subunits and activators in endosperm, pericarp and inflorescence tissues (Figure 5A). Expression levels were measured in the inflorescence at reproductive stage 3 (before anthesis, 70 days after emergency), pericarp and endosperm at reproductive stage 6 (milk stage, 90 days after emergency). Levels of mRNA from all APC subunits were higher in the endosperm, except *OsAPC1 *and *OsAPC5*, which were present in higher amounts in the inflorescence. The duplicated *OsCDC23 *genes displayed similar expression profiles in the seed tissues examined. However, the *OsAPC11 *gene seems to be differentially expressed; *OsAPC11_2 *showed reduced amounts of mRNA in the inflorescence when compared to the pericarp and endosperm. Using the Genevestigator Analysis tool, we compared the expression levels of the APC genes with qRT-PCR results (see Additional file 14) [66]. Although only the genes Os*APC2*, Os*APC7*, Os*APC10 *and Os*APC13 *are present in the microarray slides, their expression is similar to what we observed in this work.

The expression patterns of the various APC activator genes were analyzed. The *OsCCS52B *transcript was expressed at low levels in the inflorescence and *OsCDC20_1 *was barely detected in the same tissue. Interestingly, transcripts for *OsCDC20_2 *and *OsCDC20_3 *were detected in inflorescence, a tissue with a high rate of proliferation, suggesting their involvement in mitotic activities [67]. The *OsCCS52A *had high levels in the endosperm and this may be explained by the fact that the transition from a mitotic to an endocycle in endosperm cells involves a spike in activity of the master cell cycle regulators belonging to the CDK family and APC complex, which subsequently decreases as endocycle progresses [68]. These contrasting expression patterns for *OsCCS52 *and *OsCDC20 *could suggest distinct functions for these APC activators during seed development.

Based on the direct relationship between the increase of auxin and cellular proliferation we treated rice seedlings with naphthaleneacetic acid (Figure 5B). After treatment for 2 and 8 hours, all APC and activator genes increased their expression. This result indicates that the rice APC subunits and activators might be involved in the regulation of the cell division events triggered by auxin in the seedlings.

We analyzed the expression in leaves of *PtAPC10*, *PtCDC27_1*, *PtCDC20_1*, *PtCCS52A1_1 *and *PtCCS52B *genes by qRT-PCR (see Additional file 2). Interestingly, *PtCCS52B *exhibited lower mRNA levels when compared to *PtCCS52A1_1*. Recent data suggest that the *MtCCS52B *may have specific roles in M-phase progression, alternative or complementary to those of *MtCCS52A *[69]. Thus, CCS52A might have major roles in post-mitotic, differentiating cells, in which degradation of specific APC targets could contribute to differentiation of given cell types, tissues, or organs [70].

## Conclusions

Through genome-wide bioinformatics analyses of the complete genomes of Arabidopsis, rice and poplar, we identified ortholog genes of APC subunits and activators. In addition, their occurrence was demonstrated in algae, bryophyte and other monocotyledonous and dicotyledonous species as well. A distinctive feature of these genomes is the presence of characteristic duplication patterns (see Additional file 8). In Arabidopsis, only the CDC27 gene is duplicated. Rice and poplar have two duplicated subunits, CDC23-APC11 and CDC27-APC13 respectively. Among other plant genomes, grape and *P. patens *have two copies of the CDC23 and CDC27 genes, respectively, and grape has two copies of the APC13 gene. The two isoforms of CDC23 and APC11 genes in rice are differentially expressed in light-dark grown plants and one of APC11 subunits is also more expressed in the inflorescence than the other. In Arabidopsis, the duplicated copies of the CDC27 gene exhibit significant sequence differences, and seem to have assumed specialized functions, indicative of the occurrence of APC subcomplexes [23]. While the coding sequences of the rice genes are quite conserved, it is possible that their controlling elements have diverged and that they are assuming new functions in the APC during plant development. Paralogs usually display different functions, whereas orthologs may retain the same function [71]. We tested the frequency of Ka/Ks for CDC27, CDC23, APC11 and activators orthologous genes and found interesting values (see Additional file 15). The CDC23 and CDC27 TPR genes had the highest values (greater evolutionary pressure) and the activators and APC11 subunit lower. These results reveal the importance of maintenance of the amino acids conserved in RING finger proteins and the adapters. Analyses of the promoters of the rice subunit genes revealed different regulatory sites for duplicated genes, suggesting different regulation of their expression (see Additional file 16). As expected, the most common *cis*-acting elements in all genes analyzed have functions related to cell division. Strikingly, *OsCDC23 *and Os*CDC23_1 *and Os*CDC23_2 *do not show any *cis *element in common and only two *cis*-regulatory elements are found in both Os*APC11_1 *and Os*APC11_2*. However, the *OsAPC11 *and *OsCDC23 *gene sequences present very little variation across taxa, possibly due to strong purifying selection.

As in yeasts, APC7 is not present in the green algae *Chlamydomonas reinhardtii*, *V*. *carteri *and *Chlorella spp*. The absence of this subunit at the root of animal and land-plant evolution suggests that this subunit may have appeared after a gene duplication event of a TPR-containing subunit gene, and it may not be essential for APC activity in unicellular organisms. Curiously, it might be the case also in later metazoans, once Drosophila APC7 knockout mutant flies and null mutants are viable and fertile [72].

The activators CDC20 and CCS52 have many copies in plants. This is in sharp contrast with the situation in metazoans, where only one copy of each regulator is present per genome. Due to the duplications, Arabidopsis contains three CCS52 activators; CCS52A, CCS52A1 and CCS52B. In addition, six CDC20 genes were found, but At*CDC20_6 *is probably a pseudogene because the important motif KEN-box is absent [12,73,74]. In poplar, the CCS52A and CCS52B genes are also duplicated. Five CDC20 genes are present in poplar, the same number found in Arabidopsis. Interestingly, rice contains one copy of the CCS52A and CCS52B genes; and three copies of CDC20 genes. This suggests that the duplication of the CCS52 genes occurred in dicotyledonous plants after the separation from monocotyledons. Recent duplication of the CDC20 genes has occurred also in Arabidopsis and poplar.

Plants can adopt dramatically different alternative developmental pathways and must integrate cell-cycle progression, growth and development in response to environmental cues. It is believed that multiple members of a specific gene family of a particular organism are the natural products generated from the long evolutionary history that the organism experienced [75]. The number of members of a gene family reflects a succession of genomic rearrangements and expansions due to extensive duplication and diversification that occurred in the course of evolution. Phylogenetic analysis of the APC subunits and activator proteins is very illuminating. The fact that all subunit types except APC7, were found in all plant genomes analyzed, including red algae, green algae and other primitive organisms, suggests that the complete set of genes encoding the APC subunits was already present in the common ancestor of plants and animals. The activators descend from a common ancestor and correspond to well-conserved structures. In all algae species analyzed, there is only one copy of CDC20 and CCS52 genes. Land plants have amplified the number of activators and this fact may be associated with substrate specificity and or with the complexity of their developmental programs.

By comparing the similarity between different TPR domains within one protein it might be possible to trace the evolutionary history of the internal duplications. The alignment scores between domains showed limited similarities among TPR subunits of *AtCDC27*, *AtCDC16*, *AtAPC7*, *AtCDC23 *and *AtAPC5*. The sequence of TPR subunits can diverge a great deal over time, limiting their consensus motifs and making it difficult to detect unambiguous duplication patterns for any of the proteins. Nevertheless, pairwise comparisons indicated greater similarity for the central domains, especially between CDC27 and CDC23, CDC27 and CDC16, and CDC23 and CDC16. In all cases, higher similarity scores between TPR subunits 4 in CDC16 and CDC27, and TPR subunit 5 in CDC23 were detected, suggesting that the TPR tandem evolution most likely started from the middle domains. Because the primary amino acid sequence of TPR subunits tends to diverge, the higher degree of similarity among the central subunits may also indicate that stronger evolutionary constraints are applied to these regions.

Previously, we have shown that the Arabidopsis subunit genes are differentially expressed according to whether the plants are grown in the dark or under light. When Arabidopsis plants were grown in the dark, only *AtAPC1*, *AtAPC2 *and *AtCDC16 *increased mRNA levels [21]. However, qRT-PCR expression profiles of rice APC and activator genes showed spatial modulation of gene expression of the duplicated subunit genes in different tissues (root and aerial parts) of plants grown under different light treatment. *OsAPC11_1 *mRNA accumulates to higher levels in etiolated roots when compared with *OsAPC11_2 *mRNA, and *OsCDC23_1 *mRNA is present in etiolated roots at almost twice the levels of *OsCDC23_2*. Likewise, the rice *OsAPC11 *genes are differentially expressed in the inflorescence, with activators that have very complex expression patterns. Altogether, these data suggest that the duplicated rice APC subunits and activators could participate in different APC subcomplexes and that they may have assumed new specialized functions as they diverged during evolution.

Most E3 ligases are composed by one or two polypeptides [76]. In contrast, the cell cycle regulators SCF and APC are formed by a larger number of subunits. However, for both complexes, biochemical activity can be achieved by only two subunits, a Cullin related protein together with a RING finger protein. In both cases, substrate specificity is defined by the adaptor subunit. The human genome contains 69 F-Box proteins - the SCF activator - and two APC activators, CDH1 and CDC20 [77,78]. In plants, SCF-related complexes are involved in many physiological processes and approximately 700 F-box proteins have been identified in Arabidopsis [79]. The number of APC activators have also suffered an expansion in the higher plant lineages, though a more modest one, the number varying from nine in poplar and Arabidopsis to six in rice. We and others have shown that differential expression of APC subunits could be a source of complexity in regulation of the APC [21,23]. The results presented here suggest that gene duplication, followed by sequence divergence and differential expression, could be another tier of regulation of ubiquitin-mediated proteolysis mediated by specific APC complexes composed by particular subunit isoforms. Future studies employing functional genomics approaches will be required to define the impact of duplications or splicing variants on cell-cycle progression at the cellular level and the associated plant developmental processes at the whole-organism level.

## Methods

### Gene Identification and Chromosomal Location

Searching of multiple databases was performed to find members of APC, CDC20 and CCS52 in Arabidopsis, rice and polar. The strategy to obtain each gene in a genome was the following; Arabidopsis sequences have been published [80], but additional searches were carried out against the TAIR8 database http://www.arabidopsis.org. The sequences obtained were then used as queries to search against the following databases: *Oryza sativa *L. cv. Nipponbare - The Institute for Genomic Research (TIGR) http://rice.plantbiology.msu.edu, *Populus trichocarpa *Nisqually-1 - DOE Join Genome Institute http://genome.jgi-psf.org/Poptr1_1/Poptr1_1.home.html, *Sorghum bicolor *BTx623 - DOE Join Genome Institute http://genome.jgi-psf.org/Sorbi1/Sorbi1.home.html, *Vitis Vinifera *PN40024 - Genoscope http://www.genoscope.cns.fr/externe/GenomeBrowser/Vitis/, *Ostreococcus sp *RCC809 - DOE Join Genome Institute http://genome.jgi-psf.org/OstRCC809_1/OstRCC809_1.home.html, *Volvox carteri *- DOE Join Genome Institute http://genome.jgi-psf.org/Volca1/Volca1.home.html, *Chlorella sp*. NC64A - DOE Join Genome Institute http://genome.jgi-psf.org/ChlNC64A_1/ChlNC64A_1.home.html*Micromonas sp *NOUM17 (RCC299) - DOE Join Genome Institute http://genome.jgi-psf.org/MicpuN3/MicpuN3.home.html, *Physcomitrella patens *- DOE Join Genome Institute http://genome.jgi-psf.org/Phypa1_1/Phypa1_1.home.html, *Selaginella moellendorffii *- DOE Join Genome Institute http://genome.jgi-psf.org/Selmo1/Selmo1.home.html and *Cyanidioschyzon merolae *http://merolae.biol.s.u-tokyo.ac.jp databases using the BLASTP and TBLASTN programs. In addition, Arabidopsis APC and activator sequences were used to search against *Zea mays*, *Medicago truncatula *and *Saccharum officinarum *EST databases at Gene Index DFCI http://compbio.dfci.harvard.edu/tgi/cgi-bin/tgi/Blast/index.cgi. Sequences with significant similarity to Arabidopsis proteins were downloaded into our database and annotated accordingly. Sequence conservation analysis of APC and activators genes was conducted using the BLAST tool with default curve calculation parameters; amino acids sequence "conservation similarity" of 50%. Chromosomal locations of APC subunits and CDC20/CCS52 genes were obtained using the BLAST server and additional physical localization tools of each genomic browser.

### Identification of Protein Domains

The SMART database http://smart.embl-heidelberg.de was used to confirm whether each predicted proteins sequence maintains conserved domains and motifs. The TPRpred http://toolkit.tuebingen.mpg.de/tprpred search program was used to confirm the TPR domain in specific sequences [81].

### Phylogenetic Analysis

Phylogenetic analysis of TPR subunits and activators CDC20 and CCS52 was carried out using the Neighbor-Joining method in the Molecular Evolutionary Genetics Analysis software package - MEGA4 [82]. Alignment for tree construction was done using ClustalW with the Gonnet scoring matrix. Reliability of the obtained trees was tested using bootstrapping with 2,000 replicates.

### Exon-intron and TPR Domain

Exon-intron information was obtained from TAIR, TIGR and JGI databases. The sequences of the repeating domains were extract and aligned to each other using the Smith-Waterman alignment tool in the EMBOSS package and default parameters [56,57]. This gave pairwise alignment scores between all individual domains in a repeat.

### Plant Materials

Rice seeds (*Oryza sativa *L. cv. Nipponbare) were disinfected with 5% sodium hypochlorite for 20 min and thoroughly washed with water. For darkness treatment, seedlings were grown hydroponically (0.5X Hoagland's solution) in a greenhouse at 25-28°C for 2 weeks. The control group was grown with a photoperiod of 12 h light/12 h dark and the etiolated group was grown in darkness. For auxin treatment, seedlings were grown in Petri dishes (0.5X Hoagland's solution agar) in the greenhouse at 25-28°C for 2 weeks. Seedlings were transferred to new Petri dishes and incubated for 2 and 8 h in either water or 2 μM NAA solution. Harvested seedlings were frozen in liquid nitrogen immediately and store at -70°C until RNA isolation. Leaf poplar RNA was provided by Rodrigo T. Lourenço (Forest Biotechnology Group, North Carolina State University).

### Promoter analysis and significance calculations

To identify promoter regions of APC subunits, 1000 bp of sequence preceding each annotated gene from RAP-DB database were extracted manually. These putative promoter sequences begin immediately upstream of the 5' UTR for transcription units with an annotated 5' UTR. To identify overrepresented promoter elements, the putative promoter regions upstream of APC subunits were analyzed. Known plant promoter elements and their annotation were downloaded from PLACE (A Database of Plant Cis-acting Regulatory DNA Elements) http://www.dna.affrc.go.jp/PLACE/[83]. For each promoter, the null distribution for each PLACE motif was modeled by counting the number of occurrences for each word within each of all rice gene promoters (1,000 surrogates) according to [84]. This approach provided an opportunity to determine the level of consistency between the observed frequencies in a single promoter motif with that across all other sequenced gene promoters. A one-tailed p value was estimated for each motif based on the Z score of the difference of the actual word count for each promoter (Ctrue) minus the mean count from the 1,000 surrogates (Csurr) relative to standard deviation (SD) from the 1,000 surrogates (SDsurr) [i.e., Z = (Ctrue_Csurr)/SDsurr]. For each motif the p value calculated was the probability to the right of the observed count calculated on the null distribution from all promoters from the genome. If this probability was less than 5%, the motif was considered significantly overrepresented. These calculations were implemented using Delphi scripts.

### Data Analysis for Ka/Ks

The ratio of nonsynonimous to synonymous nucleotide substitution rates (Ka/Ks) was calculated as described by Siltberg and Liberles [85]

### Gene Expression Analysis

*Oryza sativa *japonica subspecies was used to prepare all generic material. Total RNA was extract from materials according to [86]. After treatment with RNAse-free DNase I (0.5 u/μg RNA), total RNA (2.5 μg) was transcribed using random hexamer primers according to the manufacturer's protocol (Applied Biosystems). The cDNA was amplified using Taqman^® ^Reverse Transcription Reagent kit (Perkin-Elmer Applied Biosystem) on the GeneAmp 9700 thermocycler (Applied Biosystems) under standard conditions. Transcript levels were determined by qRT-PCR using a 7500 Real-Time PCR System (Applied Biosystems). For poplar gene expression analysis, cDNA synthesis and qRT-PCR were performed as described for rice. The data were first normalized to the level of expression of *Os28 S, OsActin and PtActin *for each RNA sample. Primers used for real-time RT-PCR were designed in gene specific region using Primer Express V3.0. Gene primer sequences used in the qRT-PCR analysis are listed: Os28 S forward (F) 5' GCGAAGCCAGAGGAAACT 3', Os28 S reverse (R) 5'GACGAACGATTTGCACGTC 3', OsActin F 5' CTTCATAGGAATGGAAGCTGCGGGTA 3', OsActin R 5' CGACCACCTTGATCTTCATGCTGCTA 3', OsAPC1F 5' CTTGAGCTCTGCTTGCATCT 3', OsAPC1 R 5' GCTTACAGCCATCTGCAGTC 3', OsAPC2 F 5' AATGCTGGGGACAATCTTCT 3', OsAPC2 R 5' TAATGGGTCTGCTTCCACAG 3', OsCDC27 F 5' AGCGACTTGCTACCTTCACA 3', OsCDC27 R 5' TTGACAGGACACAAGGCTTC 3', OsAPC4 F 5' CGACAAGGATGGCCTGTTAT 3', OsAPC4 R 5' GAAGCGCTTGAAAATTCCTG 3', OsAPC5 F 5' TTCGTTGGTCTATGCAACCT 3', OsAPC5 R 5' AACGGGAACTTCTCTTCAGC 3', OsCDC16 F 5' ACAGGAGGAGGGTGATCAAG 3', OsCDC16 R 5' GATTTTGCTTGCGTGAAGAA 3', OsAPC7 F 5' AGACTTCAGGGGAGCTCAAG 3', OsAPC7 R 5' TTGAGAGCTTTTGCAGACTGA 3', OsCDC23_1 F 5' CGCTGAAGCTTAATCGAAAGT 3', OsCDC23_2 R 5' GACCAAGACCATACCAAGCA 3', OsCDC23_2 F 5' CAGTTCTGGTGGAATCTGTCA 3', OsCDC23_2 R 5' CAAGATGTGCACTAGCAAGGA 3', OsAPC10 F 5' TGATCCCCGAGAAACATTC 3', OsAPC10 R 5' GAAGTGAAGTGAAATGGCTGAT 3', OsAPC11_1 F 5' CCAGGATGAAACCTGTGGTA 3', OsAPC11_1 R 5' GTTTGAGAATTGACCCACTTGA 3', OsAPC11_2 F 5' AAACATGCGGCATATGCA 3', OsAPC11_2 R 5' AAGTGGCGTAGATGTCTGAGAA 3', OsCDC26 F 5' ATCGGCCTCCCTACCAT 3', OsCDC26 R 5' GAGGAGAGGCTAGGGTTTGG 3', OsAPC13 F 5' TTGTGCTGGTTGGCTTTC 3', OsAPC13 R 5' CGTCCTCATCGACATCGT 3', OsCDC20_1 F 5' ATGATCGGTGCATCAGGTT 3', OsCDC20_1 R 5' GGCAAAGAACACGAGCAGT 3', OsCDC20_2 F 5' CCTGTCCGGAATAAACCTGT 3', OsCDC20_2 R 5' CACTCGATCTCATCGGAGAA 3', OsCDC20_3 F 5' TTCACATTTGGGATGTGTCC 3', OsCDC20_3 R 5' TACCACCACCTCCAGTTGC 3', OsCCS52A F 5' GCCCCAGGAAGATCCCTA 3', OsCCS52A R 5' TGCTGCATGCATTCCATAA 3', OsCCS52B F 5' GTCACCAAGCTCTGCGATT 3', OsCCS52B R 5' TCCTCCCATGTTCCTAATCC 3', PtActin F 5' GGTCAAGGCTGGGTTTGCT 3', PtActin R 5' TCGCCAACATAGGCATCTTTT 3', PtAPC10 F 5' ATGACCCTAGGGAAACATTT 3', PtAPC10 R 5' AAAAGGCTGATGCGGAAAAG 3', PtCDC27_1 F 5' TGGCCTTCCAAACCTGTCAT 3', PtCDC27_1 R 5' GGCATGCTAAGATTGGAACCA 3', PtCDC27_2 F 5' AGCCGGATGTTATTTGCAAAA 3', PtCDC27_2 R 5' ATGCCGCTTCAGCTTCATTT 3', PtCDC20_1 F 5' TTTATTCCAAACCGGTCAGC 3', PtCDC20_2 R 5' TCGGTTCATGTTCAAGGATTC 3', PtCCS52A F 5' TTCCCCCTCGAGACCTATTT 3', PtCCS52A R 5' CATTGGGATTGTCCTCCTTC 3', PtCCS52B F 5' CGTATCATCACCCAGAGCAA 3', PtCCS52B R 5' TCGTTGCCTCCTTCTTTAACA 3'. Data were analyzed using 7500 SDS software V1.4 (Applied Biosystems).

## Authors' contributions

MFL carried out the molecular genetic studies, analyzed the data and drafted the manuscript. NBE, CP, RS, CR, TB, LV, AE and ACO carried out experiments and analyzed the data. ASH and PCGF conceived of the study, and participated in its design and coordination and helped to draft the manuscript. All authors read and approved the final manuscript.

## Supplementary Material

Additional file 1 **Text file containing amino acid sequences in FASTA format for APC and activators genes used in these analyses**. Click here for file

Additional file 2 **Confirmation of functionality of Poplar *PtCDC27_2***. A, sequencing of genomic DNA corresponding to *PtCDC27_2 *locus. Red box shows the actual triplet. B, Real-time PCR detection of *PtCDC27_2 *mRNA, as well of *PtCDC27_1*, *PtCDC20_1*, *PtAPC10*, *PtCCS52A1_1 *and *PtCCS52B*. Click here for file

Additional file 3 **Gene Index (partial) sequence of *OsAPC1***. An EST was recovered from the Gene Index Project, showing part of (upper). The coding region was underlined and stop codon was marked in red. This sequence was translated in silico (lower). Click here for file

Additional file 4 **Fragments from 5'region of *PtAPC5 *and fragments from the 3'regions of genes *PtAPC4 *and *PtAPC5 *were found in the EST database**. The additional sequence of *OsAPC4 *(not present in Arabidopsis nor poplar), product of incorporation of an intron, is boxed in yellow. Differences in 5'region of *OsAPC5 *are also highlighted in yellow. Click here for file

Additional file 5 **Comparison of 5'region of *CDC16 *and *APC11*_*2 *genes between Arabidopsis, poplar and rice**. The additional sequences are boxed in yellow. Click here for file

Additional file 6 **Re-sequencing of 5'region of *AtCDC20_6***. Click here for file

Additional file 7 **Correction of APC activator sequences**. Gray boxes: The conserved APC interacting motifs C-box, CSM, IR-tail, CBM. Differences are boxed in yellow. Click here for file

Additional file 8 **APC subunits in plant genomes and red algae**. Cr, *Chlamydomonas reinhardtii*; Vc, *Volvox carteri; *Csp, *Chlorella sp; *Msp, *Micromonas sp; *Osp, *Ostreococcus sp; *Cm, *Cyanidioschyzon merolae; *Pp, *Physcomitrella patens; *Sm, *Selaginella moellendorffii; Bd, Brachypodium distachyon; *Sb, *Sorghum bicolor*; Mt, *Medicago truncatula; *Vv, *Vitis vinifera*; Cp, *Carica papaya*; At, *Arabidopsis thaliana*; Os, *Oryza sativa*; Pt, *Populus trichocarpa*; Abbreviations: NI, not identified. Click here for file

Additional file 9 **Chromosomal locations of rice and poplar APC subunits and activators**. Chromosome numbers are indicated at the bottom of each chromosome. Paralogs are linked by dashed lines. A, chromosomal positions of genes in rice. B, chromosomal positions of genes in poplar. Seven poplar genes were assigned to scaffolds. Click here for file

Additional file 10 **Neighbor-joining tree inferred from Poisson-corrected evolutionary distances for genes involved in the cell cycle and multiple sequence alignments of plant TPR subunits proteins**. The TPR subunits (A) and activator gene family (B). The abbreviations of species names are as follows: At, *Arabidopsis thaliana; *Pt, *Populus trichocarpa; *Os, *Oryza sativa; *Vv, *Vitis vinifera; *Sb, *Sorghum bicolor; *Pp, *Physcomitrella patens; *Sm, *Selaginella moellendorffii; *Msp, *Micromonas sp; *Osp, *Ostreococcus sp; *Csp, *Chlorella sp; *Vc, *Volvox carteri; *Cm, *Cyanidioschyzon merolae; *Zm, *Zea mays; *So, *Saccharum officinarum; *Mt, *Medicago truncatula*. Click here for file

Additional file 11 **Phylogenetic relationships between Plants and algae**. Click here for file

Additional file 12 ***OsCDC23_2 *genomic sequence**. Exon (yellow boxes), intron and possible exon (gray boxes). Sequencing result - frameshift discarded. Click here for file

Additional file 13 **Pattern of internal domain duplications in Arabidopsis TPR subunits**. The intensity of shading reflects the alignment score, with a dark shading for higher scores. The numbers on each axis indicate the domains in N-to-C terminal orientation within the repeat. Click here for file

Additional file 14 **Expression patterns of rice APC genes based on Genevestigator**. Expression patterns of *OsAPC2*, *OsAPC7*, *OsAPC10*, *OsAPC11_1 *and *OsAPC13 *are shown in different tissues (A) and developmental stages (B). Click here for file

Additional file 15 **Summary statistics for Ka and Ks**. http://services.cbu.uib.no/tools/kaks. Click here for file

Additional file 16 **Promoter analysis**. The upstream 1000 bp regions of all rice APC subunits genes were considered to contain the full length promoters. Table 2, motifs found in the promoters and their occurrences. Table 3, motifs found in each duplicated gene (in red, same motifs). Click here for file
