# Supplementary material for: Genomic evolution and complexity of the Anaphase-promoting Complex (APC) in land plants
Source: BMC Plant Biol. 2010 Nov 18;10:254. doi: 10.1186/1471-2229-10-254 (PMC3095333; doi:10.1186/1471-2229-10-254)
Supplement: Additional file 1 — Text file containing amino acid sequences in FASTA format for APC and activators genes used in these analyses. [file 1471-2229-10-254-S1.PDF]

Additional file 1: Text file containing amino acid sequences in FASTA format for APC and activators genes used in these analyses. Similarity searches on nucleotide and amino acid sequences were taken using BLAST at the NCBI GenBank database (http://www.ncbi.nlm.nih.gov/BLAST/) and all databases in **Methods** - Gene Identification and Chromosomal Location.

```
>OsAPC5 - LOC_Os12g43120
MNVFAGVGGGAAGKEAGTVGGGALLELTPHKLALCHLVQVFAPPPQAGVSAPALPFPFESVAHHNRLGLFLFALTRSCEDFREPPLEEL
LRQLKAVDALVNGWLCEQLTSTLSALTSPDDLFNFFDKLRGVLSAPEGANVEDEFLDPNSQLGVFLRCCILSFNTMTFEGVCHLLANLV
EYCNSADTSYDLAEDEDFNSEMEMSNFMDTNMHVRDGVFDKYNQGYAPRSHMVDSSSSLVHAPASLHDFEEANMFKADDNLGPTCLRSR
WQLEAYLNQQADILEKDPSSVPLNSFNATMSQLQKLAPELHRVQFLQYLNALTHDDYVAALDNLHRYFDYSAGMQGLFSRTASPFQDII
VGKYESALLCLGNLHCYFGHPKKALEAFTEAVRVSQMNNDDSCLAYILGAISNLLSKIGMSSTVGTIGSPYSLGNNIGLGTPLSIQQQL
LVLLKRSLKRADTLKLTSLLSFDHLSLAKFDLKHVQRPLVSFGPNASTKLRTCPADVCKNLRLSSRVLTDFGTDGLSASNDNGSFSTSW
LRNLSAASNSWCSSSKKSGKLLTNDFDNFHFHAQPSPIPASVLQLAGSAYLLRATAWEHYGSAPMVRMNSLVYATCFADAASSSELSLA
YVKLIQHLATFKGYSAAFSALKLAEEKFPLSANSHIQLLKMQLLHERALHRGHLKVAQQICDEFAVLSSSVSGVDIELKTEARLRHART
LLAAKQFSQAANVANSLFSTCYKYNMQVENASVLLLLAEIQKNSDNAVLGLPYALASQSFCKSFNLDLLEASATLTLTELWLALGSTHA
KRALSLVCQSLPMILGHGGLELRARAHIVLAKCYLSDPKFSVSEDPSAVLDPLNQAAEDLEVLEYHEMAAEAYYLKAMVYNNLGKLDER
EEAAASFKEHTLALENPYNEEDSLAC
>AtAPC5 - At1g06590
MAGLTRTAGAFAVTPHKISVCILLQIYAPSAQMSLPFPFSSVAQHNRLGLYLLSLTKSCDDIFEPKLEKLINQLREVGEEMDAWLTDHL
TNRFSSLASPDDLLNFFNDMRGILGSLDSGVVQDDQIILDPNSNLGMFVRRCILAFNLLSFEGVCHLFSSIEDYCKEAHSSFAQFGAPN
NNLESLIQYDQMDMENYAMDKPTEEIEFQKTASGIVPFHLHTPDSLMKATEGLLHNRKETSRTSKKDTEATPVARASTSTLEESLVDES
LFLRTNLQIQGFLMEQADAIEIHGSSSSFSSSSIESFLDQLQKLAPELHRVHFLRYLNKLHSDDYFAALDNLLRYFDYSAGTEGFDLVP
PSTGCSMYGRYEIGLLCLGMMHFRFGHPNLALEVLTEAVRVSQQLSNDTCLAYTLAAMSNLLSEMGIASTSGVLGSSYSPVTSTASSLS
VQQRVYILLKESLRRADSLKLRRLVASNHLAMAKFELMHVQRPLLSFGPKASMRHKTCPVSVCKEIRLGAHLISDFSSESSTMTIDGSL
SSAWLKDLQKPWGPPVISPDSGSRKSSTFFQLCDHLVSIPGSVSQLIGASYLLRATSWELYGSAPMARMNTLVYATLFGDSSSSSDAEL
AYLKLIQHLALYKGYKDAFAALKVAEEKFLTVSKSKVLLLKLQLLHERALHCGNLKLAQRICNELGGLASTAMGVDMELKVEASLREAR
TLLAAKQYSQAANVAHSLFCTCHKFNLQIEKASVLLLLAEIHKKSGNAVLGLPYALASISFCQSFNLDLLKASATLTLAELWLGLGSNH
TKRALDLLHGAFPMILGHGGLELRARAYIFEANCYLSDPSSSVSTDSDTVLDSLRQASDELQALEYHELAAEASYLMAMVYDKLGRLDE
REEAASLFKKHIIALENPQDVEQNMA
>PtAPC5 - Pt592813
AFALTPHKVSVCLLLQTYALPAQTTPPFPFSSVSQHNRLGLYLLALTKMLQSYDDILEPKLEELLNQLKEISGSLGHWLIDHLTSRLSS
LSAPDDLFSFFTEMRGILGGLDSVVMEDNQVILDPNSNLGLFLRRCILTFNLLSFEGLCHLLTNIGSYCKEAMSSSNDLETLSEYENMD
LENFMFGKVNEEIEARKQASERVPFHLHGPKALSGLVEGIIDSSKHGDKCGETSAYVHPPGNELRDVDPYGEIFLRTNWQVQGYLMEQA
DAIEGCRHDSSFSLNSFELVLRQIKKLAPELIQVHFLRYLNSLYHDDYFAALDNLHRYFDYSAGAEGFDSAPSSSGSNSSGRYEIGLIY
LGMMHLHFGHPKQALeVLTEAVRFSQQQSNESCLAYTLAAICNVLSEFGCSSSAGVLGTSFSPITSMDTSLSVGQQLFVLLRESLKRAE
SLKLKRLVASNHLALAKFDLLTSYLFQLYFDLQHVQRPLLSFGPKASMKLRTFPINVCKVFGAFGDSIYSFSPISLLQELRLCSHLISE
FGSESSTMTTDGVFSTTWLNNLPKSMDSPLLPQENAHRNNCDAHRFFTQLSSVPKSVLQLLGSSYIMRSTAWEMYGSAPLARINSLVYA
TCFADASSSSDAASVHAKLIQHLAVFRGYKGAFAALKVAEEKFLTVSKSVILLVKLQLLHECALHRGNLKLAQQVCDELGVLASSVSGV
DKDLKTEASLRHARTLLAANQFSQAAAVAHSLFCMCYKFNMQVQNATVLLLLAEIHKKSGNAVLGLPYALASLSFCQSFNLDLLKASAT
LTLAELWLSLGSNHAKRALTLIHGALPMILGHGGLELQARAQITEAKCYLSDPSYSGSTLSSPFLDLLRQASDELQVLEYHELAAEAFY
LMAHVFDKLGQLERREEAAASFKEHMMALENPQD
>OspAPC5 - e_gw1.06.00.51.1
MERSGKTTEGFVVAARRAWARCATEFLLDTANEGEEREGEGEEEEKGDGARARWRRDGMFEPGPWEMVRRVRALARRAFEDAGDEDAGD
DVERWCVEVFLEELDLATVSLDALERCFEDARAVVLNARVDEFDDALGLEMWGLEEWTPTSTFLKTCHLGFTSAPFEATTELLRTTRAY
LDAAVRVENGIRDGCDWREARREDDAFRACAAPDALMALACRATARYDARGVDADAVLPRHRLDVLAELAPEMPTIHYLKHAEALNRRD
FPAAVEHLHRHFDASGEHVDVRADLGSRRAEGEFESANAGRERLQTALLALATTHFAFSHVNEAMSAISEAVRTAQQNGDETSLAHALA
LTTALMAQTRRGGERDAAQLPTLLRRCAAQAAELSSPHLVAYASLALTKYEIDHPSTAVTGGGDIGESEVVTSTPTRATRALIDVELTR
HAAKLASSTPASTERALAVHRARGDAAVTAGSDVYPTPKGFPSTPASAHAASSVASAMASLTATASVLTSESWHAHGCSHLARMYALRQ
LMHDDEASADDAATSCARLLASTSEREGASATEEVMDIVRDSFGARGERHKTTAVAFLKLEYERAIDRGEYGAARCAARRARALVGFGD
GADAELDFESRRMNANLDRVMQNFDAAQDELRTIIKEAELCGDEHAVMRATLTLAETHLSADAPTLALTRALPLERLAAERGLEPIRAT
VTCIACEAWLALGGSHARLARDTLDERSLALLSSDCLRTQARAYAACARALVATTPECEFPTIARRVVDALERACERYVKLDARCDAAR
AYASLADAHWRVARDATARDAAARRCRAFATEFTDATPTRDDAY
>MspAPC5 - EuGene.0000110072
MAQVYSPTPHRVALCALARYLVHSHARDERAAERLGVSPEQASRLDRPSRRAVDPSTGDRLGPCVLGPADARRLAAVLMREASRSDGFR
EPDLRAFRRSLRRHPILVRETHRHPDDDDDEDDDDEDPMAAYTDAYPYSRETFADLLDDAIASLRGVDSVAALIQEVAPRSRHEAEMRG
AHDDDDDPSHPGGSYDPFPPGPGAAEESSSLGLFLRRCVADFEAQSFEGSVRVFGVFAAYVREGAGEYDDVDAAAARDRMDVVDKRLRV
DARQAAAEAAVGEGGVEVHVDLRGDDEDDATLRRLGALHGSHLAPLTLDPRPGNATGPSQGGGVSRVPSGSEQHSSVRALDLSAASMTQ
IRDGLRDVAVRPDAKLREWIARRCRGVDQRDGYESTRDVTSALDDIERVAPKVPGAELARHLAHVSRRDFTQAMEHARRHFDYLPGTLG
TTYGDGSYADGSYSNDGRAAGGRVALDVGAGTSFGLPMNPRQAFERGREIFGTGAFAGESSLQMNQSGVQAGEATAQEHADAAAAAQRA
RLQSALLTLGVAHFRFHHSGEALKALNEAVRTAQQNGDEASLAHALAAFCALCASTAGSLASTEGAPAVEEWRAAGGDEGDKERRGVSI
STQPPAVQAAADARLLLRRLAKQARTLRIPHLMAYGELARARHGASRPPCGPAPWSRGLETEGGSKKESGKTRDSDLSTADSALLFRRV
ASSPPAAAAAASQLVEALRHAVTLGAAAPQATTQAAAAAAAASRSGADGQRANPNELYPPPKGLALSAGYPSGSESAMEQLSGSGSVLC
GAVWDAHGVPSMARMHALRHLRCDASRRFVRRDDETDADNDPGVVPGEFPESFGGSTRSSHEAEADAASAAASDTAAALAQLARHASTH
HGPDAAADVFAIAAARFPKRRMEEVAADPALAAAAAATDHDAARARGDGDACDAAARRIASLAPASTRTDPEARVEAFRARADACLISG
GLGDAVFHASEAFQGSIREGLTHATLRATLTLAEAHLAAGAPAAALQHALALEHSAAALRLDGLRAAAIVVLAECWLAMSASAAGVGST
STTVGQRRRRDGYASMAKDALDAHAPALLSRGGLALRARARMASAKAALACRSDDFDSSRMSSRTSSYPDDSTSGGWDEVLVPLEDAVA
CCAALGAHAKEAEAHELMARTFAAMGPNHVAARNAAARRWRECERRRRRAEVGDVGGVGGWGVGVGDGSARFGGIGSFPASRAVAV
```

>CspAPC5 - estExt_fgenesh3_pg.C_140186
MEAVKAADVALCILLRSYLCPTTETDPDPHSPLHALFGEALLREIRRRDEAASPSLMELLQHIQVAAAAGGDHMCPGGESAGEADAYFG
AVKAAVGAHLSALETADELVSLFTSVAEQVITQCTSTPAEAEGRGADASSAMGLYLRICYARYTAMTFEAICKLVGEVHAYFDAAVATL
QGRPSAPAPAPRLRPGPDLERFLNAQLAGLGRRVGSVAQADIEAPLGELAGAAPQVPKAHLAHHLSALHHRDVAASLDHLHRFFDHTAE
ARGAAAGGGSGAAAGQQAGEAAAARERGRLQSAALSLGSMHAQLGHMEQALQALNETVRIAQQSSDDTCLAHALALLCQVLDATTPGTI
TSVSHVPGASPAARHYTQLGQLLHRCLRRCEELHLPHLVAFSQLALARLELLHPQRPAASPGAAGGAEGPAGGGGGTAPDLPCSSSVAV
AGAVRDVAHLHMATRLAAAAPSVPPLSSGASSARVLRGVGDLFASTAALYGPDMQGMQASSAAEVEQLVAGSHLLQAACWELRGSRHLA
QAHSLACLDAFGGVTRAEEQCTALAQLAVSVAAAHGPRAAEQVLAVADERFPGAQSRVLAGARLSIAHDRALRRGDLHAAADIAAQMAA
LPPPTDSTDIGLRLEAEERIARTLLAAGCVEEAAAAAHAACAVASTTGQPLHSARLQLLLARVHQQAGAPMAALPYALTATAHARQLAA
DMLAAEAVVLLAGLWCDMGAQHAQHARRELEGALPSILAHGSLELQAAAQVALAEACMTQHVTPAGLREDGEWLLVLLEEAERVSLVLE
DRRGAAHATYLRALVQDALGNAEQRNAAAASFGALVQPAGALE
>VcAPC5 - fgenesh4_pg.C_scaffold_242000002
MHAGLGHTAEALHALNETMRLAQQCGDPVVLLHALSVLCRLLAAIAPGAPGLPPHGPAALRSSLAHHVQLLRMLRRCRERGRELGQPHL
TAFAQLAAARFAMLHDVEPSDSGLHAPPGSLTAAAAAAAVPPSAGAAPAEAPAGAGGEGELRAAPLMVSCAVRDTHALATAASLSAAAP
AAPPPTAADGTPPPPQRAAAAASDLYSSPLLFDRSLPIGAAAAMADAVQQLAGAAHLLQSAAWALHGHNTLERAHTMMYLAAFSDPQVG
CGTPARFEDRSTACAQLVAAVARRGPAAARAAAAACLGLLAASVCCDGTEDSSPSYSESSAGGGAATAVTAAAAEGSTKASGGWDGGCN
ALALWGGPALAAAWLCAVHDRALALGDSAAAAQLVGQLCALSDPQAHRDVEIRLEAARRGVLNLLAAGATEEAHRAATELFARCADAGL
QAPALRCLMLLAEVHLAAGDPHGAFLHVLACLLPAQPGPGPAGGGVFAAAAAGAAADSSSLGAAVAGGGSGGGRGGRSHDLLAAEALVL
LCRVWYELSDGQQLEEVLVLLQDALPLILAHGSVHLQARAQLVLAEMVMSEASSPADLTHCYSQLQRLLAGAAQAATAAEDFRMAAQAA
CMLAWLHHSQGSVSERDTAAALQETLLDRQAEAEAACASEGRTLHVLRA
>SbAPC5 - Sb03g010430
MSLFAGVGGGAADAASGGGTGRALLELTPHKMAVCHLVQVFAPPAQAGGDVVPPFPFESLAHHNRLGLFLFTLTRSCEDFLEPPLEEFL
RQLKAVDDLANGWFCEQLTSSLSALISPDDLFNFFDKLQGVLTASEGASAEDVFLDPNSQLGVFLRCCILAFNSMTFEGVCHLLADLVM
YCNSTDASYDLAEDEDFNSEMGNLMDADIGSQVGIFDKFHQGYASERHMGESSSALIRAPMSTNDFDDANIFKADGNPTCLRSRWQLEA
YLNQQADILEKDPGSVPLNSFNATMTQLQTLAPELHRVQFLQYLNALCHDDYVASLDNLHRYFDYSAGMQGLFGRSVAQVQDIVVGKYE
SALLCLGNLHCYFGHPKKALEAFAEAVRVSQMNNDDSCLAYVLGAISNLLSKIGISNTVGIITSPYSLGTNIGLGTPLSIQQQLLVLLK
RSLKRADALKLPSLLSFDHLLLAKFDLKHVQRPLVSFGPNASTKLRTCPADVIKNLRLGSRVLTDFGADVLSTSNDNGSFSTSWLRNLS
ATSDSWRRSSMNTKKLHINDFDNFHYHAQPSPVPAPILQLAGSACLLRATAWEHYGSAPMVRMNALVYATCFADAASSSELSLAYVKLI
QQLAVFKGYSAAFCALKLAEKKFPSSTSLHIQLLGMQILHERALHRGHLKVAQQICDEFGVLSSSVSGVDIELKTEFSVRRARTLLAAK
QFSQAAAVANSLFSTCYKYNMQVENASILLLLAEIHKKSDNAILGLPYALASQSFCKSFNLDLLEASATLTLAELWLALGSSHAKKALS
LVYQSLPMILGHGGLELRARAHIVLAKCHLADPKFSVLEDPEAVLDPLNQATEDLQALEYHEMAAEAYYLKAMAYNHLGKLDEREEAAA
RFKDHVTALENPQNEEDSLAY
>PpAPC5 - gw1.198.14.1
FVLTSHKVAMCMLLQAYASPSSASPPFCVLPSSARHRLALFLLDQTRVTDGFLEPTFEELGKELKDDLSDVGGVLFEQLGSRLPLLCTP
EELFQFFQGLKELLAPVSYSAESGRGEDETLLIQPNSLLGQFLRRCILAFNVLSFEGSGRLVVELNAYRWLESSDPRGIFVDKDDMIEG
EFDDEYEYEEDIDDVNMDGIHDLEFRFRGTAAGRRGMQGRQQSGGRNGASAFPVPNAAISGDSGVKTRSLRTVEQVEGFLKEQAGLLEK
GVGQIPKEGLDSNLTQLEKLAPDMMKVHYLRYLNHLQQSDYPATMDDLHRYFDYSAGMGGMSVGGASCDSSVGRFQAGLLSLGSMHAHF
GHVDQAMQALNEAVRIAQQYNDDACLAHALAALCHLLFDVGAANEAYAKGESAGLRDVGAGPSLGIQQQLLLLLRRCLRRSLELKLSQL
VAFSRLALAKFYLKHVRRFSSLGGLENGGELGTSPLEVCKTLRLSPYLLGDSISNGISPHVAISGTTNQQRGNGMNINQPLTAPGTMAG
GAWTSLTGRLGRTSDAVVKLAGTSHLLRAASWELYGSVPLVRVSALIHATCYADVASSDDVLLSYIKLAQHQAAFKGYAAAQSAFEVAA
KRFPAAANSLVRTAQLQLVHDHALYRGELKLAQVACGELAASASPVFGVDMERKTEATIRHIRTLLVAGHLDEAAAVARLLFSQCYKAS
MQLESVLVLLLLAEIHKTADSAVTGLPYALAGLTLCRVFNLDYLQASAKETLAELWLGLGVGHAPRALALLQECLPMVLGHGGLELRAR
TNLALARCFLSDPAFSAESQLAEVLDLLQQAAEEFELLEDYALAGEAFYLQALAYNKWGSVEERNSAAKAFQRCMQAL
>SmAPC5 - e_gw1.30.295.1
MAAGFVLTPHKLSMCVLLQLYAAPPALTPPFPLPAAVRHQLALFLLGLAKACDGFLEPTLEDLGMQLKECLGSIGEVIAEQLASRLLGF
SSPEDLFTFVVLGLRGKLFRGGGNEDSLLVEHNSPLGQFLRRCILSFNILSFEGTCRLLAELDAYRRPALSIGDGNIVAKDSLSCQQAE
GDDDEDEEEDRLEDDEDENVIASVTPVHGRERHGNGRRAFGVPLIPTEGKVNGSFCFVKRVEQVHYLQYLNSIHHGDYPAAMSRLHQYF
DYSVGRFQAGLLTLGSMHAHFGHVTQALQASFSALRIHLTFSMQNNDDACLAHSLAALCHLLSEVGVAAEVTKAGAIGDFDRSIGPQLA
AQQHLLLSLKRCLKRALELKLPNLVAFSRLALAKFDLQVIRISGLTLRISPYLLGDLVHSMSSSGSQRITSGAFGQAPGMPGNTQTGRL
GPLSESLLKLAGSSYLLRSCSWELYGSDPMMRASTLVHAYCYSKSASADDLSVAYVKLAHHLAAHKGYRVALTALEQAMKKFPLHARSS
LRSVQLQFIHSQAINRGNTRLAWVACSELAAMASPVLGVDMELKFEASYRHALTLLACKNYSEAATSAGELFALCYKYDMQLHVVKVLL
LIAEIHKKSGSAVTGLPYVLGSITLSQSLNLDLLHAASRVSLAELWLDLGADHAQRALDLLQQSLPLVLGHGSLELRARTNLCIARCYL
SSTDFSVATAPELVLDPLQLAAEEFMNLEDKDQASEAFYLLATTFNSIGRMEDRDKAAEKFQQCVVEEL
>VvAPC5 - GSVIVT00018868001
MHADIEVSAELKFKHREKTGEASSFAHHMKDTLRGIDPNGGIFLRTNWQIQGYLCEQADAIEKHSCSFPLNAFESILRQLQKLAPELHR
VHFLRYLNNLYHNDYPASLENLHCYFDYSAGAEGFDFVQPTSSASNSFGRYEIALLCLGMMHFHFGHPKQALEVLTEAVRVSQQQSNDT
CLAYTLAAICNLLSAIGISSTTEILGSSYGPVTSIGTSLSIQQQLFVLLRRSLKRADGLKLKRLVASNRLAMAKFGLTHVQRPLLSFGP
KASMKLKTCPVNVCKELRLSSHLISEFSTESSIMITDGVFSTAWLKNLQKPMGSLVLSLENASGANSNAFHFCAQPNSIPGSVLQLLGS
SYLLRATAWEIYGSAPLARINALVYATCFSNASSSADVALAYTKLIQHLAVFKGHREAFAALKLVEEKFCSISKSRILLLKLQLLHERA
LHLGHLKLAQQVCDELGVLASSVTGVDMELKTEASLRHARTLLAANQFGQAAAVAHSLFCMCYKFNLQVENATVLLLLAEIHKKSGNAV
LGLPYALASLSFCQSFNLDLLKASATLTLAELWLSLGSNHAERASILVQGALPMILGHGGLELRSRAYIAEAKCYLSNPSFSVFENSEV
VLDPLRQATEELEILEYHELAAEAFYLIAMVFDKLGQLEEREEAAASFMKHVKALENPQNEQDPLFNIH
>AtAPC7 - At2g39090
MEVPKEQIATLIEHGLYDSAEMLGCFLVSSPTVSAETSPQLKAENLILLGDALFHQREHRRAIHTYKQALHHYTRIPKQSSGISRSSLS
LSTRSSVNASSISAINENEVRFKIASSHFALNETKAAIAEMESVKTRSLEMNILMAKLHRNSGYNRGAIAFYKECLRQCPYVLEAVIGL
AELGVSAKDIISSFTQTSNRSAKVSLDQIDPTRWLQRYVEAQCCVASHAYKGALELFAELLQRFPNNVHLLTETAKVEAIIGKNDEAIM
RFEKVRSIDPYTLTSMDEYAMLLQIKCDYSRLNKLVHDLLSVDHTRAEVFVALSVLWERKDARTALSYAEKSIRVDERHIPGYIMKGNL
LLQAKRPEAAAIAFRAAQNLRSDLRSYQGLVHSYLAFGKTKEALYTAREAMNAMPQSAKALKLVGDVHAFTSSGREKAKKFYESGLRLE

```
PGYLGAVLALAELHLMEGRNGDAVSLLERYLKDYADDSLHVKLAQVFAATNMLQDSLSHFQAALRINPQNEAAKKGLDRLEKQMKGIDP
DATDENDENDVEDVDGDTEEAELM
>OsAPC7 - LOC_Os05g05720
MEAARESMAALVDAGLYDCAQTLGCFLVSSSPASSEAGMSMKVESLVLHGDALHGEKEFRRALSAYKQAMQYSKNIPRQATSNTRSSVT
ATGRSPSPNSSSLAPLNENEVKFKIALCYSALREHREALQEMEGIPSKARTLKMNLMLGKLYRISRNNRAAAVCYKECLRQCPYVFEAI
TALAEMGLSSKEFSLIFSQAPNRGGKAPGDSLDAQRWWNRYVEAQCCIASHDYKGGLDIYLDLMQRFPNNVHILLEIAKVEAIIGRNDE
AIMNFEKARLIDPNIMTYMDEYAILLKSKSDYTKLNKLVHDMLHIDPARPETCLALAALWERKDERKALTYAEKSLRVDDRHITGYIMK
GNLHLLLNRPDLAVTDFRGAQELRADLRSYQGLVRAYLALSKCKDALFTAREAMKVMHQSAKALKLVGDVHAISSSGREKARKFYESAI
RLEPGFLGAALALADLHVAEGRNKDAVLLLERYLRQWTDDSLHIKLAQVFAATNMLSDALSHYQSALRINPHNEAAKKGLERLEKQMKG
VDPDAPEEDEENEADDVDGDQDDAELL
>PtAPC7 - Pt828004
MDVPKDQITALLDHELYNSAQILGSFLVSSATVSLETSPQLKAENQILLGDALFREREFRRAIHTYKQALHYYKIIPKQSSTTSRSSLS
NRSSSPNSFNISAINENEVKFKIASCHATLNETRAALVEMEGIPSKARTLQMSLLMAKLYRSSRHTRLAITCYKECLRHCPFVIEAIVA
LAELGVAAKDVISLFSQVSNRSGRAPLDHTDSTRWLQRYVEAQCCIASNDYKGGLELFGELLQRFPNNIHILLEIARAEAIIGKNDEAI
MNFEKVRSIDPYVVTYMDEYAMLLKTKGDFSKLNKLVHDLLSIDPTRPEIFVALSVLWEKKDEIGALSYAEKSIRIDERHIPGYIMKGT
LLLSLKRPEAAVIAFRGAQELRADLRSYQGLVHSYLAFSKIKEALHAAREAMKAMPQSAKALKLVGDVHASNSGGREKAKKFYESALRL
EPGYLGAALALAELHVIEGRNGDAVSLLERYLKDWADDSLHVKLAQVFAATNMLQEALSHYQAALRINPQNEAAKKGLERLEKQMKGVD
PDAPEEDEENEVEDADGDQEETDLL
>SbAPC7 - estExt_Genewise1.C_chr_90882
MEAARESMAALLDAGLFGPAQTLGCFLVSSAGAGNDAGMSMKVESLVQHGDALYGEREFRRALNAYKQAMQYSRSIPRQATSSTRSSVS
ATGRSPSPNSSNLLSFNENEVKFKIALCHSALCEHREALHEMEGIPSKVRTLKMNMMLGKLYRISRNSRTAAVCYKECLRQCPYVFEAI
TALAEMGLSAKEFSLLFPQAPNRGGKVPGDFVDAQRWWTRYVEAQCCIASHDYKGGLDIYLELMQRFPNNVHILLEIAKVEAIIGRNDE
AIMNFEKARLIDPNIMTYMDEYAILLKSKSDYVKLNKLVHDMLHIDPARPETCVALAAMWKRKDKRKALTYAEKSLRVDDRHITGYIMK
GNLHLSLNRPDLAVTDFRGAQELRADLRSYQGLVCAYVALSKCKEALFTAREAMKVMHQSAKALKLVGDVHAISSSGREKARKFYESAI
RLEPGFLGAALALADLHVVEGRNKEAVMLLEKYLRQWADDSLHIKLAQVHASTNMLSDALSHYQSALRINPQNEAAKNGLERLEKQMKG
VDPDAPEEEEENEEDDIDDRDEFI
>SmAPC7 - estExt_fgenesh2_pg.C_140388
MEILREQMGFLLDQGLHDSAEILGSFLLCAAASNNDLAPTVRAENMVLFADALYGRREYKRALNFYRQALQQCRVTLKPNTAVTRSTLS
STGSRPSSANSSYCGTINDNEVKYKIALCHMGLRDTRSALTEMEAIPSKARTLRINLTLAKLYRVTGYDRAAVASYRECLRQCPYVLEA
IIALAELGIPAKDIQLLFPQTPSKVARAPSDGHESSRLLQKLTEVHCGIASLDYKGALENLNQLAQRFPNNLHVLLETGKVEGALGRGD
EAVHSFEKSRQVDPCNLTDMDEYAMLLRMRGDTAEMNRLVYDLLNVDAGRPEVWVSSAVYWETRDDRVRALSYADKVTAILQSIRVADR
HTPAYLLKGNLSLSLNHSEAAVMAFRKAQSLKPDLRSYQGLVRSYLALSKNKEALCAAREAMKAMPHSAKALTLVGDVYAHVPEGREKA
RKFYESALRLEPGYLGAVLALADLHRMESRNEEATLLLQRYLQNWADDALHTKLAQILALTNKLGESLSHYQAALSINPQNEAARKGLE
RLEKQMKGVDPDALEEDEENEAEDGEGDPEEAEFLYIAGLRPCWSDNHSTQLRHFSKEHGILTAQVSSRRKKLRLRCKASSQSSQQQSS
LLSFLCPLLKLIGSGDPSAPRNGLLESATSGIASLARLPWGKNVNTQIVSSRTKQPLELFKLYEFEACPFCRRVREALTELDLSAEVYP
CPKGSRVHRAFVKASGGKEQFPFLLDPNTGVSMYESSDIVNYLFQEYGERRRPTFGILESTLVTGWVPTIIRAGRGMSLWNGALPDPPQ
KLLELYSYENNQFARLVREALCELELPYILWNTGKGSLNCSKLKQISGSTQVPYLVDPNTGIQMAESLDIIRYLFANYNSKNEAFGASP
GRFSATAGNELKVGRFEVLESKDELDLIGQQPPEPLRQFNQRFQSYIRKFVLCGDLIRLCITLPSSSLLGLVLIRTLSPGISSVPFAMR
RQLVEGHDPVLWPVVNNLSLGMAKALGLSGVGKFHGGWFSLTVGQVAWSFRLVNRLPMTGCFKDG
>PpAPC7 - e_gw1.10.41.1
MGSFIEVLREQMTFLLEQGLYDSAEMLGTFLMSLVSANGELSPISRAESMILYGDALYGRKEFRRALNVYRQALQLCRATSKQPMSSAR
TPVQSRPSSAASAVHDSKINENEVKFKIGLCHLAVNDTRAALSEMEGIPSKARSLRMNLTLAKLYRITGYDRVATASYRECLRQCPYVL
EAIVALAELGVPSKDIHALFPQGQSKISRSTTDCLDPVRWLQRFADGHSSIATHDYKGGLEHFNNLAQRFPNNTHLLLENAKAEMAIMK
NDEAAHSFEKSRQIDQFNISSMDEYAMLLRNRGDHMELNRLVHELINIDSTRPEVWVAAAVYWEMRDDKIRALTYADKSLRVDDRHTSA
YVVKGNISLTLNRPEAAVMAFRKAQLLKADLRSYQGLVRAYLAIPKHKEALCAAREAMKAMPQSAKALTLVGDVYAAHQDGRDKARRFY
ESALRLEPTYLGSVFALADLYGMEGRTEEAILLLQRYLKTWADDALHTKLAQIFAASDKLGDSLFHYQTALSINPANDAAKKGLERLEK
QMKGVDPDALEEDEENEGEDPDADAEEGEFL
>VvAPC7 - GSVIVT00019804001
MDVPRDQVTTLMEHGLYTSAQMLGCFLVSSSAVNPETSPHIKAESLVLLGDALFREREHRRAIHTYKQALQHYKIIPRQNSTTRISLST
SNRSSSPNSFNISAINENEVKFKIASCHCAINENVAALAEMEGIPSKARNLQMNLLMGKLHRNSRQNRAAIACYKECLRHCPYVIEAII
ALAELGVTAKDILSLFPQTPNRSGRPPFDHFDSSRWLQRYVEAQCCIASNDYKGGLELFTELLQRFPNNIHILLEIAKVEAIIGKNDEA
IMNFEKARSIDPHIITYMDEYAMLLMIKSDHLKLNKLVHDLLSIDPTRPEVFVALSVVWERKEERGALSYAEKSIRIDERHIPGYIMKG
NLYLSMNRPDAAVVAFRGAQELKPDLRSYQGLVRSYLALSKIKEALYVAREAMKAMPQSAKALKLVGDVHASNSGGREKAKKFYESALR
LEPGYLGAALALAELHVMEGRTGDAISLLERYLKDWADDSLHVKLAQVFAATNMLQDALSHYQSALRINAQNEAAKKGLERLEKQMKGV
DPDAPEEDEENEVEDADGDQEEAELL
>OspAPC7 - 0800010039
MSSPSLRDACTAHAREALAHGAHESAARWASLALSRRDVDDEGEGAAVASGRTSDGWRRDAIDLEIKGDALAARGDARGAAAAYRRAAA
LAARVGGGDGGRASASGRDAMDGDEMSGYEMTMKLVRTLIKIGDEEAVREAKECLESTPPANRTLENRLLAAKIARREGHVRAAASAYK
EVLAKWPFAIEAVVALAELGVKSMDARRALRKTASEEDVAGEDFDVLEAYATAYAALESDDLVTAQSHMQSVKRRFPNDPYMSIIKARI
ATVGSRDVAAATREYAAVRSRDPCFVEGMDAYGMLLRESGDARALNVLSSDLAHLMPQSAESWTCMAMRFDAERGGREDAVAAAEKAVL
LNPQSNIAHLVLGSIYLRSKRYKSAVRAFNQCNAIKVSMEAYHGLVKAYLALASRPNAAMCAKQALKRSPQSALAWSLMGDVHAKNRDE
YDDAIKAYEQALAYDPRHVRSVKALAALNIKIGKVHVACAILQRQLDDYQPSDENELVQLYCRLAQALMLSRQTADGVKYYTRALAIQP
TCDIAKRALEKFEQTRNPEHAATMSDEDQEGAEDMMEDDASRDGDWMVEGRTTSA
>MspAPC7 - EuGene.0000050178
MASTQQVRAMERLLAAEMWESAEALGGFLCSASPRIPPDAVASAERARHLALFGDALLGKGEHRRALNAFRQALSVNRLAPKVPTGNNR
SSAMGTPETPATPGISPPVDEASLKFKIGRCHLALREYRAALAELETIPARARTLPVTMTLAKTYRRTGYERAAVACYKEVVRDCPYAV
DAIAALAELGCSAEEIRADAHHEEPPGDASYGWLHHLAEAHGAARSHRLEAAASHLRRLDEIFPDDPRVWCQLARVHRDRGDVQEAADC
YRRCVRSDPCVVDCMDAFAALLNGPSVELNALVNNLLENAPGRAESWSAAALYWESRGDAEKALSFAERASDIDDQHVTAHVTKGYLRL
KCKRADAAVHAFKRALQLAPATRTYAGLVASYLILGRIKEATATAKECARAAPNASASHALLGDVEAAAQGHRDRARRFYEHSLKLDPS
```

CAGVAAALAETHAASGRSEAAAELLRRHLDTHAAHDAGAQVALHCRLGAVLAQSKQLADALGHYQSALAIYPESDEARRGVSRVERLMK
GQDPDAPDEEVDEEEDDDEEVEDADADGDDGSDFMG

>OspCDC23 - 0700010235
MAYFDLGEYRRCAHTLRDARAPLPTFLREYATFLAGEKSKGQSGAVGGASVDGGSGRIDASQPTAIGTRPPGGVVEDAGESNAELESLN
QWLRAQDDTGAKDGFLTFLHGIVCRESGQPTQAKTLLAEACRKYPLNWSAWQALIPMLASEEEEKALDLPRDHWVYTWFIGVFQLEIQK
NKESLVTFDSLSNDFPQSKLLLGHVAEAHYNLREFEEAHSIYKDMQEVDPYRVEGMDNYSNVLYVQELFAELSHLAHHLVSTDKYTPET
CCVVGNYYSLKSMHNKAVVYFKRALKLNPRYLSAWTLMGHEYVEMKNPAAAIDAYRHAVDINPRDYRAWYGLGQTYEILQMPYYALYYY
QQAVKLRPDDSRMWCAMGQCYESDQLRMFTSAIRCYQRAVANNEGEGIALAKLAMLHREKNPKAAAHYYILNLKRLDKEDLESAEKHEA
LEFLAHYYMKEQRLQEAEAACARLLDVSGPARHAAKALLREIHSMQSLQ
>MspCDC23 - e_gw1.4.177.1
MAGSRVKHSELRQAVRDLQNRGLLHSARWAAEQLYGLEDEVPGAREDEDDATPAAAPATPATPFANDDASDDEMDLGTDEKPAKPPATA
PPADDAPEWRGRGGTPEAAGDDFILAKAYFDLGEYRRASHQVTENRSSLGKFLRYYSLYLAGEKRKNEEMLELETLSFDLELILKDDAP
ANAECRNDPFLHYLLGLVLVERESRDKAKVSLCAACRGYPCNWGAWEALMPLCATVEEAQALPLPDHWMRKWFIAALQLELQDNRKGLQ
AYASLVMDIPASAIGVVQMAVGHYNMREFDRAQSIFEDVYKADPYRLEGMDTYSNILYVKEATAKLSYLAHCAVLTDKYRPETCCIVGN
YYSLKAQHEKAVVYFSRALRLNWKYLSAWTLMGHEYVEMKNPAAAIDAYRHAVDINPRDYRAWYGLGQTYEILTMPYYALYYYQRATRL
RPKDPRMWCAMGQCYESDQLQMTVAAIRCYQRAHQNGDQEGIALGKLAKLHHEANNAKAAAHYHRLNLVRLLEEGADQHEDTVKALSYL
ADYYKNTKDYGKAEACCMRLLDYAGPEKQLAKALLREIHALQEAAAERAEDTSMADV
>CspCDC23 - IGS.gm_1_00472
MQAQELRQAVHDCRSRALYASAKWAATALCGLPEEEVMVSSQAAAAAPPSGSAAFELARSFFDLKEYRSAAHALRDSRDQLSLFLRGYA
TYLAGEKRKEEERIESKAAGEAAAAANTELDGLEGELQALVAAGQADGFLLYLLGLVLADREKKEEARQALAASVTAYPCNWSAWLALQ
SVCADLAAVGQLPLPDHFMRRFFLASLCVDMHHNAEALQHLQGLSDEFPRGEAVILLAALAHYNLQNFDEAQELFEGLLLRDPHRIEGM
DIYSNILYVKEEFAALSALAHRCAGADKYRPETCCVIGNYYSLRGMHERAVQYFRRALRLNPAYLAAWTLMGHEFVELKNPPAAIEAYR
HAVDVNPRDYRAWYGLGQTYELVNMPYYALYYFRRAVQLRPHDARMWNAMGHCYQQEQLGLLDAAIRCHRRALPYDKEGDGGDLEAAER
YYTRLLDFGAGSKEAAKSSLREIRTLKAAGVAPRGPAGLTPVRPDSPPDSPGSDMMAGMSPY
>VcCDC23 - fgenesh4_pg.C_scaffold_113000014
MVPERPGATVPSPATPADIAAELTLAVHDLNARGLFQAAQWAAEQLVGLELHSPHQGASGWQHHQQHHPQGHAGFTSRTSSPQTANLLS
RNDPDEQHPQYLLARAYFQSKEYRRTAHALSGLTGPLPTFLRLYATYLAGEKRRDPTAVAASATPLLFPAASAPVVQTNAPPSPTAVAA
AATLMLFPAAAAPVVQTNAPPSPTAVAAAATPLLFPAASAPVVQTNAPPSPTAIAAAATPLLIPAAAAPVVQTNAPPSPTTVAAAATLL
LFPAAAGTDTFSNILFVKEAAAPLSVLAHRVAATDKYRPETCCVLGNYYSLQAASQAGPYREVGCLPAKVWCVGRDGRDAYRRAIDVSP
QDFRAWYGLGQAYELLKMPYYALYYYRRAAQLRPTDARMWCALAQCFVHEQIGLQDAAVRAYQRAIAHDDPDGIAVHKLAKLYESRGEP
HAAERLFRDSLRRLERHSHAFLSLSFAAHNFPTSLPRMCLCPLLLLPGQFDRSTAVAALHRTGGDPVAAVRAYWYPVTGGGGGEGPEHG
A
>CmCDC23 - CML190C
MTSEVDVALAVLRQVHTEQKYRELKEALRESLDRGLVLSARWLGELVASLGRKLEPQLLDNDAETVGTKRGESDLFFYGKALFDAREYL
RAASILEACTSKLGRFLRWYSLFLHGEKRKEEMLLEVSPAGCAAPCKPQNTLAASIVQEMDKAVQSDASLPEDAYICWLRGTALKACGC
RKEALCAFIEALQRKPHLWAAWTALHELGEDYSSIISALRTLLETGKAWMFAFFLVMQSGTFSGETSIIPVLQALSSEFPDSVTLLHLL
AHAHFSAHDFETAAELCRRLRELDPYFLDAVDLYSNILFVQEDQATLSTLARDCVQIDKYRAETCCVVGNYFALRQNHEKAVQYFRRAL
TLNRSYTTAWILMGHEFLEMRNTSAAVEAYRRAIDLDPADFRPYYGLGQTYELLHMPHYALYYFEKAATLRPCDDRMWAAVSQALQDIG
RLDDAVRCLEKALTWNPDNWSYAKRAGDLFWETGQYDSAAKHYATYLEIRRALRAPHATIDTDEADVVVRMMTYLRQRGELKLANEYLE
YMTAVESAEKRETMRKLACDASDPAL
>AtCDC23 - At3g48150
MVSKECCRNEIRAAIRQLSDRCLYSAAKWAGEQLVGIEQDPSNFTPANTRFQRGSSSIRRRFSTNESISTPLPSVGFSQAATPLPEEDE
AIDGDIYLLAKSYFDCREYRRASHMLRDQVSKKSLFLRYYALYLAGEKRKEEEMIELEGPLGKSDAINRELVSLERDLSALRRTGAIDS
FGLYLYGVVLKEKGNESLARASLVESVNSYPWNWSAWSELQSLCTSIEILNSLNLNNHWMKEFFLGNAYQELRMHTESLAKYEYLQGIF
SFSNYIQAQTAKAQYSLREFDQVEIMFEELLRNDPYRVEDMDLYSNVLYAKEACAALSYLAHKVFLTDKYRPESCCIIGNYYSLKGQHE
KAVMYFRRALKLNKKYLSAWTLMGHEYVEMKNTPAAIDAYRRAVDINPTDYRAWYGLGQAYEMMGMPFYALHYFRKSIFFLPNDSRLWI
AMAKCYQTEQLYMLEEAIKCYKRAVNCTDTEGIALNQLAKLHQKLGRNEEAAYYFEKDLERMDAEGLEGPNMFEALVFLATHFKNHKKF
EEAEVYCTRLLDYSGPEKEKAKSLLRGIRMAQTGFPSMDLEHFPI
>OsCDC23_1 - LOC_Os02g43920
MASSNETYRVELRAAARQLGERGLYSAAKWAAELLVGIEPDATPAPSSVMDTPSSSGSAASGGRLLHLHRSGGSSFRRRLRPGAAEAGT
PLGGVSYVSTPIPDDDAFDVGGDRYLLAKTYFDCREYRRAAHVLRGQTGRKAVFLRCYALYTAGEKRKEEETVELEGSLGKSNAVNQEL
VALERELATHRRTGAIDSFCLYLYGIVLRDKGSEALARTVLVESVNSYPWNWSAWLELQSLCTSSDILNNLNIKNHWMKDFFLASAHLE
LKMHEEALKRYERLMGVFRCSDYIQAQIATVQYSMRDLDEADMIFEELLRTDPFRVDSMDVYSNLLYAKESSTALSFLAHRVFLTDKYR
PESCCIIANYYSLKGQHEKSVLYFQRALKLNRKYLSAWTLMGHEFVELKNTPAAIDAYRRAVDINPRDYRAWYGLGQIYEMMGMPFYAV
YYFRKSSYLQPNDARLWNAMAQCYESDQLQMIEEAIKCYERSANNNDTEGIALHQLAKLHGMLGQSEEAAFYYKKDLERMEVEERQGQN
FVEALLFLAKHCKSIGRFEEAEHYCTRLLDYTGPERETAKSILQGLKRAQSVLPLMDIDHFAM
>OsCDC23_2 - LOC_Os06g46540
MASSKEAYRVELRAAARQLGERGLYSAAKWAAELLVGIDPDATPAPSSAMDTPSSSGSGGHLLHLHRSGGSSFRRRLRPGAAGEAGTPL
GGVSYVSTPIPDDDDAFDVGADRLETLYSNIEMNGQYKVLTYSSNTIALMQQGGLRIQAGEKRKEEETVELEGSLGKSNAVNQELVALE
RELATHRRTGAIDSFCLYLYGIVLRDKGSEALARTVLVESVNSYPWNWSAWLELQSLCTSSDILNNLNLKNHWMKDFFLASAHLELKMH
EEALKRYERLMGVFRCSDYIQAQIATVQYSMRDLDEADMIFEELLRTDPFRVDSMDVYSNLLYAKESSTALSFLAHRVFLTDKYRPESC
CIIANYYSLKGQHEKSVLYFQRALKLNRKYLSAWTLMGHEFVELKNTPAAIDAYRRAVDINPRDYRAWYGLGQIYEMMGMPFYAVYYFR
KSSYLQPNDARLWNAMAQCYESDQLQMIEEAIKCYERSANNNDTEGIALHQLAKLHGMLGQSEEAAFYYKKDLERMEVEERQGQNFVEA
LLFLAKHCKSIGRFEEAEHYCTRLLDYTGPERETAKSILQGLKRSQSVLPLMDIDHFAM
>PtCDC23 - Pt834319
MNSKETCRSELRIALRQLSDRCLYSASKWAGEQLVGIEQDPAKFTPTNTRFQRGSSSIRRRFRTNDITSTPVTGMSYVSTPVLEEDEVI
DGDFYLLAKSYFDCREYKRAAHVLRDQNAKKSVFLRCYALYLAGEKRKEEEMIELEGPLGKSDAVNRELVSLERELSTLRKNGTIDPFG
LYLYGLVLKNRGNQNLARTVLVESVNSYPWNWNAWTELQSLCTTIEMLNSLNLSNHWMKDFFLASAYQELRMHNESLAKYEYLQGTFSF

SNYIQAQIAKAQYCLREFDQVEVIFEELLRNDPYRVEDMDMYSNVLYAKECFSALSYLAHRVFMTDKYRPESCCIIGNYYSLKGQHEKS
VMYFRRALKLDKKYLSAWTLMGHEYVEMKNTPAAVDAYRRAVDINPCDYRAWYGLGQAYEMMGMPFYALHYFKKSVFLQPSDSRLWIAM
AQCYETDQLHLLEDAIKCYRRAANCNDKEAIALHQLAKLHFELGRPEEAAFYYKKDLDRMEDEEREGPNMVEALLFLAQHCRTHKRLEE
AEVYCTRLLDYTGPEKEMAKNMLRGMRSESSFPSMDVEHFPP
>SbCDC23 - estExt_Genewise1.C_chr_48538
MASAKETYRAELRAAARQLGERCLYSAAKWAAELLVGVEPDAAPVPSAVMDTPSSSSATSAGRLLHLHRSGGSSFRHRPRPAGGGTSSE
AGTPLGGVSYVSTPIPDDDAFDSGGDKYLLAKTYFDCREYRRAAHVLQKQVGRKAVFLRCYALYMAGEKRKEAEMIELEGSLGKSNAVN
QELIALEKELSTHKRTGSIDSFGLYLYGIVLRDKGCEGLARTILVESVNSYPWNWSAWSELQSLCTSSDILNKLNLKNHWMKDFFLASA
YLELKMHEEALKRYERLMGVFRCSGYIQAQIATVQYSMRDLDEAEMIFEDLLRTDPFRVDSMDIYSNLLYAKESLTALSFLAHRVFLTD
KYRPESCCIIANYYSLKGQHEKSVLYFQRALKLNRKYLSAWTLMGHEYVELKNTPAAIDAYRRAVDINPRDFRAWYGLGQIYEMMGMPF
YALHYFRKSSYLQPNDARLWIAMAQCYESDPLQMIEEAIKCYERAADSNDTEGIALHQLAKLHGMLGQSEEAAFYYKKDLERMEVEERQ
GQNVVEALLFLAKYNKSIGKFEEAEDYCTRLLDYTGPEKETAKNLLQGIKRLQSGFPSMNTDHFAL
>SmCDC23 - estExt_fgenesh1_pm.C_620007
MREYRRAAHALRGATGKKSFFLRCYATYLAGEKRKEEEIIELGGPLGRSDAVNPELAGLEQELTSHSEKGTLDAFGNYLYGVVLHERDR
KSEARAVLCASVNTYPWNWSAWLELQALCTDPEILPTLRLEDHWMRDFFIASVYLDLQKNSEGLACYRSLHAMFPVSDYVLAQTATAHY
NLREFDEAEGLFEELLRTDPYRIEGMDMYSNILYVKECFAALSHLAHKAVLTDKYRPETCCIIGNYYSLKAQHEKAVLYFKRALKLNRK
YLSAWTLMGHEYVEMKNTPAAIDAYRRAVDINPRDYRAWYGLGQTYELLIMPFYALYYYRRAAQLRPHDARMWCAMGQCYENEQLQMFD
AAIRCYRRAVNNNDREGIALNKLAKLHSQLGQADQASYYYKKNLERLEADQSEGQDVVDALLFLATHSKNQGFLDDSEMYCMRLLDYGG
PAKEEAKALLREIRSVQQHASVLPSMDLEQFTP
>PpCDC23 - e_gw1.80.136.1
DDIDSEQDDDGLLLARSYFDTREYRRAAHALQSASGSTATFLRFYATYLAGEKRKEEDTVDLAGPLGRSDAVNLELVSLEQELSSLYRM
GTLDAFGMYLYGVILRERDKHAEACTVLCASVNSYPWNWSAWLELQALCTDPDIFHTLDLKDHWMRDFFVASLFLDLQRNSEGLARYQS
LNLLFPGSDHILAQTAVAHYNLREFDDAERLFEELLRADPYRIEGMDTYSNILYVKECFAALSHLAHKSVLTEKYRSETCCIIGNYYSL
KAQHEKAVLYFKRALRLNPKFLSAWTLMGHEFVEMKNTPAAIDAYRRAVDINPRDYRAWYGLGQTYEILAMPYYALYYYRRATQLRPHD
ARMWCAMGQCYESDQLQIYDAAIRCYKRAVNNNDREGIALNKLAKLHVNLGRHEQAAFYFRKNLERMEADQNESQEYVDALLFLGNYSK
NNGSLEEAEVYCTRVLDFGGPVSSYLLSCPRLSK
>VvCDC23_1 - GSVIVT00003617001
MSSKDSCRNELRFAIRQLSDRCLYSAAKWAAEQLVGIEQDPAKFTPSHTRFQRGSSSIRRRFRTNEIASTPTAGVSYVSTPVLEEDEAV
DGDFYLLAKSYFDCREYRRTAHVLRDQTGKKAVFLRCYALYLAGEKRKEEEMIELEGPLGKSDAVNHELVSLERELSTLRKNGTVDPFG
LYLYGLVLKEKGSENLARTVLVESVNSYPWNWNAWTELQSLCTTIDILNSLNLNNHWMKDFFLASVYQELRMHNESLGKYEYLQGTFSF
SNYIQAQIAKAQYSLREFEQVEIIFDELLRNDPYRVEDMDMYSNVLYAKECFSALSYLAHRVFLTDKYRPESCCIIGNYYSLKGQHEKS
VVYFRRALKLNKNYLSAWTLMGHEYVEMKNTPAAVDAYRRAVDINPCDYRAWYGLGQAYEMMFMPYYALHYFRKSVFLQPNDSRLWIAM
AQCYETDQLQMLEDAIKCYKRAANCNDTEAIALHQLAKLSKELKRSEEAAFYYKKDLERMEAEEREGPNMVEALLFLATYYKSQKRFEE
AEIYCTRLLDYTGPEKETAKSLLRGMRKAQSGFPSMDIEHLPP
>VvCDC23_2 - GSVIVT00019315001
MSSKESCRNELRTAICQLSDRCLYSAAKWAAEQLVGIEQDPAKFTPSHTRFQLGSSSIRRRFRTNEIASTPTAGVSSVSTPMLEEDEAI
DGDFYLLAKSYFDCREYRRAAHVLRDQTRKKAVFLRCYALYLAGEKRKEEEMIELEGPLGKSDAVNHELVYLERELSMLRKNGTVDPFG
LYLYGLVLNKKGSENLARTVLVESVNSYPWNWNAWTELQSLCTTIDILNSLNLNYHWMKDFFLANAYQELRMHNESLGKYEYLQGTFSF
SNYIQAQIAKAQYSLREFEQVEVIFEELLRNDPYRVEDMDMYSNVLYAKECFSTLSYLAHRVFLTDKYRPESCFIIGNYYSLKGQHEKS
VVYFRRALKLNKNYLSAWTLMGHEYVEMKNTPAAVDAYRRAVDINPCDYRAWYGLGQAYEMMGMPYYALHYFRKSVFLQPNDSRLWIAM
GQCYETDQLQMLEDAIKCYKRAANCNDTEAIALHQIAKLSKDLKRSEEAAFYYKKDLERMEAEEREGPNLVEALLFLATYYKSQKRFEE
AEIYCTRLLDYTGPEKETAKSLLRGMRKAQSGFSSIDIEHLPP
>OspCDC16 - e_gw1.01.00.209.1
MSRERALDAARAARDAYATNGSIVSAIFCADKARALSGDGDEDALALAELLRRDGQHRRASAVASVGEGIRASARWRLLRGRCALALRA
PEECLRALGEDESGTEEGVGGAARETEEARATRLADGNRADAAMCALRGRAYDAMENRARATRAYALALRFDPMCYEAYDAVLSGHAL
SESEEGALVDSLVVNEKNAWIKEVYAVLSNVRDVESELLRDSALGEEDASSQPTTSKSASDVLKTSGDVRIARARRLYDRGEFAACYTE
LRLQYDVEPSRLDGMPLYFATLVELGKKNDLYLLAHSLVDEYPKKALTWFGIGCYYMATKQFDQARKYFSKATTMDASFVQAWIGFGHA
FAAQDESDQAMAAYRTAARLFSGTHVPVMSIGIEYQRTNNLSLAYQFFRKSFEISQTDPLLYNEYGVLLYREGQYESAAQHFERALELA
PTQMTSRWESLIVNMAQALRKVGRYDDAIAYFEYALSITPRAASTYAALAFTYQVKSRCTEPRALGLAIEYYHKALSLRADDVFSQHHL
ELALIDQSAITMPRHEQVDWNVDFPTSDDMAVTPELGGAQRDRDATPDQHGVFPTPSPQSFRGTPTMGRTPFSTRGDASEMDQSVDMDQ
SVDMDESD
>MspCDC16 - EuGene.0000020339
MTRCHHDLDATIAQLRDVVEDRLDKHLASSAIFFADKLVTMSGGALGDVFLHAKALYLGTHYRRAFATLHRGGLIPRKLNPGVENGSLR
SRTVDLRAPAENSCRLLAAQCLAAVKDWDGCLAVLGERDDGDSKSLIEETDTRGEQNPSLPGCLTSARSGVVRQRTKASRVEQLDRTQG
HGSRKKREYELRDGSVSIRASLCFMRGKAHGALENWKSAELWCKEALTLDPYCFEAFDLLISSHLLSVGEEDRFLSSLQIRPEDKWVPSL
YGTICHSGFFSSAEGSSCVASSHGHMNQATDVRCNLPVESDRDVCNACDVSNIPDTLHSQTSGTQPELFALKYNSEVILARSERYFNRG
DYQRCYDTIQELLANEPTKLAAMPCYLAVTVELRLKTKLYLCAHKLVEEYPAKAISWFAVACYYYCTRQFDSARRYFGKATILEATFVP
AWLGFGHAFAAQDESDQAMAAYRTATRLYPGCHLSLMCIGMEYHRTNNFSLAGQFLSRARHLRPADPLVYNELGALAFHNGDHVSAISH
LEKAIALIPQPVTATWEAILVNLAHSNRKLNNFDEAIFWYEQALSLAPRNASTYTALGFTHQLKGNFQSRMEKAIECYHKALSLKPNDD
FAQEMLTLALIDQCAVTMPPYNFVAYDAHQPLPVKSERRS
>CspCDC16 - gw1.20.35.1
QLRSVVHDCLAKHMYEAAAFFADKLVTLSGYSPAEVYTLAQAFFCSRQFRRCLQLLRSTELIEKDLRFRYLAARCLAECKEWEECLSVL
GGLDAEEPEQLQLPMPRSTVPLGSGINYFSVVCLLRGRVHDALENFPRAVKWYQAALKADPFNYEAFQALVGSHKLSNAEELELVNSLD
IPPQQGWLKLLYTSRCKNKAGVGLAPMALSPVHEEEKSTPQAAAAAAAGWGLHDNLDVIACRAEWLYHRGAYAECYTLTASALERDPYA
TECLPVHLASALELRKKNELFIQGHKLVEEHSDRAVSWFAVGCYYMCSQQYEAARRYFGKATALDRAFAPAWVAFGHAFAAQDESDQAM
AAYRTAHRLFPGLHAPLMGMGQEYQRMNNLGLAEQCFSQAARLCPSDPLVANELGVLAYRNRQYEVAAGWLRRALSLVPGGRPTPSWEA
TLVNLGHTLRKLRQWDAAIECYLQALGLKPGQPGTYSALGYAHHLKGDYNAAIENYHKALGLRPEDVFTAEMLAEAM

>VcCDC16 - e_gw1.79.30.1
MEALCSRLRGLAQDCIDKHLTASAIFYADKLVTFSNNSPGDVYLLAQAYFAARQFHRALSLLRNAGVVELGAEFTYLAGCCLAEAGDWE
EVVALLGDDEAMQEPQAYEDSVLEPPGGGAGVRVQAGMCCLRGRAFEALENRCRAAVWYQVALQLDPYCYDAFAALLEGHLLSNEAEVV
LVDRLRGALAEGDRWLGLLMRAKCKKVRQQQQQGKGEGVQSNGGNGSSGSFGCGLGGNVDVIACRAELLFHRGDYEAAYTLTRPVLCC
GRDPYALQLLPVHLAAATQLAAYGGGARADLFLLGHRLTEEHPELAVSWYAVGCYYLAARQPEAARRYLGKATQLQKGFAPAWLAYGHA
FSAQDERDQGFFSRTPRTQLQAVSYRPSQPAVQILSSSSCACVTAVRQAYNICPDDPAVCHELGVLMYKCGQTAAAAMWLDRALQLLPG
GRPTVHWEATLVALGHCMRKLCRFPAAAECYSAALALAPASPGTLAALGYVAQLAGDPRVAVEHYHAALALRPDDPFTTDMLRLALQVY
GG
>CmCDC16 - CMF102C
MSDILRAREDLRLGRFESAAYNAERLLAGEPENPAAVEVLAQALLSAGEARRALHRLERYRTRLPLEETVFIRLIEARCLAELRLYEDQ
VAIFEERGLPSTTGERDAARGNANPLIAAYYVLVGDAYEALENPQKASECYIAALQADESCVEALERLFYSNAVVASVPLGALPPSTSS
GAQHKALSVDQIALQALWPFETPFPKDSSPSKVYLMLANDLRFDDWRKRWSLVPCFFLTMVATRFWRLGFCQEAYRLVSFLVAERKELE
LKRSFPLFVALLAHHQDLITLFRYAHQLVQEFPRAAESWYAVGMYYFASGKYDASRAYFQKATLLNSNLAYVWVAYGHAFAAVDDSEQA
LAAYRTAMRLRPNDPTPLLHVGMEFARQNHLAIARNFFERAAAAAAVVVAPVLTSSADSDADEDALANGIERSRPWNELGVLCYRDGEY
AEAVAYFQKAARPLQAYKKAWSMRLGLDQRYRISDGGGGFAPEKQAEAAVQRPVLQQSGIENSQSTSSDTNAPSATSVWCDPTSTFYGC
QEARSLLATICSNLGHALIRLQAIDLAAEALEEALLVGHAVRLAPGCAGSDALARADTLSALGYVEHVRGSVQRAAQLYHEAARELALA
GINGSTLLLDALLERAVDELAMQSISEIATTE
>SbCDC16 - estExt_Genewise1Plus.C_chr_110496
MPLSAASILHAFLLTILLGGGATASFPPGDGDGDVNPSTMFTNDTGTSRIADDTERYICYLCTGRNPLLIRYCPIYWDECHLVCYADDA
PAATAAAIPAAPLPAASANPSGVHDDDECYVMKLYRNGSYTIVTRLGCARIATCLLSCGGGDMADSDRKALQGTATKTAAPTATTAAVQ
GSLTPLLADFQRCGTQVTATPSLPADAAAEPSPKRRRIGEAADPPPEMREEALERLRGVVRDCAGKHLYKSAIFLADKVATVTGDPGDI
YMLAQALFLGRQFRRALHLLNNSRLLRDLRFRFLAAKCLEELKEWHQCLLMLGDAKVDEHGNVLDQDDGSDFYFDKDAEDREINIKSAL
CFLRGKAYEALDNRDLARQWYKAAVKADPLCYEALECLVDNYMLTCEEESELLSSLQFGEEDGWLSTFYSCLITKHEKEDLVEAKFKKL
EQEACSISSSSSGERMKNNIDVLACKAEYHHQSGEYQKCFQLTSSLLERDPFHLKSTLVHLATALELGHSNDLYLLACNLVKDYPQKAL
SWFAVGCYYYCIKKYDQARRYFGKATGLDGTFPPAWIGTGIAYAAQEEGDQAMAAFRTAARLFPGCHLPTLYMGMQYVRMHNFKLAEQF
FTQAKSICPSDPLIYNEAGVVAYNMKEYRKAVQLFELTLKHTSSSLNEMWEPTLVNLGHALRKLKEYQKAVSYYEKALTLPTKSLSVFA
GLAYTYHLMDDFEAAINYYHKALWLKPDDQFCTEMLTDALGSICQARRRIV
>SmCDC16 - estExt_fgenesh1_kg.C_1220001
MENKLRAMVRDCLDKHLHASAIFLADKLVTVGGTEEDVHLHAQALFQGRQFRRALHLLRTHGLLHLHPRYRYLAAKCLEEIKEWDECLS
VLGDYEVDQHGNYPMKDDIDPESGHQAGINIAAALCLLRGRACEALENRTRALCWYKASLRVDPYCYEAYEHIVDNHMLSSEKEVAFLS
SLKFDADDRWLSLLYSCQAKKYGQISALESKLSELEREPQKHSNVGLSLKDNNDVLACRADYLYHRGEFQLCYDITKTLLEKDPYHLKC
MPLHLGAALELGRKNELFLRAHNLVQEYSQRPIAWFAVGCYYYCIRQFDHARRYFCKATTLDGAFYPAWLGFGNAYAAQDESDQAMAAY
RTAARLFSGCHMPALCIGMEYLRTNNLNLAEQFFMQAKGICPTDPLVYNELGVMAYRNREYEEAARWLRKALVLVQETRNSLTLCWEPT
VVNLAHTLRKLKLYPEAISMYEKALALCPRGATTYAALGFTHHLQGSTGIAIDFYHKALGLKPDDTFTAEMLTAALTEECLRPSFAKEL
TALH
>PpCDC16 - e_gw1.402.10.1
MREGEVEKLRAVVRDCLGKHLYSSAIFFADKLATLAGNATQDLYMQAIAQALYLGKQYRRALHLLRRHHLITADLRFRYLAAKCLEEIK
EWDECLLMLGDSEVDEEGNLLVMDDQDTELLDKNIEEREISIGAAVCLLRGRAFEALENRARALRWYKAALKADPYCYEAFEHLIDNHM
LTSEEESVLLSSLKFDPEDRWLSLLYSCRAKKYGQVSVIEEKFQELEKEPDESRPEEERVGCSLKDNNDIIACRADYLYHQGEFQRCYD
TTKALLEKDPYQLNCMPFHIASALELGRKNDLFLRAHNLVQEYPQKAISWFAVGCYYYCIRQFDHARRYFCKATTLESSFAPAWLGFAN
SYAAQDESDQAMAAYRTSARLFAGCHLPALCIGMEYLRTNNLNLAEQFFLQARSICSTDPLVYNELGVLAYRNRDYDTASRWLRKALQL
VPPPLTEAWESTVVNLAHSLRKLKSYPEAISMYERALSLFPRGASTYAALGFTYHLQGKTGKAVDYYHKALGLNPHDTFTAEMLTSALQ
EECLRLSSAPDAEFYSLSGMPHIS
>VvCDC16 - GSVIVT00027833001
MREEEIEKLRGVVRDCLSKHLYSSAIFFADKVAAFTSDPADIYMQAQALFLGRHYRRAFHLLNASQIVLRDLRFRYLAAKCLEEQKEWD
QCLLMLGDAKVDEHGNVNDTKDCNVMYLDKEGEDREINISSAICFLRGKAYEALENRAQARLWYKAAIKADPLCYEALECLIENHMLTS
EEEASLLSSLQFAPEDGWLSSFYSCLIKKYDKETVVEAKFTELEKQSCNINPSDPSFICTLKNNTDLLACKAEYYHQCGEYQKCFELTA
ILLEKDPFHMKCTLVHLAAAMELGHSNELYLMACNLVKDYPQKALSWFAVGCYYYCIKKYDQSRRYFSKAANLDGTFPPALIGCGNAYA
AQEEGDQAMLAYRTAARLFPGCHLPTLYIGMEYMRTHSFKLAEQFFMQAKTICPSDPLVYNELGVVAYDMKEYNKAVWWFQKTLSHIPS
SLSEMWEPTIVNLAHAYRKLKMYHEAISFYEKALTLSTRSLSTYAGLAYTYHLQDNFPAAITYYHKALWLKPDDQFCTEMLTLALVDEA
RRGLDPRNESR
>AtCDC16 - At1g78770
MREEEIEKIRGVVRDCVSKHLYSSAIFFADKVAALTNDPSDIYMQAQALFLGRHYRRAFHLLNASKIVLRDLRFRYLAAKCLEELKEWD
QCLLMLGDAKVDDDGIVYDAKDGNVIDFDKDGEDREINISSAICFLRGKAYGALQNRSQARQWYKAAIKADPLCYEALECLIESHMLTS
EEESSLLSSLQFSPEDGWLSSFYSCLIKKYDKESTVELKFKKLENETSGSVSGSSMITLANNTDLLACKAEYYHQCCEYQKCFELTAAL
LEKDPFHLKCTLVHLAAAMELGNSNELYLMACNLVKDYPSKALSWFAVGCYYYCIKKYAEARRYFSKATGIDGSFSPARIGYGNSFAAQ
EEGDQAMSAYRTAARLFPGCHLPTLYIGMEYMRTHSYKLADQFFMQAKAICPSDPLVYNELGVVAYHMKEYGKAVRWFEKTLAHIPSAL
TESWEPTVVNLAHAYRKLRKDREAISYYERALTLSTKSLSTYSGLAYTYHLQGNFSAAISYYHKALWLKPDDQFCTEMLNVALMDECQN
GVDSKVELC
>OsCDC16 - LOC_Os03g13370
MPLSAASINRASYQVLLLLAAAAVSTTGGDGNTAPGNATATATTGGDDTEMYICYLCTGRNPILIRRCPIYWDYCHLNCFDDAPSTAAA
ADDVAAVPVASPAAPARRVGGVPRETLEDEECYVMKLYENGSYVIVTTLGCSQTASCLLSCGGGDLAADGEEALAAAHPAGAVGVSPPW
RMWDTKFGFPPAAPPTTAAAAQKNPKRRREAEAEGEVAAEMREEAVERLRGVVRDSVGKHLYASAIFLADKVAAATGDPADVYMLAQAL
FLGRHFRRALHILNSSKLLRDLRFRFLAAKCLEELKEWHQCLIILGDAKIDEHGNVVDQDDGSDIYFDKDAEDHEINIKAAICFLRGKA
YEALDNCDLARQWYKAAVKADPLCYEALECLVDNYMLTCEEESELLSSLKFGKEDGWLSAFYSCLIRKHEKEYIVEAKFKEFERESCSI
SSLSSGLTLKNNIDVLACKAEYYHQSGEYQKCFELTSALLERDPFHLKCTLVHLAAAMELGHSNDLYILACNLVKDYPQKALSWFAVGC
YYYCIKKYDQARRYFGKATGLDGTFPPAWIGTGIAYAAQEEGDQAMAAFRTAARLFPGCHLPTLYMGMQYLRMHNFKLAEQFFTQAKSI
CPSDPLIYNEMGVVAYNMKEYQKAVQWFELTLEHTSSSLNEMWEPTLVNLGHALRKLKKYQKAISYYEKALTFQTKSLSAFAGLAYTYH
LMDKFEAAITYYHKALWLKPDDQFSTDMLTLALESSCQITARTR

>PtCDC16 - Pt585761
MREEQIEKLRGVVRDCVSKHLYSSAIFFADKVAAFTDDPADIYMQAQALFLGRHYRRAYHLLNASKIVLRDLRFRYLAAKCLEELKEWD
QCLLMLGDAKVDEHGNVYDTKDCNVMYLDKDSEDREINISSAICFLRGRAYEALENRALARHWYKAAIKADPLCYEALECLIENHMLTF
EEVXLSQYDKECVIEAKFREVEKESCNSNPSSPSFMHTLKNNTDLLACKAEYFNQCGEYQKCFELTSDLLEKDPFHLKCTLVHLAAAVE
LGNSNELYLMASNLVKDYPQKTLSWFAVGCYYYCIKKYDQSRRYFSKATSLDGTFAPAWIGFGNAYAAQEEGDQAMSAYRTAARLFPGC
HLPTLYIGMEYMRTHSYKLAEQFFMQAKTICPSDPLVYNELGVVAYNMKEYNKAVLWFEKTLKHIPSLSQLWEPTVINLAHAYRKLKIY
HEAISCYERALALSTRSLSTYAGLAYTYHLQDNFTAAITCYHKALWLKPDDQFCTEMLSLALVDEGRRGIDPKIE
>AtCDC27a - At3g16320
MMENLLANCVQKNLNHFMFTNAIFLCELLLAQFPSEVNLQLLARCYLSNSQAYSAYYILKGSKTPQSRYLFAFSCFKLDLLGEAEAALL
PCEDYAEEVPGGAAGHYLLGLIYRYSGRKNCSIQQFRMALSFDPLCWEAYGELCSLGAAEEASTVFGNVASQRLQKTCVEQRISFSEGA
TIDQITDSDKALKDTGLSQTEHIPGENQQDLKIMQQPGDIPPNTDRQLSTNGWDLNTPSPVLLQVMDALPPLLLKNMRRPAVEGSLMSV
HGVRVRRRNFFSEELSAEAQEESGRRRSARIAARKKNPMSQSFGKDSHWLHLSPSESNYAPSLSSMIGKCRIQSSKEVIPDTVTLNDPA
TTSGQSVSDIGSSVDDEEKSNPSESSPDRFSLISGISEVLSLLKILGDGHRHLHMYKCQEALLAYQKLSQKQYNTHWVLMQVGKAYFEL
QDYFNADSSFTLAHQKYPYALEGMDTYSTVLYHLKEEMRLGYLAQELISVDRLSPESWCAVGNCYSLRKDHDTALKMFQRAIQLNERFT
YAHTLCGHEFAALEEFEDAERCYRKALGIDTRHYNAWYGLGMTYLRQEKFEFAQHQFQLALQINPRSSVIMCYYGIALHESKRNDEALM
MMEKAVLTDAKNPLPKYYKAHILTSLGDYHKAQKVLEELKECAPQESSVHASLGKIYNQLKQYDKAVLHFGIALDLSPSPSDAVKIKAY
MERLILPDELVTEENL
>AtCDC27b - At2g20000
MEAMLVDCVNNSLRHFVYKNAIFMCERLCAEFPSEVNLQLLATSYLQNNQAYSAYHLLKGTQMAQSRYLFALSCFQMDLLNEAESALCP
VNEPGAEIPNGAAGHYLLGLIYKYTDRRKNAAQQFKQSLTIDPLLWAAYEELCILGAAEEATAVFGETAALSIQKQYMQQLSTSLGLNT
YNEERNSTSTKNTSSEDYSPRQSKHTQSHGLKDISGNFHSHGVNGGVSNMSFYNTPSPVAAQLSGIAPPPLFRNFQPAVANPNSLITDS
SPKSTVNSTLQAPRRKFVDEGKLRKISGRLFSDSGPRRSSRLSADSGANINSSVATVSGNVNNASKYLGGSKLSSLALRSVTLRKGHSW
ANENMDEGVRGEPFDDSRPNTASTTGSMASNDQEDETMSIGGIAMSSQTITIGVSEILNLLRTLGEGCRLSYMYRCQEALDTYMKLPHK
HYNTGWVLSQVGKAYFELIDYLEAEKAFRLARLASPYCLEGMDIYSTVLYHLKEDMKLSYLAQELISTDRLAPQSWCAMGNCYSLQKDH
ETALKNFLRAVQLNPRFAYAHTLCGHEYTTLEDFENGMKSYQNALRVDTRHYNAWYGLGMIYLRQEKLEFSEHHFRMAFLINPSSSVIM
SYLGTSLHALKRSEEALEIMEQAIVADRKNPLPMYQKANILVCLERLDEALEVLEELKEYAPSESSVYALMGRIYKRRNMHDKAMLHFG
LALDMKPPATDVAAIKAAMEKLHVPDEIDESP
>OsCDc27 - LOC_Os06g41750
METLMVDRVHGSLRLFMHRNAVFLCERLCAQFPAETNVQLLATCYLHNNQPYAAYHILKGKKLPESRYLFAMSCFRMNLLREAEEEALCP
VNEPNIEVPSGATGHYLLGVIYRYTGRVEAAAEQFVQALTLDPLLWAAYEELCILGVAEDANECFSEATALRLQQELTSTSNVEKSNFV
NENRFLSSNVSASFGDSPKQIKQLHANTTAEVSGYPHVKSTALHMQNGAPSNLSQFDTPSPTSTQASGIAPPPLFRNMHAYQNTAGGNA
PSKPKVNAPNLTLRRKYIDEAGLKKVSGRLFNQSSDSVPRRSARLSRDTTINSNSNISQFGGNGTDHSSGNRYHVVDEMWTDNVTSTSS
STSIVDGRYPEQEKSERVLSQDSKLAIGIRELMALLRTLGEGYRLSCLFKCQEALEVYRKLPEAQFNTGWVLCQVGKTYFELVNYLEAD
HFFELAHRLSPCTLEGMDIYSTVLYHLNEEMRLSYLAQDLVSIDRLSPQAWCAVGNCFALRKDHETALKNFQRAVQLDSRVAYAHTLCG
HEYSALEDYENSIKLYRSALQVDERHYNAWYGLGVVYLRQEKFEFAEHHFRRAFQINPCSSVLMCYLGMALHALKRNEEALEMMENAIF
ADKKNPLPKYQKALILLGLQKYPDALDELERLKEIAPHESSMYALMGKIYKQLNILDKAVFCFGIALDLKPPAADVAIIKSAMEKVHLP
DELMDDDDDDDEI
>PtCDC27_1 - Pt835890
MEAILVDCVNHSLRHFMHRNAIFMCERLCAEFPSETNLQLLAGCYLQNNQAYSAYHILKGTQMAQSRYLFAISCFQMDLLNEAEAALCP
TNEPGLEVPNGAPGHYLLGLIYRYTDRRKSAIHHFKQALSIDPLFWAAYEELCILGAAEEAAAVFDEAAALCIQKQHMNHASASQNLSI
SNEDRNLVSARNFGLEDGSPRQSKHPQGNNLRDIPGNYHGATTLGGSASQPSNGGLPNLSFYNTPSPMATQLSSVAPPPLCRNMQPNGS
NLSMPGFDNSARSTLNSNMQAPRRKFVDEGKLRKISGRLFSDSGPRRSTRLAAEAGSNQNTSSTLVAGNGTNNSPKYLGGSKFSSMAIR
SVTVRKGQSWVNENYDEGIRNEAFDDSRANNTSSNCSLSLTGDSRSLETEVATMPVGGVIASPSCILSGALEILGLLRTLGEGYRLSCM
YRCQDALDVYMKLPHKHYNTGWVLCQVGKAYVELVDYLEADRAFSLARRASPYSLEGLDVYSTVLYHLKEDMKLSYLAQELISTDRLAP
QSWCAIGNCYSLQKDHETALKNFQRAVQLDSRFAYAHTLCGHEYVALEDFENGIKSYQSALRIDARHYNSWHGLGMVYLRQEKNEFSEH
HFRMAFQINPCSSVIMSYLGTALHALKRNEEALEMMERAILADKKNPLPMYQKANILVSLESFDEALEVLEELKEYAPRESSVYALMGK
IYKRRNMHEKAMFHFGLALDLKPSATDVATIKAAIEKLHVPDELEDSL
>PtCDC27_2 - Pt278795
MEAILIDCVNNSLRHFMYRNSIFMCERLCAEFPSETNLQLLAGCYLQNSQAYSAYHILKGRTQMAQSRYLFAISCFQMDLLNEAEAALC
PPNEPGAEVPNGAPGHFLLGLIYRYTDRRKSAIHHFKQALSIDPLFWAAYEQLCILGAAEEAAAVFDEAAALCIQKQYMNCASASHNLS
ISNEDHNLVSARNFGLEDGSPRQLKHLQGNNLRDIPGNYHGASTLGGPVSQPSNGFILQLSGVAPPPLCSNLQPNCSNPSTLASLIYFQ
ISGRLFFDSGPRRSTRLAAEAGANQNTSATLVAGYGTNNSSKYLGGSKLSSMAIRSVTVRIRNEAFDDSRANNTSSNCSSSPPGDSRPL
ETEVATMPVGGVIISASCILNGALEILGLLRTLGEGYRLFCMYRCQDALDVYMKLPHKHYNTGWVLCQVGKAYVELVDYLEADRAFSLA
RRASPYSLEGLDVYSTVLYHLKEEMKLSYLAQELISTDRLAPQSWXCAMGNCYSLQKDHETALKNFQRAVQLDSRFAYAHTLCGHEYVA
LDDFENGIKSYQSALRIDARHYKSWHGLGMVYLRQEKNEFSEHHFQMAFQINPHSSVIMSYLGTALHALKRNEEALEMMERAILADKKN
PLPMYQKANILVSLESFDEALDVLEELKEYAPRESSVYALMGKIYKRRNMYEKAMLHFGLALDFKPSATDVATIKADIEKLHVPDEL
>SbCDC27 - estExt_fgenesh1_pg.C_chr_33723
METLMVDRVHSSLRLFMHRNAVFLCERLCAQFPSEVAPFSPCSSTRLVRLDSLWLRVGMTNVQLLATCYLHNNQPYAAYHILKGKKMPE
SRYLFATSCFRMNLLREAEETLCPVNEPNMEVPSGATGHYLLGVIYRCTGRISAAAEQFTQALTLDPLLWAAYEELCILGIAEDTDECF
SESTALRLQQEHTSTSTLVKSNFANENRVLSSRVSASLGDISPKQIKQLHANNIAEVSGYPHTSSIMPPPLFRNVHAYQNTVSGDAPTK
QKTNGVNQPLRRKNIDEARLKKGRRQWVSGRLFNSDSIPRRSERLKDTATNSNSNTSQFGGNGAGHSSGSRYEVIDEMWTDNISGISSS
VSATDGRSFEQDKAERILLQDSKLALGIRELLGLFRTLGEGFRLSCLFKCQEALEVYRKLPESQFNTGWVLCQVGKAYFELVDYLEADR
YFELAHRLSPLQNSWMCLGHNHNRLVWKLGPHVSSFSYVSVWNVQQVDERHYNAWYGLGVVYLRQEKFEFAEHHFRRAFQINPRSSVLM
CYLGMALHSLKRDEEALEMMEKAIAADKKNPLPKYQKALILLGLMKYEEALDELERLKEIAPHESSMFALMGKIYKQLNILDKAVFCFG
IALDLKPPAADLAIIKSAMEKVHLPDELMEDDLRFKAEFEPAPSHKFAFEETMPTTILVLDSYKPDISEYSSSNAGDFESDATRNASPL
GGHHDNFNSLRQKMCCTSSSQNTCRTQLLTPKARIWIHEFHGTCKQYVPSCGVISRRVTPLDIHGWIFKS
>SmCDC27 - fgenesh2_pg.C_scaffold_143000025
MEACLVDSINASLKLYMYRNATFLCERLYAESATEANLHLLATCYFRSNQAYRTYYLLKGIKSPQCRYLFALACFEMGNMAEAEAALQP
SDSPSEVPNGAAGCYLLGLICRFTDRRQAAIAHYTQALSVDPFFWSAYEDLCLLGVEEDPLSQSQWEVKSQYTYNTPSPSVSQLVTTKS

TVAPVHPQRRKFLDEGKLRKVSGRLFTEPPRRSLRLSAESPSTINVCSIPSPVAVSTPTSTASVRVSSNSSVRVSNSRKIISGSAEGPL
DDGRRVYESLETLGSDEMSNTSSQQSSPVIDEELRRSVSVGFVSRGSKLGEGATELLALLKVLGEGFKHVCMYESQEALEAFAKLPQNQ
YETGWVLCQIGRAYFEMVDYAEAERAFSWARRVSPYRLEGTDIYSTVLYHMKKDVELSYLAQEVVSMDRLSPQAWCVIGNCFSLQKDHE
TALKFFQRALQLDSHFTYAYTLCGHEYVAMEDFEEGLTCYRNAIRMDGRHYNAWYGLGTIYLRQEKYELAEYHFRRALQINERSSVLHC
YLGMALHALKRSHEALELLGEAIRADPKNPLPKYQKANVLMSEERYNDALGVLEQLKEVAPRESSVYFLIGKVYKRLGQPESAMYHFCV
ALDLKPSTADVNLIKNAIEKLHVPDESEEENL
>PpCDC27_1 - fgenesh2_pg.scaffold_136000020
MENYLHECVRSSLRAYLYANATFLCERLCAEFPSESNVHLLATCYFRSNKAHQAYHVLKGTKSRQCRYLFALACMEMQNLEEAEAALLS
SLEPGAESQPSSAASYYLLGVICKQSDRRQGAIGHYTQALSLDPFLWSAYEDLCGLGAEEEAASVFSDAASFQLQKVEFLGQLNAMQNE
HDDNDYSLRTPVSSNGSPKHRRLHSGGAVSDAGSAFMTPTSEGMCTPAPVPNLGLGLPGPGRSTNPSASPAGGGAGEGVGRGGGGTVGY
AQQRRKFVDPDGKFRKSLRSLSMRPGYTQIPGRLFEQGAPRRSTATLHYEKLFDSFEVSGGVDEGLSNSKLEGAEEEAPLSARCISRGA
SELFQLLRILGEGYRHLCMLRCQEAVQSFSKLPQQHFATAWVLCQVGRAYLEMVNYAEAERVYSWARRVSPHCSEGMDMYSTALYHMKK
DVQLSYLAQDAVAMDRLSPQAWCVMGNCFSLQKDHETALKFFQRALQLDPNFTYAHTLCGHEYVAMEDFEEGLTCYRKAIRLDSRHYNA
WYGLGTIYFRQEKYELAEYHFRRALFVNSRSSVLHCYLGMALHALKKNGEALALLEQAIVADPKNPLPKFQRANVLMSEGRCHEALAEL
EELKELAPRESSVFFLMGRIYKRLDMLERAIHHFCIALDLKPSATDVNLIKTAIEKLPIADDLEVENL
>PpCDC27_2 - e_gw1.31.82.1
MENYLYECVQASLRAFLYANATFLCERLNAEFPSESNVHLLATCYFRSNKAHLAYHVLKGTTTRQCRYLFALVCMQMQSLEEAEATLLN
SLEPGAESLPGSATSYYLLCVICKQSGRRQAAIGHYTQALSLDPFLWSAYEDLCGLGADEESVPVFSDTVKSQLQKIESVRQSNAVKHE
SFEHDFSLRAPASSNGSPKHRKLHSGGVISDAGSTVMISTSEGVSTYASVQNLGLGPPGPGRSNNPGANATGGGSGDGIGRGGGGTAGY
AQQRRKFVDPDVKFRKSLRSLSVRPGQSQASRRVVEQVGPRMSTRLNMIGDCNTAQLQQPCPTSSVGATMVGTVARSDEERSLSARCAS
RGALELFQLLRILGEGYRHLCMLRCQEAVQSFSKLPQQHFATAWVLCQVGRAYVEMVNYPEAERVYSWARRVSPHCPVGMDMYSTALYH
MKKDVQLSYLAQDAVAMDRLSPQAWCVMGNCFSLQKDHETALKFFQRALQLDPNFTYAHTLCGHELVAMEDFEEGLICYREAIRLDSRH
YNAWYGLGTIYLRQEKYELAEYHFQKALHVHSRSSVLHCYLGMALHALKKNDEALALLEQAIVADPKNPLPKFQRANVLMSEGRYREAL
AELEELKELAPRESSVFFLMGRIYKRLEMLERAVHHFRIALDLKPSSTDVNLIKAAIEKLPIADALEVENL
>VvCDC27 - GSVIVT00016677001
MEAILVDSVLGSLRHFLHRNAIFICERLCAEFPSETNLQLLASCYLHNNQAYAAYYILKGTQMAQSRYLFAISCFQMDLLTEAEAALCP
VNEPGAEIPNGAAGHYLLGLIYRYTDRKKSAVHHFKQALSLDPLLWAAYEELCLLGAAEEATAVFGEAAALCIQKQHLHHGLASQNLQT
SIEDRNLVSGRNLSSEDVSPRQLKHIHANNLREIPGNYHGAAMSGATASQSLNSGPSSTAFYNTPSPMVAQLSGVAPPPLCRNVQQNGL
NPSTVGNDSSPRSTVNPTIQAPRRKFVDEGKLRKISGRLFSDSGPRRSTRLAGEAGANTNPSGTTVAGNGTIHSSKYLGGAKSSSAAFR
SVTVRKGQTLANESFDEGTRQEVFDDSRSYISAATSTSTSTSGDPKSLEQDEATMTIGGVITNTSKIINGAAEVLNLLRTLGEGYRLSC
MYRCQDALDVYMKLPHKHYNTGWVLSQIGKAYFELVDYLGADRAFSSARQASPYSLEGMDIYSTVLYHLREDMKLSYLAQELISTDRLA
PQSWCAMGNCYSLQKDHETALKNFQRAVQLNSRFAYAHTLCGHEYVALEYFENGIKSYQSALRIDDRHYNSWYGLGMICLRQEKFEFAE
HHFRMAFQINPRSSVILCYLGTALHALKRSGEALYMMEKAILADKKNPLPMYEKANILLGLDNFDEALEVLEELKEYAPRESSVYALMG
KIYKRRNMYDKAMLHFGIALDLKPSAADVATIKAAIEKLHVPDEIEDNL
>OspCDC27 - e_gw1.06.00.221.1
MQHVGASPDDDGAHLSRVPEDGEASYDTGHYDVHRDQGTWSWQPSFAARTAEGTVLAFKALRPLAEGLRHLAMYRCEDALRSFEQLTRA
QYDTAYVLCAVAKAHAEMVDYPNAARYFEEARAADPHRLEGLDVYSTVLWHLKEEVKLSNLAQEVQGIDRLAPQTWCVLGNCFSLQKEH
ELALKFFQRAIQLDPKYTYAHTLSGHEYFANEDFEKSMNCYRAALRLDSRHYNAWYGLGTVYYRQEKYVMSEYHFRYALNINSKSSVLF
CYAGMAKHALGENSDAMTLLSQAIALDEKNPLARYEMAAVLMSEENYDQALEELQTLQEIAPKEASVFFLMGRIYKKLGLQEKAMINFS
IALDLRPSNADVNSIKSAIEKLDSDEVSDEEDI
>MspCDC27 - e_gw1.6.34.1
MTVIVPEQTPAGEAHLEPFLVELVHESLNSYAYSNAAFLCERLHAAAPTEANAHLLATCYYRADQANRAYHTLKGRTSPKCRYLFALCC
VKLRRLPEAEAALCRSPLPAGGRPPATDSSAAERPAANARATSDVPNGAHGLYLLGRVCKETGRDKAAAAHFADALALDPFMWCAYEEL
CALGAEAEAEAATEAALRSADRYPKLADLGKFGASFGGAAESMSFGTYGGETTSAATNSEGTSGLSGGGGGGTHSGASSNPNHALGGVL
GTVAEGTPTVEKSLGFAKPPHSGLPSASGPPVPMSTGGESAYYGAYGDEKTPAPDSTRGLMPPPPPSTATAAGVPADDLVTPAPIGGEA
DHGKPPGPPGGPGGGGGGGGGGGGRGDGGRRKFVDEGKLRKVSGQLFQESANAGSGAVRRSSRLAAQTGGGGGAGLDFSTPAMEAGSAE
GAEGADPTTSPPHHRARGRGGRSTHNPRGSLDGGSRPPLPLPPTSSSGHGGMNAGMNAGYGSNGAGRAGYASNGTALHAGHGGYVGCGR
FAEGAAATAALLRPLADGLRTFSMFRCEDALAHLRELPRSQYVTGYVLCLVGRAYAEMVNYPEAQRAFEWARTVCPHGLDGMEVYSTVL
WHLKKEVELSYLAQECVQLDRLAPQTWCVLGNCFSLQKEHETALRFFQRALQLDPRCTYAHTLCGHEFFANEDFEKAMGCYRNALRLDG
RHYNAWYGLGTVYYRQEKYELSEYHFRHALSINSRSSVLFCYLGMAQHALRRNADALTLLQHAIDLDKRNPLAKYEKASVLLSEDRLED
ALEELERLKEVAPREASVFFLIGRIHKKLGAADAAMVAFSTALDLKPASADVNLIKSAIEKLHVPDDSEEEDL
>CspCDC27 - e_gw1.12.33.1
MGSPGPPSAALEEHLVACVQHSLGLYLFDNACFLCERLVAQFPSEANLFLLATCYHRSNQSFRAYHLLKGLTGEQSRYLYALCAMQLGK
LTEAETALLPDNDASRVPNGGAGFYLLGRIHQLSNRHSAAIAYYSTALQLDPMLWSAFEELCGLGADHEAGQYLAAAGAAGTAAAATGA
AGPAATAGGAPHSAMGGGGMPSTSTSTFQHGVDSGGTPSPGSYVTPSPGGRPSAAPPPPAPKVGGPAAARPSWPATNTPAPLPVTAGGS
LAMPGTSGGGGGGGGAQRKFVDEGKLRKVSNKLFADPASMLKELRWQEGEAGGGASGSGASGGGGGGAAGAAGGSLADVAALHGVPRGQ
RSQEGQQQALPLLQALGEGYRLLCMYRCQEAVDALSRLPPHQYQTGWVLCCVGRAFFEMVDYPEAAKAFSWARQVDPYRLRGLEVYSTV
LWHCKREVELAQLAQAASSLDRHSPYAWCAMGNCFSLQKEHETALRYFQRALQLDPTLPYAYTLAGHEYFANEDFEKGITCYRNAIRID
PRHYNAWFGMGHIYYRQEKYGMAEYHFRRALSINDRSSVLRCYLGMALHKLKRSGEALETLGQAIAADPRNPLAKFERAAVLMAEDRWR
DALAELHALKDLAPREASVLFHMGKIYKKLDMLDEAMACFAHALDLQPPSADTNLIKGAIEKLRTPDDNEEEEI
>VcCDC27 - e_gw1.56.50.1
MAESRTALLQLLAPLMEGVRHLAAYRCSEALAALSRLPMSQARTAWVMGAMGRAHFESMNYAKAAQVRFESARQLDRTRVEGMEIYSTV
LWHTKREYELSHLAQECVATDRLAPQTWCVLGNLFSSQKEHEAAIEFFLRAAQVDPTFTYAYTLAGHEYFANEDYDKAAACYRSALKLD
PRHYKAMYGLGQIAYRQEKYAEALQNFRLAAGINPRSSVLRCYVGMSAAKLGQTPLALEKLQEAIDLDPANPLARFERASVLASLERIG
EALAELEALQRMAPGEASVAFQMGKLFKRLNNRTVSKSTYVHVCFGGRTSKSVKVLLSTLSTLLGVEQFPNCL
>CmCDC27 - CMR407C
MERDLVGRGAALECSLRRALQRQMPEEHVFFLAERLCAEKSTAESFALYAQALAQYGRHRQAVDVVAKYWQQDLECRYWYALCCIEANE
LAAAYKALSFLLQENANENLTKEVRSSLNNARGLPSERSLSVPWQAEGLYLLGRVFRLSNTRVDQAAQCFRRALEIDPFLWCCIEELSS
LYRYIDYFVPEMFGISGKEEQVFQSGRSSLRLDNCILRWKTRSSGSPQDNFQALATVRALVEGYHCREAVALIATLPLALQQAPVVLKW

QGRAYLDAGELSECARTFEKYLSLNRSGSLDGLEYYSTALWHMRRDVELNALARYALERDRFSAATWCIVGNAFSLQRDTDSAIEFFLR
AAQIDPRNPYPCTLAGHEYLYLDNYDAAMRCYQDALYRNSRHYNAWFGIGQVYQRQEKFRLAEKHYRIALDLNSNNSMLWYYLGHVIRV
GGGREVDALNALEKALEMNPRNPVARFECCKLYMQIGRLQDAWKELYQLRNMVPREAAIYYQMGVIARELGKNAVELFSIALDLDPKQ
PLYRQALLSLDETQSAP
>AtCCS52A1 - At4g22910
MEEEDPTASNVITNSNSSSMRNLSPAMNTPVVSLESRINRLINANQSQSPSPSSLSRSIYSDRFIPSRSGSNFALFDLSPSPSKDGKED
GAGSYATLLRAAMFGPETPEKRDITGFSSSRNIFRFKTETHRSLNSFSPFGVDDDSPGVSHSGPVKAPRKVPRSPYKVLDAPALQDDFY
LNLVDWSAQNVLAVGLGNCVYLWNACSSKVTKLCDLGAEDSVCSVGWALRGTHLAVGTSTGKVQIWDASRCKRTRTMEGHRLRVGALAW
GSSVLSSGSRDKSILQRDIRCQEDHVSKLAGHKSEVCGLKWSYDNRELASGGNDNRLFVWNQHSTQPVLKYSEHTAAVKAIAWSPHVHG
LLASGGGTADRCIRFWNTTTNTHLSSIDTCSQVCNLAWSKNVNELVSTHGYSQNQIIVWKYPTMSKIATLTGHTYRVLYLAVSPDGQTI
VTGAGDETLRFWNVFPSPKSQNTDSEIGSSFFGRTTIR
>AtCCS52A2 - At4g11920
MEEDESTTPKKKSDSQLNLPPSMNRPTVSLESRINRLIDSNHYHSPSKPIYSDRFIPSRSGSNFALFDLASSSPNKKDGKEDGAGSYAS
LLKTALFGPVTPEKSDVVNGFSPSGNIFRFKTETQRSLNLYPPFDSDVVSGVSPSPVKSPRKILRSPYKVLDAPALQDDFYLNLVDWSA
QNVLAVGLGNCVYLWNACSSKVTKLCDLGVDETVCSVGWALRGTHLAIGTSSGTVQIWDVLRCKNIRTMEGHRLRVGALAWSSSVLSSG
SRDKSILQRDIRTQEDHVSKLKGHKSEICGLKWSSDNRELASGGNDNKLFVWNQHSTQPVLRFCEHAAAVKAIAWSPHHFGLLASGGGT
ADRCIRFWNTTTNTHLNCVDTNSQVCNLVWSKNVNELVSTHGYSQNQIIVWKYPTMSKLATLTGHSYRVLYLAVSPDGQTIVTGAGDET
LRFWNVFPSPKSQSRESEIGALSFGRTTIR
>AtCCS52B - At5g13840
MASPQSTKTGLNLPAGMNQTSLRLETFSSSFRGISSLSSPSKSTCSDRFIPCRSSSRLHAFDLQDKEPTTPVKEGGNEAYSRLLKSELF
GSDFASPLLSPAGGQGSASSPMSPCTNMLRFKTDRSNSSPSSPFSPSILGNDNGHSSDSSPPPKPPRKVPKTPHKVLDAPSLQDDFYLN
VVDWSSQNVLAVGLGTCVYLWTASNSKVTKLCDLGPNDSVCSVQWTREGSYISIGTSHGQVQVWDGTQCKRVRTMGGHQTRTGVLAWNS
RILSSGSRDRNILQHDIRVQSDFVSKLVGHKSEVCGLKWSHDDRELASGGNDNQLLVWNNHSQQPILKLTEHTAAVKAITWSPHQSSLL
ASGGGTADRCIRFWNTTNGNQLNSIDTGSQVCNLAWSKNVNEIVSTHGYSQNQIMLWKYPSMSKVATLTGHSMRVLYLATSPDGQTIVT
GAGDETLRFWNVFPSVKMQTPVKDTGLWSLGRTQIR
>AtCDC20_1 - At4g33260
MDAGLNRCPLQEHFLPRKNSKENLDRFIPNRSAMNFDYAHFALTEERKGKDQSATVSSPSKEAYRKQLAETMNLNHTRILAFRNKPQAP
VELLPSNHSASLHQQPKSVKPRRYIPQTSERTLDAPDIVDDFYLNLLDWGSANVLAIALDHTVYLWDASTGSTSELVTIDEEKGPVTSI
NWAPDGRHVAVGLNNSEVQLWDSASNRQLRTLKGGHQSRVGSLAWNNHILTTGGMDGLIINNDVRIRSPIVETYRGHTQEVCGLKWSGS
GQQLASGGNDNVVHIWDRSVASSNSTTQWLHRLEEHTSAVKALAWCPFQANLLATGGGGGDRTIKFWNTHTGACLNSVDTGSQVCSLLW
SKNERELLSSHGFTQNQLTLWKYPSMVKMAELTGHTSRVLYMAQSPDGCTVASAAGDETLRFWNVFGVPETAKKAAPKAVAEPFSHVNR
IR
>AtCDC20_2 - At4g33270
MDAGMNNTSSHYKTQARCPLQEHFLPRKPSKENLDRFIPNRSAMNFDYAHFALTEGRKGKDQTAAVSSPSKEAYRKQLAETMNLNHTRI
LAFRNKPQAPVELLPSNHSASLHQQPKSVKPRRYIPQTSERTLDAPDIVDDFYLNLLDWGSANVLAIALDHTVYLWDASTGSTSELVTI
DEEKGPVTSINWAPDGRHVAVGLNNSEVQLWDSASNRQLRTLKGGHQSRVGSLAWNNHILTTGGMDGLIINNDVRIRSPIVETYRGHTQ
EVCGLKWSGSGQQLASGGNDNVVHIWDRSVASSNSTTQWLHRLEEHTSAVKALAWCPFQANLLATGGGGGDRTIKFWNTHTGACLNSVD
TGSQVCSLLWSKNERELLSSHGFTQNQLTLWKYPSMVKMAELTGHTSRVLYMAQSPDGCTVASAAGDETLRFWNVFGVPETAKKAAPKA
VSEPFSHVNRIR
>AtCDC20_3 - At5g26900
MDSGMRATCTVPEHFLPRKLSKQNLDRFIPNRSAKDFDFANYALTQGSKRNLDEVTSASRKAYMTQLAVVMNQNRTRILAFRNKPKSLL
STNHSDSPHQNPKPVKPRRYIPQNSERVLDAPGLRDDFSLNLLDWGSANVLAIALGDTVYLWDASSGSTSELVTIDEDKGPVTSINWTQ
DGLDLAVGLDNSEVQLWDCVSNRQVRTLRGGHESRVGSLAWDNHILTTGGMDGKIVNNDVRIRSSIVETYLGHTEEVCGLKWSESGNKQ
ASGGNDNVVHIWDRSLASSKQTRQWLHRFEEHTAAVRALAWCPFQASLLATGGGVGDGKIKFWNTHTGACLNSVETGSQVCSLLWSQSE
RELLSSHGFTQNQLTLWKYPSMSKMAELNGHTSRVLFMAQSPNGCTVASAAGDENLRLWNVFGEPPKTTKKAASKKYPELFSHVNSLR
>AtCDC20_4 - At5g27080
MDSDTCTVPDHFLPRKLSKQNLDRFIPNRSAMDFDFANYALTQGRKRNVDEITSASRKAYMTQLAVVMNQNRTRILAFRNKPKALLSSN
HSDSPHQNPKSVKPRRYIPQNSERVLDAPGLMDDFYLNLLDWGSANVLAIALGDTVYLWDASSGSTSELVTIDEDKGPVTSINWTQDGL
DLAVGLDNSEVQLWDFVSNRQVRTLIGGHESRVGSLAWNNHILTTGGMDGKIVNNDVRIRSSIVGTYLGHTEEVCGLKWSESGKKLASG
GNYNVVHIWDHRSVASSKPTRQWLHRFEEHTAAVRALAWCPFQATLLATGGGVGDGKIKFWNTHTGACLNSVETGSQVCSLLWSQRERE
LLSSHGFTQNQLTLWKYPSMSKMAELNGHTSRVLFMAQSPNGCTVASAAGDENLRLWNVFGEPPKTTKKAASKNYLELFSHVNSLR
>AtCDC20_5 - At5g27570
MMNTSSHLKAQASCPLVEHFLRRKLSKENFDRFIPNRSAMMDFDFANYALTQGRKRNVDEVTSASRKAYMTQLAEAMNQNRTRILAFRN
KPKALLSSNHSDPPHQQPISVKPRRYIPQNSERVLDAPGIADDFYLNLLDWGSSNVLAIALGDTVYLWDASSGSTYKLVTIDEEEGPVT
SINWTQDGLDLAIGLDNSEVQLWDCVSNRQVRTLRGGHESRVGSLAWNNHILTTGGMDGKIVNNDVRIRSSIVETYLGHTEEVCGLKWS
ESGKKLASGGNDNVVHIWDHRSVASSNPTRQWLHRFEEHTAAVRALAWCPFQASLLATGGGVGDGKIKFWNTHTGACLNSVETGSQVCS
LLWSKSERELLSSHGFTQNQLTLWKYPSMVKMAELNGHTSRVLFMAQSPDGCTVASAAGDETLRLWNVFGEPPKTTKKAASKKYTDPFA
HVNHIR
>AtCDC20_6 - At5g27945
MMKSIALALCLTHHSPMVLSIVDYGDSQEKTIDSLWKKPHGSDLTWSGFSRVGVTEAYFAVPVDHFLTNPPSLRLSQTIYCRRGSSYVE
TEKVEEEDRDDFLKQVWFLTDNLVFFFVDIEEYIVIEQLGDTVYLWDASSCYTSKLVTIDDENGPVTSINWTQDGLDLAVGLDNSEVQV
WDCVSNRHVRTLRGGHESRVGSLAWNNHILTTGGMDGKIVNNDVRIRSSIIGTYVGHTEEVCGLKWSESGKKLASGGNDNVVHIWDRSL
ASSNPTRQWLHRFEEHTAAVRALAWCPFQASLLATGGGVGDGKINFWNTHTGACLNSVETGSQVCSLLWSKSERELLSAHGFTQNQLTL
WKYPSMVKMAELNGHTSRVLFMAQSPDGCTVASAAGDETLRLWNVFGEPPKTTKKAASKKYTEPFAHVNHIR
>MtCDC20 - ABD32462
MNSRYPFLTNPSSSKDKWDRFIPNRSAMDFDYACYMVMEGTKVRKENPNRNSEYQKRLAEACNMNDRTRILAFKNKPPIPVELVPKELV
FPSPPPRPQSKPSKPRRISKTCTRIVDAPDISGDFYTNLLDWGSDNVISIALQNTVYLWNASDCSASELVTVDEEHGPVTSVSWSPDGC
HLAIGLNDSLVQFWDTTAERMVRTLRGGHRERVSALAWNGHTLTTGGMDGKIVNNDVRARSHIVETLKGHDRGVCGLKWSPSGQQLASG

GNDDVAQIWDRSVASSNSPTRWLHRFEEHESAVKALAWCPFQGNLLASGGGALDRCIKLWNTQTGEKLNSVDTGSQVCALLWNKNEPEL
LSSHGFHENQLILWKYSSMVKMAELKGHTSRVLHMTQSPDGDMVATAAADETLRLWNVFGTRKRVEPFTSFNRIR
>MtCCS52A - AF134835_1
MDGTGNRNPPPTSTVGDNSPPPEPSPESLRHVSRMINSNHYTSPSRTIYSDRFIPSRSASKFALFDINTPTEGRDDSSSAYTTLLRTAL
FGPDVAGPVTPEKTDSPSMTLPNRNIFRYKTETRQSMHSLSPFMDDDFVPGINHSPVKAPRKVPRSPYKVLDAPALQDDFYLNLVDWSS
HNVLAVGLGNCVYLWNACSSKVTKLCDLGVDDCVCSVGWAQRGTHLAVGTNNGKVQIWDAARCKKIRSMEGHRLRVGALAWSSSLLSSG
GRDKNIYQRDIRTQEDFVSKLSGHKSEVCGLKWSYDNRELASGGNDNKLFVWNQHSTQPVLKYCEHTAAVKAIAWSPHLHGLLASGGGT
ADRCIRFWNTTTNSHLSCMDTGSQVCNLVWSKNVNELVSTHGYSQNQIIVWRYPTMSKLATLTGHTYRVLYLAISPDGQTIVTGAGDET
LRFWNVFPSPKSQNTESEIGALSLGRTTIR
>MtCCS52B - AAQ72359
MSTITSLRLETLSTPPSSASPRAISNLSSTPSPSKSSKCSDRFIPCRSSSRLHTFGLIDNQSPVKEGSNEAYNRLLKSELFGPDFASPS
SSPAGCGVGSPLVSPSKNMLMFKTESCGPSSPFSLPSIFGRNDGFCNEGSTPPKPPRKVPKTPHKVLDAPSLQDDFYLNLVDWSSQNTL
AVGLGTCVYLWSASNSKVTKLCDLGPYDGVCSVQWTKEGSFISIGTNGGQVQIWDGTKCKKVRTMGGHQTRTGVLAWNSRILASGSRDR
NILQHDMRVPSDFIGKLVGHKSEVCGLKWSCDDRELASGGNDNQLLVWNQHSQQPTLRLTEHTAAVKAIAWSPHQSNLLVSGGGTPDRC
IRFWNTTNGHQLNSVDTGSQVCNLAWSKNVNELVSTHGYSQNQIMVWKYPSLAKVATLTGHSMRVLYLAMSPDGQTIVTGAGDETLRFW
NVFPSMKTPAPVKDTGLWSLGRTQIR
>ZmCDC20 - NP_001170191
MDAGTYSISSEKSHTAAAARPPLQEAGTRPYMPSLSTGSRNPSAKCYGDRFIPDRSAMDMDMAHYLLTEPRRDKENAVAASPSKEAYRR
LLAEKLLNNRTRILAFRNKPPVSENVSAAITASSHHAKLVKQRRHIPQSAERTLDAPELVDDYYLNLLDWGSNNVLSIALGDTVYLWDA
SSGSTSELVTIHEDSGPITSVNWAPDGHHIAIGLNSSDIQLWDTSSNRLLRTLRGVHEERVGSLAWNNNILTTGSMDGKIVNNDVRIRN
HVVQTYEGHSQEVCGLKWSGSGQQLASGGNDNLLHIWDVSMASPMSTAGRNQRLHRLEDHMSAVKALAWCPFQSNLLATGGGGSDRCIK
FWNTHTGACLNSVNTGSQVCALLWNKNERELLSSHGFTQNQLTLWKYPSMVKMAELSGHTSRVLFMAQSPDGCTVASAAADETLRFWNV
FGDPEVAKPAAKASHTGMFNSFNHIR
>ZmCCS52_1 - XP_002468612
MGNSAAGASSKPPTPASTPNSRLASAPSSRHAATPPHASAAASAPAPASRTVYSDRFIPSRTGSNLALFDLAPSPSSAASSSHSHDAGP
AASSGSAQPAASPYCTLLRAALFGPDTPDRVASSAAACSSSSSSFSPGPSPVGTPATGNIFRFKTEVRRNAKRALFSGDQEEDALFPGI
FTTRGAGPRKVPRSPYKVLDAPALQDDFYLNLVDWSSHNVLAVGLGNCVYLWNACSSKVTKLCDLGVDDNVCSVGWAQRGTHLAVGTKQ
GKVQIWDATRCKRIRTMESHRMRVGALAWSSSLLSSGSRDKSILHHDIRAQEDHVSKLTGHKSEVCGLKWSYDNRQLASGGNDNRLFVW
NPHSVQPVLKYTEHTAAVKAIAWSPHLHGLLASGGGTADRCIRFWNTTTNAHLSCVDTGSQVCNLAWSKNVNELVSTHGYSQNQIIVWR
YPTMSKLATLTGHTYRVLYLAISPDGQTIVTGAGDETLRFWNVFPSPKSQSSDSLSCVGGTSFVRSYIR
>ZmCCS52_2 - ACG33710
MAVAAPASDAKPRLNVPPSMAAALRLDPVGLGAGPSMPSPSRRLAEPPKTPSPSKTTYSDRFIPCRSSSRLQNFALIDSPLSPAKDDTP
YSRLLRAELFGPDSPKPTTATSAASASPNTNLFRFKKDHSAPTSPFAKAAAAHHDCTGGSGDAPSPQKPPRKVPKTPHKVLDAPSLQDD
FYLNLVDWSSQNVLAVGLGTCVYLWSASNSKVTKLCDLGPRDSVCAVHWSREGSYLSIGTGLGDVQIWDSSRCKRIRNMGGHQTRTGVL
AWSSCILSSGSRDKNILQHDIRVPNDYISKFSGHRSEVCGLEWSHDDRELASGGNDNQLLVWNQRSQQPVLRLTEHTAAVKAIAWSPHQ
QGLLASGGGTADRCIRFWNTANGNVLNSIDTGSQVCNLAWCKNVNELVSTHGYSQNQIMVWKYPSMSKVATLTGHTMRVLYLASSPDGQ
TIVTGAGDETLRFWNIFPSVRTQTPVRDIGLSSFSRSHIR
>SoCCS52_1 - SCCCLR1080G07
PTRPAQRGTHLAVGTNQGKVQIWDATRCKRIRTMESHRMRVGALAWSSSLLSSGSRDKSILHHDIRAQEDYVSKLTGHKSEVCGLKWSY
DNRQLASGGNDNRLFVWNPHSVQPVLKYTEHTAAVKAIAWSPHLHGLLASGGGTADRCIRFWNTTTNTHLSCVDTGSQVCNLVWSKNVN
ELVSTHGYSQNQIIVWRYPTMSKLATLTGHTYRVLYLAISPDGQTIVTGAGDETLRFWNVFPSPKSQSSDSLSCIGGTSFVRSYIR
>SoCCS52_2 - SCQGAM2028C12
EIPNSQPHLRDASSPQKPPRKVPKTPHKVLDAPSLQDDFYLNLVDWSSQNVLAVGLGTCVYLWSASNSKVTKLCDLGPRDSVCAVHWSR
EGSYLSIGTGLGDVQIWDSSRCKRIRNMGGHQTRTGVLAWSSCILSSGSRDKNILQHDIRVPSDYISKFCGHRSEVCGLKWSHDDRELA
SGGNDNQLLVWNQRSQQPVLRLTEHTAAVKAIAWSPHQQGLLASGGGTADRCIRFWNTANGNVLNSIDTGSQVCNLAWCKNVNELVSTH
GYSQNQIMVWKYPSM
>SoCDC20 - SCCCLR2C02F11
MDAGSHSISSEKSRAVAPRPPLQEAVSRPYMPSLGSGCRNPSAKCYGDRFIPDRSAMDMDMAHYLLTEPKKDKENVAASPSKEVYRRLL
AEKLLNNRTRILAFRNKPPEPENVSFADAASSNLQAKPAKQRRHIPQSAERTLDAPELVDDYYLNLLDWGSNNVLSIALGDTLYLWDAS
SGSTSELVTIDEDSGPITSVSWAPDGRHIAVGLNSSDVQLWDTSSNRLLRTLRGVHEARVGSLAWNNSILTTGGMDGKIVNNDVRIRDH
VVQTYEGHSQEVCGLKWSGSGQQLASGGNDNLLHIWDVSMASSMPSAGRNQWLHRLEDHMAAVKALAWCPFQSNLLATGGGGSDRCIKF
WNTHTGACLNSVDTGSQVCALLWNKNERELLSSHGFTQNQLTLWKYPSMVKMAELTGHTSRVLFMAQSPDGCTVASAAADETLRFWNVF
GAPETPKPAAKASHTGMFNSFNHIR
>VvCCS52_1 - GSVIVT00001067001
MDDPTTPSTTNQSALRSSSGVGYQPSPSRTIYSDRFIPSRTGSNFALFDISPLANSPAEGREDGSGAYATLLRTALFGPDAGVCSPGTP
DKLMRLDGKNSSVYPSSRNIFRYKTETRQSMHSLSPFGFEDALPGVSHGPVKAARKVPRSPYKVLDAPALQDDFYLNLVDWSAHNVLAV
GLGNCVYLWNACSSKVTKLCDLGMDVSVCSVGWAQRGTHLAVGTSNGKLQIWDASRCKRVRTMEGHRLRIGALAWSSSMLSSGSRDKTI
LQRDIRAQDDFVNKLAGHKSEVCGLKWSYDNRELASGGNDNRLFVWNQHSTQPVLKYCEHTAAVKAIAWSPHLHGLLASGGGTADRCIR
FWNTTTNSHLSCMDTGSQVCNLVWSKNVNELVSTHGYSQNQIIVWRYPTMSKLATLTGHTYRVLYLAISPDGQTIVTGAGDETLRFWNV
FPSPKSQNTDSEIGASSLGRTQIR
>VvCCS52_2 - GSVIVT00018459001
MDSSERRKSGLNLPAGMSETSLRLETFSGSYRAISNLSSPSKATSCSDRFIPCRSSSRLHTFGLIEKASPVKEGGNEAYFRLLKQELFG
SDFGSSPAGQGSPMSPSKNMLRFKTDHSGPNSPFSPSIFGPDSGFSSEASTPPKPPRKVPKTPHKVLDAPSLQDDFYLNLVDWSSQNVL
AVGLGTCVYLWTASTSKVTKLCDLGPSDSVCSVQWTREGSYISIGTHLGQVQVWDGTQCKKVRTMSGHQTRTGVLAWSSRILSSGSRDR
NILQHDLRVSNDFVSKLVGHKSEVCGLKWSHDDRELASGGNDNQLLVWNQHSQQPVLKLTEHTAAVKAIAWSPHQSGLLASGGGTADRC
IRFWSTTNGNQLNHVDTGSQVCNLAWSKNVNELVSTHGYSQNQIMVWKYPSMTKVATLTGHSLRVLYLAMSPDGQTIVTGAGDETLRFW
NIFPSMKTPAPVKDTGVWSMGRTHIR

>VvCDC20_1 - GSVIVT00002414001
MDAGSLTSSNKYQSKCPDQRRTVRENLDRFIPNRSAMDFDYAHYMLTEGRKGKENPAASSPSKEAYRKQMAETLNINRTRILAFKNKPP
TPVELIPQEFYSASIPQQSKASKPRRHIPQTSERTLDAPDLVDDYYLNLLDWGSSNVLAIALGGTVYLWDASDGSTSELVTLEDETGPV
TSVSWAPDGRHIAIGLNNSDVQLWDSTANRLLRTLKGGHASRVGSLAWNNNHVLTTGGMDGKIINNDVRVRSHIVETYRGHRQEVCGLKW
SASGQQLASGGNDNLLHIWDRSSASSNSPTQWLHRMEDHTAAVKALAWCPFQGNLLASGGGGGDRCIKFWNTHTGACLNSVDTGSQVCA
LLWNKNERELLSSHGFTQNQLTLWKYPSMVKMAELTGHTSRVLFMAQSPDGCTVASAAGDETLRFWNVFGTPEVAAKPAPKAHPEPFAH
LNRIR
>VvCDC20_2 - GSVIVT00014075001
MDAGSLNSCPQQVQFLQRWENLDRFIPNRSAMDFDFAHYMLTERGKGKENQSVVRSQSKEAYLKLLAETFNMNRSRILAFKNKPPTPVK
LIPDEFYSSVHQSKPSKPLRRIPQTPERTLDAPDIIDDFCLNLMDWGSSNVLALALQNTVYLWDASNGSASELVTVDDENGPVTSVSWA
ADGQYIAIGLNSSDVQLWDSTANRLLRTLRGGHQSRVGSLDWKNHILTTGGMDGQIINNDVRAHSHIVATFRGHRQEVCGLKWSTSGQQ
LASGGNDNLLYIWDRSMASMHSRSQWLHRLEDHTAAVKALAWCPFQRNLLASGGGGSDGCIKFWNTHTSACLNSVDTGSQVCALLWNKN
ERELLSSHGFMQNQMTLWMYPSMVKIAELTGHTSRVLFMAQSPDGRTVATAAGDETLKFWNAFGTPEVKKAAPKAEHPGPFPHLRRIR
>VvCDC20_3 - GSVIVT00038823001
MDAGSLSSLNCNSTYKCPLQEQFHRRRKTRENLDRFIPNRSAMDFDYAHYMLTKGRKGKENPSVLSPSIEAYLKLLANTFHMNRGRILA
FKNKPPTPVELTPREFLSPVRQFKPSKPKQHIPQTPERTLDAPDIIDDYYLNLLDWGSSNILAIGLGSTVHFWDGSNGSTSELVTVDDE
NGPVTSISWAADGQHIAIGLNNSDVQLWDSTANQLLRTLRGGHQSRVGSLAWNNNHILTTGGRDGKIINNDVRVRSHIVETYRGHHQEVC
GLKWSASGQQLASGGNDNMLYIWDRSMSSSNSRSQWLHRLEDHTAAVKALAWCPFQSNLLASGGGGNDLCIRFWNTHTGACLNTVDTGS
QVCALLWNKKERELLSSHGFSQNQLTLWKYPSMVKITELTGHTSRVLFMAQSPDGCTVVTAAGDETLKFWNVFGTTPEVKNAAPKELFP
HFSRIR
>VvCDC20_4 - GSVIVT00001962001
MDFDFAHYMLTERGKGKENQSVVRSQSKEAYLKLLAETFNMNRSRILAFKNKPPTPVKLIPDEFYSSVHQSKPSKPLRRIPQTPERTLD
APDIIDDFCLNLMDWGSSNVLALALQNTVYLWDASNGSASELVTVDDENGPVTSVSWAADGQYIAIGLKSSDVQLWDSTANRLLRTLRG
GHQSRVGSLDWKNHILTTGGMDGQIINNDVRVHSHIVATFRGHRQEVCGLKWSTSGQQLASGGNDNLLYIWDRSMASMHSRSQWLHRLE
DHTAAVKALAWCPFQRNLLASGGGGSDGCIKFWNTHTSACLNSVDTGSQVCALLWNKNERELLSSHGFMQNQMTLWMYPSMVKIAELTG
HTSRVLFMAQSPDGRTVATAAGDETLKFWNAFGTPEVKKAAPKAEHPGPFPHLRRIR
>OsCCS52A - LOC_Os03g03150
MDHHHHHLPPPPPRSPMENSASSKPPTPASTPSSRLAAAPSSRVSSAAPHPSPSSSAPTPASRTVYSDRFIPSRAGSNLALFDLAPSPS
HHDAAAAAASPGAPPPSGSTPASSPYCALLRAALFGPTTPDRVASSASACSSSSSAGASPVGSPATGNIFRFKAEVPRNAKRALFSDGD
DEGVLFPGVFTTRGTGPRKIPRSPYKVLDAPALQDDFYLNLVDWSSHNILAVGLGNCVYLWNACSSKVTKLCDLGVDDNVCSVGWAQRG
THLAVGTNQGKVQVWDATRCKRIRTMESHRMRVGALAWNSSLLSSGSRDKSILHHDIRAQDDYISRLAGHKSEVCGLKWSYDNRQLASG
GNDNRLYVWNQHSAHPVLKYTEHTAAVKAIAWSPHLHGLLASGGGTADRCIRFWNTTTNMHLNCVDTGSQVCNLVWSKNVNELVSTHGY
SQNQIIVWRYPTMSKLATLTGHTYRVLYLAISPDGQTIVTGAGDETLRFWNVFPSPKSQSSDSLSSIGATSFVRSYIR
>OsCCS52B - LOC_Os01g74146
MATDASPKPAPPRLNVPPAMAGGLRLDPAVASPARLLLDVPKTPSPSKTTYSDRFIPCRSSSRLHNFALLDRDRASPSSTTDDAPYSRL
LRAEIFGPDSPSPAPSSPNTNLFRFKTDHPSPKSPFAASAAATAGHYDCTAGSAESSTPRKPPRKVPKTPHKVLDAPSLQDDFYLNLVD
WSSQNTLAVGLGNCVYLWSASNCKVTKLCDLGPRDSVCAVHWTREGSYLAIGTSLGDVQIWDSSRCKRIRNMGGHQTRTGVLAWSSRIL
SSGSRDKNILQHDIRVPSDYISKFSGHRSEVCGLKWSHDDRELASGGNDNQLLVWNQRSQQPILRLTEHTAAVKAIAWSPHQQGLLASG
GGTADRCIRFWNTVNGNMLNSVDTGSQVCNLAWCKNVNELVSTHGYSQNQIMVWKYPSMSKVATLTGHTLRVLYLAMSPDGQTIVTGAG
DETLRFWNIFPSMKTQAPVRDIGLWSFSRSHIR
>OsCDC20_1 - LOC_Os09g06680
MDAGSHSISSEKSHGLAPRPPLQEAGSRPYMPSLSTASRNPSAKCYGDRFIPDRSAMDMDMAHYLLTEPKKDKENAAASPSKEVYRRLL
AEKLLNNRTRILAFRNKPPEPENVSAADTASTHQAKPAKQRRYIPQSAERTLDAPDLVDDYYLNLLDWGSKNVLSIALGDTVYLWDASS
GSTSELVTVDEDSGPITSVSWAPDGQHVAVGLNSSDIQLWDTSSNRLLRTLRGVHESRVGSLAWNNNILTTGGMDGNIVNNDVRIRNHV
VQTYQGHSQEVCGLKWSGSGQQLASGGNDNLLHIWDVSMASSVPSAGRNQWLHRLEDHTAAVKALAWCPFQSNLLATGGGGSDRCIKFW
NTHTGACLNSVDTGSQVCALLWNKNERELLSSHGFTQNQLTLWKYPSMVKMAELTGHTSRVLFMAQSPDGCTVASAAADETLRFWNVFG
SPEAPKPAAKASHTGMFNSFNHLR
>OsCDC20_2 - LOC_Os04g51110
MDAGSHSISSEKSSRYVAPRQPLQEAGSRPYMPSLSTASRNPSAKCYGDRFIPDRSAMDMDMAHYLLTEPRKDKENAAASPAKEAYRKL
LAEKILNNRTRILSFRNKPPEPESILTELRADAASIQAKPAKQRRYIPQSAERTLDAPELVDDYYLNLLDWGSSNVLSIALGNSVYLWD
ATNSSTSELVTVDEDNGPVTSVSWAPDGRHIAVGLNSSDVQLWDTSSNRLLRTMRGVHDSRVGSLAWNNNILTTGGMDGKIVNNDVRIR
NHVVQTYQGHQQEVCGLKWSGSGQQLASGGNDNLLHIWDVSMASSMPSAGRTQWLHRLEDHLAAVKALAWCPFQSNLLASGGGGSDRCI
KFWNTHTGACLNSIDTGSQVCSLVWNKNERELLSSHGFAQNQLTLWKYPSMVKMAELTGHTSRVLFTAQSPDGLTVASAAADETLRFWN
VFGAPEAPKTATKGSHTGMFNNSNHIHIR
>OsCDC20_3 - LOC_Os02g47180
MEAPGSGSVPTAKRRRLVPRPPPVPLEVAGARGPYMPPLCIKSKNPSAKCYGDRFIPDRSAMDMDMAYFLLTEPKKEKENTDMLSPAEE
AYKRLLAEKLLNNRSRILAFRNKPPEPEGIVQQLLYETLTSSQTKPARKCRHIPQSSERTLDAPGIVDDFYLNILDWGCKNVMSIALGN
TLYLWNSADGSIMDLVTIDEDDGPITSVSWSCDGQWIAVGLNSSDIQLWDTSSNRMLRTLHGVHQSRVGSLAWNKNILTTGGMDGNIVN
NDVRMRSHVVHIYRGHEDEVCGLRWSGSGQQLASGGNDNLVHIWDVSMASSNLSLGHNRWLHRFGDHLAAVKALAWCPFQSNLLASGGG
GDDRCIRFWNTHTGLCLNSVDTGSQVCGLLWNKNEKELLSAHGYVQNSLALWKYPSMVKLAELEDHTARVLCLAQSPDGFTVASVAADE
TLRLWKIFETSEDAKPVFKTVNTGMFNSFSHIR
>VcCCS52 - e_gw1.19.8.1
MEPEYASAAGASGHAQPSSPLRDHSRHNLHTSSPLRSPIWSPKSRVTYSDRFIPSRAAAARLDYSILDREMATSDDLNPAYNLLLRSEL
LGSSCPGPISPDKVRDPGWLRAPASPGKSLFRFKTGDVHSPHGGPAAQSPFVVSPVGDDTAAGSPFASPRRAQRKIARAPFKVLDAPSL
ADDFYLNLVDWSSQNVLAVGLGTCVYLWSAMSSTVTKLCDLAPHDTVCSVEWSRRGTFLSVGTNSGKVQIWDVAKLKLVRTLEGHRARV
GTQAWGSHVLCSGSRDRHILQRDIRCPEHFTAKLVGHRSEVCGLKWSPDDRQLASGGNDNQLYIWSLPSSSPVYKFADHTAAVKAIAWS
PHQHSLLASGGGTADRCIRFWNTATGMPLNCIDTGSQVCNISWSKNANEIVSTHGYSQNQVIIWKYPSMAKLATLTGHTLRVLYLAVSP
DGQTIVTGAGDETLRFWSVFPSAKSSGPDTTVASMSRTTIR

>VcCDC20 - estExt_fgenesh4_pg.C_490111
MSGVQPPTPGSKAGGSPGGGNKSVKLSFGTKTPGNGKGKEKLDRFIPTRSAMDFDSANYTLNKDAKQSSDQRDGQGSPSKEDYQKALAA
SLSVNDSSRILAFKQKAPAAPEGYENNLKSLYNQNLAPNAAKKQFRHVPTTQERILDAPELMDDYYLNLLDWGSQNLIAVALGRSVYLW
NAGSGNVEELCTVPNEGDYISSLRWGSDGNFLAVGTSDAKVQIWDATRRKQVRELCGHTNRVSCLSWNGSILSSGSRDSTIANWDVRKR
RDEACVATLRVHEQEVCGLTWSLCGQQLASGGNDNILAIHDASFSLVNKVQAHTAAVKALAWCPYQSNLLATGGGTADRHVRFWNTHTC
AMLSQIDTGSQVCALQWNPHERELLSSHGYSKHQLCLWKYPSLVKVAELSGHQGRVLHMATSPDGCSVVTAGADETLRFWRPFGEPPAP
KDADSKLPTNNHGNQLPTGQNHIHDVLSTPSASK
>CspCDC20 - e_gw1.22.48.1
MRARGGRGGLTVGPGWAGEIAWGCGRQRSPPALQMDRFIPNRSASNLDVANYNVSREAKDVENLDALSPTKLEYKKQLAANLGQDDSAR
ILAFKQKAPAPAEGFENHMAALYSANAGPRPKKAFRAVPQQPDRILDAPDLVDDYYLNLLDWSSTNAVAVALNQAVYLWNASSGDIQEL
LSAQGEDYITSLSWAADGKHLAVGYSSALTQIWDAERCKPVRNLGGHAARVSSLSWNNHTLSTGGRDSLILHHDVRVREHVTATLRGHE
QEVCGLKWSPNGTQLASGGNDNLLMIWDAAADRATHRITAHQAAVKALAWCPFQSNLLATGGGTADRTIKFHNTHTGALLNSIDTGSQV
CSLQWNRHERELLSSHGFSQNQLCLWKYPSMAKVAELTGHTSRVLHLAQSPDGTTVVSAAADETLRFWRCFGEPAGAAPKGGAKPGAAA
GSLLRSVNIR
>CspCCs52 - estExt_fgenesh3_pg.C_320025
MDLASTSGGGAQEGGGGSGGADGASGSGLPEPQQHQAFSSPVKQRPAAFGQTPLRSPKVVYSDRFIPSRATSSRLDFSILDREAAASET
PRRTAEKEESNGAYNMLLRSELLGCPAAPVSPEKGAGAAGVAGVASTSGAHPVSPSKSPSRKLFRYMAGDVDTPTAGLPTQSPYARGPI
GGDDSVASTLASPTRMLRKIPRAPFKVLDAPALADDYYLNLVDWSAQNTLAVGLGTCVYLWSACTSKVTRLVDFGEGGGVCSVSWSQRG
SYLSIGSDKGEVQVWDTTKCKRIRTFPGHKQRVGCMAWSHHTLATGSRDRSILLRDVRAPEPYVQKLGGHRSEVCGLRWSPDDRELASG
GNDNQLFVWHQHSAQPVLRFSEHQAAVKAIAWSPHQHGLLVSGGGTADRCIRFWNTTTGQALQCIDTGSQVCNLSWSKNINELVSTHGY
SQNQIIVWRYPTMQKLATLTGHTMRVLYLAVSPDGQTIVTGAGDETLRFWNVFPGPKAQGSGSDSGMGSMMRTLIR
>OspCDC20 - fgenesh1_pg.C_Chr_04.0001000310
MFTQEAYKKSLADNFHVERGSDSAKILAFKSKAPAPPSGMENSARGVYTNNSAGVRAKKTCRQIPSAPERILDAPELIDDYYLNLIDWG
SSNQVAVALGCTVYMWNADTGAINQLCQTNPADEEDYITSVNWGADGKHIAVGTNSAEVQIWDASQCKKVRTLRGHAARVGAISWNGSQ
LATGGRDNTIMIHDVRIREHCTSTLRVHQQEVCGLKWSPSGNQLASGGNDNLLHIFDATSIGNRQALHRLDAHQAAVKALAWCPFQSNL
LASGGGTADRCIKFWNTNTGAMLNSVDTHSQVCSLQWNTHERELLSSHGYSQNQLCLWKYPTMTKMAELTGHQARVLHMAQSPDGTTVV
SAAADETLRFWKCFDNSVEKTKKVRDANDSSVLRRFNFR
>OspCCS52 - fgenesh1_pm.C_Chr_13.0001000041
MLGHRSRAGTLAWNSHTLSSGSRDRAILNRDIRSPSDYSNKLLGHKSEVCGLKWSYDDQQLASGGNDNQLFVWNSHSSSPTLRCSEHTA
AVKAIAWSPHQHGLLASGGGTADRCIRFWNTVTNTPLQCIDTGSQVCNLVWSKNVNEIVSTHGYSQNQIVVWRYPSMSKLTTLTGHTLR
VLFLAISPDGQTIVTGAGDETLRFWNVFPGVKSQVSGAGDNTVCALARTSIR
>MspCCS52 - e_gw2.06.248.1
MSTPGTPNVKTPGGKRPTVYSDRFVPSRSASAGLQGFNLLDTGSPPASASHPNSGEREDTSAAYSTLLRSEMLGENGASILGASPVNPS
KKLFRFKSDGVLSEGINAADSPYSLSPVGGDDALNGRHPQRRSHRKIARSPFKVLDAPALQDDFYLNLVDWSSHNVLAVGLGTCVYLWS
ACTSRVTKLCDLGPGDSVCSVGWTQRGTYLAVGTNNGEVQIWDATRCKRIRSMGGHRTRVGTLAWSSSTLSSGSRDRNILQRDVRAPEH
YTSKLSGHKSEVCGLKWSYDDRELASGGNDNQLYVWSANSTHPLLRYSDHTAAVKAIAWSPHQHGLLASGGGTADRCIRFWNTTTNTPL
SCIDTGSQVCNLVWSKNVNELVSTHGYSQNQIIVWRYPNMSKLATLTGHTLRVLYLAISPDGQTVVTGAGDETLRFWNVFPGPKSQGSV
HDNSVWSMGRTHIR
>MspCDC20 - fgenesh2_pm.C_Chr_05000098
MLHRFGDENAPKTPGKARPDRFIPNRSALDLDIAHYNLVKENANANDLDLAAEVASPSKEEYKKQLASNFLSQDGGASSAKILAFKSKA
PEPPAGMENSARGVYTNNAGAGVKSKKTFRHVPSAPERILDAPELIDDYYLNLIDWGSTNQVAVALGCVVYLWNADSGDIQQLCQTDPN
NGDDYVTSVQWGGDGKHIAVGTNDAEVQIWDVSRLKQVRTLRGHNARVGALAWNGTQLATGSRDNTVMMHDVRIREHRTATLTSHSQEV
CGLKWAPSGNQLASGGNDNLLHIWDQNSIGNGTHLHRLDAHQAAVKALAWCPFQSNLLASGGGTADRCIKFWNTNTGALLNSIDTHSQV
CSLQWNKHERELLSSHGYSQNQLCLWKYPTMTKMAELTGHSARVLHMAQSPDGTTVVSAAADETLRFWKCFSDSDAGKAKKLKDGASDS
SVLRRFNFR
>PtCCS52B - Pt820353
MDSTPRRKSGLNLPSGMNETSLRLETFSSSSSFRAVTCVSSPRAISSLSSPSKTSSCSDRFIPCRSSSRLHTFGLVEKGSPVKEGGNEA
YARLLKSELFGSDFGSFSSPAGGQGGLSSPNKNMLRFKTDHSGPNSPFSPSILGHDSGISSESSTPPKPPRKVPKTPHKVLDAPSLQDD
FYLNLVDWSSQNVLAVGLGTCVYLWTASNSKVTRLCDLGPNDSVCSVQWTREGSYISVGTHLGQVQVWDGTQCKRVRTMGGHQTRTGVL
AWNSRTLASGSRDRHILQHDLRISSDYVSKLIGHKSEVCGLKWSHDDRELASGGNDNQLLVWNQHSQLPILKLTEHTAAVKAIAWSPHQ
SGLLASGGGTADRCIRFWNTTNGHQLNYVDTGSQVCNLAWSKNVNELVSTHGYSQNQIMVWKYPSLSKVATLVGHSLRVLYLAMSPDGQ
TIVTGAGDETLRFWNVFPSMKTQTPVKDTGLWSLGRTQIR
>PtCCS52B_2 - Pt833809
MDSTPRRKSGLNLPSGMNETSLRLETFSSSSSFRAVTCVSSPRAISSLSSPSKTSSCSDRFIPCRSSSRLQTFGLIEKGSPVKEGGNEA
YARLLKSELFGSDFGSFSSPAGGQGGLGSPSKNMLRFKTDHSGPNSPYSPSILGHDSGISSESSTTPKPPRKVPKTPHKVLDAPSLQDD
FYLNLVDWSSQNVLAVGLGTCVYLWTASNSKVTRLCDLGPNDSVCSLQWTREGSYISIGTHLGQVQVWDGTQCKRVRTMGGHQTRTSVL
AWNSRTLASGSRDRNILQHDLRVSSDHVSKLIGHKSEVCGLKWSHDDRELASGGNDNQLLVWNQHSQLPVLTLTEHTAAVKAIAWSPHQ
SGLLASGGGTADRCIRFWNTTNGHQLNHVDTGSQVCNLAWSKNVNELVSTHGYSQNQIMVWKYPSLSKVATLVGHSMRVLYLAMSPDGQ
TIVTGAGDETLRFWNVFPSMKTQTPVKDTGLWSLGRTQIR
>PtCCS52A1_1 - Pt415429
MADPMISQSTQSKLNIAASTTPRHHHHLHLENPTPSPSSSKHVNRLINSNHYISPSRPIYSDRFIPCRSSSNFALFNISFPQPSATAGI
SPGCGGKEDNPSAYAALLRNALFGPQTPDKKDWGAGAAGQNIFRYKMETRQSLHSLSPFGFDDMSDLGVSNVAIKTPRKVSRSPYKVGV
LVNWCHILTFLRFDEIWIFIGIFLLIDPAFCFYVLDAPALQDDFYLNLVDWSSHNVLAVGLGNCVYLWNACSSKVTKLCDLGNDDGVCS
VGWAHRGTHLAIGTSNGKVQIWDASRCKRIRTMEGHRLRVGALAWSSSMLSSGSRDKSILQRDIRAREDFVSKLSGHKSEVCGLKWSYD
NRELASGGNDNRLFVWNQHSSQPVLKYCDHTAAVKAIAWSPHLHGLLASGGGTADRCIRFWNTTTNSHLSCIDTGSQVCNLVWSKNVNE
LVSTHGYSQNQIILWRYPTMSKLATLTGHTFRVLYLAISPDGQTIVTGAGDETLRFWSVFPSPKSQNTDSEIGASSLGRTTIR
>PtCCS52A1_2 - Pt180625
MADPTMSPPNQSQLNVAASTTQRRQHHLNLESLAPSPSSSEHVNRLINSNHYISPSRPIYSDRFIPCRSSSNFALFNISLPSPSATAGS
SPGDGGKEDNPNAYAALLRNALFGPQTPDKKDWGTGAAGRNIFRYKTETRQSMHSLSPFGFDGLSGPGVSNVAIKAPRKVSRSPYKVLD

APALHDDFYLNLVDWSSHNVLAVGLGTCVYLWNACSSKVTKLCDLGNDDGVCSVGWAHRGTHLAVGTSNGKVQIWDASRCKRIRIMEGH
RLRVGALAWSSSMLSSGSRDKSILQRDIRAQEDFVSKLSGHKSEVCGLKWSYDNRELASGGNDNRLFVWNQHSTQPVLKYCEHTAAVKA
IAWSPHLHGLLASGGGTADRCIRFWNTTTNSHLSCMDTGSQVCNLVWSKNVNELVSTHGYSQNQIIVWRYPTMSKLATLTGHTYRVLYL
AISPDGQTIVTGAGDETLRFWNVFPSPKSQNTESEIGASSLGRTTIR
>PtCDC20_1 - Pt571123
MDAGSLNSSSYMKAQSRFPLQEQFLHRKNSKDNLDRFIPNRSAMDLDYAHYMLTQGRKGGKENPTATVNSPSREAYRKQLAEALNLNRT
RILAFKNKPPTPVELIPRDHLSSSLHYQAKPTKPRRYIPQTSERTLDAPDLVDDFYLNLLDWGSKNVLAIALENTVYLWDASNGSTSEL
VTVGDEVGPVTSVNWAPDGLHLAIGLNNSNVQLWDSASCKQLRNLRGCHRSRVGSMAWNNNHILTTGGMDGKIINNDVRIRSHIVETYRG
HQQEVCGLKWSASGQQLASGGNDNIIHIWDRSVASSNSATQWFHRLEEHTSAVKALAWCPFQGNLLASGGGGGDRSIKFWNTHTGACLN
SIDTGSQVCALLWNKNERELLSSHGFTQNQLVLWKYPSMLKMAELTGHTSRVLYMAQSPDGCTVATAAGDETLRFWNVFGVPEVAKAAP
KANPEPFSRFNRIR
>PtCDC20_2 - Pt272847
MDAGSINSSSSLKAQSRFPLQQQFLPRMNSKENLDRFIPNRSAMDMDYAHFMLTEGRKGKENPTVNSPSREAYRKQLAESLNMNRTRIL
AFKNKPPAPVELMPQDHSHHHHQPKTAKPRRHIPQTSERTLDAPDLVDDFYLNLLDWGSSNVLAIALGSTVYLWDASDGSTSELVTVDD
EDGPITSVNWAPDGRHIAIGLNNSHIQLWDSASNRQLRTLKGGHRSRVGSLAWNNNHILTTGGMDGQIINNDVRIRSHIVETYRGHTQEV
CGLKWSASGQQLASGGNDNLIHIWDRSTALSNSATQWLHRLEDHTSAVKALAWCPFQGNLLASGGGGGDKSIKFWNTHTGACLNSIDTG
SQVCSLLWNKNERELLSSHGFTQNQLTVWKYPSMVKMAELTGHTSRVLYMAQSPDGCTVATAAGDETLRFWNVFGVPEVAAKAAPKANP
EPFSHLNRLR
>PtCDC20_3 - Pt738273
MDAGSMNTSSSLKAQSRFPLQQQFLPRTNSKENLDRFIPNRSAMDMDYARFMLTEGRKGKENPTVNSPSREAYRKQLADSLNMNRTRIL
AFKNKPPAPVELMPQDHSHHHHQPKTAKPRRHIPQTSERTLDAPDLVDDFYLNLLDWGSSNVLAIALGSTVYLWDASDGSTSELVTVDD
EDGPVTSVNWAPDGRHIAIGLNNSHIQLWDSASNRQLRTLKGGHRSRVGSMAWNNNHILTTGGMDGQIINNDVRIRSHIVETYRGHTQEV
CGLKWSASGQQLASGGNDNLIHIWDRSTALSNSATQWLHRLEDHTSAVKALAWCPFQGNLLASGGGGGDKSIKFWNTHTGACLNSIDTG
SQVCSLLWNKNERELLSSHGFTQNQLTVWKYPSMVKMAELTGHTSRVLYMAQSPDGCTVATAAGDETLRFWNVFGVPEIAAKAAPKANP
EPFSHLNRIR
>PtCDC20_4 - Pt256238
MDSSSSSTTTRMFHPRSALRENPQRKKSYENVSLQNYILDRFIPNRSAMDMDFAHYMLTEGRKAKESPPSQSLYQKLLAEAFNMNGRRI
LAFKNKPPTLVDPIPLFSSSSVHSSKPVKPQRHIPQRPEMTLDAPDIVDDFYLNLLDWGNNNVLAIALGTTVYLWNASNSSISEVVTVD
EEDGPVTSISWAPDGRHLAVGLDNSNVQLWDSATNQMLRTLRGGHRLRVTSLAWNHHLLTTGGKDAKVINNDVRIREHIVESYEGHRQE
VCGLKWSASGQQLASGGNDNLLFIWDRFMASSNSPRHWLHKLEDHTAAVKALAWCPFQSNLLASGGGGNDRHIKFWNTQTGTCLNSVDT
GSQVCALQWNKHERELLSSHGFTENQLILWKYPSMVKMAELSGHTSPVLFMTQSPDGYTVASAAGDETLRFWNVFGNPKAAKPAPKAIA
EPFANVSHFR
>PtCDC20_5 - Pt257786
HIWDRSVASSNSATQWFHRLEEHTSAVKALAWCPFQGNLLASGGGGGDRSIKFWNTHTGACLNSIDTGSQVCALLWNKNERELLSSHGF
TQNQLVLWKYPSMLKMAELTGHTSRVLYMAQSPDGCTVATAAGDETLRFWNVFGVPEVAKAAPKANPEPFSRFNRIR
>SbCCS52_2 - Sb03g047370
MTLAAPASDAKPRLNVPPSMAAALRLDPVGLGAGPSTPSPSRRLAEPPKTPSPSKTTYSDRFIPCRSSSRLQNFALLDSPLSPAKDDTP
YSRLLRAELFGPDSPKPATAASASPNTNLFRFKKDHSAPTSPFAKAAAAHHDCTAGSGDAPSPQKPPRKVPKTPHKVLDAPSLQDDFYL
NLVDWSSQNVLAVGLGTCVYLWSASNSKVTKLCDLGPRDSVCAVHWSREGSYLSIGTGLGDVQIWDSSRCKRIRNMGGHQTRTGVLAWS
SCILSSGSRDKNILQHDIRVPSDYISKFCGHRSEVCGLKWSHDDRELASGGNDNQLLVWNQRSQQPVLQLTEHTAAVKAIAWSPHQQGL
LASGGGTADRCIRFWNTANGNVLNSIDTGSQVCNLAWCKNVNELVSTHGYSQNQIMVWKYPSMSKVATLTGHTMRVLYLASSPDGQTIV
TGAGDETLRFWNIFPSVKTQTPVRDIGLWSFSRSHIR
>SbCCS52_1 - Sb01g048980
MDHHLTPSPPSPMENSAAGPSSKPPTPASTPNSRLASAPSSRHSATTSHASAPSSAPTPASRTVYSDRFIPSRTGSNLALFDLAPSPSA
ASSSHEGGPAVSSGSATAASPYCALLRAALFGPDTPDRVASSATACSSSSSPGPSPVGTPATGNIFRFKTEVRRSAKRALFSGEEEEDA
LFPGIFTTRGAGPRKVPRSPYKVLDAPALQDDFYLNLVDWSSHNVLAVGLGNCVYLWNACSSKVTKLCDLGVDDNVCSVGWAQRGTHLA
VGTNQGKVQIWDATRCKRIRTMESHRMRVGALAWSSSLLSSGSRDKSILHHDIRAQEDYVSKLTGHKSEVCGLKWSYDNRQLASGGNDN
RLFVWNPHSVQPVLKYTEHTAAVKAIAWSPHLHGLLASGGGTADRCIRFWNTTTNTNLSCVDTGSQVCNLAWSKNVNELVSTHGYSQNQ
IIVWRYPTMSKLATLTGHTYRVLYLAISPDGQTIVTGAGDETLRFWNVFPSPKSQSSDSLSCIGGTSFVRSYIR
>SbCDC20_1 - Sb04g009980
MDAGTYSISSEKSHKAAKAAAAPRPPLQEAGSQPYMPSLSTGSRNPSAKCYGDRFIPDRSAMDMDVAQYLLTEPRKDKENAAAAASPSK
EMYRRLLAEKLLNNRTRILAFRNKPPEPENVSATIAASAHHAKPAKQRRHIPQSAERTLDAPELVDDYYLNLLDWGSNNVLSIALGDTV
YLWDASTGSTSELVTIDEDSGPITSVSWAPDGKHIAVGLNSSDVQLWDTSSNRLLRTLRGVHEARVGSLAWNNSILTTGGMDGKIVNND
VRIRNHVVQTYEGHSQEVCGLKWSGSGQQLASGGNDNLLHIWDVSMASSMPSAGRNQWLHRLEDHTAAVKALAWCPFQSNLLATGGGGS
DRCIKFWNTHTGACLNSVDTGSQVCALLWNKNERELLSSHGFTQNQLTLWKYPSMVKMAELTGHTSRVLFMAQSPDGCTVASAAADETL
RFWNVFGAPEAPKPVKASHTGMFNSFNHIR
>SbCDC20_2 - fgenesh1_pg.C_chr_4002652
MDAGSHSISSEKSRAAAAPRPPLQEAVSRPYMPSLGSGCRNPSAKCYGDRFIPDRSAMDMDMAHFLLTEPRKDKENAAASPSKEAYRRL
LAEKLLNNRTRILAFRNKPPEPENVSFADAASSNLQAKPAKQRRHIPQSAERTLDAPELVDDYYLNLLDWGSNNVLSIALGDTLYLWDA
SSGSTSELVTIDEDSGPITSVSWAPDGRHIAVGLNSSDVQLWDTSSNRLLRTLRGVHEARVGSLAWNNSILTTGGMDGKIVNNDVRIRD
HVVQTYEGHSQEVCGLKWSGSGQQLASGGNDNLLHIWDVSMASSMPSAGRNQWLHRLEDHTAAVKALAWCPFQSNLLATGGGGSDRCIK
FWNTHTGACLNSVDTGSQVCALLWNKNERELLSSHGFTQNQLTLWKYPSMVKMAELTGHTSRVLFMAQSPDGCTVASAAADETLRFWNV
FGAPEAPKPAAKASHTGMFNSFNHIR
>SbCDC20_3 - Sb06g027390
GDRFIPERSAMDMDLAHYLLTEPRKGKENPTARVSPAKVAYRKQLAEKLLNNRTRILAFRNKPPEPESMLTDPRADATQAKPTKQRRQI
PQSAEKVLDAPELVDDYYLNLLDWGSSNVLSIALGYKVYLWDASSQSVTELVTIDEDSGPVTSVSWAPDGRHIAIGLNSSDVQLWDSTS
NRLVRTLRGVHESRVGSLAWQNSVLWHGGMDGKIMNNDVRIRNNAVQTYHGHEQETCGLRWSGSGQQLASGGNDNLLHIWDVSMSSSVQ
TAGHTQWLHRLQGHLSAVKALAWCSFQSNLLASGGGGDDRCIKFWNTHTGACLNSVDTGSQVCALLWNKNERELLSSHGFTQNQLILWK
YPSMVKLAELNGHTSRVLFMAQSPDGCNVASGAADETLRIWNVFGTPETVPKAPYTGIFNSFNHIR

>SmCDC20_1 - e_gw1.8.1605.1
MAAQGTATCSTPGRAPCLISNPPRKTPGKLQLQSDRFIPDRSAMNFDVANMLVLGKENSHSQQQQQQQQHLRYDCCQEEYKKQLAENLL
KDANILHKESRILAFKNRPPPPPEGFDKESSLLYSENTAPGASRPRKMFRHIPQAPERTLDAPEILDDYYLNLLDWGSNNVVAVALGHT
VYLWNASTGNIEELMQANEEDGPVTSVAWAPDGKHISVGLSNADVQLWDSLSLRQVRSLKAHSARVGSLAWNGPILSTGGRDNVIFNHD
VRIREHVTGKMVAHQQEVCGLKWSPSGQQLASGGNDNLLHIWDAAAAVSGGTSSYLHRLDEHQAAVKALAWCPFQSNLLASGGGTADRC
IKFWNTHTGACIQSVDTGSQVCALQWSKHERELLSSHGFSQNQLILWKYPSMVKMAELTGHTSRVLHLAQSPDGYTVASAAGDETLRFW
QVFGNPDTAKAAVRTKARETYSALNSRCIR
>SmCDC20_2 - fgenesh1_pm.C_scaffold_0000129
MAALISSNGGKDPAATPAKKLPQIDRFIPDRSAMNFDVANMLVLGKENSQLQQQPLRSDCSQEEYKKQLAENLLRDANIFQKKSRILAF
KSKPPPPPEGFERESRLLYSENTAPGASKPRRMFRQIPQAPERTLDAPEILDDYYLNLLDWGTNNILAVALGHTVYLWNATTGGIEELM
QVSEDDGPVTSVSWAPDGKHISVGLNNADVQLWDAFSLRQVRSLKAHTARVGSLAWNGPILSTGGRDNVIFNHDVRIREHVTGKMAAHQ
QEVCGLKWSPSGQQLASGGNDNLLYVWDAAAAASRGNSTYLHRLDEHQAAVKALAWCPFQSNLLASGGGTADRCIKFWNTHTGACIQSV
DTASQVCALQWSKHERELLSSHGFSQNQLILWKYPSMVKMAELTGHTSRVLHMAQSPDGYTVASAAGDETLRFWQVFGTPDTTKATAVR
TKASSSALNSRCIR
>SmCDC20_3 - e_gw1.13.45.1
MASRTRLSPAPLSPLARRPSISMVASSSHIFHVIGDIQGDRFIPNRGAMDLDLAHFNLLHEARENSHTPSEVASPVKEDYRRILAESLL
SCETGSPKILAFTKKIPSLSIQRCLDTELDILPSSKKPHRHICQTPERILDAPEIVDDYYLNLLDWSCNNTVAVALGPAVYLWDADTGE
SFQLSKCEEHDTVTSVAWSDDGRLIAVGLSSACIQLWHATSRSQIRTFRGHSSRVSSLAWNGSLLSSGSRDHKIINHDVRARAHKASVL
AGHCQEVCGLKWSPCGQQLASGGNDNLLHIWDAAVASTFDSIHPGSRCAFRFDCHRAAVKALAWCPFQSRLLASGGGTVDRCIKFWNTQ
TGTCLSSIDTLSQVCALQWSRHQKEILSSHGYGLNQLCVWKYPSMIRIAELRGHTARVIHLAQSPEGTTVASAAADETLRFWRVFGSPN
KKCGSDMKPSGLSRSASSIR
>SmCCS52_1 - e_gw1.2.298.1
MEDCEDFSEEIKGLEAESPRAKIAARRSLVFSSSGFNAGKVESAAAVASSPKVNRITYSDRFIPSRSSSNLTGFALLDKSPSSNVSNVS
HGSETREDSAAAYSMLLRTELFGSDPGGPMVPNTPEKLLGGSSRDSARTPMSPTRNLFRFKNEHRGGAGACASPESPFSLSPVGLDVAL
AGTVTSPRKAPRKIARSPYKVLDAPALQDDFYLNLVDWSSLNVLAVGLGPCVYLWSACTSKVTKLCDLSPNDGVCSVGWTQRGTYLAVG
TNLGEVQIWDATRCKRVRTMGGHRTRVGTLAWSSNVLSSGSRDRNILQRDIRAPEDFVNRLVGHKSEVCGLKWSYDDRELASGGNDNQL
FVWNQLSTQPVLKFSEHTAAVKAIAWSPHQHGLLASGGGTADRCIRFWNTGTSTHLSCVDTGSQVCNLVWSKNVNELVSTHGYSQNQII
VWRYPAMSKLSTLTGHSYRVLYLAISPDGQTIVTGAGDETLRFWNVFPCPKSQSAVRNTGIWSLGRTHIR
>SmCCS52_2 - e_gw1.36.131.1
MAAERLRVEISRAVSVLNPAVAGGGGGGSTPSPTRMSYSDRFIPSRSSSNLAGFALLDNSPSLGSDASKEEGVAVYSMLLRSELLGGDA
GPLSPGTPDKNSLVSAGVRDSTKSPLSPSRNLLRFKSDSRAAGAPCHKSESLSPGALDFGTTGASSSPVKLQRKVSRVPFKVLDAPALQ
DDFYLNLVDWSAHNVLAVGLGNCVYLWSASTSKVTKLCDLGHDDAVCSVAWTHRGTYLAVGSNAGEVQIWDAIRCKKVRTMEGHRTRVG
TLAWNSVTLSSGSRDRNILNHDVRVPEDYTSKLCGHKSEASSVCGLKWSFDDRELASGGNDNQLFVWNQLSTQPVCKFSEHTAAVKAIA
WSPHQHGLLTSGGGTADRCIRFWNTSTNSHINCVDTGGQVCNLMWSKNVNELVSTHGYSQNQIIVWRYPTMTKLTTLTGHTYRVLYLSM
SPDGQTIVTGAGDETLRFWNVFPSPKSQNAVRDSGMWSLGRTHIR
>PpCDC20_1 - e_gw1.27.202.1
MDHINLDQTNFLSHQSSYSILADRFITDRSAMDFNIANYMLAGLEENAVNNGSVHSPSKEEYKKQLAENLLRCNNHQRQSRILAFKSKP
PPPPEGFENSRKSLYSQNASPGESKPRAYFRHIPQTAERTLDAPDLLDDYYLNLLDWSANNVLAIALGNTVYLWDATTCSIAELLTADE
DGPVTSVHWAPDGRYLAVGLNNADVQLWDSQELRQLRSLKGHSARVGSLAWNGPVLSSGSRDSSIINHDVRIRDHVIGRMEAHEQEVCG
LKWSPSGHQLASGGNDNLLYIWDASAASNQGPSPYLLRLDDHRAAVKALAWCPFQSNLLASGGGTADRCIKFWNTHTGVCLNSIDTQSQ
VCALQWSKHEREILSSHGFSQNQLCLWKYPSMVKLAELTGHTSRVLHLAQSPDGYTIASAAGDETLRFWKVFGDPEALKAKSRSKAKEV
GSVLHSLTRIR
>PpCDC20_2 - e_gw1.73.92.1
MHLAPRSQILAGGYDRFITDRSAMDFNVANLMLTKENSSMDVISPSRDEYKKQLAESLLNNNGQKQSRILAFKSKPPPPPEGLLNNNRT
LYSQNVGAAQFKPKKMFRHIPQAPERTLDAPDMVDDYYLNLMDWSSSNVLAIALGMTVYLWDATTSSIEELVTVDEEGPITSVSWAPDG
QYLAVGLNNSTVQLWDSTSLRQLRTLRGHSARVGALAWNGPTLATGGRDNAILNHDVRIRDHVIGSMEAHEQEVCGLKWSPSGQQLASG
GNDNILHIWDASAASSASASPLHSLDEHQAAVKALAWCPFQSNLLASGGGTADRCIKFWNTHTGACVNSIDTHSQVCALQWSKHEKEIL
SSHGFSQNQLCLWKYPSMVKMTELSGHTSRVLHLAQSPDGYTVASAAGDETLRFWNVFGTPETKEVNISHRTKKVGSALTSLTRIR
>PpCDC20_3 - e_gw1.84.190.1
MELTKKLEIFFVVDIEVCLDFIPDGPFHHDRFITDRSAMDFEVANYLLSKENSSSEATSPMKMAYRKHLAENLLNDNCQKQSRILAFKS
KPPPPSEGFQNARTTLYSQNVGAGDQKPRKTFRYIPQAPERTLDAPDLLDDYYLNLLDWSSNNVLAIALGMTVYLWDATTSSIEELMTV
DEEGPITSVSWAPDGQYIAVGLNNSTVQLWDSTSLRQLRTLRGHSARVGALAWNGPTLATGGRDSTILNHDVRIRNHVIGKLTGHEQEV
CGLKWSPSGQQLASGGNDNLLHIWDSAAASNSSSYLHRLDDHQAAVKALAWCPFQSNLLASGGGTADRCIKFWNTHTGVCVNSIDTQSQ
VCALQWSKHEKEILSSHGFSQNQLCLWKYPSMVKMAEFTGHTSRVLHLAQSPDGYTVASAAGDETLRFWQVFGTPETKQTSQKRTKEVG
SALTCLNRIR
>PpCDC20_4 - e_gw1.8.222.1
MLLTIFLNSPPQTDRFIADRSAMDFNVANLMLTRENSSVDVISPSKDEYKKQLAESLLNNNGQKQSRILAFKSKPPPPPEGFQNGRQTL
YSQNVSAQSKPKKMFRHIPQAAERTLDAPDMLDDYYLNLLDWSSGNVLAVALGMTVYLWDATTSSIEELMTVDEEGPITSVSWAPDGQY
LAVGLNNSSVQLWDSTTLRQLRTLRGHSARVSALAWNGPTLATGGRDNMILNHDVRIRENVIGCMAAHEQEVCGLKWSPSGQQLASGGN
DNLLHIWDASAASSLSASPLHSLDEHQAAVKALAWCPFQSNLLASGGGTADRCIKFWNTHTGACVNSIDTQSQVCALQWSKHEKEILSS
HGFSQNQLCLWKYPSMVKMAEFTGHTSRVLHLAQQSPDGYTVASAAGDETLRFWNVFGTPEAKLVNQAKKTREVESALTSLTRIR
>PpCCS52_1 - e_gw1.160.27.1
MKHGVCTTTSSPTRTSYSDRFIPSRSSSNLTGFALLDRSPSGANISQANDTREEGGAAYSMLLRTELFGQEAGSPATPENRTGISARDP
MRSPISPTVSRNLFRFKSDPKPSTGSKARPERPYDISPAGIDSSLAGTPMSPRKAPRKIARSPYKVLDAPALQDDFYLNLVDWSSNNVL
AVGLGTCVYLWSASSSKVTKLCDLGPTDSICSVSWTHRGTYLAVGTNLGEVQLWDAAKCRIVRTMGGHRTRVGALAWNSHILSSGSRDR
NILQRDVRVPDDFVSKLVGHKSEVCGLKWSYDDRELASGGNDNQLLVWNRQSTQPVVKFSEHGAAVKAIAWSPHQHGLLASGGGTADRC
IRFWNTATSTALNCYDTGSQVCNLVWSKNVNEIVSTHGYSQNQIIVWRYPTMSKLTTLTGHTMRVLYLAISPDGQTIVTGAGDETLRFW
NVFPSPKSQSVVRDNEVWSLGRTYIR

>PpCCS52_2 - fgenesh1_pm.scaffold_118000004
MKQGISSAATSSPTRTSYSDRFIPSRSSSNLTGFALLDRSPSGLNIAQLNDVREDGGAAYSMLLRSELFGQEAASPATPEKSMGISLRD
PMRSPISSSVSRNLFRFKSEPKPSTGPNARPENLFDLSPVGIDSALVAATMSPRKAPRKIARSPYKVLDAPALQDDFYLNLVDWSSQNV
LAVGLGTCVYLWSACTSKVTKLCDLGPTDSVCSVGWTQRATYLAVGTNLGEVQLWDATKCRKVRTMGGHRTRVGTLAWSSHLLSSGSRD
RNILQRDVRVPEDFVSKLIGHKSEVCGLKWSYDDRELASGGNDNQLLVWNQQSTQPVVKFSEHGAAVKAMAWSPHQHGLLASGGGTADR
CIRFWNTATSTALNCYDTGSQVCNLVWSKNVNELVSTHGYSQNQIIVWRYPTMSKLATLTGHTMRVLYLAISPDGQTIVTGAGDETLRF
WNVFPSPKSQSAVRDTGVWSLGRTHIR
>PpCCS52_3 - fgenesh1_pg.scaffold_112000011
MIGAASTLSPTKTSYSDRFIPSRSSSNLSSFSLLDRALSGANIVQSSDSKEDGSGAYSMLLRSELFGQVGSSPVTPEKSFGIGSRDSMR
SPISPKSRNMFRFKSECKPSTGLNSQPESPYSLSPVGIDSTMTGATVSPRKAPRKIARSPCKVLDAPALQDDFYLNLVDWSSSNVLAVG
LGTCVYLWSACSSKVTKLCDLGLTDSVCSVGWTQRGTYLAVGTNLGDVQIWDATRYRKVRTLGGHRTRVGALAWSSHMLSSGSRDRSIF
QRDVRSPEDFVSKLVGHKSEVCGLKWSCDDRELASGGNDNQLLVWNQHAAQPIVKFSEHTAAVKAIAWSPHQNGLLASGGGSADRCIRF
WNTATSSHLNCYDTGSQVCNLAWSKNVNEIVSTHGYSHNQIIVWKYPTMSKLATLTGHSMRVLYLATSPDGQTIVTGAGDETLRFWNVF
PSPKSQSAVRDTGVWSLGRTHIR
>CmCDC20 - CMA138C
MESLDVIEANIGENAPVPRWKRRQQRQLAEQLARADSARPVSIRSHDDNETRSVLERSNSAAAVLRERNQITSPSLVHRKRSRSTLPSP
ASHAASPRCDRFIPDRSAMDLELGRFVMTAALGRSITPGTGPAETLESVEGSQASRTEVTVERPAWGSGAFSAPEHAVHTHELLDDRAN
RPGASSSSPTSSTYQRSGTLSSAQSWASSLDTFSSIPSTADELTGEWQTSDVRSPARLAAAAEKIHAPGAVDPRQLAQSLEYRSQLASE
LGTAGLLGSVIDSPSDEPWQAHEDTSGLGSRQTAKRGNTAESMPGSGTKILAFKPKPPVHLGDKAAHAAIAVVYTQNRLGAACRRHLHR
HIPSAPERILDAPEMVDDYYLNLLDWSANNVLAVALGSAVYLWNASTGGIEQLTDLAPGDQHTNQDYVCSLKWVQGSGCAPHANAEASA
PYLAVGTAFGHVQIWDVEANKRLRTLRTHQGRVGSLHWNGPLLCSGSRDSTVQLHDVREARHLASTLVAHEQEVCGLQWSPNGMQLATG
GNDNLLMVWDRRALQHPRLRFDEHTAAVKALGWCPWQSHLLASGGGTTDRMLRFWNTHTGVCLQAVDTESQVCALQWSMHYRELVTGHG
FSRNQLVVWKYPDLNKVAELTGHGARVLHLTTSPDGQTVASAAADETLRFWKIFPKPQTSRFGVGKALLAGTARETLVPEDTRQLAHAG
TASSSSSSQRSHAGDRSASATGRLPFTAMWKSLGSAESAPAGDTDASPAVEGTRFTEASASAFVRVSAARTADASRTGDTASMTTLEQL
SSLRNELNRRIGSRPLTTMRSSAIR
>CmCCS52 - CMQ242C
MSTAWTPRRSRRSGDRFIPVRERDSALGLDSTQLRFQLQVATELSFQGRNTPSRDIAAESENNASDVGQVTESSPSRRLRDTSPMHQRR
LLMAARARHVEGSEALATFVPGHQIESELSLAQAAERYIENARRDWSDIFGASPSAPALLVSGSERAGQRSLQLETSPDADEGTLTYQS
RLGRSVNEPPVESSRESGTSPNRGLAAAERAGVRAELETLLATLSVGRASDARNGAYDGSERPARTSRAEQPPARRQSRRERELADLLE
RMDREQRDANHSVSSSSRSSTVEQIRALLQAAAELGDAADDVAVVDLAMELFADPRALYSMETLRGSVQHLAGTLMRGTLASPGGTGIS
SLAPGMLATDVGSRVTELQGVITWSEGGTVTRPGAANANAVLALSAHLPPHAQHATIESATRSANEAAYLTQTSGTGGAAPNTSTPGIS
PSTTGRFVVSAPGGPSTYAIILENELLHEVNTTRTELATDMDALVAENESTSPTEPPPSGRRPEPGFGLATPPRHSNPTRATRNSPVAA
SPGASHVSSSSATPVVARRSLLEFSRQDTSVHESSPSPRHRQYSENNGSPIRLDALRTSPVLAADPTIARQLGLGLRRKQRKISRVPFK
VLDAPNLADDFYLNLLDWSARNILAVGLGNSVYLWNAYNSKVSKLCELDTPPQGVCSVSWAPSGDLIAVGLASGVVHLYDPTRQEAAQM
LTGHTARVGCLAWNGPLLASGSRDRTIMEHDVRAGREPVRTLEAHRQEVCGLRWSFDQTQLASGGNDNKLFIWTPQARRPLFRFEEHEA
AVKAVAWSPHQHCLLASGGGTADRCIRLWNTTTGSLLQCVDTGSQVCNLLWSRAVNELVSTHGYSQNQIVLWRYPSMQKVVTLTGHLLR
VLYLAASPDGSVIVTGAGDETLRFWNVFPPPRSAARMSQQRAASSLSPSRLGGTRSLYAAALADLTTLPSAYAPTPSQASPMHDLWNTS
AMIPSSFIR
>AtApc1 - At5g05560
MPPGVRQLTVLGKFKPFGLIAEATDGKSPDDSYQYFLFDPELTGERDDADGNDANFSRQREHELFIRDNCNVRKGYALVEANFIGYWYN
ACWSNLGRGTEAFLCVLQIACLTIYNTSGEVVSVPLMRTVKSIWPLPCGLLLEQAGEVNPPSHVPFSPVSPILGSREMLRQRKEVGNSS
PQNFHSPVAHDLISKRDMPCMSSHLILRDPLEEPGPTYVEERGKLTIMKDYDERTIWTSDRLPLMTSYNKGKMQHSVWAAEFIESNLEA
SASCSSGIVPDAVLSKRVSFRRIWQAKGAKKAASKVFLATDNSVPVICFLILEQKKLLSVGLQTVEINNEILFDVKPDISWSVSAIAAA
PVVVTRSQVKIGLLPHLDIIVLSPENDLFLYSGKQCLCRYVLPSWLGESIGSGDGESAKTDSGFRNLKITGLSDAVLGSINLSVNHSQI
FRCALTGKPSSSLANDCIAAIAEGLRSDLYSLFLSLLWGDGHSDLQGSSIHFEWEALCNIFLEICQKPTVVHRKQPKTASESSWEFLLI
SKFHKTYSRFHNGITSINRLDLEGIVPFDSKICSEETLGSSCELMVQSLDCLHAVYESLKMDNLRKQDLHHLAVLLCNIAKFLDEKCYL
DYYIRDFPRLSTTIGACTTLSSSRKPPNLFRWLENCLRRGCLSTNFDDLPDLIRRDGCSIVSWARKVVSFYSVLFGDKPEGRTLSSGVP
CNIAPGSYSCNEELTILAMAGERFGLHQLDLLPSGVSLPLRHALDSCRESPPADWPAIAYVLLGREDMALSVFRNFSSSKEFEMQSNTS
LISMSIPYMLHLHPVIVPSSESIGLENTKIEDTNSVDGSVIDGMEHIFNSYTQLRYGRDLRLNEVRRLLCSARPVVVQTAANPTISDQE
QQQAFTVPKLVLAGRLPSQQNAIVNLDPNIRNIQELKTWPEFHNAVAAGLRLAPLQGKVSRTWIRYNKPGEPNAVHAGLLFGLGLQGYL
HVLNLSDIYQYFTQDHESTTVGLMLGLAASYRGTMQPDIAKALFFHVPARYQASYTEFEIPTLLQSAALVSVGMLFEGSAHQQTMQLLL
GEIGRRSAGDNVLEREGYAVSAGFSLGLVALGRGGDALGSMDSLVNRLLLYLGAKEERSILVPSLEDHRSAAQITDGSTSNVDITAPGA
IIALTLMYLKTESEVIFSKLSIPQTHYDLECVRPDFIMLRVIARNLIMWSRICPTCDWIQSQVPEVVKNGISQLRDDMDNMYEVDVEAL
VQAYVNIVAGACISLGLRFAGTRDGNARDLLNSYALYLLNEIKPLSATPGNAFPRGISKFVDRGTLEMCLYLIIISLSVVMAGSGDLQV
FRLLRFLRSRNSADGHANYGTQMAVSLATGFLFLGGGMRTFSTNNGSLAMLLITLYPRLPSGPNDNRCHLQAFRHLYVLATEARWLQTI
DVDSGLPVYAPLEVTVKETKLYSETKFCEITPCILPERAILKRICVCGPRYWPQQIELVFGLRTLGESNLIANSHRELDSDSVDHLVST
FSSDPSLIAFAQLCCDKSWNNSFDFLILDLILWSQVALAYNEAVSTGRLASSGGFVQSIFLASLRKRCEEVLNCSTELKINLRNYLTSE
AWPYDKNSKLQKDIIILSWYLKWFNVPSPSIIKAAVEKIKSKSKNSTSAIPLLRLLLPNTHISVIGEIDRVFFPSN
>OsApc1 - TC286185
CSARPVAIQTPNNPSVSDQDLQQQQLWNFAQRTTALPFGRGAFTLATTYTLLTEALVFPKLVLAGRLPAQQNATVNLDLSTRSVSEFKS
WAEFHNGVAAGLRLAPFQEKMLRTWIQYNRPSEPNFTHAGLLLAFGLHEHLRVLTMTDAYRYLSQEHDITRLGLLLGLAASNRGTMHPA
ISKMLYFHVPSRHPSSTPELELPTLLQSAAVMGIGLLYEGSAHALTMKILLGEIGRRSGGDNVLEREGYAVAAGSALGLVALGRGSNAF
GFMDTFLDRLFEYIGSKEVYHEKHLNAAIAADEQSGNTGQMMEGAQINVDVTAPGAIIALALIFLKAESEEIAARLSVPNSHFDLQYVR
PDFVMLRIVARNLILWNRIQPTKDWVESQVPSFVNFGVSNTSQEAMDSDELDSEALFQAYVNIVTGACIALGLKYAGSRNSDAQELLYA
YAVHFLNEIKHISIQTASILPKGLLQHVDRGTLELCLHLIVLSLSLVMAGSGHLQTFRLLRYLRGRSSAEGQVNYGLQMAVSLAIGFLF
LGGGTHTFSTSNSAVAALLITLYPRLPTGPNDNRCHLQAFRHLYVIATEPRWIQTVDVDTGLPVYCPLEVTVAETEYYDETNYCEVTPC
LLPERSVLKNIRVCGPRYWSQVITLTPEDKPWWKSGDRTDPFNGGVLYIKRKVGSCSYSDDPIGCQSLLSRAMHEVCDTPSTSCSNQAN
SATRSSLRVDQLVSTFSANPSLIAFAKLCCQSWKDRRNGSFEEFCSQILYECMSKDRPALLQVYISFYTIIETMWEHLKIGHFPFSDSL

FLSSLKVASAYNEALIDGRITTGGIIQSTFLESLMKRIEYIFAELPNLHDSFINYLNKGKWPDAQNEAVLLSWYLQWYSIPPPHIVSSA
IEKVKPRTRTSLSMLPLLRLLLPTTHLVGLMEIEKLHMTHGHEGLTLH
>PtApc1 - Pt765590
MAVRVCELTVLGEFKPFGLIAEALDGKPPDTDPDDYDYFLFDPEIARDRNEIDETDTCGSALRDRSDHELFIRGNKIIWSTGARVFKRF
TLPSPVIMACWCHLGDLSEALLCILLTDSLTIYNISGEVVSIPIPCTITSIWPLPFGLLLQSASENSPMQNHLSSPSPLFGVCDMSRAK
REIVHSPHHNFGVLGTFDHVIKGDSAIMSSHLILKDLLEEPHLMHVEERGKLTIMKDFDERTIWTSNRIPLMASYNKGKMQHSLWVAEI
INSNFEAENASLSGAALDDVLDKNFSFRRIWQGKGAQTAASKVFLATDDDAAPVICFLLQEQKKLLSVKLQSLEINNEIIFDIKPDVSW
SVAAVAAAPVSVTHPRVKVGLLPYTDIVVLAPDNSLLLISGKQLLCKYLLPSFFGKGHLSHNLEFSETASVPLDSKILGLTDAVEGRVN
LILNNGQMFRCTLRRSPSSSLVNDCITAMAEGLSSGFYNHFLALLWGDSNSDYLSRADSSVDSEWNSFCNIILQMCRKPSATSQKHSDL
ENLEQHSSWEFLVNSKFHKNYHKLNFISRVSSSELSFDPEKMDSFGSNMEGNRSSENSFYFELLQESLDCLHALYESLKLDKLRKRDLE
LVAVLLCNIAKFLGEGNYLDHYIRDFPGLISKIGTCEMPFSQKTPPSLFRWLENCMQHGCSSANTDDLPPLICKDGNFVVSWARKIVSF
YSLLCGGKQTGKKLSSGVYCNIAMGSCCTSEELTVLAMVGERFGLQQLDSLPSGVSLPLRHALDKCRESPPTDWSAAAYVLLGREDLAL
SRSALPCKSGELETQPNVNLISMSTPYMLHLHPVTIPSTVSDTTGLESAKFEDSDSADGSMMDGMEHIFNSSTQLQYGRDQRLNEVRRL
LCSTRPVAIQTSVNPSASDQDIQQILILLCLLLSLLLVKAQLWHLAQRTTALPLGRGAFTLATISTLLTEAFTVPKLVLAGRLPAQQNA
TVNLDPNIRNIQELKSWSEFHNAVAAGLRLAPLQGKVSRTWIIYNKPEEPNAIHAGLLLALGLHGYLRVLVISDIYTYFTQEHESTTVG
LMLGLAASYRKTMHPAISKSLYFHIPSRHSSSFPDLELPTLVQSAALVSAGLLYEGSVHPPTMQILLGEIGRRSGGDNVLEREGYAVSA
GFSLGLVALGRGEDALGFLNSLVDRLFQYIGGKEMHNERPLFLTPSMDEQNHGAGQMMDGTAVNVDVTAPGAIIALALMFLKTESEAVV
SRLSIPQTHFDLQYVRPDFIMLRVIARNLIMWSRVHPSNDWIQSQIPNIVKSGVNGLEDHVNDMDEMDAETFVQAYVNIVAGACISLGL
RFAGTKDGNAQELLYEYAVYFLNEIKHVCATSGNAFPKGLSRYVDRGTLEICLHLIVLSLSVVMAGSGHLQTFRLLRFLRSRNSADGHA
NYGTQMAVSLAIGFLFLGGGMRTFSTSNSSIAALLITLYPRLPTVPNDNRCHLQAFRHLYVLATEARLLQTVDVDSGLPVYAPVEVTVR
ETEHYSETSFCEVTPCILPERAILKSVRVCGPRYWPQVMELVPEDKPWWSIGETNDPFNSGVIYIKRKVGACSYVDDPIGCQSLLSRAM
HKVFGLTNIKVGDPSTSDHSGPGSVTVDQLVSAFSSDPSLIAFAQLCCDPSWNCKSDVEFQEFCLQVLFECISKDRPALLQVYLSLYTT
IGSMTDQVTNGTFILGDSLALSSLKHTECGCHLGHGAKADQCLGLVSFMLELHDNHHKLLALTYNEALLSGRLTTPRGSIIQSVFLGSL
KKRVEELLHCSEGLKIDFCNYLNFGRWPNDQTEGEKNSVLLSWYLQWFAVPSSSIIKTAMERVKPKLVSASSVPLLRLLLPRTHINAIG
EIDKLLVSPQGVKWGATAPVSKRNLLEVLLLARGGITMASDLPALMVGGSELDSTRNGAR
>AtApc2 - At2g04660
MEALGSSDCNLEILETLSDDAIQEITESYDGFFTTVESLIAGTGDSLVEDEFVSHVYCLCKYGLDSLVRDHFLRSLEQAFEKGGASSFW
QHFDAYSEKKHHNYGEEIQIVLCKALEEISIEKQYHEKCLSIVVHALQSFKEQSSDDRQNSDTERVHLFSRFQSMLSSTLMTTLPQHFP
EILHWYFKERLEELSAIMDGDGIEEQEDDCMDLDEKLRYKNGEMDVDEGCSQGKRLGHDKLVKNIGKVVRDLRSIGFTSMAENAYASAI
FLLLKAKVHDLAGDDYRTSVLESIKEWIQTVPLQFLNALLSYLGDSVSYGTTSSGLTSPLACCPSPSFSRVVTPSEGIVRWKLRLEYFA
YETLQDLRIAKLFEIIVDYPESSPAIEDLKQCLEYTGQHSKLVESFISSLKYRLLTAGASTNDILHQYVSTIKALRAIDPAGVFLEAVG
EPIRDYLRGRKDTIKCIVTMLTDGSGGNANGSGNPGDSLLEELMRDEESQENVGFDDDFHTDDKQAWINASRWEPDPVEADPLKGSLSQ
RKVDILGMLVDIIGSKEQLVNEYRVMLAEKLLNKTDYDIDTEIRTVELLKIHFGEASMQRCEIMLNDLIDSKRVNTNIKKASQTGAELR
ENELSVDTLTSTILSTNFWPPIQDEPLELPGPVDKLLSDYANRYHEIKTPRKLLWKKNLGTVKLELQFEDRAMQFTVSPTHAAIIMQFQ
EKKSWTYKDLAEVIGIPIDALNRRVNFWISKGVLRESTGANSNSSVLTLVESITDSGKNEGEELLTGEEEGETSIASVEDQLRKEMTIY
EKFIMGMLTNFGSMALERIHNTLKMFCVADPSYDKSLQQLQSFLSGLVSEEKLEFRDGMYLLKK
>OsApc2 - LOC_Os04g40830
MQLVDDADGALDSWARFCDLSDELFGGAGDLSAGPRLAPVVADLCARGLAELLRDQFIRSLEGIFRSNAVKKFWQQFHPYCNSSAVERI
KFCVQENWPEDILSKALEDICLEKNYQEKCVLALVHSLQSYEDRSPHRKSKALDCSSSLMPRYQLMVSSVLLTTLPLSFPEILNVYFKK
KLEELNIMMAGLDGSDPFDNHDLFERNSTSAWHSEMDIDGQEPGISESRNLVKNIGKVVRDLRYLGFTSMTEDSYSSAIIWLLKSKVHE
LAGDDYRIPVLGCVKKWIQAVPLKFLHAQLTYLGDSLDNESGSSGLKSPLASRPSSFPGIGVPSEALVRWHMRLEYFAYETLQDLRIGK
LFEIIVDYPESSPAIEDLKQCLEYTGQHSKLVDSFISSLRYRLLTAGASTNDILHQYVSTIKALRTIDPTGVFLEAVGEPIRDYLRGRK
DTIKCIVTMLTDGSGGNANGTGNAGDNLLEELNRDAENQENVDYDDHTNIDEKQAWLNAESWEPDPVEADPLKGSRNRRKIDILGLIVS
IIGSKDQLVNEYRVMLAEKLLNKSDFDIDSDIRTLELLKIHFGESSMQKCEIMLNDLIDSKRTNSNIKTSLSKTSQTVGTVQEETELSH
EVLDATIISSNFWPPIQTEDLTVPASVDQLLSDYAKRFHQIKTPRKLLWKKNLGTVKLELQFEDRSMQFTVAPVHAAIIMQFQEKPSWT
SKTLATAIGVPVDSLNRKISFWTSKGVLTESVGPDADDPTFTVVDSTSDFNKNSTVNQLSERFQITEEEGESSIASVEEQLRKEMTVYE
KFIIGMLTNFGSMTLDRIHNTLKMFCIAEPSYDKSLQQLQSFLSGLVSDEKLEMRDGSYLLRK
>PtApc2 - Pt832637
MEESTLSLVSNLEILDTLSADSVQEIVGSYGSFCSATLSLLHGGDASDLFSHVQILCKHGLLSLVRDFFLKSLEEAFERNLASKFWRHF
DCYSNVGANYEIELQQVLCIALEEISLEKQYQEKCLLLLVRALLLEGKTDSDVEREYLFSKYQLMVSSVLMASLPRHFPELLHWYFKGR
LEELSTIMDGEFNGGDDDASQDKDDMDLDEMGKMLHRNGAMDIDESCLQGKFTENNNLVKNIGKVVRDLRSLGFTSMTEDAYASAIFLL
LKAKVHDLAGDDYRASVLGSINEWIKDVPLQFLHALLAYLGETTSYYSPSPGHRSPLASHPSACYPAINAPSEGLVRWHLRLEYFAYET
LQDLRISKLFEIIVDYPDSSPAIEDLKQCLDYTGQHSKLVESFISALRYRLLTAGASTNDILHQYVSTIKALRTIDPAGVFLEAVGEPI
KDYLRGRKDTIKCIVTMLTDGTGGNPNGSGITGDSLLEELNRDEESQENVGADDDFNTDDKQAWVNAASWVPDPVEADPLKGSRNQRKV
DILGMIVGIIGSKDQLVNEYRVMLAEKLLNKSDYDIDSEIRTLELLKIHFGESSMQRCEIMLNDLIDSKRTNHNIKATIKSAQTGSEPA
ETGASMDILNATILSSNFWPPIQDEALNVPEPVNQLLTDYAKRFHEIKTPRKLLWKKNLGTVKLELQFEDRTLQLSVAPIHAAIIMQFQ
DQTSWTSNRLATVIGVPVDVLNRRINFWISKGILTESLGKDLNDHVFTLVEGIVDAGKNSGNTGSCEELLGGDEEGERSVASVEDQIRK
EMTIYEKFIMGMLTNFGSMALDRIHNTLKMFCVADPPYDKSLQQLQSFLSGLVSEEKLELRDGMYFLKK
>AtApc4 - At4g21530
MSEMASDEEENIIPFQLQFDKPIPFQIKIAEWNPEKDLLAMVTEDSKILLHRFNWQRLWTISPGKPVTSLCWRPDGKAIAVGLEDGTIS
LHDVENGKLLRNLKPHDVAVVCLNWEEDGQSNTDESGNFSVYEDRTSRFFPPAPRPPKMPGLVAGDSSFMDDGEDSLAELSNTSFRKFN
ILCTGDRDGNICFSIFGIFQIGKINIHELSLPVPHLDEHASCKLFNASIYKVALSKDLCRLVVMCTGELKDCDIKPREEKINVQDLPGL
HCLAMDTSIFWKRKYELHQVAQQASNIEDLTEVIRASLSVMNKQWADAMKTFHEKFHSLSTLIIDNGLESSPQEEFLSLLGGARISPAL
NQFLVNSLGEVGVKRVLKSVCGTGKELQQVVLDHLQPAAEIIGFRIGELRGLSRWRARYQGIGLDEMLLNEATENTGLLLVQVQRFMMV
LSSVVQQFSNFFNWLVRSIKYLMQEPNDQLLSYNSELLVVFLKFLYDQDPVKDLLELSEAGDDIEIDLKTIGRVKELLQFGGFSECDFL
QRTLAKEFQHMESSFKMAFQMPFTTISRKISCMKLLPLCPLQLSTTQTPTTIPMSLSFYKNELSDDTPCQSGYTDYISFQVPDETFPEI
SNCIGIAKGYKQNSNNEKNGYTSLEAVLLSVPNGYTCVDLSLYKDKELVLLLNKTNTDSEGSGEACMMVVQTGDLAFISISGSSSLNQW
ELEDLKGSIVNLEMENEKVRKVPHSVIAPLAVSASRGVACVFAERRRALVYILEEDEDEEISDEK

>OsApc4 - LOC_Os02g54490
MAEWNPEKDLLAMVTDDSKVVLHRFNWQRLWTISPGKCITSICWSPDGKIVALGTEDGLVLLHDVENGKMLRRIKSHDVAIVCLNWAED
ELLSRTDKDGLLSYEDRTARFFPPAPVIPRIGGLSSGDTGLSDENEESIQEFSSASCQRFNILCSGDKGGCICFSIFGIFPVGKININE
VPIHFQSSGNKTSYRLQDASISKVCLSGNLHQLVLLCPGKLIDIDNLSHSNHISTGLHCLHLDTSIFFNRKNELHQISQQASSIQDLVE
VVRSSLSMMAKQWSSAMNLFNEKFSALPSLIAAHGMESSSEDEFMSLLFGTRTSPALHQFLVSSLGEAALKRIAKAVDSAGRELRVVVS
EHLQPAVEIISFRLAELRGLARWRSRFQNVGLDEKLIYGVTEKIGMLVVQVERFSRVAATVLYLVPFFSACFLAMFSILNLCFNLGLLP
EIPLPVSEYDILVHVVSAEFQNFFSWVLKCVKILLSEPTDQVPAANSELVVLFLKFLLDKDPIKQLLDANQRFECDLDTVRHLEQLVVL
GGFTDTHFLEKTLMKQFNELDESLEEAFSMPFTTISSQIHCQELLPLYPITSSVDLSSTCILTSVSFYKDEDSQNSGSSYSLTDYICFK
IPDGSLNLKNCIGVIKDFSNSSASGPSSSGFLLHIPDEYECVDVSLYKDSQIVVLLSERSYSDGPGSSYIVMLQMENFSFVPLSRMFPS
NIYSVQELSAQELQLDTDYGKKVRSIPHAVSTPLAVSASRGVACVFSSRRHALVYILDEDEDEDEDESSDME
>PtApc4 - Pt817758
METDETDRVLPFQLQFDKPVASQVKIAEWNPEKDLLAMVTEDSKILLHRFNWQRLWTISPGRNITSLCWRPDGKAIAVGLEDGTIYLHD
VENGKLLRSLKSHTVAVVCLNWEEEGQLIRDDSKNSSSYEDRTSRFFPPAPRVPRMPGVVSGDTGFMDDSEDSYRELSNSSYQRFNILC
SGDKDGSICFSIFGIFPIGKIVSLSKDLCRLIVMCSGELNENTESRESQMVKQGMHSLVLDTSIFWKRKSELHQLAQQASNIEDLTEVI
RASLSVMCKQWSDAMHTFHEKFDSLSTLIIDHALDSTPQEEFLSLLGGARTSSAVHQFLVNSLGEVGVKRVLKVICGTAKELQRIVLDH
LQPAAEIIGFRMGELRGLSRWRARYHGIGLDEMLINNATEKSGMILVQIERFMRVLSSVEQQFSNFFNWLLKCIKLLMQEPSDQLLPYN
SELVVIFLKFLYDQDPVKQLLEVDHDIEVDL
>AtApc10 - At2g18290
MATESSESEEEGKISGGNYKLIIDDDLREMGKNAAWSVSSCKPGNGVTTLRDDNLETYWQSDGLQPHLINIQFQKKVKLQLVVLYVDFK
LDESYTPSKISIRAGDGFHNLKEIKSVELVKPTGWVCLSLSGTDPRETFVNTFMLQIAILSNHLNGRDTHIRQIKVYGPRPNPIPHQPF
QFTSMEFLTYSTLR
>OsApc10 - LOC_Os05g50360
MESDGEEEAAATPGAGGAPAAGRLKGCPELMVDDDMREMAKTAAWSVSSCKPGNGVASLRDDNLDTYWQSDGAQPHLVNIQFQKKVQLQ
LVVVYVDFKLDESYTPSKISVRAGDGFHNLKEIKTVELSKPVGWVHISLSGADPRETFIHTFMLQISVLSNHLNGRDTHIRQIKIYGPR
PNHVPHQPFHFTSREFVTYSTVR
>PtApc10 - Pt796785
MATESSEGEEEGKITGGNKHLVIEDDLREMGKKAAWSVSSCKPGNGVSSLRDDNLDTYWQSDGAQPHLVNIQFQKKVKLQLVVLYVDFK
LDESYTPSKISIRAGDGFHNLKEIKTMELVKPTGWVYLSLSGNDPRETFVNTFMLQIAVLSNHLNGRDTHVRQIKVYGPRPNPFPHQPF
QFTSREFIMYSSVR
>AtApc11 - At3g05870
MKVKILRWHAVASWTWDAQDETCGICRMAFDGCCPDCKLPGDDCPLIWGACNHAFHLHCILKWVNSQTSQAHCPMCRREWQFKE
>OsApc11_1 - LOC_Os03g19059
MKVKILQWHSVASWTWDAQDETCGICRMAFDGCCPDCKFPGDDCPLIWGSCNHAFHLHCILKWVNSQTSTPLCPMCRREWQFKG
>OsApc11_2 - LOC_Os07g22840
MKVKILQWHGVASWTWNAQDETCGICRMAFDGCCPDCKFPGDDCPLIWGSCNHAFHLHCILKWVNSQTSTPLCPMCRREWQFKG
>PtApc11 - Pt292476
MKVKLLRWHAVASWTWDAQDETCGICRMAFDGCCPDCKLPGDDCPLIWGACNHAFHLHCILKWVNSQTSQAHCPMCRREWQFKE
>AtCDC26 - TC308166
MLRRKPTKIQLKIEDREELEQSRKSQPSTTTTTAPSSSSAASSLHHLIDPKHKNPSSKSDRIGLS
>OsCDC26 - TC356501
MLRRKPTRIELRSSDRDELEDHLRAAAAAAASTPTASSTPTTTPPPSNSNPLLHLLHPPPGAAPSKSHRIGLPTNPNPNPKP
>PtCDC26 - TC118342
MLRRKPTKIQVKIEDKEELEESRKPTITTTTTSSSTTATSTSSLLHLLDHSKPNPSSKSNRIGLSP
>AtApc13 - At1g73177
MAEVSLGMLIDIVDEEWMRDTLPDDDLPLPPVLAVKTDDTEETNQETQQADAETWRDLALDTQ
>OsApc13 - TC311476
MGGVEQEQLLSLGVLIDIVDEQWMRDTLPADDVPVPPAMAVKTEEAEDPAPANQESQPAQGDVWRDFTLENL
>PtApc13_1 - Pt647861
MAELNMGILIDIVDEEWMRDTLPDDDLALPPVMVVRTDDTEDSNQETQHVDTDAWHDLALGNQ
>PtApc13_2 - Pt660762
MAELNMGILIDIVDEEWMRDTLPDDDLALPPVMVVRTDDAEDSNQDTQHVDADAWHDLALGNQ
