# Supplementary material for: Genomic evolution and complexity of the Anaphase-promoting Complex (APC) in land plants
Source: BMC Plant Biol. 2010 Nov 18;10:254. doi: 10.1186/1471-2229-10-254 (PMC3095333; doi:10.1186/1471-2229-10-254)

Additional file 2: **Confirmation of functionality of Poplar *PtCDC27_2***. A, sequencing of genomic DNA corresponding to PtCDC27_2 locus. Red box shows the actual triplet. B, Real-time PCR detection of *PtCDC27*_2 mRNA, as well of *PtCDC27*_1, *PtCDC20*_1, *PtAPC10, PtCCS52A1_1* and *PtCCS52B*

A

PtCDC27_2

TTGAAGGAAGACATGAAGTTAAGTTACCTGGCTCAAGAACTGATATCAACTGATCGTTTAGCTCCTCAATCTTGXXXGTGTGCCATGGGAAATTGCTATA

Genomic sequence PtCDC27_2

TTGAAGGAAGAAATGAAGTTAAGTTACCTGGCTCAGGAACTGATATCAACTGATCGTTTAGCTCCTCAATCTTGGTAGTGTGCCATGGGAAATTGCTATA

B

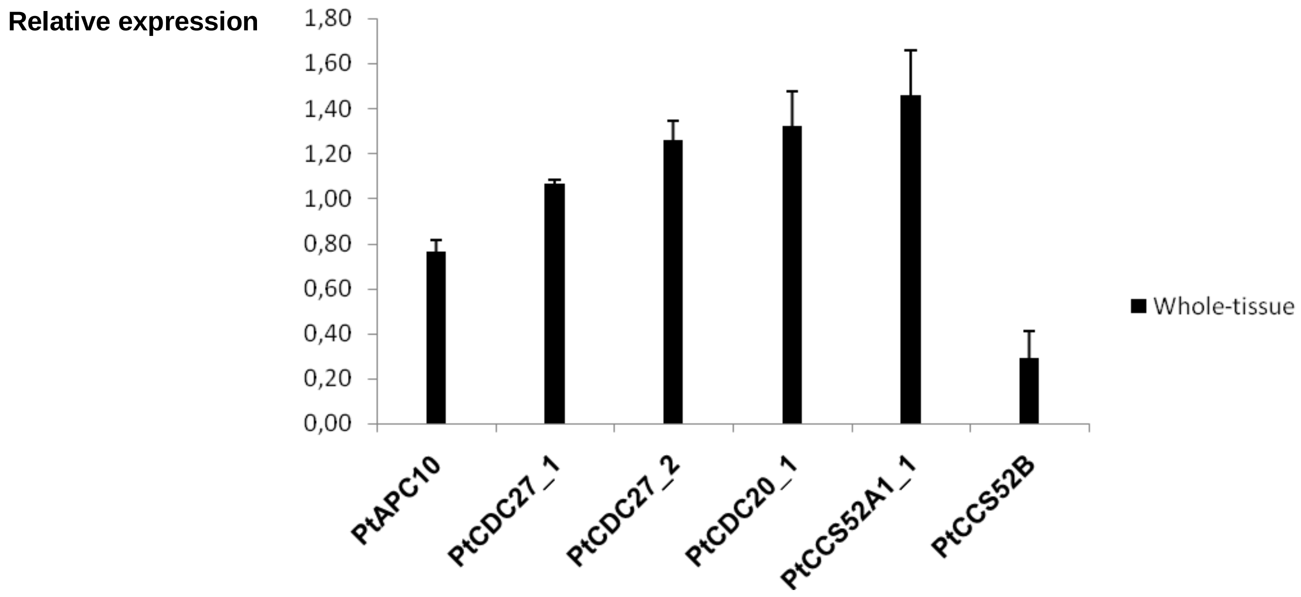

Supplement: Additional file 2 — Confirmation of functionality of Poplar PtCDC27_2. A, sequencing of genomic DNA corresponding to PtCDC27_2 locus. Red box shows the actual triplet. B, Real-time PCR detection of PtCDC27_2 mRNA, as well of PtCDC27_1, PtCDC20_1, PtAPC10, PtCCS52A1_1 and PtCCS52B. [file 1471-2229-10-254-S2.PDF]
