# Supplementary material for: Genomic evolution and complexity of the Anaphase-promoting Complex (APC) in land plants
Source: BMC Plant Biol. 2010 Nov 18;10:254. doi: 10.1186/1471-2229-10-254 (PMC3095333; doi:10.1186/1471-2229-10-254)
Supplement: Additional file 3 — Gene Index (partial) sequence of OsAPC1. An EST was recovered from the Gene Index Project, showing part of (upper). The coding region was underlined and stop codon was marked in red. This sequence was translated in silico (lower). [file 1471-2229-10-254-S3.PDF]

Additional file 3: **Gene Index (partial) sequence of *OsAPC1***. An EST was recovered from the Gene Index Project, showing part of (upper). The coding region was underlined and stop codon was marked in red. This sequence was translated *in silico* (lower).

Gene Index

>TC286185

TGCTCAGCAAGACCTGTGGCAATACAGACACCCAATAATCCTAGTGTGTCTGATCAGGATCTACAACAGCAACAACTATGGAACTTTGC
ACAAAGAACAACAGCTTTACCTTTTGGGCGTGGGGCTTTTACTTTAGCTACAACTTACACACTGTTAACAGAGGCTCTGGTTTTCCCAA
AGCTTGTTTTGGCTGGTCGGTTGCCTGCACAACAGAATGCAACAGTTAATCTTGACCTAAGTACTAGAAGCGTCTCAGAATTCAAATCT
TGGGCTGAGTTTCACAATGGTGTCGCTGCTGGGCTCAGGCTTGCTCCTTTTCAGGAGAAAATGTTAAGAACCTGGATACAATATAATAG
ACCTTCTGAACCAAATTTTACTCATGCTGGTCTGCTTCTTGCATTTGGTTTGCATGAACATCTACGAGTTTTAACTATGACCGACGCTT
ACCGATATCTCTCCCAGGAGCATGATATAACTAGACTTGGTTTGCTACTTGGTTTGGCTGCATCTAATAGGGGGACAATGCACCCGGCA
ATTTCAAAGATGCTCTATTTTCATGTCCCTTCCCGGCATCCATCTTCTACGCCAGAGTTGGAATTACCTACTCTTCTGCAGTCAGCAGC
AGTGATGGGAATTGGACTTCTCTATGAAGGGTCAGCACATGCATTAACCATGAAAATTCTTCTGGGTGAGATTGGCCGAAGAAGTGGTG
GTGATAATGTGCTAGAAAGAGAAGGCTATGCTGTTGCTGCAGGCTCTGCATTAGGACTTGTTGCATTAGGTCGTGGAAGCAATGCTTTC
GGATTCATGGACACTTTCCTGGACCGGCTTTTTGAATACATTGGCAGTAAAGAAGTCTACCATGAAAAACACTTAAATGCAGCAATAGC
TGCTGATGAGCAAAGTGGCAACACCGGACAGATGATGGAAGGAGCACAAATAAATGTTGATGTTACTGCACCTGGAGCAATAATTGCTC
TAGCCTTGATATTTCTGAAAGCAGAGTCAGAAGAAATTGCAGCTAGACTCAGTGTTCCCAATAGTCACTTTGATCTGCAGTATGTGAGA
CCTGATTTTGTAATGCTTCGCATTGTAGCACGGAATTTAATATTGTGGAACAGAATACAACCTACCAAAGATTGGGTTGAGTCTCAAGT
TCCTTCATTTGTCAACTTTGGTGTTTCCAATACGAGTCAAGAGGCTATGGACAGTGATGAATTGGATAGCGAAGCTCTGTTTCAAGCTT
ATGTTAATATTGTCACTGGAGCATGCATTGCACTTGGTCTCAAATATGCTGGCAGTAGAAATAGTGATGCCCAAGAACTACTCTACGCT
TATGCCGTCCATTTTCTCAATGAGATCAAACACATATCTATTCAGACTGCAAGCATATTGCCAAAGGGATTGTTGCAGCATGTTGATCG
TGGAACTCTTGAGCTCTGCTTGCATCTTATTGTTCTCTCTCTATCACTGGTAATGGCAGGATCTGGACATTTACAAACTTTCCGCTTAC
TGCGTTATCTGAGGGGAAGAAGTTCTGCAGAAGGACAAGTAAATTATGGACTGCAGATGGCTGTAAGCTTGGCTATAGGATTCTTATTC
CTTGGAGGTGGTACACACACATTCTCAACTTCAAATAGTGCAGTTGCCGCATTACTCATCACCCTTTACCCACGTTTGCCTACTGGACC
AAATGATAATCGCTGTCATCTCCAGGCATTCAGGCATCTGTATGTAATAGCTACAGAACCTCGATGGATTCAGACAGTAGATGTTGACA
CAGGACTTCCGGTGTATTGTCCTCTTGAAGTGACAGTTGCTGAAACGGAGTATTATGACGAGACTAATTACTGTGAAGTGACACCTTGT
CTTCTGCCAGAGCGCTCAGTGCTGAAGAACATTCGAGTTTGTGGACCCAGATATTGGTCTCAGGTGATTACACTTACACCTGAAGATAA
GCCATGGTGGAAATCTGGAGACAGGACTGACCCATTCAATGGTGGAGTACTCTATATAAAACGGAAAGTTGGATCTTGTTCCTACTCAG
ATGATCCAATTGGATGCCAATCTTTGCTTTCTCGAGCAATGCATGAGGTTTGTGATACACCATCCACGAGCTGCAGTAATCAGGCAAAC
AGTGCTACTCGTAGTTCACTTAGAGTCGATCAGTTAGTAAGCACATTTTCTGCTAATCCAAGCTTGATAGCTTTTGCAAAACTGTGTTG
TCAGTCATGGAAGGACAGGCGCAATGGCAGTTTTGAAGAGTTCTGCTCTCAAATACTTTATGAATGTATGAGCAAGGATAGGCCAGCAC
TATTGCAGGTGTATATAAGTTTTTACACAATAATTGAGACAATGTGGGAGCATCTAAAGATTGGACATTTTCCTTTCTCTGATTCTCTT
TTCCTTTCCAGCTTGAAGGTTGCATCAGCCTATAATGAAGCACTGATTGATGGTAGAATTACAACTGGAGGCATTATTCAGTCCACATT
TTTGGAATCACTTATGAAGCGCATAGAGTACATCTTCGCAGAATTGCCGAACCTGCATGACAGCTTCATCAATTACCTGAACAAAGGCA
AATGGCCTGATGCACAAAACGAAGCAGTGCTCCTTTCTTGGTATCTCCAGTGGTACAGCATCCCACCTCCCCATATTGTGTCATCCGCA
ATTGAAAAGGTGAAGCCCAGAACTCGGACCAGCTTGTCCATGCTCCCGCTTCTTCGGCTCCTGCTGCCAACCACACATCTCGTGGGGCT
CATGGAGATTGAGAAGCTTCACATGACTCATGGTCACGAGGGCTTGACCTTGCAC**TGA**AAATGCAAGAAACGTTTAGCAACGAGTGAGC
CGATTGTGACTGGACTGAATAGTATCAGGGCTCATTGTTCTTATCTACTAGTAAGGATCATACCTAAGTGCTGGAATGCCAAGGCTTTG
TGTGGAGGCCTCAATCATGTGATCCTACCTTGCAATGCTCATATTGCCATCTGATATTGTACCCTATGATCCTGTTGCATTTAGATGTA
AGTGTCCATTTTGGCTTTAGAATAGGAATGACCGAATGAGTGAGCCTAGTTTGAGCCTTTGATAACTGCTACATTTGGGCTCAATGCAA
CATGGGTTCCCAGTGTATGCCGACCGGTTTCAATAAACTGGTGGTTACGAATTGAGCTATAATAGAGCACATTTTGTTTCTCAGGCTAA
AAANAA

>OsAPC1

CSARPVAIQTPNNPSVSDQDLQQQQLWNFAQRTTALPFGRGAFTLATTYTLLTEALVFPKLVLAGRLPAQQNATVNLDLSTRSVSEFKS
WAEFHNGVAAGLRLAPFQEKMLRTWIQYNRPSEPNFTHAGLLLAFGLHEHLRVLTMTDAYRYLSQEHDITRLGLLLGLAASNRGTMHPA
ISKMLYFHVPSRHPSSTPELELPTLLQSAAVMGIGLLYEGSAHALTMKILLGEIGRRSGGDNVLEREGYAVAAGSALGLVALGRGSNAF
GFMDTFLDRLFEYIGSKEVYHEKHLNAAIAADEQSGNTGQMMEGAQINVDVTAPGAIIALALIFLKAESEEIAARLSVPNSHFDLQYVR
PDFVMLRIVARNLILWNRIQPTKDWVESQVPSFVNFGVSNTSQEAMDSDELDSEALFQAYVNIVTGACIALGLKYAGSRNSDAQELLYA
YAVHFLNEIKHISIQTASILPKGLLQHVDRGTLELCLHLIVLSLSLVMAGSGHLQTFRLLRYLRGRSSAEGQVNYGLQMAVSLAIGFLF
LGGGTHTFSTSNSAVAALLITLYPRLPTGPNDNRCHLQAFRHLYVIATEPRWIQTVDVDTGLPVYCPLEVTVAETEYYDETNYCEVTPC
LLPERSVLKNIRVCGPRYWSQVITLTPEDKPWWKSGDRTDPFNGGVLYIKRKVGSCSYSDDPIGCQSLLSRAMHEVCDTPSTSCSNQAN
SATRSSLRVDQLVSTFSANPSLIAFAKLCCQSWKDRRNGSFEEFCSQILYECMSKDRPALLQVYISFYTIIETMWEHLKIGHFPFSDSL
FLSSLKVASAYNEALIDGRITTGGIIQSTFLESLMKRIEYIFAELPNLHDSFINYLNKGKWPDAQNEAVLLSWYLQWYSIPPPHIVSSA
IEKVKPRTRTSLSMLPLLRLLLPTTHLVGLMEIEKLHMTHGHEGLTLH*
