# Supplementary material for: Genomic evolution and complexity of the Anaphase-promoting Complex (APC) in land plants
Source: BMC Plant Biol. 2010 Nov 18;10:254. doi: 10.1186/1471-2229-10-254 (PMC3095333; doi:10.1186/1471-2229-10-254)
Supplement: Additional file 4 — Fragments from 5'region of PtAPC5 and fragments from the 3'regions of genes PtAPC4 and PtAPC5 were found in the EST database. The additional sequence of OsAPC4 (not present in Arabidopsis nor poplar), product of incorporation of an intron, is boxed in yellow. Differences in 5'region of OsAPC5 are also highlighted in yellow. [file 1471-2229-10-254-S4.PDF]

Additional file 4: Fragments from 5´region of *PtAPC5* and fragments from the 3´regions of genes *PtAPC4 and PtAPC5* were found in the EST database. The additional sequence of *OsAPC4* (not present in Arabidopsis nor poplar), product of incorporation of an intron, is boxed in yellow. Differences in 5´region of *OsAPC5* are also highlighted in yellow.

Before EST support:

```
AtAPC4          MSEMASDEEENIIPFQLQFDKPIPFQIKIAEWNPEKDLLAMVTEDSKILLHRFNWQRLWT 60
PtAPC4          ---METDETDRVLPFQLQFDKPVASQVKIAEWNPEKDLLAMVTEDSKILLHRFNWQRLWT 57
OsAPC4          ----------------------------MAEWNPEKDLLAMVTDDSKVVLHRFNWQRLWT 32
                                            :**************:***::***********

AtAPC4          ISPGKPVTSLCWRPDGKAIAVGLEDGTISLHDVENGKLLRNLKPHDVAVVCLNWEEDGQS 120
PtAPC4          ISPGRNITSLCWRPDGKAIAVGLEDGTIYLHDVENGKLLRSLKSHTVAVVCLNWEEEGQL 117
OsAPC4          ISPGKCITSICWSPDGKIVALGTEDGLVLLHDVENGKMLRRIKSHDVAIVCLNWAEDELL 92
                ****: :**:** **** :*:* *** : ********:** :*.* **:***** *:

AtAPC4          NTDESGNFSVYEDRTSRFFPPAPRPPKMPGLVAGDSSFMDDGEDSLAELSNTSFRKFNIL 180
PtAPC4          IRDDSKNSSSYEDRTSRFFPPAPRVPRMPGVVSGDTGFMDDSEDSYRELSNSSYQRFNIL 177
OsAPC4          SRTDKDGLLSYEDRTARFFPPAPVIPRIGGLSSGDTGLSDENEESIQEFSSASCQRFNIL 152
                   :. .   *****:*******  *:: *: :**:.: *:.*:*  *:*.:* ::****

AtAPC4          CTGDRDGNICFSIFGIFQIGKINIHELSLPVPHLDEHASCKLFNASIYKVALSKDLCRLV 240
PtAPC4          CSGDKDGSICFSIFGIFPIGKI---------------------------VSLSKDLCRLI 210
OsAPC4          CSGDKGGCICFSIFGIFPVGKININEVPIHFQSSGNKTSYRLQDASISKVCLSGNLHQLV 212
                *:**:.* ********* :***                           *.** :* :*:

AtAPC4          VMCTGELKDCDIKPREEKINVQDLPGLHCLAMDTSIFWKRKYELHQVAQQASNIEDLTEV 300
PtAPC4          VMCSGELNEN-TESRESQMVKQ---GMHSLVLDTSIFWKRKSELHQLAQQASNIEDLTEV 266
OsAPC4          LLCPGKLIDIDNLSHSNHISTG----LHCLHLDTSIFFNRKNELHQISQQASSIQDLVEV 268
                ::*.*:* :    .:..::       :*.* :*****::** ****::****.*:**.**

AtAPC4          IRASLSVMNKQWADAMKTFHEKFHSLSTLIIDNGLESSPQEEFLSLLGGARISPALNQFL 360
PtAPC4          IRASLSVMCKQWSDAMHTFHEKFDSLSTLIIDHALDSTPQEEFLSLLGGARTSSAVHQFL 326
OsAPC4          VRSSLSMMAKQWSSAMNLFNEKFSALPSLIAAHGMESSSEDEFMSLLFGTRTSPALHQFL 328
                :*:***:* ***:.**: *:*** :*.:**  :.::*:.::**:*** *:* *.*::***

AtAPC4          VNSLGEVGVKRVLKSVCGTGKELQQVVLDHLQPAAEIIGFRIGELRGLSRWRARYQGIGL 420
PtAPC4          VNSLGEVGVKRVLKVICGTAKELQRIVLDHLQPAAEIIGFRMGELRGLSRWRARYHGIGL 386
OsAPC4          VSSLGEAALKRIAKAVDSAGRELRVVVSEHLQPAVEIISFRLAELRGLARWRSRFQNVGL 388
                *.****..:**: * : .:.:**: :* :*****.***.**:.*****:***:*::.:**

AtAPC4          DEMLLNEATENTGLLLVQVQRFMMVLSS-------------------------------- 448
PtAPC4          DEMLINNATEKSGMILVQIERFMRVLSS-------------------------------- 414
OsAPC4          DEKLIYGVTEKIGMLVVQVERFSRVAATVLYLVPFFSACFLAMFSILNLCFNLGLLPEIP 448
                ** *:  .**: *:::**::**  * ::

AtAPC4          ------------VVQQFSNFFNWLVRSIKYLMQEPNDQLLSYNSELLVVFLKFLYDQDPV 496
PtAPC4          ------------VEQQFSNFFNWLLKCIKLLMQEPSDQLLPYNSELVVIFLKFLYDQDPV 462
OsAPC4          LPVSEYDILVHVVSAEFQNFFSWVLKCVKILLSEPTDQVPAANSELVVLFLKFLLDKDPI 508
                            *  :*.***.*:::.:* *:.**.**: . ****:*:***** *:**:

AtAPC4          KDLLELSEAGDDIEIDLKTIGRVKELLQFGGFSECDFLQRTLAKEFQHMESSFKMASQMP 556
PtAPC4          KQLLEV---DHDIEVDL------------------------------------------- 476
OsAPC4          KQLLDAN---QRFECDLDTVRHLEQLVVLGGFTDTHFLEKTLMKQFNELDESLEEAFSMP 565
                *:**:     . :* **

AtAPC4          FTTISRKISCMKLLPLCPLQLSTTQTPTTIPMSLSFYKNELSDDTPCQSGYTDYISFQVP 616
PtAPC4          ------------------------------------------------------------
OsAPC4          FTTISSQIHCQELLPLYPITSSVDLSSTCILTSVSFYKDEDSQNSGSSYSLTDYICFKIP 625


AtAPC4          DETFPEISNCIGIAKGYKQNSNNEKNGYTSLEAVLLSVPNGYTCVDLSLYKDKELVLLLN 676
PtAPC4          ------------------------------------------------------------
OsAPC4          DGSLN-LKNCIGVIKDFSNSSASG----PSSSGFLLHIPDEYECVDVSLYKDSQIVVLLS 680


AtAPC4          KTNTDSEGSGEACMMVVQTGDLAFISISGSSSLNQWELEDLKGSIVNLEMEN-EKVRKVP 735
PtAPC4          ------------------------------------------------------------
OsAPC4          ER-SYSDGPGSSYIVMLQMENFSFVPLSRMFPSNIYSVQELSAQELQLDTDYGKKVRSIP 739


AtAPC4          HSVIAPLAVSASRGVACVFAERRRALVYILEEDEDEEISDEK--- 777
PtAPC4          ---------------------------------------------
OsAPC4          HAVSTPLAVSASRGVACVFSSRRHALVYILDEDEDEDEDESSDME 784
```

(Highlighted in yellow in the alignment: OsAPC4 residues "VLYLVPFFSACFLAMFSILNLCFNLGLLPEIP" (line ending 448) and "LPVSEYDILVHVVSAE" (line ending 508).)

After EST support – 3´region.

```
AtAPC4          MSEMASDEEENIIPFQLQFDKPIPFQIKIAEWNPEKDLLAMVTEDSKILLHRFNWQRLWT 60
PtAPC4          ---METDETDRVLPFQLQFDKPVASQVKIAEWNPEKDLLAMVTEDSKILLHRFNWQRLWT 57
OsAPC4          ----------------------------MAEWNPEKDLLAMVTDDSKVVLHRFNWQRLWT 32
                                            :*************:***::***********

AtAPC4          ISPGKPVTSLCWRPDGKAIAVGLEDGTISLHDVENGKLLRNLKPHDVAVVCLNWEEDGQS 120
PtAPC4          ISPGRNITSLCWRPDGKAIAVGLEDGTIYLHDVENGKLLRSLKSHTVAVVCLNWEEEGQL 117
OsAPC4          ISPGKCITSICWSPDGKIVALGTEDGLVLLHDVENGKMLRRIKSHDVAIVCLNWAEDELL 92
                ****: :**:** **** :*:* *** : ********:** :*.* **:***** *:

AtAPC4          NTDESGNFSVYEDRTSRFFPPAPRPPKMPGLVAGDSSFMDDGEDSLAELSNTSFRKFNIL 180
PtAPC4          IRDDSKNSSSYEDRTSRFFPPAPRVPRMPGVVSGDTGFMDDSEDSYRELSNSSYQRFNIL 177
OsAPC4          SRTDKDGLLSYEDRTARFFPPAPVIPRIGGLSSGDTGLSDENEESIQEFSSASCQRFNIL 152
                   :. .    *****:*******  *:: *: :**:.: *:.*:*  *:*.:* ::****

AtAPC4          CTGDRDGNICFSIFGIFQIGKINIHELSLPVPHLDEHASCKLFNASIYKVALSKDLCRLV 240
PtAPC4          CSGDKDGSICFSIFGIFPIGKINIHKFSVPTPFIDKQTPRQILNSSIYKVSLSKDLCRLI 237
OsAPC4          CSGDKGGCICFSIFGIFPVGKININEVPIHFQSSGNKTSYRLQDASISKVCLSGNLHQLV 212
                *:**:.* ********* :*****::..:      .:::. :: ::** **.** :* :*:

AtAPC4          VMCTGELKDCDIKPREEKINVQDLPGLHCLAMDTSIFWKRKYELHQVAQQASNIEDLTEV 300
PtAPC4          VMCSGELNEN-TESRESQMVKQ---GMHSLVLDTSIFWKRKSELHQLAQQASNIEDLTEV 293
OsAPC4          LLCPGKLIDIDNLSHSNHISTG----LHCLHLDTSIFFNRKNELHQISQQASSIQDLVEV 268
                ::*.*:* :     .:..::        :*.* :*****::** ****::****.*:**.**

AtAPC4          IRASLSVMNKQWADAMKTFHEKFHSLSTLIIDNGLESSPQEEFLSLLGGARISPALNQFL 360
PtAPC4          IRASLSVMCKQWSDAMHTFHEKFDSLSTLIIDHALDSTPQEEFLSLLGGARTSSAVHQFL 353
OsAPC4          VRSSLSMMAKQWSSAMNLFNEKFSALPSLIAAHGMESSSEDEFMSLLFGTRTSPALHQFL 328
                :*:***:* ***:.**: *:*** :*.:**  .::*:.::**:*** *:* *.*::***

AtAPC4          VNSLGEVGVKRVLKSVCGTGKELQQVVLDHLQPAAEIIGFRIGELRGLSRWRARYQGIGL 420
PtAPC4          VNSLGEVGVKRVLKVICGTAKELQRIVLDHLQPAAEIIGFRMGELRGLSRWRARYHGIGL 413
OsAPC4          VSSLGEAALKRIAKAVDSAGRELRVVVSEHLQPAVEIISFRLAELRGLARWRSRFQNVGL 388
                *.****..:**: * : .:.:**: :* :*****.***.**:.*****:***:*::.:**

AtAPC4          DEMLLNEATENTGLLLVQVQRFMMVLSS-------------------------------- 448
PtAPC4          DEMLINNATEKSGMILVQIERFMRVLSS-------------------------------- 441
OsAPC4          DEKLIYGVTEKIGMLVVQVERFSRVAATVLYLVPFFSACFLAMFSILNLCFNLGLLPEIP 448
                ** *:  .**: *:::**::**  * ::

AtAPC4          ------------VVQQFSNFFNWLVRSIKYLMQEPNDQLLSYNSELLVVFLKFLYDQDPV 496
PtAPC4          ------------VEQQFSNFFNWLLKCIKLLMQEPSDQLLPYNSELVVIFLKFLYDQDPV 489
OsAPC4          LPVSEYDILVHVVSAEFQNFFSWVLKCVKILLSEPTDQVPAANSELVVLFLKFLLDKDPI 508
                            *  :*.***.*:::.:* *:.**.**: .  ****:*:***** *:**:

AtAPC4          KDLLELSEAGDDIEIDLKTIGRVKELLQFGGFSECDFLQRTLAKEFQHMESSFKMASQMP 556
PtAPC4          KQLLEV---DHDIEVDLQ---RVKELVQFGGFSDCEYLQRTLAKEFQQMEDSFKEAFLMP 543
OsAPC4          KQLLDAN---QRFECDLDTVRHLEQLVVLGGFTDTHFLEKTLMKQFNELDESLEEAFSMP 565
                *:**:      . :* **.   ::::*: :***:: .:*::** *:*:.::.*:: *  **

AtAPC4          FTTISRKISCMKLLPLCPLQLSTTQTPT--TIPMSLSFYKNELSDDTPCQSGYTDYISFQ 614
PtAPC4          FTTISRKMLCEDLLPLFPLPSSSSSASVSMAIPMSISYYSQAVSSNQTCQHSFVDYVCFQ 603
OsAPC4          FTTISSQIHCQELLPLYPITSSVDLSST--CILTSVSFYKDEDSQNSGSSYSLTDYICFK 623
                ***** :: * .**** *:  *   :..   *  *:*:*.:  *.:  .. . .**:.*:

AtAPC4          VPDETFPEISNCIGIAKGYKQNSNNEKNGYTSLEAVLLSVPNGYTCVDLSLYKDKELVLL 674
PtAPC4          VPDEPFSDIANCIGVIRGFTHDLSSSKNGYTSLEAVLLYVPAGYECVDLSLYKDSQIVLL 663
OsAPC4          IPDGSLN-LKNCIGVIKDFSNSSASG----PSSSGFLLHIPDEYECVDVSLYKDSQIVVL 678
                :** .:  : ****: :.:.:.  .      .* ...** :*  * ***:*****.::*:*

AtAPC4          LNKTNTDSEGSGEACMMVVQTGDLAFISISGSSSLNQWELEDLKGSIVNLEMENEK-VRK 733
PtAPC4          LNGASASSESSGDACMMIVQASELPFISISRFTDLNLWNLYQLKDSTVQLQMENE----K 719
OsAPC4          LSER-SYSDGPGSSYIVMLQMENFSFVPLSRMFPSNIYSVQELSAQELQLDTDYGKKVRS 737
                *.   : *:..*.: ::::*  ::.*:.:*     * :.: :*. . ::*: :       .

AtAPC4          VPHSVIAPLAVSASRGVACVFAERRRALVYILEEDEDEEISDEK--- 777
PtAPC4          VPHSVIAPLAVSASRGVACVFAARKRALVYILEEDE-EEVPDTE--- 762
OsAPC4          IPHAVSTPLAVSASRGVACVFSSRRHALVYILDEDEDEDEDESSDME 784
                :**:* :**************: *::******:*** *:  :  : .
```

```
AtAPC5          MAGLTRTAGAFAVTPHKISVCILLQIYAPSAQMSLPFPFSSVAQHNRLGLYLLSLT---K 57
PtAPC5          ---------AFALTPHKVSVCLLLQTYALPAQTTPPFPFSSVSQHNRLGLYLLALTKMLQ 51
OsAPC5          ------------------------------------------------------------


AtAPC5          SCDDIFEPKLEKLINQLREVGEEMDAWLTDHLTNRFSSLASPDDLLNFFNDMRGILGSLD 117
PtAPC5          SYDDILEPKLEELLNQLKEISGSLGHWLIDHLTSRLSSLSAPDDLFSFFTEMRGILGGLD 111
OsAPC5          ------------------------------------------------------------


AtAPC5          SGVVQDDQIILDPNSNLGMFVRRCILAFNLLSFEGVCHLFSSIEDYCKEAHSSFAQFGAP 177
PtAPC5          SVVMEDNQVILDPNSNLGLFLRRCILTFNLLSFEGLCHLLTNIGSYCKEAMSS------- 164
OsAPC5          ------------------------------MTFEGVCHLLANLVEYCNSADTS------- 23
                                              ::***:***::.:  .**:.*  :*

AtAPC5          NNNLESLIQYDQMDMENYAMDKPTEEIEFQKTASGIVPFHLHTPDSLMKATEGLLHNRKE 237
PtAPC5          SNDLETLSEYENMDLENFMFGKVNEEIEARKQASERVPFHLHGPKALSGLVEGIID---- 220
OsAPC5          YDLAEDEDFNSEMEMSNFMDTN-------MHVRDGVFDKYNQGYAPRSHMVD-------- 68
                 :  *      .:*::.*:    :         :  .  .  : :    .     .:

AtAPC5          TSRTSKKDTEATPVARASTSTLEESLVDESLFLRTNLQIQGFLMEQADAIEIHGSSSSFS 297
PtAPC5          SSKHGDKCGETSAYVHPPGNELRDVDPYGEIFLRTNWQVQGYLMEQADAIEGCRHDSSFS 280
OsAPC5          -SSSSLVHAPASLHDFEEANMFKADDNLGPTCLRSRWQLEAYLNQQADILE--KDPSSVP 125
                 *  .      ::        . :.          **:.  *::.:*  :*** :*      **..

AtAPC5          SSSIESFLDQLQKLAPELHR-------------------VHFLRYLNKLHSDDYFAALDN 338
PtAPC5          LNSFELVLRQIKKLAPELIQ-------------------VHFLRYLNSLYHDDYFAALDN 321
OsAPC5          LNSFNATMSQLQKLAPELHRNCEEFLVTMVYFDLYSTCQVQFLQYLNALTHDDYVAALDN 185
                 .*::  : *::****** :                      *:**:*** *  ***.*****

AtAPC5          LLRYFDYSAGTEG-FDLVPPSTGCSMYGRYEIGLLCLGMMHFRFGHPNLALEVLTEAVRV 397
PtAPC5          LHRYFDYSAGAEG-FDSAPSSSGSNSSGRYEIGLIYLGMMHLHFGHPKQALEVLTEAVRF 380
OsAPC5          LHRYFDYSAGMQGLFSRTASPFQDIIVGKYESALLCLGNLHCYFGHPKKALEAFTEAVRV 245
                * ******** :* *. ....       *:** .*: ** :*  ****: ***.:*****.

AtAPC5          SQQLSNDTCLAYTLAAMSNLLSEMGIASTSGVLGSSYSP--VTSTASSLSVQQRVYILLK 455
PtAPC5          SQQQSNESCLAYTLAAICNVLSEFGCSSSAGVLGTSFSP--ITSMDTSLSVGQQLFVLLR 438
OsAPC5          SQMNNDDSCLAYILGAISNLLSKIGMSSTVGTIGSPYSLGNNIGLGTPLSIQQQLLVLLK 305
                **  .:::**** *.*:.*:**::* :*: *.:*:.:*      .  :.**: *:: :**:

AtAPC5          ESLRRADSLKLRRLVASNHLAMAKFELM------------HVQRPLLSFGPKASMRHKTC 503
PtAPC5          ESLKRAESLKLKRLVASNHLALAKFDLLTSYLFQLYFDLQHVQRPLLSFGPKASMKLRTF 498
OsAPC5          RSLKRADTLKLTSLLSFDHLSLAKFDLK------------HVQRPLVSFGPNASTKLRTC 353
                .**:**::***  *:: :**::***:*             ******:****:** : :*

AtAPC5          PVSVCK-------------------EIRLGAHLISDFSSESSTMTID-GSLSSAWLKDLQ 543
PtAPC5          PINVCKVFGAFGDSIYSFSPISLLQELRLCSHLISEFGSESSTMTTD-GVFSTTWLNNLP 557
OsAPC5          PADVCK-------------------NLRLSSRVLTDFGTDGLSASNDNGSFSTSWLRNLS 394
                * .***                   ::** ::::::*.::. : : * * :*::**.:*

AtAPC5          KPWGPPVISPDSGS---RKSSTFFQLCDHLVSIPGSVSQLIGASYLLRATSWELYGSAPM 600
PtAPC5          KSMDSPLLPQENAH---RNNCDAHRFFTQLSSVPKSVLQLLGSSYIMRSTAWEMYGSAPL 614
OsAPC5          AASNSWCSSSKKSGKLLTNDFDNFHFHAQPSPIPASVLQLAGSAYLLRATAWEHYGSAPM 454
                 . ..   . ...     :.   .::  :  .:* ** ** *::*::*:*:** *****:

AtAPC5          ARMNTLVYATLFGDSSSSSDAELAYLKLIQHLALYKGYKDAFAALKVAEEKFLTVSKSKV 660
PtAPC5          ARINSLVYATCFADASSSSDAASVHAKLIQHLAVFRGYKGAFAALKVAEEKFLTVSKSVI 674
OsAPC5          VRMNSLVYATCFADAASSSELSLAYVKLIQHLATFKGYSAAFSALKLAEEKFPLSANSHI 514
                .*:*:***** *.*::***:   .: ******* ::**. **:***:*****   ::* :

AtAPC5          LLLKLQLLHERALHCGNLKLAQRICNELGGLASTAMGVDMELKVEASLREARTLLAAKQY 720
PtAPC5          LLVKLQLLHECALHRGNLKLAQQVCDELGVLASSVSGVDKDLKTEASLRHARTLLAANQF 734
OsAPC5          QLLKMQLLHERALHRGHLKVAQQICDEFAVLSSSVSGVDIELKTEARLRHARTLLAAKQF 574
                 *:*:***** *** *:**:**::*:*:. *:*:. *** :**.** **.*******:*:

AtAPC5          SQAANVAHSLFCTCHKFNLQIEKASVLLLLAEIHKKSGNAVLGLPYALASISFCQSFNLD 780
PtAPC5          SQAAAVAHSLFCMCYKFNMQVQNATVLLLLAEIHKKSGNAVLGLPYALASLSFCQSFNLD 794
OsAPC5          SQAANVANSLFSTCYKYNMQVENASVLLLLAEIQKNSDNAVLGLPYALASQSFCKSFNLD 634
                **** **:***. *:*:*:*:::*:********:*:*.************ ***:*****

AtAPC5          LLKASATLTLAELWLGLGSNHTKRALDLLHGAFPMILGHGGLELRARAYIFEANCYLSDP 840
PtAPC5          LLKASATLTLAELWLSLGSNHAKRALTLIHGALPMILGHGGLELQARAQITEAKCYLSDP 854
OsAPC5          LLEASATLTLTELWLALGSTHAKRALSLVCQSLPMILGHGGLELRARAHIVLAKCYLSDP 694
                **:*******:****.***.*:**** *:  ::***********:*** *  *:******

AtAPC5          SSS------------VSTDSDTVLDSLRQASDELQALEYHELAAEASYLMAMVYDKLGRL 888
PtAPC5          SYS------------GSTLSSPFLDLLRQASDELQVLEYHELAAEAFYLMAHVFDKLGQL 902
OsAPC5          KFSEKPVPLCPFIMAVSEDPSAVLDPLNQAAEDLEVLEYHEMAAEAYYLKAMVYNNLGKL 754
                . *            *  ....** *.**:::*:.*****:**** ** * *:::**:*

AtAPC5          DEREEAASLFKKHIIALENPQDVEQNMA- 916
PtAPC5          ERREEAAASFKEHMMALENPQD------- 924
OsAPC5          DEREEAAASFKEHTLALENPYNEEDSLAC 783
                :.*****.**:*:*****:
```
