# Supplementary material for: Genomic evolution and complexity of the Anaphase-promoting Complex (APC) in land plants
Source: BMC Plant Biol. 2010 Nov 18;10:254. doi: 10.1186/1471-2229-10-254 (PMC3095333; doi:10.1186/1471-2229-10-254)
Supplement: Additional file 5 — Comparison of 5'region of CDC16 and APC11_2 genes between Arabidopsis, poplar and rice. The additional sequences are boxed in yellow. [file 1471-2229-10-254-S5.PDF]

Additional file 5: Comparison of 5´region of *CDC16* and *APC11_2* genes between *Arabidopsis*, poplar and rice. The additional sequences are boxed in yellow.

CDC16

```
PtCDC16         ------------------------------------------------------------
AtCDC16         ------------------------------------------------------------
OsCDC16         MPLSAASINRASYQVLLLLAAAAVSTTGGDGNTAPGNATATATTGGDDTEMYICYLCTGR 60


PtCDC16         ------------------------------------------------------------
AtCDC16         ------------------------------------------------------------
OsCDC16         NPILIRRCPIYWDYCHLNCFDDAPSTAAAADDVAAVPVASPAAPARRVGGVPRETLEDEE 120


PtCDC16         ------------------------------------------------------------
AtCDC16         ------------------------------------------------------------
OsCDC16         CYVMKLYENGSYVIVTTLGCSQTASCLLSCGGGDLAADGEEALAAAHPAGAVGVSPPWRM 180


PtCDC16         --------------------------------------MREEQIEKLRGVVRDCVSKHLY 22
AtCDC16         --------------------------------------MREEEIEKIRGVVRDCVSKHLY 22
OsCDC16         WDTKFGFPPAAPPTTAAAAQKNPKRRREAEAEGEVAAEMREEAVERLRGVVRDSVGKHLY 240
                                                      **** :*::******.*.****

PtCDC16         SSAIFFADKVAAFTNDPADIYMQAQALFLGRHYRRAYHLLNASKIVLRDLRFRYLAAKCL 82
AtCDC16         SSAIFFADKVAALTNDPSDIYMQAQALFLGRHYRRAFHLLNASKIVLRDLRFRYLAAKCL 82
OsCDC16         ASAIFLADKVAAATGDPADVYMLAQALFLGRHFRRALHILNSSK-LLRDLRFRFLAAKCL 299
                :****:****** *.**:*:** *********:*** *:**:** :*******:******

PtCDC16         EELKEWDQCLLMLGDAKVDEHGDVYDTKDCNVMYLDKDSEDREINISAATCFLRGRAYEA 142
AtCDC16         EELKEWDQCLLMLGDAKVDDDGIVYDAKDGNVIDFDKDGEDREINISSAICFLRGKAYGA 142
OsCDC16         EELKEWHQCLIILGDAKIDEHGNVVDQDDGSDIYFDKDAEDHEINIKAAICFLRGKAYEA 359
                ******.***::*****:*:.* * * .* . : :***.**:****.:* *****:** *

PtCDC16         LENRALARQWYKAAIKADPLCYEALECLIENHMLTCEEETRLLSSLQFGPEDGWLSSFYS 202
AtCDC16         LQNRSQARQWYKAAIKADPLCYEALECLIESHMLTSEEESSLLSSLQFSPEDGWLSSFYS 202
OsCDC16         LDNCDLARQWYKAAVKADPLCYEALECLVDNYMLTCEEESELLSSLKFGKEDGWLSAFYS 419
                *:*   ********:*************::.:***.***: *****:*. ******:***

PtCDC16         CLIKKYEKKSVVEAKFREVEKESCNSNPSSPSIKHTLKNDTDLLTCKAEYFNQCGEYQKC 262
AtCDC16         CLIKKYDKESTVELKFKKLENETSGS--VSGSSMITLANNTDLLACKAEYYHQCCEYQKC 260
OsCDC16         CLIRKHEKEYIVEAKFKEFERESCSI--SSLSSGLTLKNNIDVLACKAEYYHQSGEYQKC 477
                ***:*::*:  ** **::.*.*:..    * *   ** *: *:*:*****::*. *****

PtCDC16         FELTSESSELYLLFHSLLEKDPFHLKCTLVHIAAAMELGNSNELYLMASNLVKDYPQKAL 322
AtCDC16         FELTA----------ALLEKDPFHLKCTLVHLAAAMELGNSNELYLMACNLVKDYPSKAL 310
OsCDC16         FELTS----------ALLERDPFHLKCTLVHLAAAMELGHSNDLYILACNLVKDYPQKAL 527
                ****:          :***:***********:*******:**:**::*.*******.***

PtCDC16         SWFAVGCYYYCIKKYDQSRRYFSKATSLEGTFAPAWIGFGNAYAAQEEGDQAMSAYRTAA 382
AtCDC16         SWFAVGCYYYCIKKYAEARRYFSKATGIDGSFSPARIGYGNSFAAQEEGDQAMSAYRTAA 370
OsCDC16         SWFAVGCYYYCIKKYDQARRYFGKATGLDGTFPPAWIGTGIAYAAQEEGDQAMAAFRTAA 587
                *************** ::****.***.::*:*.** ** * ::**********:*:****

PtCDC16         RLFPGCHLPTLYIGMEYMRTHSYKLAEQFFMQAKAICPSDPLVYNELGVVAYNMKEYNKS 442
AtCDC16         RLFPGCHLPTLYIGMEYMRTHSYKLADQFFMQAKAICPSDPLVYNELGVVAYHMKEYGKA 430
OsCDC16         RLFPGCHLPTLYMGMQYLRMHNFKLAEQFFTQAKSICPSDPLIYNEMGVVAYNMKEYQKA 647
                ************:**:*:* *.:***:*** ***:*******:***:*****:**** *:

PtCDC16         VLWFEKTLKHIP-SLSQLWEPTIVNLAHAYRKLKIYHEAISYYERALTLSPRSLSTYAGL 501
AtCDC16         VRWFEKTLAHIPSALTESWEPTVVNLAHAYRKLRKDREAISYYERALTLSTKSLSTYSGL 490
OsCDC16         VQWFELTLEHTSSSLNEMWEPTLVNLGHALRKLKKYQKAISYYEKALTFQTKSLSAFAGL 707
                * *** ** * . :*.: ****:***.** ***:  ::******:***:..:***:::**

PtCDC16         AYTYHLQ------------ALWLKPDDQFCTEMLSLALVDEGRRGTDPKIEFR 542
AtCDC16         AYTYHLQGNFSAAISYYHKALWLKPDDQFCTEMLNVALMDECQNGVDSKVELC 543
OsCDC16         AYTYHLMDKFEAAITYYHKALWLKPDDQFSTDMLTLALESSCQITARTR---- 756
                ******             **********.*:**.:** .. :  . .:
```

APC11

```
AtAPC11         -------------------------------MKVKILRWHAVASWTWDAQDETCGICRMA 29
PtAPC11         -------------------------------MKVKLLQWHAVASWTWDAQDETCGICRMA 29
OsAPC11_1       -------------------------------MKVKILQWHSVASWTWDAQDETCGICRMA 29
OsAPC11_2       MQVGGTAAAAAEVSMSYRETRREEEKRRGTKMKVKILQWHGVASWTWNAQDETCGICRMA 60
                                               ****:*:**.******:************

AtAPC11         FDGCCPDCKLPGDDCPLIWGACNHAFHLHCILKWVNSQTSQAHCPMCRREWQFKE 84
PtAPC11         FDGCCPDCKLPGDDCPLIWGACNHAFHLHCILKWVNSQTSQAHCPMCRREWQFKE 84
OsAPC11_1       FDGCCPDCKFPGDDCPLIWGSCNHAFHLHCILKWVNSQTSTPLCPMCRREWQFKG 84
OsAPC11_2       FDGCCPDCKFPGDDCPLIWGSCNHAFHLHCILKWVNSQTSTPLCPMCRREWQFKG 115
                *********:**********:******************* . ***********
```
