# Supplementary material for: Genomic evolution and complexity of the Anaphase-promoting Complex (APC) in land plants
Source: BMC Plant Biol. 2010 Nov 18;10:254. doi: 10.1186/1471-2229-10-254 (PMC3095333; doi:10.1186/1471-2229-10-254)
Supplement: Additional file 6 — Re-sequencing of 5'region of AtCDC20_6. [file 1471-2229-10-254-S6.PDF]

Additional file 6: re-sequencing of 5´region of *AtCDC20_6*.

```
actaaacaactttcaataattcttccgacgcaaaagactctcttgagaaaccctaatttcaactgaaaaaATGGCAGAAACCGCCGA
TACAATCAACACCACCGTCTCAACGCCGCAGCCACAATTAGAAAGCAGATCAGACGAGACAAGTTGCCTCCAAAAACACAGATCTGA
CGCAACATCAGAGGTGACGAAAGAAGAGAAATCTGGAGGAATCTTGTTCAGCGTTTGGCCACCGTGTCAGAAAAGTCGCGACTACGT
GGTGAATAGTATGATCAAGACCTTATCCACAGACTCGATCCTCTCCTACAAATACGGAACGATTAAACCTGAGGAAGCCTCCGCCGT
CGCGAAATCTATAGAGGAAAAGGCTTACGACATCGCATCAAGGTTCGTGTCGAGTGATGGGATTAAGAATCTTGAGGTCTATGGCAT
AGAGACTAGCGAGCGTATGATTGAATCTGCGGAGGTTCGATTCAAAGCCAATGGAAGTATGGAACTACTACTAAATCAAACAATAAA
GATGATGCAGTTGCTGATTTAGaggagaagcttgagcgttaagtttccttatctcttttatgatctatctctagggtttgcttttgc
tagatttgagattctcaaaccagatttgtttccttcttcttaatatgagtcgttgattgcttttgcattatgtggtttagtatcaca
cttattattcctatataatgttcaatttctttcgtccaccaccagtaactctatgcaacttccctcattaggttatgaataatcctt
gtaaacttaaaattatgattaaggctaggaggcacgttgatagctcaaacggattcgttgaggctcgtgaataagcaaagagaagca
gagaaacagagtgaaaacagagcaaggagaaacagattgtttaaccactagcttgagagtctagtgagttgtatataaatagataga
agagtattgcacaagttatgtattgatgtgtgtgtaacaaacgtatggtttgttagaggagagttaggagatctcttgaacaaatct
tcattgttagggcttggaagaattttaatctgaaactcacaaggcattgtaatctggaaattcaccattggcatctaaatccaacag
agccagagtgtttccacctattggacacagcactacttttaacaacatcttttgacggaagctgccactagagcatctgacacataa
aactgtgtcctcttgtcttgcactacaggttttgagttaatcgcttctctttcaaaacaactttgctccactgaattccattcacag
aattgaaccgaaaactacagtttttgagttcatcgcatcgatcgattactcaattagttgcttatttgcgtcaattatggacatcta
acattcagcgtttattgtatactgtattaaagctcttaatatagactcagacaaaaatagaattgagaattgcattcataatactta
ggaaaaccataccaatcagacaattaaaactcataatctaaaCTAGAAACAGATCGAGTCAGGGACTTGATAGAAGCTTCCGTTGTC
ATGGTTATAAAGAAAGACAGAATATTTGCTCTGATGTTGACTCAATGATGAAGTCGATAGCTCTTGCTCTGTGTTTGACACACCATT
CTCCAATGGTTCTGTCCATTGTTGATTATGGTGATTCTCAGGAGAAGACAATAGATTCGTTGTGGAAGAAACCGCACGGCTCTGACC
TAACTTGGTCTGGTTTTTCTCGGGTTGGAGTAACAGAAGCTTATTTTGCAGTACCCGTAGACCACTTTCTAACGAATCCTCCATCTC
TAAGACTTTCTCAGACAATTTATTGTCGTCGAGGATCTTCTTACGTAGAAACAGAGAAAGGTTTGTCCTTTTCTTGCATCGCTCTCT
CTCGTGTAATCTGCTAAACCTCTCAGCCATGTCTCGAACTTTTGATTGATCTTCCGGCCATGTCTTGATGAGTTCTCCGTCACGACC
ATAAAGTATGACACAGACTTCGATGTCGCAGAGAGTCGAAAGCTCCAAGGCTTTCTTAAACATGGTCTGTTCTCTCATAGACAAATT
CGTGGTCTTTTTTGCAGTGGAAGAAGAAGACAGAGACGATTTCTTGAAGCAAGTTTGGTTTCTCACAGACAATTTGGTTTTCTTCTT
CGTCGACATTGAAGAATACATTGTGATgttttgtttacacacagaacaaaaagacgcttttatttgttatcaaagaagagaaaataa
actctgatcgagagaattgggatacctaagTGAACAGTTGGGAGACACTGTTTACTTGTGGGATGCGTCTAGTTGTTATACATCTAA
GCTTGTGACCATTGATGATGAGAATGGACCTGTCACAAGTATTAACTGGACGCAAGATGGTCTTGATCTGGCAGTTGGTCTTGACAA
CAGTGAAGTCCAGGTTTGGGATTGTGTTTCCAATCGTCATGTGAGAACATTGAGAGGTGGTCACGAGTCAAGAGTAGGATCATTGGC
ATGGAACAATCACATTCTAACAACTGGAGGAATGGATGGAAAGATCGTCAACAATGATGTGAGGATCAGATCATCCATTATTGGAAC
TTACGTGGGTCACACTGAAGAGGTTTGTGGTCTTAAGTGGTCAGAATCTGGAAAAAAACTAGCAAGTGGTGGTAACGACAATGTAGT
ACACATATGGGATCGTTCTCTCGCTTCCTCAAACCCAACTAGGCAATGGCTGCATAGGTTTGAGGAACATACTGCTGCAGTGAGAGC
TCTTGCGTGGTGCCCTTTCCAAGCAAGTTTGCTTGCAACTGGTGGTGGTGTTGGAGATGGGAAGATTAACTTCTGGAATACACACAC
TGGGGCTTGCTTGAATTCAGTAGAAACTGGCTCTCAAGTATGTTCATTGTTATGGAGCAAAAGTGAAAGAGAGTTGCTTAGCGCACA
TGGGTTTACGCAGAACCAGCTTACACTTTGGAAGTACCCATCTATGGTGAAAATGGCTGAACTCAATGGTCATACATCAAGAGTTCT
TTTTATGGCACAGAGTCCGGATGGTTGTACCGTGGCTTCAGCAGCAGGTGACGAGACTCTCAGGCTTTGGAATGTTTTTGGAGAACC
ACCTAAGACCACCAAGAAAGCTGCTTCAAAAAAATATACGGAGCCATTTGCTCATGTGAATCATATTAGGTGAatctactgtgaaga
ctaacaaggagaaaaccattgataggtaaataatgtatagagaagcaatgtattcatctttttttcctgattcattattcat
```

Sequencing

At5g27945

Sequencing 5´region of At5g27945

```
CGTTAAGTTTCCTTATCTCTTTTATGATCTATCTCTAGGGTTTGCTTTTGCTAGATTTGAGATTCTCAAACCAGATTTGTTTCCCTT
CTTCTTAATATGAGTCGTTGATTGCTTTTGCATTATGTGGTTTAGTATCACACTTATTATTCCTATATAATGTTCAATTTCTTTCGT
CCACCACCAGTAACTCTATGCAACTTCCCTCATTAGGTTATGAATAATCCTTGTAAACTTAAAATTATGATTAAGGCTAGGAGGCAC
GTTGATAGCTCAAACGGATTCGTTGAGGCTCGTGAATAAGCAAAGAGAAGCAGAGAAACAGAGTGAAAACAGAGCAAGGAGAAACAG
ATTGTTTAACCACTAGCTTGAGAGTCTAGTGAGTTGTATATAAATAGATAGAAGAGTATTGCACAAGTTATGTATTGATGTGTGTGT
AACAAACGTATGGTTTGTTAGAGGAGAGTTAGGAGATCTCTTGAACAAATCTTCATTGTTAGGGCTTGGAAGAATTTTAATCTGAAA
CTCACAAGGCATTGTAATCTGGAAATTCACCATTGGCATCTAAATCCAACAGAGCCAGAGTGTTTCCACCTATTGGACACAGCACTA
CTTTTAACAACATCTTTTGACGGAAGCTGCCACTAGAGCATCTGACACATAAAACTGTGTCCTCTTGTCTTGCACTACAGGTTTTGA
GTTAATCGCTTCTCTTTCAAAACAACTTTGCTCCACTGAATTCCATTCACAGAATTGAACCGAAAACTACAGTTTTTGAGTTCATCG
CATCGATCGATTACTCAATTAGTTGCTTATTTGCGTCAATTATGGACATCTAACATTCAGCGTTTATTGTATACTGTATTAAAGCTC
TTAATATAGACTCAGACAAAAATAGAATTGAGAATTGCATTCATAATACTTAGGAAAACCATACCAATCAGACAATTAAAACTCATA
ATCTAAACTAGAAACAGATCGAGTCAGGGACTTGATAGAAGCTTCCGTTGTCATGGTTATAAAGAAAGACAGAATATTTGCTCTGAT
GTTGACTCAATGATGAAGTCGATAGCTCTTGCTCTGTGTTTGACACACCATTCTCCAATGGTTCTGTCCATTGTTGATTATGGTGAT
TCTCAGGAGAAGACAATAGATTCGTTGTGGAAGAAACCGCACGGCTCTGACCTAACTTGGTCTGGTTTTTCTCGGGTTGGAGTAACA
GAAGCTTATTTTGCAGTACCCGTAGACCACTTTCTAACGAATCCTCCATCTCTAAGACTTTCTCAGACAATTTATTGTCGTCGAGGA
TCTTCTTACGTAGAAACAGAGAAAGGTTTGTCCTTTTCTTGCATCGCTCTCTCTCGTGTAATCTGCTAAACCTCTCAGCCATGTCTC
GAACTTTTGATTGATCTTCCGGCCATGTCTTGATGAGTTCTCCGTCACGACCATAAAGTATGACACAGACTTCGATGTCGCAGAGAG
TCGAAAGCTCCAAGGCTTTCTTAAACATGGTCTGTTCTCTCATAGACAAATTCGTGGTCTTTTTTGCAGTGGAAGAAGAAGACAGAG
ACGATTTCTTGAAGCAAGTTTGGTTTCTCACAGACAATTTGGTTTTCTTCTTCGTCGACATTGAAGAATACATTGTGATGTTTTGTT
TACACACAGAACAAAAAGACGCTTTTATTTGTTATCAAAGAAGAGAAAATAAACTCTGATCGAGAGAATTGGGATACCTAAGTGAAC
AGTTGGGAGACACTGTTTACTTGTGGGATGCGT
```
