# Supplementary material for: Genomic evolution and complexity of the Anaphase-promoting Complex (APC) in land plants
Source: BMC Plant Biol. 2010 Nov 18;10:254. doi: 10.1186/1471-2229-10-254 (PMC3095333; doi:10.1186/1471-2229-10-254)
Supplement: Additional file 7 — Correction of APC activator sequences. Gray boxes: The conserved APC interacting motifs C-box, CSM, IR-tail, CBM. Differences are boxed in yellow. [file 1471-2229-10-254-S7.PDF]

Additional file 7: Correction of APC activator sequences. Gray boxes: The conserved APC interacting motifs C-box, CSM, IR-tail, CBM. Differences are boxed in yellow.

Before EST support: Activators CDC20 and CCS52

```
OsCDC20_2        ----------MDA-GSHSISSEKSSRYVAPRQP---LQEAGSR-PYMPSLSTASRNPSA- 44
OsCDC20_3        ----------MDA-GSHSISSEKS-HGLAPRPP---LQEAGSR-PYMPSLSTASRNPSA- 43
OsCDC20_1        ----------MEAPGSGSVPTAKR-RRLVPRPPPVPLEVAGARGPYMPPLCIKSKNPSA- 48
PtCDC20_4        ------------------------------------------------------------
AtCDC20_3        --------------------------------------MDSGMRATCTVPEHFLPRKLS- 21
AtCDC20_4        --------------------------------------MDS---DTCTVPDHFLPRKLS- 18
AtCDC20_5        ------------------------------------------------------------
AtCDC20_6        --------------------------------------------------------MMK- 3
AtCDC20_1        -----------------------------MDAGLN----------RCPLQEHFLPRKNS- 20
AtCDC20_2        -----------------------------MDAGMNNTSSHYKTQARCPLQEHFLPRKPS- 30
PtCDC20_1        -----------------------------MDAGSINSSSSLKAQSRFPLQQQFLPRMNS- 30
PtCDC20_3        -----------------------------MDAGSMNTSSSLKAQSRFPLQQQFLPRTNS- 30
PtCDC20_2        -----------------------------MDAGSLNSSSYMKAQSRFPLQEQFLHRKNS- 30
PtCDC20_5        ------------------------------------------------------------
AtCCS52A1        ---MEEEDPTASNVITNSNSSSMRNLSPAMNTPVVSLESR----INRLINANQSQSPSP- 52
AtCCS52A2        ---MEEDESTTP----KKKSDSQLNLPPSMNRPTVSLESR----INRLIDSNHYHSPS-- 47
PtCCS52A1        --------------------------------PSPSSSEH----VNRLINSNHYISPS-- 22
PtCCS52A1_2      -------------------------------TPSPSSSKH----VNRLINSNHYISPS-- 23
OsCCS52A1        MDHHHHHLPPPPPRSPMENSASSKPPTPASTPSSRLAAAP----SSRVSSAAPHPSPSSS 56
PtCCS52B         ---MDSTPRRKSG---LNLPSGMNETSLRLETFSSSSSFRAVTCVSSPRAISSLSSPSKT 54
PtCCS52B_2       ---MDSTPRRKSG---LNLPSGMNETSLRLETFSSSSSFRAVTCVSSPRAISSLSSPSKT 54
AtCCS52B         ---MASPQSTKTG---LNLPAGMNQTSLRLETFSSS-----------FRGISSLSSPSK- 42
OsCCS52B         ---MATDASPKPAPPRLNVPPAMAG-GLRLDPAVASP-------ARLLLDVPKTPSPSK- 48


OsCDC20_2        -------KCYGDRFIPDRSAMDMDMAHYLLTE-----------------PRKDKEN-AAA 79
OsCDC20_3        -------KCYGDRFIPDRSAMDMDMAHYLLTE-----------------PKKDKEN-AAA 78
OsCDC20_1        -------KCYGDRFIPDRSAMDMDMAYFLLTE-----------------PKKEKENTDML 84
PtCDC20_4        ----------LDRFIPNRSAMDMDFAHYMLTE-----------------GRKAKES---- 29
AtCDC20_3        -------KQNLDRFIPNRSAKDFDFANYALTQ-----------------GSKRN-LDEVT 56
AtCDC20_4        -------KQNLDRFIPNRSAMDFDFANYALTQ-----------------GRKRN-VDEIT 53
AtCDC20_5        --------------------MDFDFANYALTQ-----------------GRKRN-VDEVT 22
AtCDC20_6        -------SIALALCLTHHSPMVLSIVDYGDSQ-----------------EKTIDSLWKKP 39
AtCDC20_1        -------KENLDRFIPNRSAMNFDYAHFALTEERK--------------G-KDQS-ATVS 57
AtCDC20_2        -------KENLDRFIPNRSAMNFDYAHFALTEGRK--------------G-KDQT-AAVS 67
PtCDC20_1        -------KENLDRFIPNRSAMDMDYAHFMLTEGRK--------------G-KENP--TVN 66
PtCDC20_3        -------KENLDRFIPNRSAMDMDYARFMLTEGRK--------------G-KENP--TVN 66
PtCDC20_2        -------KDNLDRFIPNRSAMDLDYAHYMLTQGRK--------------GGKENPTATVN 69
PtCDC20_5        ------------------------------------------------------------
AtCCS52A1        --SSLSRSIYSDRFIPSRSGSNFALFDLSPSPS---------------KDGKEDGAGSYA 95
AtCCS52A2        ------KPIYSDRFIPSRSGSNFALFDLASSSPNK-------------KDGKEDGAGSYA 88
PtCCS52A1        ------RPIYSDRFIPCRSSSNFALFNISLPSPSATAGSSPG------DGGKEDNPNAYA 70
PtCCS52A1_2      ------RPIYSDRFIPCRSSSNFALFNISFPQPSATAGISPG------CGGKEDNPSAYA 71
OsCCS52A1        APTPASRTVYSDRFIPSRAGSNLALFDLAPSPSHHDAAAAAASPGAPPPSGSTPASSPYC 116
PtCCS52B         -------SSCSDRFIPCRSSSRLHTFGLVEKG----------------SPVKEGGNEAYA 91
PtCCS52B_2       -------SSCSDRFIPCRSSSRLQTFGLIEKG----------------SPVKEGGNEAYA 91
AtCCS52B         -------STCSDRFIPCRSSSRLHAFDLQDKEPT--------------TPVKEGGNEAYS 81
OsCCS52B         -------TTYSDRFIPCRSSSRLHNFALLDRDRA--------------SPSSTTDDAPYS 87


OsCDC20_2        SPAKEAYR-----KLLAEKILNNRT---------------RILSFRNKPPEPESILTE-L 118
OsCDC20_3        SPSKEVYR-----RLLAEKLLNNRT---------------RILAFRNKPPEPENVS---A 115
OsCDC20_1        SPAEEAYK-----RLLAEKLLNNRS---------------RILAFRNKPPEPEGIVQQLL 124
PtCDC20_4        PPSQSLYQ-----KLLAEAFNMNGR---------------RILAFKNKPPTLVDPIP--L 67
AtCDC20_3        SASRKAY-----MTQLAVVMNQNRT---------------RILAFRNKPKS----LLSTN 92
AtCDC20_4        SASRKAY-----MTQLAVVMNQNRT---------------RILAFRNKPKA----LLSSN 89
AtCDC20_5        SASRKAY-----MTQLAEAMNQNRT---------------RILAFRNKPKA----LLSSN 58
AtCDC20_6        HGSDLTWSG---FSRVGVTEAYFAV---------------PVDHFLTNPPS----LRLS- 76
AtCDC20_1        SPSKEAYR-----KQLAETMNLNHT---------------RILAFRNKPQAPVELLPSNH 97
AtCDC20_2        SPSKEAYR-----KQLAETMNLNHT---------------RILAFRNKPQAPVELLPSNH 107
PtCDC20_1        SPSREAYR-----KQLAESLNMNRT---------------RILAFKNKPPAPVELMPQDH 106
PtCDC20_3        SPSREAYR-----KQLADSLNMNRT---------------RILAFKNKPPAPVELMPQDH 106
PtCDC20_2        SPSREAYR-----KQLAEALNLNRT---------------RILAFKNKPPTPVELIPRDH 109
PtCDC20_5        ------------------------------------------------------------
AtCCS52A1        TLLRAAMFGPETPEKRDIT-GFSSS--------------RNIFRFKTETH--RSLN--SF 136
AtCCS52A2        SLLKTALFGPVTPEKSDVVNGFSPS--------------GNIFRFKTETQ--RSLN--LY 130
PtCCS52A1        ALLRNALFGPQTPDKKDWGTGAAG---------------RNIFRYKTETR--QSMH--SL 111
PtCCS52A1_2      ALLRNALFGPQTPDKKDWGAGAAG---------------QNIFRYKMETR--QSLH--SL 112
OsCCS52A1        ALLRAALFGPTTPDRVASSASACSSSSSAGASPVGSPATGNIFRFKAEVP--RNAKRALF 174
PtCCS52B         RLLKSELFGSDFGS-FSSPAGGQGGLSS---------PNKNMLRFKTDHS--GPNSPFSP 139
PtCCS52B_2       RLLKSELFGSDFGS-FSSPAGGQGGLGS---------PSKNMLRFKTDHS--GPNSPYSP 139
AtCCS52B         RLLKSELFGSDFASPLLSPAGGQGSASSP------MSPCTNMLRFKTDRSNSSPSSPFSP 135
OsCCS52B         RLLRAEIFGPDSPS----PAPSS--------------PNTNLFRFKTDHP--SPKSPFAA 127
```

```
OsCDC20_2        RA----------DAASIQAKPAKQRRYIPQSAERT------------------------- 143
OsCDC20_3        AD----------TASTHQAKPAKQRRYIPQSAERT------------------------- 140
OsCDC20_1        YE----------TLTSSQTKPARKCRHIPQSSERT------------------------- 149
PtCDC20_4        FS----------SSSVHSSKPVKPQRHIPQRPEMT------------------------- 92
AtCDC20_3        HS----------DSPHQNPKPVKPRRYIPQNSERV------------------------- 117
AtCDC20_4        HS----------DSPHQNPKSVKPRRYIPQNSERV------------------------- 114
AtCDC20_5        HS----------DPPHQQPISVKPRRYIPQNSERV------------------------- 83
AtCDC20_6        QT----------IYCRRGSSYVETEKVEEEDRDDF------------------------- 101
AtCDC20_1        S-----------ASLHQQPKSVKPRRYIPQTSERT------------------------- 121
AtCDC20_2        S-----------ASLHQQPKSVKPRRYIPQTSERT------------------------- 131
PtCDC20_1        ------------SHHHHQPKTAKPRRHIPQTSERT------------------------- 129
PtCDC20_3        ------------SHHHHQPKTAKPRRHIPQTSERT------------------------- 129
PtCDC20_2        LS----------SSLHYQAKPTKPRRYIPQTSERT------------------------- 134
PtCDC20_5        ------------------------------------------------------------
AtCCS52A1        SPFGVD----DDSPGVSHSGPVKAPRKVPRSPYKV------------------------- 167
AtCCS52A2        PPFDSD----VVS-GVSPS-PVKSPRKILRSPYKV------------------------- 159
PtCCS52A1        SPFGFD----GLSGPGVSNVAIKAPRKVSRSPYKV------------------------- 142
PtCCS52A1_2      SPFGFD----DMSDLGVSNVAIKTPRKVSRSPYKVGVLVNWCHILTFLRFDEIWIFIGIF 168
OsCCS52A1        SDGDDE----GVLFPGVFTTRGTGPRKIPRSPYKV------------------------- 205
PtCCS52B         SILGHD-----SGISSESSTPPKPPRKVPKTPHKV------------------------- 169
PtCCS52B_2       SILGHD-----SGISSESSTTPKPPRKVPKTPHKV------------------------- 169
AtCCS52B         SILGND-----NGHSSDSSPPPKPPRKVPKTPHKV------------------------- 165
OsCCS52B         SAAATAGHYDCTAGSAESSTPRKPPRKVPKTPHKV------------------------- 162


OsCDC20_2        -----------LDAPELVDDYYLNLLDWGSSNVLSIALGNSVYLWDATNSSTSELVTVDE 192
OsCDC20_3        -----------LDAPDLVDDYYLNLLDWGSKNVLSIALGDTVYLWDASSGSTSELVTVDE 189
OsCDC20_1        -----------LDAPGIVDDFYLNILDWGCKNVMSIALGNTLYLWNSADGSIMDLVTIDE 198
PtCDC20_4        -----------LDAPDIVDDFYLNLLDWGNNNVLAIALGTTVYLWNASNSSISEVVTVDE 141
AtCDC20_3        -----------LDAPGLRDDFSLNLLDWGSANVLAIALGDTVYLWDASSGSTSELVTIDE 166
AtCDC20_4        -----------LDAPGLMDDFYLNLLDWGSANVLAIALGDTVYLWDASSGSTSELVTIDE 163
AtCDC20_5        -----------LDAPGIADDFYLNLLDWGSSNVLAIALGDTVYLWDASSGSTYKLVTIDE 132
AtCDC20_6        -----------LKQVWFLTDNLVFFFVDIEEYIVIEQLGDTVYLWDASSCYTSKLVTIDD 150
AtCDC20_1        -----------LDAPDIVDDFYLNLLDWGSANVLAIALDHTVYLWDASTGSTSELVTIDE 170
AtCDC20_2        -----------LDAPDIVDDFYLNLLDWGSANVLAIALDHTVYLWDASTGSTSELVTIDE 180
PtCDC20_1        -----------LDAPDLVDDFYLNLLDWGSSNVLAIALGSTVYLWDASDGSTSELVTVDD 178
PtCDC20_3        -----------LDAPDLVDDFYLNLLDWGSSNVLAIALGSTVYLWDASDGSTSELVTVDD 178
PtCDC20_2        -----------LDAPDLVDDFYLNLLDWGSKNVLAIALENTVYLWDASNGSTSELVTVGD 183
PtCDC20_5        ------------------------------------------------------------
AtCCS52A1        -----------LDAPALQDDFYLNLVDWSAQNVLAVGLGNCVYLWNACSSKVTKLCDLGA 216
AtCCS52A2        -----------LDAPALQDDFYLNLVDWSAQNVLAVGLGNCVYLWNACSSKVTKLCDLGV 208
PtCCS52A1        -----------LDAPALHDDFYLNLVDWSSHNVLAVGLGTCVYLWNACSSKVTKLCDLGN 191
PtCCS52A1_2      LLIDPAFCFYVLDAPALQDDFYLNLVDWSSHNVLAVGLGNCVYLWNACSSKVTKLCDLGN 228
OsCCS52A1        -----------LDAPALQDDFYLNLVDWSSHNILAVGLGNCVYLWNACSSKVTKLCDLGV 254
PtCCS52B         -----------LDAPSLQDDFYLNLVDWSSQNVLAVGLGTCVYLWTASNSKVTRLCDLGP 218
PtCCS52B_2       -----------LDAPSLQDDFYLNLVDWSSQNVLAVGLGTCVYLWTASNSKVTRLCDLGP 218
AtCCS52B         -----------LDAPSLQDDFYLNVVDWSSQNVLAVGLGTCVYLWTASNSKVTKLCDLGP 214
OsCCS52B         -----------LDAPSLQDDFYLNLVDWSSQNTLAVGLGNCVYLWSASNCKVTKLCDLGP 211


OsCDC20_2        DNGPVTSVSWAPDGRHIAVGLNSSDVQLWDTSSNRLLRTMRGVHDSRVGSLAWNNNILTT 252
OsCDC20_3        DSGPITSVSWAPDGQHVAVGLNSSDIQLWDTSSNRLLRTLRGVHESRVGSLAWNNNILTT 249
OsCDC20_1        DDGPITSVSWSCDGQWIAVGLNSSDIQLWDTSSNRMLRTLHGVHQSRVGSLAWNKNILTT 258
PtCDC20_4        EDGPVTSISWAPDGRHLAVGLDNSNVQLWDSATNQMLRTLRGGHRLRVTSLAWNHHLLTT 201
AtCDC20_3        DKGPVTSINWTQDGLDLAVGLDNSEVQLWDCVSNRQVRTLRGGHESRVGSLAWDNHILTT 226
AtCDC20_4        DKGPVTSINWTQDGLDLAVGLDNSEVQLWDFVSNRQVRTLIGGHESRVGSLAWNNHILTT 223
AtCDC20_5        EEGPVTSINWTQDGLDLAIGLDNSEVQLWDCVSNRQVRTLRGGHESRVGSLAWNNHILTT 192
AtCDC20_6        ENGPVTSINWTQDGLDLAVGLDNSEVQVWDCVSNRHVRTLRGGHESRVGSLAWNNHILTT 210
AtCDC20_1        EKGPVTSINWAPDGRHVAVGLNNSEVQLWDSASNRQLRTLKGGHQSRVGSLAWNNHILTT 230
AtCDC20_2        EKGPVTSINWAPDGRHVAVGLNNSEVQLWDSASNRQLRTLKGGHQSRVGSLAWNNHILTT 240
PtCDC20_1        EDGPITSVNWAPDGRHIAIGLNNSHIQLWDSASNRQLRTLKGGHRSRVGSLAWNNHILTT 238
PtCDC20_3        EDGPVTSVNWAPDGRHIAIGLNNSHIQLWDSASNRQLRTLKGGHRSRVGSMAWNNHILTT 238
PtCDC20_2        EVGPVTSVNWAPDGLHLAIGLNNSNVQLWDSASCKQLRNLRGCHRSRVGSMAWNNHILTT 243
PtCDC20_5        ------------------------------------------------------------
AtCCS52A1        EDS-VCSVGWALRGTHLAVGTSTGKVQIWDASRCKRTRTMEG-HRLRVGALAWGSSVLSS 274
AtCCS52A2        DET-VCSVGWALRGTHLAIGTSSGTVQIWDVLRCKNIRTMEG-HRLRVGALAWSSSVLSS 266
PtCCS52A1        DDG-VCSVGWAHRGTHLAVGTSNGKVQIWDASRCKRIRIMEG-HRLRVGALAWSSSMLSS 249
PtCCS52A1_2      DDG-VCSVGWAHRGTHLAIGTSNGKVQIWDASRCKRIRTMEG-HRLRVGALAWSSSMLSS 286
OsCCS52A1        DDN-VCSVGWAQRGTHLAVGTNQGKVQVWDATRCKRIRTMES-HRMRVGALAWNSSLLSS 312
PtCCS52B         NDS-VCSVQWTREGSYISVGTHLGQVQVWDGTQCKRVRTMGG-HQTRTGVLAWNSRTLAS 276
PtCCS52B_2       NDS-VCSLQWTREGSYISIGTHLGQVQVWDGTQCKRVRTMGG-HQTRTSVLAWNSRTLAS 276
AtCCS52B         NDS-VCSVQWTREGSYISIGTSHGQVQVWDGTQCKRVRTMGG-HQTRTGVLAWNSRILSS 272
OsCCS52B         RDS-VCAVHWTREGSYLAIGTSLGDVQIWDSSRCKRIRNMGG-HQTRTGVLAWSSRILSS 269
```

```
OsCDC20_2        GGMDGKIVNNDVRIRNHVVQTYQGHQQEVCGLKWSGSGQQLASGGNDNLLHIWD-VSMAS 311
OsCDC20_3        GGMDGNIVNNDVRIRNHVVQTYQGHSQEVCGLKWSGSGQQLASGGNDNLLHIWD-VSMAS 308
OsCDC20_1        GGMDGNIVNNDVRMRSHVVHIYRGHEDEVCGLRWSGSGQQLASGGNDNLVHIWD-VSMAS 317
PtCDC20_4        GGKDAKVINNDVRIREHIVESYEGHRQEVCGLKWSASGQQLASGGNDNLLFIWD-RFMAS 260
AtCDC20_3        GGMDGKIVNNDVRIRSSIVETYLGHTEEVCGLKWSESGNKQASGGNDNVVHIWD-RSLAS 285
AtCDC20_4        GGMDGKIVNNDVRIRSSIVGTYLGHTEEVCGLKWSESGKKLASGGNYNVVHIWDHRSVAS 283
AtCDC20_5        GGMDGKIVNNDVRIRSSIVETYLGHTEEVCGLKWSESGKKLASGGNDNVVHIWDHRSVAS 252
AtCDC20_6        GGMDGKIVNNDVRIRSSIIGTYVGHTEEVCGLKWSESGKKLASGGNDNVVHIWD-RSLAS 269
AtCDC20_1        GGMDGLIINNDVRIRSPIVETYRGHTQEVCGLKWSGSGQQLASGGNDNVVHIWD-RSVAS 289
AtCDC20_2        GGMDGLIINNDVRIRSPIVETYRGHTQEVCGLKWSGSGQQLASGGNDNVVHIWD-RSVAS 299
PtCDC20_1        GGMDGQIINNDVRIRSHIVETYRGHTQEVCGLKWSASGQQLASGGNDNLIHIWD-RSTAL 297
PtCDC20_3        GGMDGQIINNDVRIRSHIVETYRGHTQEVCGLKWSASGQQLASGGNDNLIHIWD-RSTAL 297
PtCDC20_2        GGMDGKIINNDVRIRSHIVETYRGHQQEVCGLKWSASGQQLASGGNDNIIHIWD-RSVAS 302
PtCDC20_5        ------------------------------------------------------------
AtCCS52A1        GSRDKSILQRDIRCQEDHVSKLAGHKSEVCGLKWSYDNRELASGGNDNRLFVWN---QHS 331
AtCCS52A2        GSRDKSILQRDIRTQEDHVSKLKGHKSEICGLKWSSDNRELASGGNDNKLFVWN---QHS 323
PtCCS52A1        GSRDKSILQRDIRAQEDFVSKLSGHKSEVCGLKWSYDNRELASGGNDNRLFVWN---QHS 306
PtCCS52A1_2      GSRDKSILQRDIRAREDFVSKLSGHKSEVCGLKWSYDNRELASGGNDNRLFVWN---QHS 343
OsCCS52A1        GSRDKSILHHDIRAQDDYISRLAGHKSEVCGLKWSYDNRQLASGGNDNRLYVWN---QHS 369
PtCCS52B         GSRDRHILQHDLRISSDYVSKLIGHKSEVCGLKWSHDDRELASGGNDNQLLVWN---QHS 333
PtCCS52B_2       GSRDRNILQHDLRVSSDHVSKLIGHKSEVCGLKWSHDDRELASGGNDNQLLVWN---QHS 333
AtCCS52B         GSRDRNILQHDIRVQSDFVSKLVGHKSEVCGLKWSHDDRELASGGNDNQLLVWN---NHS 329
OsCCS52B         GSRDKNILQHDIRVPSDYISKFSGHRSEVCGLKWSHDDRELASGGNDNQLLVWN---QRS 326


OsCDC20_2        SMPSAGRTQWLHRLEDHLAAVKALAWCPFQSNLLASGGGGSDRCIKFWNTHTGACLNSID 371
OsCDC20_3        SVPSAGRNQWLHRLEDHTAAVKALAWCPFQSNLLATGGGGSDRCIKFWNTHTGACLNSVD 368
OsCDC20_1        SNLSLGHNRWLHRFGDHLAAVKALAWCPFQSNLLASGGGGDDRCIRFWNTHTGLCLNSVD 377
PtCDC20_4        SNSPR---HWLHKLEDHTAAVKALAWCPFQSNLLASGGGGNDRHIKFWNTQTGTCLNSVD 317
AtCDC20_3        SKQTR---QWLHRFEEHTAAVRALAWCPFQASLLATGGGVGDGKIKFWNTHTGACLNSVE 342
AtCDC20_4        SKPTR---QWLHRFEEHTAAVRALAWCPFQATLLATGGGVGDGKIKFWNTHTGACLNSVE 340
AtCDC20_5        SNPTR---QWLHRFEEHTAAVRALAWCPFQASLLATGGGVGDGKIKFWNTHTGACLNSVE 309
AtCDC20_6        SNPTR---QWLHRFEEHTAAVRALAWCPFQASLLATGGGVGDGKINFWNTHTGACLNSVE 326
AtCDC20_1        SNSTT---QWLHRLEEHTSAVKALAWCPFQANLLATGGGGGDRTIKFWNTHTGACLNSVD 346
AtCDC20_2        SNSTT---QWLHRLEEHTSAVKALAWCPFQANLLATGGGGGDRTIKFWNTHTGACLNSVD 356
PtCDC20_1        SNSAT---QWLHRLEDHTSAVKALAWCPFQGNLLASGGGGGDKSIKFWNTHTGACLNSID 354
PtCDC20_3        SNSAT---QWLHRLEDHTSAVKALAWCPFQGNLLASGGGGGDKSIKFWNTHTGACLNSID 354
PtCDC20_2        SNSAT---QWFHRLEEHTSAVKALAWCPFQGNLLASGGGGGDRSIKFWNTHTGACLNSID 359
PtCDC20_5        ----------------HTSAVKALAWCPFQGNLLASGGGGGDRSIKFWNTHTGACLNSID 44
AtCCS52A1        TQP-------VLKYSEHTAAVKAIAWSPHVHGLLASGGGTADRCIRFWNTTTNTHLSSID 384
AtCCS52A2        TQP-------VLRFCEHAAAVKAIAWSPHHFGLLASGGGTADRCIRFWNTTTNTHLNCVD 376
PtCCS52A1        TQP-------VLKYCEHTAAVKAIAWSPHLHGLLASGGGTADRCIRFWNTTTNSHLSCMD 359
PtCCS52A1_2      SQP-------VLKYCDHTAAVKAIAWSPHLHGLLASGGGTADRCIRFWNTTTNSHLSCID 396
OsCCS52A1        AHP-------VLKYTEHTAAVKAIAWSPHLHGLLASGGGTADRCIRFWNTTTNMHLNCVD 422
PtCCS52B         QLP-------ILKLTEHTAAVKAIAWSPHQSGLLASGGGTADRCIRFWNTTNGHQLNYVD 386
PtCCS52B_2       QLP-------VLTLTEHTAAVKAIAWSPHQSGLLASGGGTADRCIRFWNTTNGHQLNHVD 386
AtCCS52B         QQP-------ILKLTEHTAAVKAITWSPHQSSLLASGGGTADRCIRFWNTTNGNQLNSID 382
OsCCS52B         QQP-------ILRLTEHTAAVKAIAWSPHQQGLLASGGGTADRCIRFWNTVNGNMLNSVD 379
                                 *  :**:*::*.*.    ***:***   *   *.****  ..   *.  ::

OsCDC20_2        TGSQVCSLVWNKNERELLSSHGFAQNQLTLWKYPSMVKMAELTGHTSRVLFTAQVIFCSL 431
OsCDC20_3        TGSQVCALLWNKNERELLSSHGFTQNQLTLWKYPSMVKMAELTGHTSRVLFMAQ------ 422
OsCDC20_1        TGS---------------------QNSLALWKYPSMVKLAELEDHTARVLCLAQ------ 410
PtCDC20_4        TGSQVCALQWNKHERELLSSHGFTENQLILWKYPSMVKMAELSGHTSPVLFMTQ------ 371
AtCDC20_3        TGSQVCSLLWSQSERELLSSHGFTQNQLTLWKYPSMSKMAELNGHTSRVLFMAQ------ 396
AtCDC20_4        TGSQVCSLLWSQRERELLSSHGFTQNQLTLWKYPSMSKMAELNGHTSRVLFMAQ------ 394
AtCDC20_5        TGSQVCSLLWSKSERELLSSHGFTQNQLTLWKYPSMVKMAELNGHTSRVLFMAQ------ 363
AtCDC20_6        TGSQVCSLLWSKSERELLSAHGFTQNQLTLWKYPSMVKMAELNGHTSRVLFMAQ------ 380
AtCDC20_1        TGSQVCSLLWSKNERELLSSHGFTQNQLTLWKYPSMVKMAELTGHTSRVLYMAQ------ 400
AtCDC20_2        TGSQVCSLLWSKNERELLSSHGFTQNQLTLWKYPSMVKMAELTGHTSRVLYMAQ------ 410
PtCDC20_1        TGSQVCSLLWNKNERELLSSHGFTQNQLTVWKYPSMVKMAELTGHTSRVLYMAQ------ 408
PtCDC20_3        TGSQVCSLLWNKNERELLSSHGFTQNQLTVWKYPSMVKMAELTGHTSRVLYMAQ------ 408
PtCDC20_2        TGSQVCALLWNKNERELLSSHGFTQNQLVLWKYPSMLKMAELTGHTSRVLYMAQ------ 413
PtCDC20_5        TGSQVCALLWNKNERELLSSHGFTQNQLVLWKYPSMLKMAELTGHTSRVLYMAQ------ 98
AtCCS52A1        TCSQVCNLAWSKNVNELVSTHGYSQNQIIVWKYPTMSKIATLTGHTYRVLYLAV------ 438
AtCCS52A2        TNSQVCNLVWSKNVNELVSTHGYSQNQIIVWKYPTMSKLATLTGHSYRVLYLAV------ 430
PtCCS52A1        TGSQVCNLVWSKNVNELVSTHGYSQNQIIVWRYPTMSKLATLTGHTYRVLYLAI------ 413
PtCCS52A1_2      TGSQVCNLVWSKNVNELVSTHGYSQNQIILWRYPTMSKLATLTGHTFRVLYLAI------ 450
OsCCS52A1        TGSQVCNLVWSKNVNELVSTHGYSQNQIIVWRYPTMSKLATLTGHTYRVLYLAI------ 476
PtCCS52B         TGSQVCNLAWSKNVNELVSTHGYSQNQIMVWKYPSLSKVATLVGHSLRVLYLAM------ 440
PtCCS52B_2       TGSQVCNLAWSKNVNELVSTHGYSQNQIMVWKYPSLSKVATLVGHSMRVLYLAM------ 440
AtCCS52B         TGSQVCNLAWSKNVNEIVSTHGYSQNQIMLWKYPSMSKVATLTGHSMRVLYLAT------ 436
OsCCS52B         TGSQVCNLAWCKNVNELVSTHGYSQNQIMVWKYPSMSKVATLTGHTLRVLYLAM------ 433
                 * *                     :*.: :*:**:: *:* * .*:  **  :
```

```
OsCDC20_2         YLPFKLTNIALNRLLITCLVVILQSPDGLTVASAAADETLRFWNVFG-APEAPKTATK-- 488
OsCDC20_3         ------------------------SPDGCTVASAAADETLRFWNVFG-SPEAPKPAAK-- 455
OsCDC20_1         ------------------------SPDGFTVASVAADETLRLWKIFE-TSEDAKPVFKTF 445
PtCDC20_4         ------------------------SPDGYTVASAAGDETLRFWNVFG-NPKAAK------ 400
AtCDC20_3         ------------------------SPNGCTVASAAGDENLRLWNVFGEPPKTTKKAAS-- 430
AtCDC20_4         ------------------------SPNGCTVASAAGDENLRLWNVFGEPPKTTKKAAS-- 428
AtCDC20_5         ------------------------SPDGCTVASAAGDETLRLWNVFGEPPKTTKKAAS-- 397
AtCDC20_6         ------------------------SPDGCTVASAAGDETLRLWNVFGEPPKTTKKAAS-- 414
AtCDC20_1         ------------------------SPDGCTVASAAGDETLRFWNVFG-VPETAKKAAP-- 433
AtCDC20_2         ------------------------SPDGCTVASAAGDETLRFWNVFG-VPETAKKAAP-- 443
PtCDC20_1         ------------------------SPDGCTVATAAGDETLRFWNVFG-VPEVAAKAAP-- 441
PtCDC20_3         ------------------------SPDGCTVATAAGDETLRFWNVFG-VPEIAAKAAP-- 441
PtCDC20_2         ------------------------SPDGCTVATAAGDETLRFWNVFG-VPEVAK------ 442
PtCDC20_5         ------------------------SPDGCTVATAAGDETLRFWNVF-------------- 120
AtCCS52A1         ------------------------SPDGQTIVTGAGDETLRFWNVFP-SPKSQNTD---- 469
AtCCS52A2         ------------------------SPDGQTIVTGAGDETLRFWNVFP-SPKSQSRE---- 461
PtCCS52A1         ------------------------SPDGQTIVTGAGDETLRFWNVFP-SPKSQNTE---- 444
PtCCS52A1_2       ------------------------SPDGQTIVTGAGDETLRFWSVFP-SPKSQNTD---- 481
OsCCS52A1         ------------------------SPDGQTIVTGAGDETLRFWNVFP-SPKSQSSDSL-- 509
PtCCS52B          ------------------------SPDGQTIVTGAGDETLRFWNVFP-SMKTQTPV---- 471
PtCCS52B_2        ------------------------SPDGQTIVTGAGDETLRFWNVFP-SMKTQTPV---- 471
AtCCS52B          ------------------------SPDGQTIVTGAGDETLRFWNVFP-SVKMQTPV---- 467
OsCCS52B          ------------------------SPDGQTIVTGAGDETLRFWNIFP-SMKTQAPV---- 464
                                          **:* *:.: *.**.**:*.:*

OsCDC20_2         -----------GSHTGMFNNSNHIHIR------------------------ 504
OsCDC20_3         -------------ASHTGMFNSFNHLR------------------------ 469
OsCDC20_1         VLVFQDNVRVGPWGTDCSYFQDLIALVVTMVTGSQKL-------------- 482
PtCDC20_4         ---------------------------------------------------
AtCDC20_3         -------------KKYPELFSHVNSLR------------------------ 444
AtCDC20_4         -------------KNYLELFSHVNSLRWCLVSFASGIDVLGKSNIIHSNCD 466
AtCDC20_5         -------------KKYTDPFAHVNHIR------------------------ 411
AtCDC20_6         -------------KKYTEPFAHVNHIR------------------------ 428
AtCDC20_1         -------------KAVAEPFSHVNRIR------------------------ 447
AtCDC20_2         -------------KAVSEPFSHVNRIR------------------------ 457
PtCDC20_1         -------------KANPEPFSHLNRLR------------------------ 455
PtCDC20_3         -------------KANPEPFSHLNRIR------------------------ 455
PtCDC20_2         ---------------------------------------------------
PtCDC20_5         ---------------------------------------------------
AtCCS52A1         -------------SEIGSSFFGRTTIR------------------------ 483
AtCCS52A2         -------------SEIGALSFGRTTIR------------------------ 475
PtCCS52A1         -------------SEIGASSLGRTTIR------------------------ 458
PtCCS52A1_2       -------------SEIGASSLGRTTIR------------------------ 495
OsCCS52A1         -------------SSIGATSFVRSYIR------------------------ 523
PtCCS52B          -------------KDTGLWSLGRTQIR------------------------ 485
PtCCS52B_2        -------------KDTGLWSLGRTQIR------------------------ 485
AtCCS52B          -------------KDTGLWSLGRTQIR------------------------ 481
OsCCS52B          -------------RDIGLWSFSRSHIR------------------------ 478
```

After EST support: Activators CDC20 and CCS52

```
AtCDC20_1        ---------------------MDAGLN-------------------RCPLQEHFLPRKNS 20
AtCDC20_2        ---------------------MDAGMNNTSSHYK---------TQARCPLQEHFLPRKPS 30
PtCDC20_1        ---------------------MDAGSLNSSSYMK---------AQSRFPLQEQFLHRKNS 30
PtCDC20_5        ------------------------------------------------------------
PtCDC20_2        ---------------------MDAGSINSSSSLK---------AQSRFPLQQQFLPRMNS 30
PtCDC20_3        ---------------------MDAGSMNTSSSLK---------AQSRFPLQQQFLPRTNS 30
AtCDC20_3        --------------------------MDSG-------------MRATCTVPEHFLPRKLS 21
AtCDC20_4        --------------------------MDS----------------DTCTVPDHFLPRKLS 18
AtCDC20_5        -------------------------MMNTSSHLK---------AQASCPLVEHFLRRKLS 26
AtCDC20_6        -------------------------MMKS---------------IALALCLTHHSPMVLS 20
PtCDC20_4        -----------------------MDSSSSSTTTR---------MFHPRSALRENPQRKKS 28
OsCDC20_1        ------------------MDA-GSHSISSEKSHG-LAPRPP---LQEAGSR-PYMPSLST 36
OsCDC20_2        ------------------MDA-GSHSISSEKSSRYVAPRQP---LQEAGSR-PYMPSLST 37
OsCDC20_3        ------------------MEAPGSGSVPTAKRRR-LVPRPPPVPLEVAGARGPYMPPLCI 41
AtCCS52A1        ------MEEEDPTASNVITNSNSSSMRNLSPAMNTPVVSLESR----INRLINANQSQSP 50
AtCCS52A2        ------MEEDESTTP----KKKSDSQLNLPPSMNRPTVSLESR----INRLIDSNHYHSP 46
PtCCS52A1        -MADPMISQSTQSKLNIAASTTPRHHHHLHLENPTPSPSSSKH----VNRLINSNHYISP 55
PtCCS52A1_2      -MADPTMSPPNQSQLNVAASTTQRRQHHLNLESLAPSPSSSEH----VNRLINSNHYISP 55
OsCCS52A         MDHHHHHLPPPPPRSPMENSASSKPPTPASTPSSRLAAAPSSR----VSSAAPHPSPSSS 56
PtCCS52B         ------MDSTPRRKSG---LNLPSGMNETSLRLETFSSSSSFRAVTCVSSPRAISSLSSP 51
PtCCS52B_2       ------MDSTPRRKSG---LNLPSGMNETSLRLETFSSSSSFRAVTCVSSPRAISSLSSP 51
AtCCS52B         ------MASPQSTKTG---LNLPAGMNQTSLRLETFSSS-----------FRGISSLSSP 40
OsCCS52B         ------MATDASPKPAPPRLNVPPAMAG-GLRLDPAVASP-------ARLLLDVPKTPSP 46

AtCDC20_1        KENL-------DRFIPNR-SAMNFDYAHFALTEERKG---------KDQS-ATVSSPSKE 62
AtCDC20_2        KENL-------DRFIPNR-SAMNFDYAHFALTEGRKG---------KDQT-AAVSSPSKE 72
PtCDC20_1        KDNL-------DRFIPNR-SAMDLDYAHYMLTQGRKGG--------KENPTATVNSPSRE 74
PtCDC20_5        ------------------------------------------------------------
PtCDC20_2        KENL-------DRFIPNR-SAMDMDYAHFMLTEGRKG---------KENP--TVNSPSRE 71
PtCDC20_3        KENL-------DRFIPNR-SAMDMDYARFMLTEGRKG---------KENP--TVNSPSRE 71
AtCDC20_3        KQNL-------DRFIPNR-SAKDFDFANYALTQGSKR------------NLDEVTSASRK 61
AtCDC20_4        KQNL-------DRFIPNR-SAMDFDFANYALTQGRKR------------NVDEITSASRK 58
AtCDC20_5        KENF-------DRFIPNRSAMMDFDFANYALTQGRKR------------NVDEVTSASRK 67
AtCDC20_6        --------------IVDYGDSQEKTID----SLWKKP------------HGSDLTWSG-- 48
PtCDC20_4        YENVSLQNYILDRFIPNR-SAMDMDFAHYMLTEGRKA---------------KESPPSQS 72
OsCDC20_1        ASRNPSAKCYGDRFIPDR-SAMDMDMAHYLLTEPKKD-----------KEN-AAASPSKE 83
OsCDC20_2        ASRNPSAKCYGDRFIPDR-SAMDMDMAHYLLTEPRKD-----------KEN-AAASPAKE 84
OsCDC20_3        KSKNPSAKCYGDRFIPDR-SAMDMDMAYFLLTEPKKE-----------KENTDMLSPAEE 89
AtCCS52A1        SPSSLSRSIYSDRFIPSR---SGSNFALFDLSPSPS---------------KDGKEDGAG 92
AtCCS52A2        S-----KPIYSDRFIPSR---SGSNFALFDLASSSPNK-------------KDGKEDGAG 85
PtCCS52A1        S-----RPIYSDRFIPCR---SSSNFALFNISFPQPSATAGISPG------CGGKEDNPS 101
PtCCS52A1_2      S-----RPIYSDRFIPCR---SSSNFALFNISLPSPSATAGSSPG------DGGKEDNPN 101
OsCCS52A         APTPASRTVYSDRFIPSR---AGSNLALFDLAPSPSHHDAAAAAASPGAPPPSGSTPASS 113
PtCCS52B         SKT----SSCSDRFIPCR---SSSRLHTFGLVEKG----------------SPVKEGGNE 88
PtCCS52B_2       SKT----SSCSDRFIPCR---SSSRLQTFGLIEKG----------------SPVKEGGNE 88
AtCCS52B         SK-----STCSDRFIPCR---SSSRLHAFDLQDKEPT--------------TPVKEGGNE 78
OsCCS52B         SK-----TTYSDRFIPCR---SSSRLHNFALLDRDRA--------------SPSSTTDDA 84

AtCDC20_1        AYRKQLAETMNLN----------------------------HTRILAFRNKPQAPVELLP 94
AtCDC20_2        AYRKQLAETMNLN----------------------------HTRILAFRNKPQAPVELLP 104
PtCDC20_1        AYRKQLAEALNLN----------------------------RTRILAFKNKPPTPVELIP 106
PtCDC20_5        ------------------------------------------------------------
PtCDC20_2        AYRKQLAESLNMN----------------------------RTRILAFKNKPPAPVELMP 103
PtCDC20_3        AYRKQLADSLNMN----------------------------RTRILAFKNKPPAPVELMP 103
AtCDC20_3        AYMTQLAVVMNQN----------------------------RTRILAFRNKPKS----LL 89
AtCDC20_4        AYMTQLAVVMNQN----------------------------RTRILAFRNKPKA----LL 86
AtCDC20_5        AYMTQLAEAMNQN----------------------------RTRILAFRNKPKA----LL 95
AtCDC20_6        --FSRVGVTEAYF----------------------------AVPVDHFLTNPPS----LR 74
PtCDC20_4        LYQKLLAEAFNMN----------------------------GRRILAFKNKPPTLVDPIP 104
OsCDC20_1        VYRRLLAEKLLNN----------------------------RTRILAFRNKPPEPENVS- 114
OsCDC20_2        AYRKLLAEKILNN----------------------------RTRILSFRNKPPEPESILT 116
OsCDC20_3        AYKRLLAEKLLNN----------------------------RSRILAFRNKPPEPEGIVQ 121
AtCCS52A1        SYATLLRAAMFGPETPEKRDIT-GFSSS--------------RNIFRFKTETH--RSLN- 134
AtCCS52A2        SYASLLKTALFGPVTPEKSDVVNGFSPS--------------GNIFRFKTETQ--RSLN- 128
PtCCS52A1        AYAALLRNALFGPQTPDKKDWGAGAAG---------------QNIFRYKMETR--QSLH- 143
PtCCS52A1_2      AYAALLRNALFGPQTPDKKDWGTGAAG---------------RNIFRYKTETR--QSMH- 143
OsCCS52A         PYCALLRAALFGPTTPDRVASSASACSSSSSAGASPVGSPATGNIFRFKAEVP--RNAKR 171
PtCCS52B         AYARLLKSELFGSDFGS-FSSPAGGQGGLSS---------PNKNMLRFKTDHS--GPNSP 136
PtCCS52B_2       AYARLLKSELFGSDFGS-FSSPAGGQGGLGS---------PSKNMLRFKTDHS--GPNSP 136
AtCCS52B         AYSRLLKSELFGSDFASPLLSPAGGQGSASSP------MSPCTNMLRFKTDRSNSSPSSP 132
OsCCS52B         PYSRLLRAEIFGPDSPS----PAPSS--------------PNTNLFRFKTDHP--SPKSP 124
```

```
AtCDC20_1        SNHS-----------ASLHQQPKSVKPRRYIPQTSERTLDAPDIVDDFYLNLLDWGSANV 143
AtCDC20_2        SNHS-----------ASLHQQPKSVKPRRYIPQTSERTLDAPDIVDDFYLNLLDWGSANV 153
PtCDC20_1        RDHLS----------SSLHYQAKPTKPRRYIPQTSERTLDAPDLVDDFYLNLLDWGSKNV 156
PtCDC20_5        ------------------------------------------------------------
PtCDC20_2        QDH------------SHHHHQPKTAKPRRHIPQTSERTLDAPDLVDDFYLNLLDWGSSNV 151
PtCDC20_3        QDH------------SHHHHQPKTAKPRRHIPQTSERTLDAPDLVDDFYLNLLDWGSSNV 151
AtCDC20_3        STNHS----------DSPHQNPKPVKPRRYIPQNSERVLDAPGLRDDFSLNLLDWGSANV 139
AtCDC20_4        SSNHS----------DSPHQNPKSVKPRRYIPQNSERVLDAPGLMDDFYLNLLDWGSANV 136
AtCDC20_5        SSNHS----------DPPHQQPISVKPRRYIPQNSERVLDAPGIADDFYLNLLDWGSSNV 145
AtCDC20_6        LS-QT----------IYCRRGSSYVETEKVEEEDRDDFLKQVWFLTDNLVFFFVDIEEYI 123
PtCDC20_4        LFS------------SSSVHSSKPVKPQRHIPQRPEMTLDAPDIVDDFYLNLLDWGNNNV 152
OsCDC20_1        --AAD----------TASTHQAKPAKQRRYIPQSAERTLDAPDLVDDYYLNLLDWGSKNV 162
OsCDC20_2        E-LRA----------DAASIQAKPAKQRRYIPQSAERTLDAPELVDDYYLNLLDWGSSNV 165
OsCDC20_3        QLLYE----------TLTSSQTKPARKCRHIPQSSERTLDAPGIVDDFYLNILDWGCKNV 171
AtCCS52A1        -SFSPFGVD----DDSPGVSHSGPVKAPRKVPRSPYKVLDAPALQDDFYLNLVDWSAQNV 189
AtCCS52A2        -LYPPFDSD----VVS-GVSPS-PVKSPRKILRSPYKVLDAPALQDDFYLNLVDWSAQNV 181
PtCCS52A1        -SLSPFGFD----DMSDLGVSNVAIKTPRKVSRSPYKVLDAPALQDDFYLNLVDWSSHNV 198
PtCCS52A1_2      -SLSPFGFD----GLSGPGVSNVAIKAPRKVSRSPYKVLDAPALHDDFYLNLVDWSSHNV 198
OsCCS52A         ALFSDGDDE----GVLFPGVFTTRGTGPRKIPRSPYKVLDAPALQDDFYLNLVDWSSHNI 227
PtCCS52B         FSPSILGHD-----SGISSESSTPPKPPRKVPKTPHKVLDAPSLQDDFYLNLVDWSSQNV 191
PtCCS52B_2       YSPSILGHD-----SGISSESSTTPKPPRKVPKTPHKVLDAPSLQDDFYLNLVDWSSQNV 191
AtCCS52B         FSPSILGND-----NGHSSDSSPPPKPPRKVPKTPHKVLDAPSLQDDFYLNVVDWSSQNV 187
OsCCS52B         FAASAAATAGHYDCTAGSAESSTPRKPPRKVPKTPHKVLDAPSLQDDFYLNLVDWSSQNT 184


AtCDC20_1        LAIALDHTVYLWDASTGSTSELVTIDEEKGPVTSINWAPDGRHVAVGLNNSEVQLWDSAS 203
AtCDC20_2        LAIALDHTVYLWDASTGSTSELVTIDEEKGPVTSINWAPDGRHVAVGLNNSEVQLWDSAS 213
PtCDC20_1        LAIALENTVYLWDASNGSTSELVTVGDEVGPVTSVNWAPDGLHLAIGLNNSNVQLWDSAS 216
PtCDC20_5        ------------------------------------------------------------
PtCDC20_2        LAIALGSTVYLWDASDGSTSELVTVDDEDGPITSVNWAPDGRHIAIGLNNSHIQLWDSAS 211
PtCDC20_3        LAIALGSTVYLWDASDGSTSELVTVDDEDGPVTSVNWAPDGRHIAIGLNNSHIQLWDSAS 211
AtCDC20_3        LAIALGDTVYLWDASSGSTSELVTIDEDKGPVTSINWTQDGLDLAVGLDNSEVQLWDCVS 199
AtCDC20_4        LAIALGDTVYLWDASSGSTSELVTIDEDKGPVTSINWTQDGLDLAVGLDNSEVQLWDFVS 196
AtCDC20_5        LAIALGDTVYLWDASSGSTYKLVTIDEEEGPVTSINWTQDGLDLAIGLDNSEVQLWDCVS 205
AtCDC20_6        VIEQLGDTVYLWDASSCYTSKLVTIDDENGPVTSINWTQDGLDLAVGLDNSEVQVWDCVS 183
PtCDC20_4        LAIALGTTVYLWNASNSSISEVVTVDEEDGPVTSISWAPDGRHLAVGLDNSNVQLWDSAT 212
OsCDC20_1        LSIALGDTVYLWDASSGSTSELVTVDEDSGPITSVSWAPDGQHVAVGLNSSDIQLWDTSS 222
OsCDC20_2        LSIALGNSVYLWDATNSSTSELVTVDEDNGPVTSVSWAPDGRHIAVGLNSSDVQLWDTSS 225
OsCDC20_3        MSIALGNTLYLWNSADGSIMDLVTIDEDDGPITSVSWSCDGQWIAVGLNSSDIQLWDTSS 231
AtCCS52A1        LAVGLGNCVYLWNACSSKVTKLCDLGAEDS-VCSVGWALRGTHLAVGTSTGKVQIWDASR 248
AtCCS52A2        LAVGLGNCVYLWNACSSKVTKLCDLGVDET-VCSVGWALRGTHLAIGTSSGTVQIWDVLR 240
PtCCS52A1        LAVGLGNCVYLWNACSSKVTKLCDLGNDDG-VCSVGWAHRGTHLAIGTSNGKVQIWDASR 257
PtCCS52A1_2      LAVGLGTCVYLWNACSSKVTKLCDLGNDDG-VCSVGWAHRGTHLAVGTSNGKVQIWDASR 257
OsCCS52A         LAVGLGNCVYLWNACSSKVTKLCDLGVDDN-VCSVGWAQRGTHLAVGTNQGKVQVWDATR 286
PtCCS52B         LAVGLGTCVYLWTASNSKVTRLCDLGPNDS-VCSVQWTREGSYISVGTHLGQVQVWDGTQ 250
PtCCS52B_2       LAVGLGTCVYLWTASNSKVTRLCDLGPNDS-VCSLQWTREGSYISIGTHLGQVQVWDGTQ 250
AtCCS52B         LAVGLGTCVYLWTASNSKVTKLCDLGPNDS-VCSVQWTREGSYISIGTSHGQVQVWDGTQ 246
OsCCS52B         LAVGLGNCVYLWSASNCKVTKLCDLGPRDS-VCAVHWTREGSYLAIGTSLGDVQIWDSSR 243


AtCDC20_1        NRQLRTLKGGHQSRVGSLAWNNHILTTGGMDGLIINNDVRIRSPIVETYRGHTQEVCGLK 263
AtCDC20_2        NRQLRTLKGGHQSRVGSLAWNNHILTTGGMDGLIINNDVRIRSPIVETYRGHTQEVCGLK 273
PtCDC20_1        CKQLRNLRGCHRSRVGSMAWNNHILTTGGMDGKIINNDVRIRSHIVETYRGHQQEVCGLK 276
PtCDC20_5        ------------------------------------------------------------
PtCDC20_2        NRQLRTLKGGHRSRVGSLAWNNHILTTGGMDGQIINNDVRIRSHIVETYRGHTQEVCGLK 271
PtCDC20_3        NRQLRTLKGGHRSRVGSMAWNNHILTTGGMDGQIINNDVRIRSHIVETYRGHTQEVCGLK 271
AtCDC20_3        NRQVRTLRGGHESRVGSLAWDNHILTTGGMDGKIVNNDVRIRSSIVETYLGHTEEVCGLK 259
AtCDC20_4        NRQVRTLIGGHESRVGSLAWNNHILTTGGMDGKIVNNDVRIRSSIVGTYLGHTEEVCGLK 256
AtCDC20_5        NRQVRTLRGGHESRVGSLAWNNHILTTGGMDGKIVNNDVRIRSSIVETYLGHTEEVCGLK 265
AtCDC20_6        NRHVRTLRGGHESRVGSLAWNNHILTTGGMDGKIVNNDVRIRSSIIGTYVGHTEEVCGLK 243
PtCDC20_4        NQMLRTLRGGHRLRVTSLAWNHHLLTTGGKDAKVINNDVRIREHIVESYEGHRQEVCGLK 272
OsCDC20_1        NRLLRTLRGVHESRVGSLAWNNNILTTGGMDGNIVNNDVRIRNHVVQTYQGHSQEVCGLK 282
OsCDC20_2        NRLLRTMRGVHDSRVGSLAWNNNILTTGGMDGKIVNNDVRIRNHVVQTYQGHQQEVCGLK 285
OsCDC20_3        NRMLRTLHGVHQSRVGSLAWNKNILTTGGMDGNIVNNDVRMRSHVVHIYRGHEDEVCGLR 291
AtCCS52A1        CKRTRTMEG-HRLRVGALAWGSSVLSSGSRDKSILQRDIRCQEDHVSKLAGHKSEVCGLK 307
AtCCS52A2        CKNIRTMEG-HRLRVGALAWSSSVLSSGSRDKSILQRDIRTQEDHVSKLKGHKSEICGLK 299
PtCCS52A1        CKRIRTMEG-HRLRVGALAWSSSMLSSGSRDKSILQRDIRAREDFVSKLSGHKSEVCGLK 316
PtCCS52A1_2      CKRIRIMEG-HRLRVGALAWSSSMLSSGSRDKSILQRDIRAQEDFVSKLSGHKSEVCGLK 316
OsCCS52A         CKRIRTMES-HRMRVGALAWNSSLLSSGSRDKSILHHDIRAQDDYISRLAGHKSEVCGLK 345
PtCCS52B         CKRVRTMGG-HQTRTGVLAWNSRTLASGSRDRHILQHDLRISSDYVSKLIGHKSEVCGLK 309
PtCCS52B_2       CKRVRTMGG-HQTRTSVLAWNSRTLASGSRDRNILQHDLRVSSDHVSKLIGHKSEVCGLK 309
AtCCS52B         CKRVRTMGG-HQTRTGVLAWNSRILSSGSRDRNILQHDIRVQSDFVSKLVGHKSEVCGLK 305
OsCCS52B         CKRIRNMGG-HQTRTGVLAWSSRILSSGSRDKNILQHDIRVPSDYISKFSGHRSEVCGLK 302
```

```
AtCDC20_1      WSGSGQQLASGGNDNVVHIWD-RSVASSNSTT---QWLHRLEEHTSAVKALAWCPFQANL 319
AtCDC20_2      WSGSGQQLASGGNDNVVHIWD-RSVASSNSTT---QWLHRLEEHTSAVKALAWCPFQANL 329
PtCDC20_1      WSASGQQLASGGNDNIIHIWD-RSVASSNSAT---QWFHRLEEHTSAVKALAWCPFQGNL 332
PtCDC20_5      -----------------HIWD-RSVASSNSAT---QWFHRLEEHTSAVKALAWCPFQGNL 39
PtCDC20_2      WSASGQQLASGGNDNLIHIWD-RSTALSNSAT---QWLHRLEDHTSAVKALAWCPFQGNL 327
PtCDC20_3      WSASGQQLASGGNDNLIHIWD-RSTALSNSAT---QWLHRLEDHTSAVKALAWCPFQGNL 327
AtCDC20_3      WSESGNKQASGGNDNVVHIWD-RSLASSKQTR---QWLHRFEEHTAAVRALAWCPFQASL 315
AtCDC20_4      WSESGKKLASGGNYNVVHIWDHRSVASSKPTR---QWLHRFEEHTAAVRALAWCPFQATL 313
AtCDC20_5      WSESGKKLASGGNDNVVHIWDHRSVASSNPTR---QWLHRFEEHTAAVRALAWCPFQASL 322
AtCDC20_6      WSESGKKLASGGNDNVVHIWD-RSLASSNPTR---QWLHRFEEHTAAVRALAWCPFQASL 299
PtCDC20_4      WSASGQQLASGGNDNLLFIWD-RFMASSNSPR---HWLHKLEDHTAAVKALAWCPFQSNL 328
OsCDC20_1      WSGSGQQLASGGNDNLLHIWD-VSMASSVPSAGRNQWLHRLEDHTAAVKALAWCPFQSNL 341
OsCDC20_2      WSGSGQQLASGGNDNLLHIWD-VSMASSMPSAGRTQWLHRLEDHLAAVKALAWCPFQSNL 344
OsCDC20_3      WSGSGQQLASGGNDNLVHIWD-VSMASSNLSLGHNRWLHRFGDHLAAVKALAWCPFQSNL 350
AtCCS52A1      WSYDNRELASGGNDNRLFVWNQHSTQP----------VLKYSEHTAAVKAIAWSPHVHGL 357
AtCCS52A2      WSSDNRELASGGNDNKLFVWNQHSTQP----------VLRFCEHAAAVKAIAWSPHHFGL 349
PtCCS52A1      WSYDNRELASGGNDNRLFVWNQHSSQP----------VLKYCDHTAAVKAIAWSPHLHGL 366
PtCCS52A1_2    WSYDNRELASGGNDNRLFVWNQHSTQP----------VLKYCEHTAAVKAIAWSPHLHGL 366
OsCCS52A       WSYDNRQLASGGNDNRLYVWNQHSAHP----------VLKYTEHTAAVKAIAWSPHLHGL 395
PtCCS52B       WSHDDRELASGGNDNQLLVWNQHSQLP----------ILKLTEHTAAVKAIAWSPHQSGL 359
PtCCS52B_2     WSHDDRELASGGNDNQLLVWNQHSQLP----------VLTLTEHTAAVKAIAWSPHQSGL 359
AtCCS52B       WSHDDRELASGGNDNQLLVWNNHSQQP----------ILKLTEHTAAVKAITWSPHQSSL 355
OsCCS52B       WSHDDRELASGGNDNQLLVWNQRSQQP----------ILRLTEHTAAVKAIAWSPHQQGL 352
                                :*:                .   :* :**:*::*.*.   *

AtCDC20_1      LATGGGGGDRTIKFWNTHTGACLNSVDTGSQVCSLLWSKNERELLSSHGFTQNQLTLWKY 379
AtCDC20_2      LATGGGGGDRTIKFWNTHTGACLNSVDTGSQVCSLLWSKNERELLSSHGFTQNQLTLWKY 389
PtCDC20_1      LASGGGGGDRSIKFWNTHTGACLNSIDTGSQVCALLWNKNERELLSSHGFTQNQLVLWKY 392
PtCDC20_5      LASGGGGGDRSIKFWNTHTGACLNSIDTGSQVCALLWNKNERELLSSHGFTQNQLVLWKY 99
PtCDC20_2      LASGGGGGDKSIKFWNTHTGACLNSIDTGSQVCSLLWNKNERELLSSHGFTQNQLTVWKY 387
PtCDC20_3      LASGGGGGDKSIKFWNTHTGACLNSIDTGSQVCSLLWNKNERELLSSHGFTQNQLTVWKY 387
AtCDC20_3      LATGGGVGDGKIKFWNTHTGACLNSVETGSQVCSLLWSQSERELLSSHGFTQNQLTLWKY 375
AtCDC20_4      LATGGGVGDGKIKFWNTHTGACLNSVETGSQVCSLLWSQRERELLSSHGFTQNQLTLWKY 373
AtCDC20_5      LATGGGVGDGKIKFWNTHTGACLNSVETGSQVCSLLWSKSERELLSSHGFTQNQLTLWKY 382
AtCDC20_6      LATGGGVGDGKINFWNTHTGACLNSVETGSQVCSLLWSKSERELLSAHGFTQNQLTLWKY 359
PtCDC20_4      LASGGGGNDRHIKFWNTQTGTCLNSVDTGSQVCALQWNKHERELLSSHGFTENQLILWKY 388
OsCDC20_1      LATGGGGSDRCIKFWNTHTGACLNSVDTGSQVCALLWNKNERELLSSHGFTQNQLTLWKY 401
OsCDC20_2      LASGGGGSDRCIKFWNTHTGACLNSIDTGSQVCSLVWNKNERELLSSHGFAQNQLTLWKY 404
OsCDC20_3      LASGGGGDDRCIRFWNTHTGLCLNSVDTGSQVCGLLWNKNEKELLSAHGYVQNSLALWKY 410
AtCCS52A1      LASGGGTADRCIRFWNTTTNTHLSSIDTCSQVCNLAWSKNVNELVSTHGYSQNQIIVWKY 417
AtCCS52A2      LASGGGTADRCIRFWNTTTNTHLNCVDTNSQVCNLVWSKNVNELVSTHGYSQNQIIVWKY 409
PtCCS52A1      LASGGGTADRCIRFWNTTTNSHLSCIDTGSQVCNLVWSKNVNELVSTHGYSQNQIILWRY 426
PtCCS52A1_2    LASGGGTADRCIRFWNTTTNSHLSCMDTGSQVCNLVWSKNVNELVSTHGYSQNQIIVWRY 426
OsCCS52A       LASGGGTADRCIRFWNTTTNMHLNCVDTGSQVCNLVWSKNVNELVSTHGYSQNQIIVWRY 455
PtCCS52B       LASGGGTADRCIRFWNTTNGHQLNYVDTGSQVCNLAWSKNVNELVSTHGYSQNQIMVWKY 419
PtCCS52B_2     LASGGGTADRCIRFWNTTNGHQLNHVDTGSQVCNLAWSKNVNELVSTHGYSQNQIMVWKY 419
AtCCS52B       LASGGGTADRCIRFWNTTNGNQLNSIDTGSQVCNLAWSKNVNEIVSTHGYSQNQIMLWKY 415
OsCCS52B       LASGGGTADRCIRFWNTVNGNMLNSVDTGSQVCNLAWCKNVNELVSTHGYSQNQIMVWKY 412
               **:***  *  *.****  ..  *.  ::* **** * * :  .*::*:**: :*.: :*:*

AtCDC20_1      PSMVKMAELTGHTSRVLYMAQSPDGCTVASAAGDETLRFWNVFG-VPETAKKAAPKAVAE 438
AtCDC20_2      PSMVKMAELTGHTSRVLYMAQSPDGCTVASAAGDETLRFWNVFG-VPETAKKAAPKAVSE 448
PtCDC20_1      PSMLKMAELTGHTSRVLYMAQSPDGCTVATAAGDETLRFWNVFG-VPEVA-KAAPKANPE 450
PtCDC20_5      PSMLKMAELTGHTSRVLYMAQSPDGCTVATAAGDETLRFWNVFG-VPEVA-KAAPKANPE 157
PtCDC20_2      PSMVKMAELTGHTSRVLYMAQSPDGCTVATAAGDETLRFWNVFG-VPEVAAKAAPKANPE 446
PtCDC20_3      PSMVKMAELTGHTSRVLYMAQSPDGCTVATAAGDETLRFWNVFG-VPEIAAKAAPKANPE 446
AtCDC20_3      PSMSKMAELNGHTSRVLFMAQSPNGCTVASAAGDENLRLWNVFGEPPKTTKKAASKKYPE 435
AtCDC20_4      PSMSKMAELNGHTSRVLFMAQSPNGCTVASAAGDENLRLWNVFGEPPKTTKKAASKNYLE 433
AtCDC20_5      PSMVKMAELNGHTSRVLFMAQSPDGCTVASAAGDETLRLWNVFGEPPKTTKKAASKKYTD 442
AtCDC20_6      PSMVKMAELNGHTSRVLFMAQSPDGCTVASAAGDETLRLWNVFGEPPKTTKKAASKKYTE 419
PtCDC20_4      PSMVKMAELSGHTSPVLFMTQSPDGYTVASAAGDETLRFWNVFG--NPKAAKPAPKAIAE 446
OsCDC20_1      PSMVKMAELTGHTSRVLFMAQSPDGCTVASAAADETLRFWNVFG-SPEAPKPAAKASHTG 460
OsCDC20_2      PSMVKMAELTGHTSRVLFTAQSPDGLTVASAAADETLRFWNVFG-APEAPKTATKGSHTG 463
OsCDC20_3      PSMVKLAELEDHTARVLCLAQSPDGFTVASVAADETLRLWKIFE-TSEDAKPVFKTVNTG 469
AtCCS52A1      PTMSKIATLTGHTYRVLYLAVSPDGQTIVTGAGDETLRFWNVFP-SPKSQNTD--SEIGS 474
AtCCS52A2      PTMSKLATLTGHSYRVLYLAVSPDGQTIVTGAGDETLRFWNVFP-SPKSQSRE--SEIGA 466
PtCCS52A1      PTMSKLATLTGHTFRVLYLAISPDGQTIVTGAGDETLRFWSVFP-SPKSQNTD--SEIGA 483
PtCCS52A1_2    PTMSKLATLTGHTYRVLYLAISPDGQTIVTGAGDETLRFWNVFP-SPKSQNTE--SEIGA 483
OsCCS52A       PTMSKLATLTGHTYRVLYLAISPDGQTIVTGAGDETLRFWNVFP-SPKSQSSDSLSSIGA 514
PtCCS52B       PSLSKVATLVGHSLRVLYLAMSPDGQTIVTGAGDETLRFWNVFP-SMKTQTPV--KDTGL 476
PtCCS52B_2     PSLSKVATLVGHSMRVLYLAMSPDGQTIVTGAGDETLRFWNVFP-SMKTQTPV--KDTGL 476
AtCCS52B       PSMSKVATLTGHSMRVLYLATSPDGQTIVTGAGDETLRFWNVFP-SVKMQTPV--KDTGL 472
OsCCS52B       PSMSKVATLTGHTLRVLYLAMSPDGQTIVTGAGDETLRFWNIFP-SMKTQAPV--RDIGL 469
               *:: *:* * .*:  **  : **:* *:.: *.**.**:*.:*
```

```
AtCDC20_1        PFSHVNR--IR 447
AtCDC20_2        PFSHVNR--IR 457
PtCDC20_1        PFSRFNR--IR 459
PtCDC20_5        PFSRFNR--IR 166
PtCDC20_2        PFSHLNR--LR 455
PtCDC20_3        PFSHLNR--IR 455
AtCDC20_3        LFSHVNS--LR 444
AtCDC20_4        LFSHVNS--LR 442
AtCDC20_5        PFAHVNH--IR 451
AtCDC20_6        PFAHVNH--IR 428
PtCDC20_4        PFANVSH--FR 455
OsCDC20_1        MFNSFNH--LR 469
OsCDC20_2        MFNNSNHIHIR 474
OsCDC20_3        MFNSFSH--IR 478
AtCCS52A1        SFFGRTT--IR 483
AtCCS52A2        LSFGRTT--IR 475
PtCCS52A1        SSLGRTT--IR 492
PtCCS52A1_2      SSLGRTT--IR 492
OsCCS52A         TSFVRSY--IR 523
PtCCS52B         WSLGRTQ--IR 485
PtCCS52B_2       WSLGRTQ--IR 485
AtCCS52B         WSLGRTQ--IR 481
OsCCS52B         WSFSRSH--IR 478
                      .   :*
```
