# Supplementary material for: Genomic evolution and complexity of the Anaphase-promoting Complex (APC) in land plants
Source: BMC Plant Biol. 2010 Nov 18;10:254. doi: 10.1186/1471-2229-10-254 (PMC3095333; doi:10.1186/1471-2229-10-254)
Supplement: Additional file 8 — APC subunits in plant genomes and red algae. Cr, Chlamydomonas reinhardtii; Vc, Volvox carteri; Csp, Chlorella sp; Msp, Micromonas sp; Osp, Ostreococcus sp; Cm, Cyanidioschyzon merolae; Pp, Physcomitrella patens; Sm, Selaginella moellendorffii; Bd, Brachypodium distachyon; Sb, Sorghum bicolor; Mt, Medicago truncatula; Vv, Vitis vinifera; Cp, Carica papaya; At, Arabidopsis thaliana; Os, Oryza sativa; Pt, Populus trichocarpa; Abbreviations: NI, not identified. [file 1471-2229-10-254-S8.PDF]

Additional file 8. APC subunits in plant genomes and red algae.

Table S1: APC subunits in plant genomes and red algae.

	APC Subunits											
**Plants**	***APC1***	***APC2***	***CDC27***	***APC4***	***APC5***	***CDC16***	***APC7***	***CDC23***	***APC10***	***APC11***	***CDC26***	***APC13***
**Cr**	1	1	1	1	1	1	NI	1	1	1	1	1
**Vc**	1	1	1	1	1	1	NI	1	1	1	NI	NI
**Csp**	1	1	1	1	1	1	NI	1	1	1	NI	NI
**Msp**	1	1	1	1	1	1	1	1	1	1	NI	NI
**Osp**	1	1	1	1	1	1	1	1	1	1	NI	NI
**Cm**	1	1	1	1	NI	1	NI	1	1	1	NI	NI
**Pp**	1	1	2	1	1	1	1	1	1	1	1	1
**Sm**	1	1	1	1	1	1	1	1	1	1	1	1
**Bd**	1	1	1	1	1	1	1	1	1	2	1	1
**Sb**	1	1	1	1	1	1	1	1	1	1	1	1
**Mt**	1	1	1	1	1	1	1	1	1	1	1	1
**Vv**	1	1	1	1	1	1	1	2	1	2	1	2
**Cp**	1	1	1	1	1	1	1	1	1	1	1	1
**At**	1	1	2	1	1	1	1	1	1	1	1	1
**Os**	1	1	1	1	1	1	1	2	1	2	1	1
**Pt**	1	1	2	1	1	1	1	1	1	1	1	2

Cr, *Chlamydomonas reinhardtii*; Vc, *Volvox carteri;* Csp, *Chlorella sp;* Msp, *Micromonas sp;* Osp, *Ostreococcus sp;* Cm, *Cyanidioschyzon merolae;* Pp, *Physcomitrella patens;* Sm, *Selaginella moellendorffii; Bd, Brachypodium distachyon;* Sb, *Sorghum bicolor*; Mt, *Medicago truncatula;* Vv, *Vitis vinifera*; Cp, *Carica papaya*; At, *Arabidopsis thaliana*; Os, *Oryza sativa*; Pt, *Populus trichocarpa*; Abbreviations: NI, not identified.
