# Supplementary material for: Genomic evolution and complexity of the Anaphase-promoting Complex (APC) in land plants
Source: BMC Plant Biol. 2010 Nov 18;10:254. doi: 10.1186/1471-2229-10-254 (PMC3095333; doi:10.1186/1471-2229-10-254)

Additional file 9: **Chromosomal locations of rice and poplar APC subunits and activators**. Chromosome numbers are indicated at the bottom of each chromosome. Paralogs are linked by dashed lines. A, chromosomal positions of genes in rice. B, chromosomal positions of genes in poplar. Seven poplar genes were assigned to scaffolds.

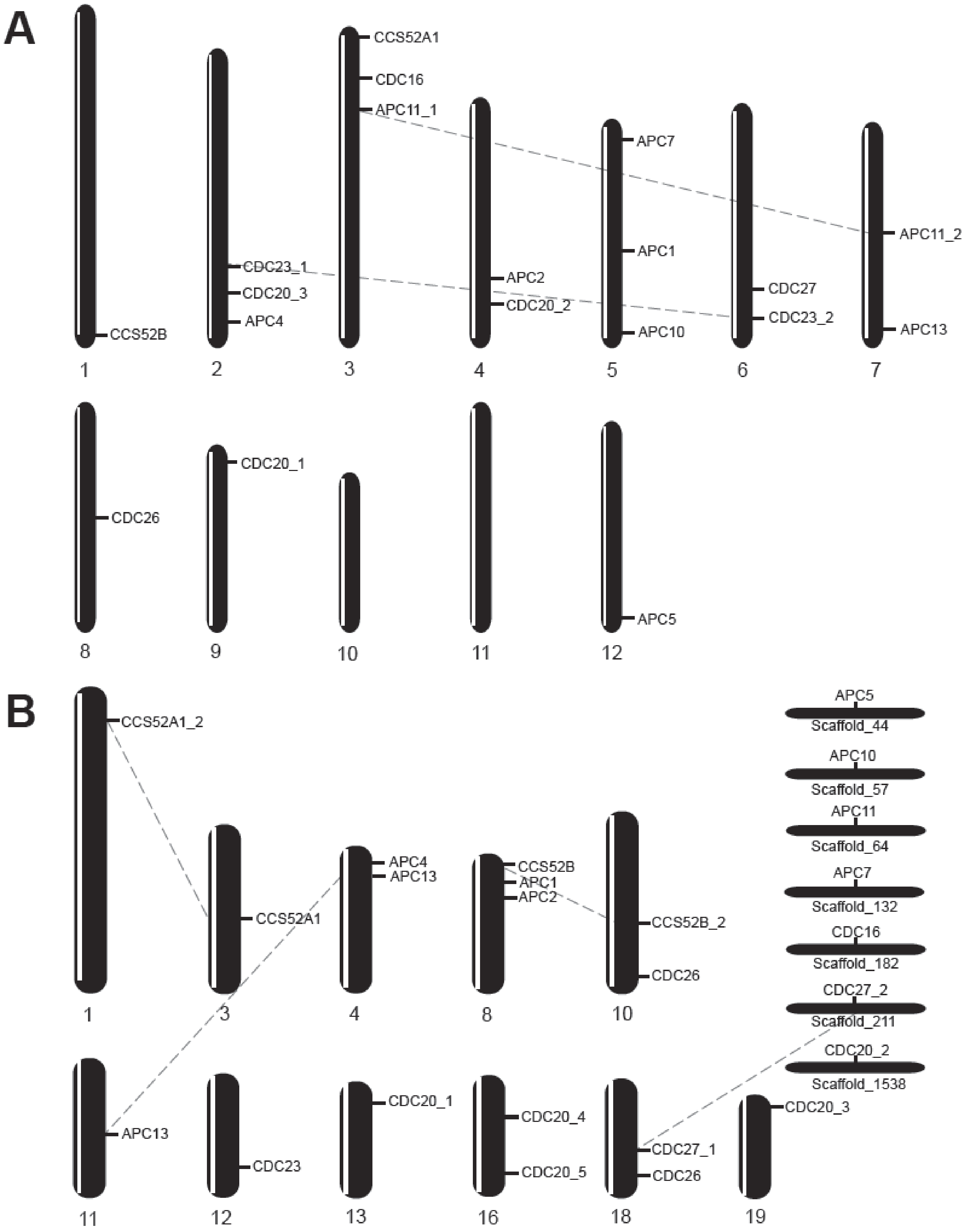

Supplement: Additional file 9 — Chromosomal locations of rice and poplar APC subunits and activators. Chromosome numbers are indicated at the bottom of each chromosome. Paralogs are linked by dashed lines. A, chromosomal positions of genes in rice. B, chromosomal positions of genes in poplar. Seven poplar genes were assigned to scaffolds. [file 1471-2229-10-254-S9.PDF]
