# Supplementary material for: Genomic evolution and complexity of the Anaphase-promoting Complex (APC) in land plants
Source: BMC Plant Biol. 2010 Nov 18;10:254. doi: 10.1186/1471-2229-10-254 (PMC3095333; doi:10.1186/1471-2229-10-254)
Supplement: Additional file 10 — Neighbor-joining tree inferred from Poisson-corrected evolutionary distances for genes involved in the cell cycle and multiple sequence alignments of plant TPR subunits proteins. The TPR subunits (A) and activator gene family (B). The abbreviations of species names are as follows: At, Arabidopsis thaliana; Pt, Populus trichocarpa; Os, Oryza sativa; Vv, Vitis vinifera; Sb, Sorghum bicolor; Pp, Physcomitrella patens; Sm, Selaginella moellendorffii; Msp, Micromonas sp; Osp, Ostreococcus sp; Csp, Chlorella sp; Vc, Volvox carteri; Cm, Cyanidioschyzon merolae; Zm, Zea mays; So, Saccharum officinarum; Mt, Medicago truncatula. [file 1471-2229-10-254-S10.PDF]

Additional file 10: Neighbor-joining tree inferred from Poisson-corrected evolutionary distances for genes involved in the cell cycle and multiple sequence alignments of plant TPR subunits proteins. The TPR subunits (A) and activator gene family (B). The abbreviations of species names are as follows: At, *Arabidopsis thaliana*; Pt, *Populus trichocarpa*; Os, *Oryza sativa*; Vv, *Vitis vinifera*; Sb, *Sorghum bicolor*; Pp, *Physcomitrella patens*; Sm, *Selaginella moellendorffii*; Msp, *Micromonas sp*; Osp, *Ostreococcus sp*; Csp, *Chlorella sp*; Vc, *Volvox carteri*; Cm, *Cyanidioschyzon merolae*; Zm, *Zea mays*; So, *Saccharum officinarum*; Mt, *Medicago truncatula*.

A

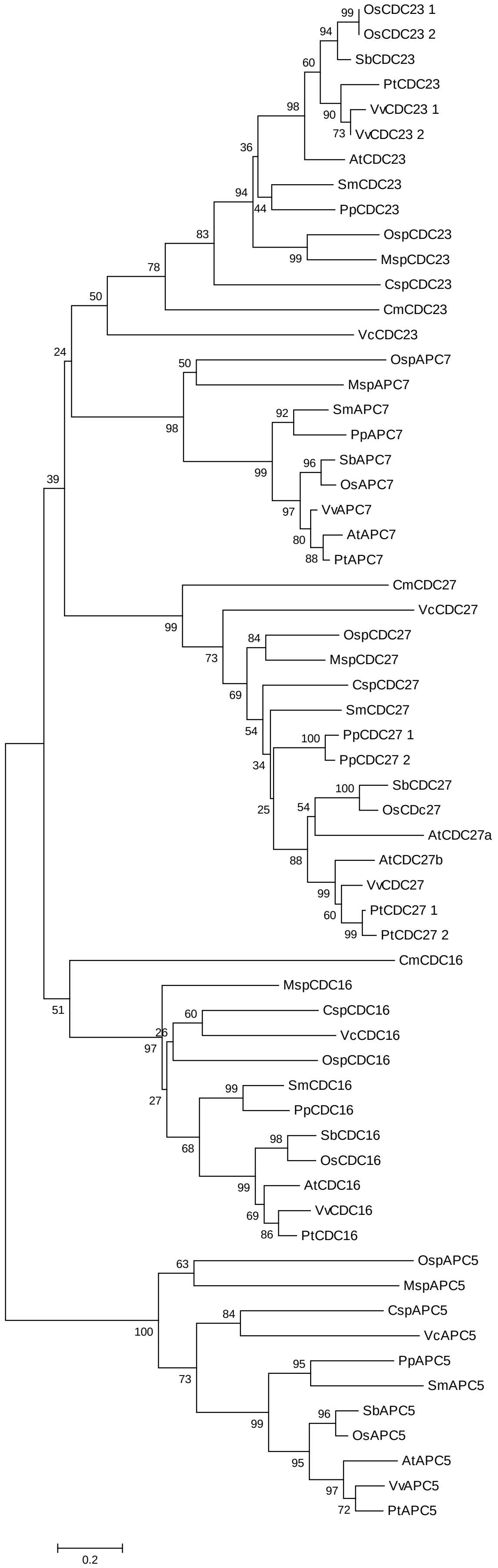

B

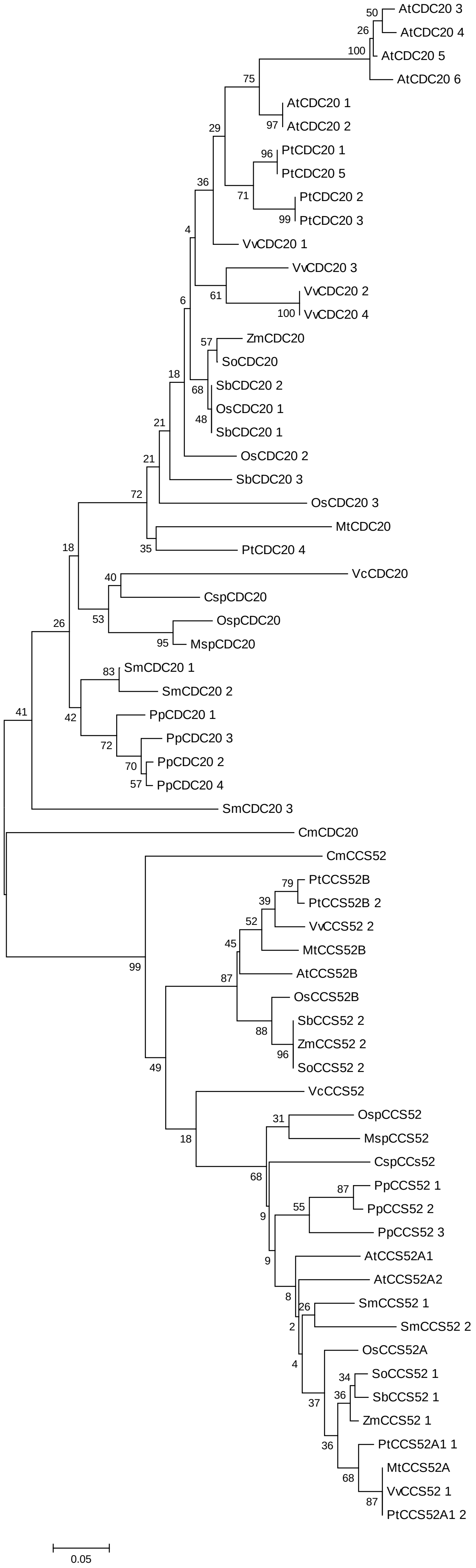
AtCDC20 3
AtCDC20 4
AtCDC20 5
AtCDC20 6
AtCDC20 1
AtCDC20 2
PtCDC20 1
PtCDC20 5
PtCDC20 2
PtCDC20 3
VvCDC20 1
VvCDC20 3
VvCDC20 2
VvCDC20 4
ZmCDC20
SoCDC20
SbCDC20 2
OsCDC20 1
SbCDC20 1
OsCDC20 2
SbCDC20 3
OsCDC20 3
MtCDC20
PtCDC20 4
VcCDC20
CspCDC20
OspCDC20
MspCDC20
SmCDC20 1
SmCDC20 2
PpCDC20 1
PpCDC20 3
PpCDC20 2
PpCDC20 4
SmCDC20 3
CmCDC20
CmCCS52
PtCCS52B
PtCCS52B 2
VvCCS52 2
MtCCS52B
AtCCS52B
OsCCS52B
SbCCS52 2
ZmCCS52 2
SoCCS52 2
VcCCS52
OspCCS52
MspCCS52
CspCCs52
PpCCS52 1
PpCCS52 2
PpCCS52 3
AtCCS52A1
AtCCS52A2
SmCCS52 1
SmCCS52 2
OsCCS52A
SoCCS52 1
SbCCS52 1
ZmCCS52 1
PtCCS52A1 1
MtCCS52A
VvCCS52 1
PtCCS52A1 2
0.05

```
OsAPC5          MNVFAGVGGGAAGKEAGTVGGGALLELTPHKLALCHLVQVFAP----------------- 43
SbAPC5          MSLFAGVGGGAADAASGGGTGRALLELTPHKMAVCHLVQVFAP----------------- 43
PtAPC5          -----------------------AFALTPHKVSVCLLLQTYAL----------------- 20
VvAPC5          ------------------------------------------------------------
AtAPC5          --------------MAGLTRTAGAFAVTPHKISVCILLQIYAP----------------- 29
PpAPC5          -------------------------FVLTSHKVAMCMLLQAYAS---------------- 19
SmAPC5          --------------------MAAGFVLTPHKLSMCVLLQLYAA----------------- 23
CspAPC5         -----------------------MEAVKAADVALCILLRSYLC----------------- 20
VcAPC5          ------------------------------------------------------------
MspAPC5         --------------------MAQVYSPTPHRVALCALARYLVHSHARDERAAERLGVSPE 40
OspAPC5         --------------MERSGKTTEGFVVAARRAWARCATEFLLD----------------- 29

OsAPC5          -------PPQAGVSAPALPFPFESVAHHNRLGLFLFALT---RSCEDFREPPLEELLRQL 93
SbAPC5          -------PAQAGG-DVVPPFPFESLAHHNRLGLFLFTLT---RSCEDFLEPPLEEFLRQL 92
PtAPC5          -------PAQTTP-----PFPFSSVSQHNRLGLYLLALTKMLQSYDDILEPKLEELLNQL 68
VvAPC5          ------------------------------------------------------------
AtAPC5          -------SAQMSL-----PFPFSSVAQHNRLGLYLLSLT---KSCDDIFEPKLEKLINQL 74
PpAPC5          -------PSSASP-----PFCVLPSSARHRLALFLLDQT---RVTDGFLEPTFEELGKEL 64
SmAPC5          -------PPALTP-----PFP-LPAAVRHQLALFLLGLA---KACDGFLEPTLEDLGMQL 67
CspAPC5         -------PTTETD----------PDPHSPLHALFGEALLREIRRRDEAASPSLMELLQHI 63
VcAPC5          --------MHAGL----------GHTAEALHALN-ETMRLAQQCGDPVVLLHALSVLCRL 41
MspAPC5         QASRLDRPSRRAVDPSTGDRLGPCVLGPADARRLAAVLMREASRSDGFREPDLRAFRRSL 100
OspAPC5         -------TANEGE-----------EREGEGEEEEKGDGARARWRRDGMFEPGPWEMVRRV 71

OsAPC5          KAVDALVNGWLCEQLTS-------------------------------TLSALTSPDDLF 122
SbAPC5          KAVDDLANGWFCEQLTS-------------------------------SLSALISPDDLF 121
PtAPC5          KEISGSLGHWLIDHLTS-------------------------------RLSSLSAPDDLF 97
VvAPC5          ------------------------------------------------------------
AtAPC5          REVGEEMDAWLTDHLTN-------------------------------RFSSLASPDDLL 103
PpAPC5          KDDLSDVGGVLFEQLGS-------------------------------RLPLLCTPEELF 93
SmAPC5          KECLGSIGEVIAEQLAS-------------------------------RLLGFSSPEDLF 96
CspAPC5         QVAAAAGGDHMCPGGESAGEADA--------------YFGAVKAAVGAHLSALETADELV 109
VcAPC5          LAAIAPGAPGLPPHG-------------------------------------------PA 58
MspAPC5         RRHPILVRETHRHPDDDDDEDDDDEDPMAAYTDAYPYSRETFADLLDDAIASLRGVDSVA 160
OspAPC5         RALARRAFEDAGDEDAG---------------------DDVERWCVEVFLEELDLATVSL 110

OsAPC5          NFFDKLR----GVLSAPEGANVED----------------EFLDPNSQLGVFLRCCILSF 162
SbAPC5          NFFDKLQ----GVLTASEGASAED----------------VFLDPNSQLGVFLRCCILAF 161
PtAPC5          SFFTEMR----GILGGLDSVVMEDNQ--------------VILDPNSNLGLFLRRCILTF 139
VvAPC5          ------------------------------------------------------------
AtAPC5          NFFNDMR----GILGSLDSGVVQDDQ--------------IILDPNSNLGMFVRRCILAF 145
PpAPC5          QFFQGLKELLAPVSYSAESGRGEDET--------------LLIQPNSLLGQFLRRCILAF 139
SmAPC5          TFVV------LGLRGKLFRGGGNEDS--------------LLVEHNSPLGQFLRRCILSF 136
CspAPC5         SLFTSVA---EQVITQCTSTPAEAEG--------------RGADASSAMGLYLRICYARY 152
VcAPC5          ALRSSLA---HHVQLLRMLRRCRERG--------------R------ELGQPHLTAFAQL 95
MspAPC5         ALIQEVAPRSRHEAEMRGAHDDDDDPSHPGGSYDPFPPGPGAAEESSSLGLFLRRCVADF 220
OspAPC5         DALERCFEDARAVVLNARVDEFDDALG----------LEMWGLEEWTPTSTFLKTCHLGF 160

OsAPC5          NTMTFEGVCHLLANLVEYCN-----------SADTSYDLAEDEDFNSEMEMSNFMDTNMH 211
SbAPC5          NSMTFEGVCHLLADLVMYCN-----------STDASYDLAEDEDFN--SEMGNLMDADIG 208
PtAPC5          NLLSFEGLCHLLTNIGSYCKEAMS-------SSNDLETLSEYENMDLENFMFGKVNEEIE 192
VvAPC5          ----------------------------------------------------------MH 2
AtAPC5          NLLSFEGVCHLFSSIEDYCKEAHSSFAQFGAPNNNLESLIQYDQMDMENYAMDKPTEEIE 205
PpAPC5          NVLSFEGSGRLVVELNAYRWLESSDPRGIFVDKDDMIEGEFDDEYEYEEDIDDVNMDGIH 199
SmAPC5          NILSFEGTCRLLAELDAYRRPALSIGDGNIVAKDSLSCQQAEGDDDEDEEEDRLEDD--- 193
CspAPC5         TAMTFEAICKLVGEV--------------------------------------------- 167
VcAPC5          AAARFAMLHDVE------------------------------------------------ 107
MspAPC5         EAQSFEGSVRVFGVFAAYVREGAGEYDDVDAAAARDRMDVVDKRLRVDARQAAAEAAVGE 280
OspAPC5         TSAPFEATTELLRTTRAYLDAAVR------------------------------------ 184

OsAPC5          VRDGVFDKYNQGYAP---------------------------------RSHMVDSSSSLV 238
SbAPC5          SQVGIFDKFHQGYAS---------------------------------ERHMGESSSALI 235
PtAPC5          ARKQASERVPFHLHGPKALSGL---------------------VEGIIDSSKHGDKCG-- 229
VvAPC5          ADIEVSAELKF----------------------------------------KHREKTG-- 20
AtAPC5          FQKTASGIVPFHLHTPDSLMKA---------------------TEGLLHNRKETSRTSKK 244
PpAPC5          DLEFRFRGTAAGRRG------------------------------------MQGRQQSGG 223
SmAPC5          ----EDENVIASVTP------------------------------------VHGRERHG- 212
CspAPC5         --HAYFDAAVATLQG--------------------------------------------- 180
VcAPC5          ----PSDSGLHAPPG--------------------------------------------- 118
MspAPC5         GGVEVHVDLRGDDEDDATLRRLGALHGSHLAPLTLDPRPGNATGPSQGGGVSRVPSGSEQ 340
OspAPC5         ----VENGIRDGCDWR-------------------------------------------- 196
```

```
OsAPC5          HAPASLHDFEEANMFKADDNLGPTCLRSRWQLEAYLNQQADILE--KDPSSVPLNSFNAT 296
SbAPC5          RAPMSTNDFDDANIFKADGN--PTCLRSRWQLEAYLNQQADILE--KDPGSVPLNSFNAT 291
PtAPC5          --ETSAYVHPPGNELRDVDPYGEIFLRTNWQVQGYLMEQADAIEGCRHDSSFSLNSFELV 287
VvAPC5          --EASSFAHHMKDTLRGIDPNGGIFLRTNWQIQGYLCEQADAIE--KHSCSFPLNAFESI 76
AtAPC5          DTEATPVARASTSTLEESLVDESLFLRTNLQIQGFLMEQADAIEIHGSSSSFSSSSIESF 304
PpAPC5          RNGASAFPVPNA-AISGDSGVKTRSLRTVEQVEGFLKEQAGLLE--KGVGQIPKEGLDSN 280
SmAPC5          -NGRRAFGVP----LIPTEGKVNGSFCFVKRVE--------------------------- 240
CspAPC5         ---------------RPSAPAPAPRLRPGPDLERFLNAQLAGLG--RRVGSVAQADIEAP 223
VcAPC5          ---------------SLTAAAAAAAVPP----------------------SAGAAPAEAP 141
MspAPC5         HSSVRALDLSAASMTQIRDGLRDVAVRPDAKLREWIARRCRGVD--QRDGYESTRDVTSA 398
OspAPC5         ---EARREDDAFRACAAPDALMALACRATARYDARGVDADAVLP-------------RHR 240


OsAPC5          MSQLQKLAPELHRVQFLQYLNALTHDDYVAALDNLHRYFDYSAGMQGLFS---------- 346
SbAPC5          MTQLQTLAPELHRVQFLQYLNALCHDDYVASLDNLHRYFDYSAGMQGLFG---------- 341
PtAPC5          LRQIKKLAPELIQVHFLRYLNSLYHDDYFAALDNLHRYFDYSAGAEGFD----------- 336
VvAPC5          LRQLQKLAPELHRVHFLRYLNNLYHNDYPASLENLHCYFDYSAGAEGFDF---------- 126
AtAPC5          LDQLQKLAPELHRVHFLRYLNKLHSDDYFAALDNLLRYFDYSAGTEGFD----------- 353
PpAPC5          LTQLEKLAPDMMKVHYLRYLNHLQQSDYPATMDDLHRYFDYSAGMGGMS----------- 329
SmAPC5          ------------QVHYLQYLNSIHHGDYPAAMSRLHQYFDY------------------- 269
CspAPC5         LGELAGAAPQVPKAHLAHHLSALHHRDVAASLDHLHRFFDHTAEARGAAAGGGSGAA--- 280
VcAPC5          AGAGGEGELRAAPLMVSCAVRDTHALATAASLSAAAPAAPPPTAADGTPPPPQRAAA--- 198
MspAPC5         LDDIERVAPKVPGAELARHLAHVSRRDFTQAMEHARRHFDYLPGTLGTTYGDGSYADGSY 458
OspAPC5         LDVLAELAPEMPTIHYLKHAEALNRRDFPAAVEHLHRHFDASGEHVDVRADLGS------ 294
                                              ::.

OsAPC5          ------------------------------------------------------------
SbAPC5          ------------------------------------------------------------
PtAPC5          ------------------------------------------------------------
VvAPC5          ------------------------------------------------------------
AtAPC5          ------------------------------------------------------------
PpAPC5          ------------------------------------------------------------
SmAPC5          ------------------------------------------------------------
CspAPC5         ------------------------------------------------------------
VcAPC5          ------------------------------------------------------------
MspAPC5         SNDGRAAGGRVALDVGAGTSFGLPMNPRQAFERGREIFGTGAFAGESSLQMNQSGVQAGE 518
OspAPC5         ------------------------------------------------------------


OsAPC5          ----RTASPFQDIIVGKYESALLCLGNLHCYFGHPKKALEAFTEAVRVSQMN----NDDS 398
SbAPC5          ----RSVAQVQDIVVGKYESALLCLGNLHCYFGHPKKALEAFAEAVRVSQMN----NDDS 393
PtAPC5          ----SAPSSSGSNSSGRYEIGLIYLGMMHLHFGHPKQALEVLTEAVRFSQQQ----SNES 388
VvAPC5          ----VQPTSSASNSFGRYEIALLCLGMMHFHFGHPKQALEVLTEAVRVSQQQ----SNDT 178
AtAPC5          ----LVPPSTGCSMYGRYEIGLLCLGMMHFRFGHPNLALEVLTEAVRVSQQL----SNDT 405
PpAPC5          -----VGGASCDSSVGRFQAGLLSLGSMHAHFGHVDQAMQALNEAVRIAQQY----NDDA 380
SmAPC5          -------------SVGRFQAGLLTLGSMHAHFGHVTQALQASFSALRIHLTFSMQNNDDA 316
CspAPC5         -AGQQAGEAAAARERGRLQSAALSLGSMHAQLGHMEQALQALNETVRIAQQS----SDDT 335
VcAPC5          -AASDLYSSPLLFDRSLPIGAAAAMADAVQQLAGAAHLLQSAAWALHGHNTL----ERAH 253
MspAPC5         ATAQEHADAAAAAQRARLQSALLTLGVAHFRFHHSGEALKALNEAVRTAQQN----GDEA 574
OspAPC5         -RRAEGEFESANAGRERLQTALLALATTHFAFSHVNEAMSAISEAVRTAQQN----GDET 349
                    .   :.     :      :.    :::

OsAPC5          CLAYILGAISNLLSKIGMSSTVGTIGSPYSLG---------------NNIGLGTPLSIQQ 443
SbAPC5          CLAYVLGAISNLLSKIGISNTVGIITSPYSLG---------------TNIGLGTPLSIQQ 438
PtAPC5          CLAYTLAAICNVLSEFGCSSSAGVLGTSFSP-----------------ITSMDTSLSVGQ 431
VvAPC5          CLAYTLAAICNLLSAIGISSTTEILGSSYGP-----------------VTSIGTSLSIQQ 221
AtAPC5          CLAYTLAAMSNLLSEMGIASTSGVLGSSYSP-----------------VTSTASSLSVQQ 448
PpAPC5          CLAHALAALCHLLFDVGAANEAYAKGESAGLR----------------DVGAGPSLGIQQ 424
SmAPC5          CLAHSLAALCHLLSEVGVAAEVTKAG-AIGDF----------------DRSIGPQLAAQQ 359
CspAPC5         CLAHALALLC---QVLDATTPGTITSVSHVPG-------------------ASPAARHYT 373
VcAPC5          TMMYLAAFSD---PQVGCGTP---------------------------------------A 272
MspAPC5         SLAHALAAFCALCASTAGSLASTEGAPAVEEWRAAGGDEGDKERRGVSISTQPPAVQAAA 634
OspAPC5         SLAHALALTTALMAQTRRGGE-----------------------------------RDAA 374
                 : :  .

OsAPC5          QLLVLLKRSLKRADTLKLTSLLSFDHLSLAKFD------------LKHVQRPLVSFGPNA 491
SbAPC5          QLLVLLKRSLKRADALKLPSLLSFDHLLLAKFD------------LKHVQRPLVSFGPNA 486
PtAPC5          QLFVLLRESLKRAESLKLKRLVASNHLALAKFDLLTSYLFQLYFDLQHVQRPLLSFGPKA 491
VvAPC5          QLFVLLRRSLKRADGLKLKRLVASNRLAMAKFG------------LTHVQRPLLSFGPKA 269
AtAPC5          RVYILLKESLRRADSLKLRRLVASNHLAMAKFE------------LMHVQRPLLSFGPKA 496
PpAPC5          QLLLLLRRCLRRSLELKLSQLVAFSRLALAKFY------------LKHVRRFSSLGGLEN 472
SmAPC5          HLLLSLKRCLKRALELKLPNLVAFSRLALAKFD------------LQVIR----ISGL-- 401
CspAPC5         QLGQLLHRCLRRCEELHLPHLVAFSQLALARLE------------LLHPQRPAASPGAAG 421
VcAPC5          RFEDRSTACAQLVAAVARRGPAAARAAAAACLG------------LLAASVCCDGTEDSS 320
MspAPC5         DARLLLRRLAKQARTLRIPHLMAYGELARARHG--------ASRPPCGPAPWSRGLETEG 686
OspAPC5         QLPTLLRRCAAQAAELSSPHLVAYASLALTKYE------------IDHPSTAVTGGGDIG 422
                               :      :      :
```

```
OsAPC5          STKLRTCPADVCK-------------------NLRLSSRVLTDFGTDGLSASNDNGSFST 532
SbAPC5          STKLRTCPADVIK-------------------NLRLGSRVLTDFGADVLSTSNDNGSFST 527
PtAPC5          SMKLRTFPINVCKVFGAFGDSIYSFSPISLLQELRLCSHLISEFGSE-SSTMTTDGVFST 550
VvAPC5          SMKLKTCPVNVCK-------------------ELRLSSHLISEFSTE-SSIMITDGVFST 309
AtAPC5          SMRHKTCPVSVCK-------------------EIRLGAHLISDFSSE-SSTMTIDGSLSS 536
PpAPC5          GGELGTSPLEVCK-------------------TLRLSPYLLGDSISNGISPHV---AISG 510
SmAPC5          --------------------------------TLRISPYLLGDLVHS----------MSS 419
CspAPC5         GAEGPAGGGGGTAPD---------------LPCSSSVAVAGAVRDVAHLHMATRLAAAAP 466
VcAPC5          PSYSESSAGGGAAT-------------------------------------AVTAAAAEG 343
MspAPC5         GSKKESGKTRDSDLSTADSALLFRRVASSPPAAAAAASQLVEALRHAVTLGAAAPQATTQ 746
OspAPC5         ESEVVTSTPTRATR-----------------------ALIDVELTRHAAKLASSTPASTE 459


OsAPC5          SWLRNLSAASNSWCSSSKKSGKLLTNDFDNFHFHAQPSPIPASVLQLAGSAYLLRATAWE 592
SbAPC5          SWLRNLSATSDSWRRSSMNTKKLHINDFDNFHYHAQPSPVPAPILQLAGSACLLRATAWE 587
PtAPC5          TWLNNLPKSMDSPLLPQENA---HRNNCDAHRFFTQLSSVPKSVLQLLGSSYIMRSTAWE 607
VvAPC5          AWLKNLQKPMGSLVLSLENA---SGANSNAFHFCAQPNSIPGSVLQLLGSSYLLRATAWE 366
AtAPC5          AWLKDLQKPWGPPVISPDSG---SRKSSTFFQLCDHLVSIPGSVSQLIGASYLLRATSWE 593
PpAPC5          TTNQQRGNGMNINQPLTAPGT---MAGGAWTSLTGRLGRTSDAVVKLAGTSHLLRAASWE 567
SmAPC5          SGSQRITSGAFG----QAPG----MPG---NTQTGRLGPLSESLLKLAGSSYLLRSCSWE 468
CspAPC5         SVPPLSSGASSARVLRGVGDLFASTAALYGPDMQGMQASSAAEVEQLVAGSHLLQAACWE 526
VcAPC5          STKASGGWDGGCNALALWGGPALAAAWLCAVHDRALALGDSAAAAQLVG----------- 392
MspAPC5         AAAAAAAASRSGADGQRANPNELYPPPKGLALSAGYPSGSESAMEQLSGSGSVLCGAVWD 806
OspAPC5         RALAVHRARGDAAVTAGSDVYPTPKGFPSTPASAHAASSVASAMASLTATASVLTSESWH 519
                                                             .* .

OsAPC5          HYGSAPMVRMNSLVYATCFAD--------------------------------------- 613
SbAPC5          HYGSAPMVRMNALVYATCFAD--------------------------------------- 608
PtAPC5          MYGSAPLARINSLVYATCFAD--------------------------------------- 628
VvAPC5          IYGSAPLARINALVYATCFSN--------------------------------------- 387
AtAPC5          LYGSAPMARMNTLVYATLFGD--------------------------------------- 614
PpAPC5          LYGSVPLVRVSALIHATCYAD--------------------------------------- 588
SmAPC5          LYGSDPMMRASTLVHAYCYSK--------------------------------------- 489
CspAPC5         LRGSRHLAQAHSLACLDAFGG--------------------------------------- 547
VcAPC5          -----------QLCALSDPQA--------------------------------------- 402
MspAPC5         AHGVPSMARMHALRHLRCDASRRFVRRDDETDADNDPGVVPGEFPESFGGSTRSSHEAEA 866
OspAPC5         AHGCSHLARMYALRQLMHDDE--------------------------------------- 540
                            *

OsAPC5          ---AASSSELSLAYVKLIQHLATFKGYSAAFSALKLAEEKFPL------SANSHIQLLKM 664
SbAPC5          ---AASSSELSLAYVKLIQQLAVFKGYSAAFCALKLAEKKFPS------STSLHIQLLGM 659
PtAPC5          ---ASSSSDAASVHAKLIQHLAVFRGYKGAFAALKVAEEKFLT------VSKSVILLVKL 679
VvAPC5          ---ASSSADVALAYTKLIQHLAVFKGHREAFAALKLVEEKFCS------ISKSRILLLKL 438
AtAPC5          ---SSSSSDAELAYLKLIQHLALYKGYKDAFAALKVAEEKFLT------VSKSKVLLLKL 665
PpAPC5          ---VASSDDVLLSYIKLAQHQAAFKGYAAAQSAFEVAAKRFPA------AANSLVRTAQL 639
SmAPC5          ---SASADDLSVAYVKLAHHLAAHKGYRVALTALEQAMKKFPL------HARSSLRSVQL 540
CspAPC5         ---VTRAEEQCTALAQLAVSVAAAHGPRAAEQVLAVADERFPG------AQSRVLAGARL 598
VcAPC5          ---HRDVEIRLEAARRGVLNLLAAGATEEAHRAATELFARCAD------AGLQAPALRCL 453
MspAPC5         DAASAAASDTAAALAQLARHASTHHGPDAAADVFAIAAARFPKRRMEEVAADPALAAAAA 926
OspAPC5         ----ASADDAATSCARLLASTSEREGASATEEVMDIVRDSFGARG----ERHKTTAVAFL 592
                               :         .   :  .

OsAPC5          QLLHERALHRGHLKVAQQICDEFAVLSSSVSGVDIELKTEARLRHARTLLAAKQFSQAAN 724
SbAPC5          QILHERALHRGHLKVAQQICDEFGVLSSSVSGVDIELKTEFSVRRARTLLAAKQFSQAAA 719
PtAPC5          QLLHECALHRGNLKLAQQVCDELGVLASSVSGVDKDLKTEASLRHARTLLAANQFSQAAA 739
VvAPC5          QLLHERALHLGHLKLAQQVCDELGVLASSVTGVDMELKTEASLRHARTLLAANQFGQAAA 498
AtAPC5          QLLHERALHCGNLKLAQRICNELGGLASTAMGVDMELKVEASLREARTLLAAKQYSQAAN 725
PpAPC5          QLVHDHALYRGELKLAQVACGELAASASPVFGVDMERKTEATIRHIRTLLVAGHLDEAAA 699
SmAPC5          QFIHSQAINRGNTRLAWVACSELAAMASPVLGVDMELKFEASYRHALTLLACKNYSEAAT 600
CspAPC5         SIAHDRALRRGDLHAAADIAAQMAALPPPTDSTDIGLRLEAEERIARTLLAAGCVEEAAA 658
VcAPC5          MLLAEVHLAAGDPHGAFLHVLACLLPAQPGPGPAGGG-----------VFAAAAAGAAAD 502
MspAPC5         ATDHDAARARGDGDACDAAARRIASLAPASTRTDPEARVEAFRARADACLISGGLGDAVF 986
OspAPC5         KLEYERAIDRGEYGAARCAARRARALVGFGDGADAELDFESRRMNANLDRVMQNFDAAQD 652
                    .     *.   .                                         *

OsAPC5          VANSLFSTCYKYNMQVENASVLLLLAEIQKNSDNAVLGLPYALASQSFCKSFNLDLLEAS 784
SbAPC5          VANSLFSTCYKYNMQVENASILLLLAEIHKKSDNAILGLPYALASQSFCKSFNLDLLEAS 779
PtAPC5          VAHSLFCMCYKFNMQVQNATVLLLLAEIHKKSGNAVLGLPYALASLSFCQSFNLDLLKAS 799
VvAPC5          VAHSLFCMCYKFNLQVENATVLLLLAEIHKKSGNAVLGLPYALASLSFCQSFNLDLLKAS 558
AtAPC5          VAHSLFCTCHKFNLQIEKASVLLLLAEIHKKSGNAVLGLPYALASISFCQSFNLDLLKAS 785
PpAPC5          VARLLFSQCYKASMQLESVLVLLLLAEIHKTADSAVTGLPYALAGLTLCRVFNLDYLQAS 759
SmAPC5          SAGELFALCYKYDMQLHVVKVLLLIAEIHKKSGSAVTGLPYVLGSITLSQSLNLDLLHAA 660
CspAPC5         AAHAACAVASTTGQPLHSARLQLLLARVHQQAGAPMAALPYALTATAHARQLAADMLAAE 718
VcAPC5          SSSLGAAVAGGG--------------------------------SGGGRGGRSHDLLAAE 530
MspAPC5         HASEAFQGSIREGLTHATLRATLTLAEAHLAAGAPAAALQHALALEHSAAALRLDGLRAA 1046
OspAPC5         ELRTIIKEAELCGDEHAVMRATLTLAETHLSADAPTLALTRALPLERLAAERGLEPIRAT 712
                        .                                             : : *
```

```
OsAPC5          ATLTLTELWLALG-------------------STHAKRALSLVCQSLPMILGHGGLELRA 825
SbAPC5          ATLTLAELWLALG-------------------SSHAKKALSLVYQSLPMILGHGGLELRA 820
PtAPC5          ATLTLAELWLSLG-------------------SNHAKRALTLIHGALPMILGHGGLELQA 840
VvAPC5          ATLTLAELWLSLG-------------------SNHAERASILVQGALPMILGHGGLELRS 599
AtAPC5          ATLTLAELWLGLG-------------------SNHTKRALDLLHGAFPMILGHGGLELRA 826
PpAPC5          AKETLAELWLGLG-------------------VGHAPRALALLQECLPMVLGHGGLELRA 800
SmAPC5          SRVSLAELWLDLG-------------------ADHAQRALDLLQQSLPLVLGHGSLELRA 701
CspAPC5         AVVLLAGLWCDMG-------------------AQHAQHARRELEGALPSILAHGSLELQA 759
VcAPC5          ALVLLCRVWYELSD------------------GQQLEEVLVLLQDALPLILAHGSVHLQA 572
MspAPC5         AIVVLAECWLAMSASAAGVGSTSTTVGQRRRRDGYASMAKDALDAHAPALLSRGGLALRA 1106
OspAPC5         VTCIACEAWLALG-------------------GSHARLARDTLDERSLALLSSDCLRTQA 753
                        *  :.                         .   :      :*. . :  ::

OsAPC5          RAHIVLAKCYLSDPKFSVSEDP-----------------SAVLDPLNQAAEDLEVLEYHE 868
SbAPC5          RAHIVLAKCHLADPKFSVLEDP-----------------EAVLDPLNQATEDLQALEYHE 863
PtAPC5          RAQITEAKCYLSDPSYSGSTLS-----------------SPFLDLLRQASDELQVLEYHE 883
VvAPC5          RAYIAEAKCYLSNPSFSVFENS-----------------EVVLDPLRQATEELEILEYHE 642
AtAPC5          RAYIFEANCYLSDPSSSVSTDS-----------------DTVLDSLRQASDELQALEYHE 869
PpAPC5          RTNLALARCFLSDPAFSAESQL-----------------AEVLDLLQQAAEEFELLEDYA 843
SmAPC5          RTNLCIARCYLSSTDFSVATAP-----------------ELVLDPLQLAAEEFMNLEDKD 744
CspAPC5         AAQVALAEACMTQHVTPAGLRED---------------GEWLLVLLEEAERVSLVLEDRR 804
VcAPC5          RAQLVLAEMVMSEASSPADLTHC---------------YSQLQRLLAGAAQAATAAEDFR 617
MspAPC5         RARMASAKAALACRSDDFDSSRMSSRTSSYPDDSTSGGWDEVLVPLEDAVACCAALGAHA 1166
OspAPC5         RAYAACARALVATTPECEFPTIAR----------------RVVDALERACERYVKLDARC 797
                 :    *.  ::                             .   *  *

OsAPC5          MAAEAYYLKAMVYNNLGK--LDEREEAAASFKEHTLALENPYNEEDSLAC---------- 916
SbAPC5          MAAEAYYLKAMAYNHLGK--LDEREEAAARFKDHVTALENPQNEEDSLAY---------- 911
PtAPC5          LAAEAFYLMAHVFDKLGQ--LERREEAAASFKEHMMALENPQD----------------- 924
VvAPC5          LAAEAFYLIAMVFDKLGQ--LEEREEAAASFMKHVKALENPQNEQDPLFNIH-------- 692
AtAPC5          LAAEASYLMAMVYDKLGR--LDEREEAASLFKKHIIALENPQDVEQNMA----------- 916
PpAPC5          LAGEAFYLQALAYNKWGS--VEERNSAAKAFQRCMQAL---------------------- 879
SmAPC5          QASEAFYLLATTFNSIGR--MEDRDKAAEKFQQCVVEEL--------------------- 781
CspAPC5         GAAHATYLRALVQDALGN--AEQRNAAAASFGALVQPAGALE------------------ 844
VcAPC5          MAAQAACMLAWLHHSQGS--VSERDTAAALQETLLDRQAEAEAACASEGRTLHVLRA--- 672
MspAPC5         KEAEAHELMARTFAAMGPNHVAARNAAARRWRECERRRRRAEVGDVGGVGGWGVGVGDGS 1226
OspAPC5         DAARAYASLADAHWRVAR-DATARDAAARRCRAFATEFTDATPTRDDAY----------- 845
                  ..*    *      .      *: **

OsAPC5          -----------------
SbAPC5          -----------------
PtAPC5          -----------------
VvAPC5          -----------------
AtAPC5          -----------------
PpAPC5          -----------------
SmAPC5          -----------------
CspAPC5         -----------------
VcAPC5          -----------------
MspAPC5         ARFGGIGSFPASRAVAV 1243
OspAPC5         -----------------


SmAPC7          ----MEILREQ----MGFLLDQGLHDSAEILGSFLLCAAA--SNNDLAPTVRAENMVLFA 50
PpAPC7          MGSFIEVLREQ----MTFLLEQGLYDSAEMLGTFLMSLVS--ANGELSPISRAESMILYG 54
OsAPC7          ----MEAARES----MAALVDAGLYDCAQTLGCFLVSSSP--ASSEAGMSMKVESLVLHG 50
SbAPC7          ----MEAARES----MAALLDAGLFGPAQTLGCFLVSSAG--AGNDAGMSMKVESLVQHG 50
PtAPC7          ----MDVPKDQ----ITALLDHELYNSAQILGSFLVSSAT--VSLETSPQLKAENQILLG 50
VvAPC7          ----MDVPRDQ----VTTLMEHGLYTSAQMLGCFLVSSSA--VNPETSPHIKAESLVLLG 50
AtAPC7          ----MEVPKEQ----IATLIEHGLYDSAEMLGCFLVSSPT--VSAETSPQLKAENLILLG 50
MspAPC7         ----MASTQQVR--AMERLLAAEMWESAEALGGFLCSASPRIPPDAVASAERARHLALFG 54
OspAPC7         --MSSPSLRDACTAHAREALAHGAHESAARWASLALSRRDVDDEGEGAAVASGRTSDGWR 58
                        ::         :       *   . :  .          .     .

SmAPC7          DALYGRREYKRALNFYRQALQQCRVTLKPNTAVTRSTLSSTGSRPSSANSSYCG-TINDN 109
PpAPC7          DALYGRKEFRRALNVYRQALQLCRATSKQPMSSARTPVQ---SRPSSAASAVHDSKINEN 111
OsAPC7          DALHGEKEFRRALSAYKQAMQYSKNIPRQATSNTRSSVTATGRSPSPNSSSLAP--LNEN 108
SbAPC7          DALYGEREFRRALNAYKQAMQYSRSIPRQATSSTRSSVSATGRSPSPNSSNLLS--FNEN 108
PtAPC7          DALFREREFRRAIHTYKQALHYYKIIPKQSS-TTSRS-SLSNRSSSPNSFNISA--INEN 106
VvAPC7          DALFREREHRRAIHTYKQALQHYKIIPRQNS-TTRISLSTSNRSSSPNSFNISA--INEN 107
AtAPC7          DALFHQREHRRAIHTYKQALHHYTRIPKQSSGISRSSLSLSTR-SSVNASSISA--INEN 107
MspAPC7         DALLGKGEHRRALNAFRQALSVNRLAPKVPTGNNRSS--AMGTPETPATPGISP-PVDEA 111
OspAPC7         RDAIDLEIKGDALAARGDARGAAAAYRRAAALAARVGGGDGGRASASGRDAMDGDEMSGY 118
                           *:    :*        :                 :          ..

SmAPC7          EVKYKIALCHMGLRDT---RSALTEMEAIPSKARTLRINLTLAKLYRVTGYDRAAVASYR 166
PpAPC7          EVKFKIGLCHLAVNDT---RAALSEMEGIPSKARSLRMNLTLAKLYRITGYDRVATASYR 168
OsAPC7          EVKFKIALCYSALREH---REALQEMEGIPSKARTLKMNLMLGKLYRISRNNRAAAVCYK 165
SbAPC7          EVKFKIALCHSALCEH---REALHEMEGIPSKVRTLKMNMMLGKLYRISRNSRTAAVCYK 165
PtAPC7          EVKFKIASCHATLNET---RAALVEMEGIPSKARTLQMSLLMAKLYRSSRHTRLAITCYK 163
VvAPC7          EVKFKIASCHCAINEN---VAALAEMEGIPSKARNLQMNLLMGKLHRNSRQNRAAIACYK 164
AtAPC7          EVRFKIASSHFALNET---KAAIAEMESV--KTRSLEMNILMAKLHRNSGYNRGAIAFYK 162
MspAPC7         SLKFKIGRCHLALREY---RAALAELETIPARARTLPVTMTLAKTYRRTGYERAAVACYK 168
OspAPC7         EMTMKLVRTLIKIGDEEAVREAKECLESTPPANRTLENRLLAAKIARREGHVRAAASAYK 178
                .:  *:      : :      *   :*      *.*   :  .*  *     * *   *:
```

```
SmAPC7          ECLRQCPYVLEAIIALAELGIPAKDIQLLFPQTPSKVARAPSDGHESSRLLQKLTEVHCG 226
PpAPC7          ECLRQCPYVLEAIVALAELGVPSKDIHALFPQGQSKISRSTTDCLDPVRWLQRFADGHSS 228
OsAPC7          ECLRQCPYVFEAITALAEMGLSSKEFSLIFSQAPNRGGKAPGDSLDAQRWWNRYVEAQCC 225
SbAPC7          ECLRQCPYVFEAITALAEMGLSAKEFSLLFPQAPNRGGKVPGDFVDAQRWWTRYVEAQCC 225
PtAPC7          ECLRHCPFVIEAIVALAELGVAAKDVISLFSQVSNRSGRAPLDHTDSTRWLQRYVEAQCC 223
VvAPC7          ECLRHCPYVIEAIIALAELGVTAKDILSLFPQTPNRSGRPPFDHFDSSRWLQRYVEAQCC 224
AtAPC7          ECLRQCPYVLEAVIGLAELGVSAKDIISSFTQTSNRSAKVSLDQIDPTRWLQRYVEAQCC 222
MspAPC7         EVVRDCPYAVDAIAALAELGCSAEEIR-----ADAHHEEPPGD--ASYGWLHHLAEAHGA 221
OspAPC7         EVLAKWPFAIEAVVALAELGVKSMDARRALRKTASEEDVAGED----FDVLEAYATAYAA 234
                * : . *:..:*: .***:*  : :           .       *           .

SmAPC7          IASLDYKGALENLNQLAQRFPNNLHVLLETGKVEGALGR-GDEAVHSFEKSRQVDPCNLT 285
PpAPC7          IATHDYKGGLEHFNNLAQRFPNNTHLLLENAKAEMAIMK-NDEAAHSFEKSRQIDQFNIS 287
OsAPC7          IASHDYKGGLDIYLDLMQRFPNNVHILLEIAKVEAIIGR-NDEAIMNFEKARLIDPNIMT 284
SbAPC7          IASHDYKGGLDIYLELMQRFPNNVHILLEIAKVEAIIGR-NDEAIMNFEKARLIDPNIMT 284
PtAPC7          IASNDYKGGLELFGELLQRFPNNIHILLEIARAEAIIGK-NDEAIMNFEKVRSIDPYVVT 282
VvAPC7          IASNDYKGGLELFTELLQRFPNNIHILLEIAKVEAIIGK-NDEAIMNFEKARSIDPHIIT 283
AtAPC7          VASHAYKGALELFAELLQRFPNNVHLLTETAKVEAIIGK-NDEAIMRFEKVRSIDPYTLT 281
MspAPC7         ARSHRLEAAASHLRRLDEIFPDDPRVWCQLARVHRDRGD-VQEAADCYRRCVRSDPCVVD 280
OspAPC7         LESDDLVTAQSHMQSVKRRFPNDPYMSIIKARIATVGSRDVAAATREYAAVRSRDPCFVE 294
                  :     . .    : . **::  :    .:           *   :      *   :

SmAPC7          DMDEYAMLLRMRGDTAEMNRLVYDLLNVDAGRPEVWVSSAVYWET-RDDRVRALSYADKV 344
PpAPC7          SMDEYAMLLRNRGDHMELNRLVHELINIDSTRPEVWVAAAVYWEM-RDDKIRALTYADK- 345
OsAPC7          YMDEYAILLKSKSDYTKLNKLVHDMLHIDPARPETCLALAALWER-KDERK-ALTYAEK- 341
SbAPC7          YMDEYAILLKSKSDYVKLNKLVHDMLHIDPARPETCVALAAMWKR-KDKRK-ALTYAEK- 341
PtAPC7          YMDEYAMLLKTKGDFSKLNKLVHDLLSIDPTRPEIFVALSVLWEK-KDEIG-ALSYAEK- 339
VvAPC7          YMDEYAMLLMIKSDHLKLNKLVHDLLSIDPTRPEVFVALSVVWER-KEERG-ALSYAEK- 340
AtAPC7          SMDEYAMLLQIKCDYSRLNKLVHDLLSVDHTRAEVFVALSVLWER-KDART-ALSYAEK- 338
MspAPC7         CMDAFAALLN--GPSVELNALVNNLLENAPGRAESWSAAALYWES-RGDAEKALSFAER- 336
OspAPC7         GMDAYGMLLRESGDARALNVLSSDLAHLMPQSAESWTCMAMRFDAERGGREDAVAAAEK- 353
                 ** :. **        :* *  ::       .*   . :  :.  :     *:: *::

SmAPC7          TAILQSIRVADRHTPAYLLKGNLSLSLNHSEAAVMAFRKAQSLKPDLRSYQGLVRSYLAL 404
PpAPC7          -----SLRVDDRHTSAYVVKGNISLTLNRPEAAVMAFRKAQLLKADLRSYQGLVRAYLAI 400
OsAPC7          -----SLRVDDRHITGYIMKGNLHLLLNRPDLAVTDFRGAQELRADLRSYQGLVRAYLAL 396
SbAPC7          -----SLRVDDRHITGYIMKGNLHLSLNRPDLAVTDFRGAQELRADLRSYQGLVCAYVAL 396
PtAPC7          -----SIRIDERHIPGYIMKGTLLLSLKRPEAAVIAFRGAQELRADLRSYQGLVHSYLAF 394
VvAPC7          -----SIRIDERHIPGYIMKGNLYLSMNRPDAAVVAFRGAQELKPDLRSYQGLVRSYLAL 395
AtAPC7          -----SIRVDERHIPGYIMKGNLLLQAKRPEAAAIAFRAAQNLRSDLRSYQGLVHSYLAF 393
MspAPC7         -----ASDIDDQHVTAHVTKGYLRLKCKRADAAVHAFKRALQLAPATRTYAGLVASYLIL 391
OspAPC7         -----AVLLNPQSNIAHLVLGSIYLRSKRYKSAVRAFNQCNAIKVSMEAYHGLVKAYLAL 408
                     :  :  :    .::  * : *  :: . *.  *. .  :    .:* *** :*: :

SmAPC7          SKNKEALCAAREAMKAMPHSAKALTLVGDVYAHVPEGREKARKFYESALRLEPGYLGAVL 464
PpAPC7          PKHKEALCAAREAMKAMPQSAKALTLVGDVYAAHQDGRDKARRFYESALRLEPTYLGSVF 460
OsAPC7          SKCKDALFTAREAMKVMHQSAKALKLVGDVHAISSSGREKARKFYESAIRLEPGFLGAAL 456
SbAPC7          SKCKEALFTAREAMKVMHQSAKALKLVGDVHAISSSGREKARKFYESAIRLEPGFLGAAL 456
PtAPC7          SKIKEALHAAREAMKAMPQSAKALKLVGDVHASNSGGREKAKKFYESALRLEPGYLGAAL 454
VvAPC7          SKIKEALYVAREAMKAMPQSAKALKLVGDVHASNSGGREKAKKFYESALRLEPGYLGAAL 455
AtAPC7          GKTKEALYTAREAMNAMPQSAKALKLVGDVHAFTSSGREKAKKFYESGLRLEPGYLGAVL 453
MspAPC7         GRIKEATATAKECARAAPNASASHALLGDVEAAAQGHRDRARRFYEHSLKLDPSCAGVAA 451
OspAPC7         ASRPNAAMCAKQALKRSPQSALAWSLMGDVHAKNRDEYDDAIKAYEQALAYDPRHVRSVK 468
                    :*   *::. .   ::: :  *:*** *      : * : ** .:  :*     .

SmAPC7          ALADLHRMESRNEEATLLLQRYLQNWADD------ALHTKLAQILALTNKLGESLSHYQA 518
PpAPC7          ALADLYGMEGRTEEAILLLQRYLKTWADD------ALHTKLAQIFAASDKLGDSLFHYQT 514
OsAPC7          ALADLHVAEGRNKDAVLLLERYLRQWTDD------SLHIKLAQVFAATNMLSDALSHYQS 510
SbAPC7          ALADLHVVEGRNKEAVMLLEKYLRQWADD------SLHIKLAQVHASTNMLSDALSHYQS 510
PtAPC7          ALAELHVIEGRNGDAVSLLERYLKDWADD------SLHVKLAQVFAATNMLQEALSHYQA 508
VvAPC7          ALAELHVMEGRTGDAISLLERYLKDWADD------SLHVKLAQVFAATNMLQDALSHYQS 509
AtAPC7          ALAELHLMEGRNGDAVSLLERYLKDYADD------SLHVKLAQVFAATNMLQDSLSHFQA 507
MspAPC7         ALAETHAASGRSEAAAELLRRHLDTHAAHDAGAQVALHCRLGAVLAQSKQLADALGHYQS 511
OspAPC7         ALAALNIKIGKVHVACAILQRQLDDYQPSDENELVQLYCRLAQALMLSRQTADGVKYYTR 528
                ***      .:    *  :*.: *              *: :*.     :    :.: ::

SmAPC7          ALSINPQNEAARKGLERLEKQMKGVDPDAL------EEDEENEAEDGEGDPEE-AEFLYI 571
PpAPC7          ALSINPANDAAKKGLERLEKQMKGVDPDAL------EEDEENEGEDPDADAEE-GEFL-- 565
OsAPC7          ALRINPHNEAAKKGLERLEKQMKGVDPDAP------EEDEENEADDVDGDQDD-AELL-- 561
SbAPC7          ALRINPQNEAAKNGLERLEKQMKGVDPDAP------EEEEENEEDDID-DRD---EFI-- 558
PtAPC7          ALRINPQNEAAKKGLERLEKQMKGVDPDAP------EEDEENEVEDADGDQEE-TDLL-- 559
VvAPC7          ALRINAQNEAAKKGLERLEKQMKGVDPDAP------EEDEENEVEDADGDQEE-AELL-- 560
AtAPC7          ALRINPQNEAAKKGLDRLEKQMKGIDPDAT------DENDENDVEDVDGDTEE-AELM-- 558
MspAPC7         ALAIYPESDEARRGVSRVERLMKGQDPDAPDEEVDEEEDDDEEVEDADADGDDGSDFMG- 570
OspAPC7         ALAIQPTCDIAKRALEKFEQTRNPEHAATMS-----DEDQEGAEDMMEDDASRDGDWMVE 583
                ** *  .  : *:..:.:.*:  :  .. :        :*:::   :  : * .   : :

SmAPC7          AGLRPCWSDNHSTQLRHFSKEHGILTAQVSSRRKKLRLRCKASSQSSQQQSSLLSFLCPL 631
PpAPC7          ------------------------------------------------------------
OsAPC7          ------------------------------------------------------------
SbAPC7          ------------------------------------------------------------
PtAPC7          ------------------------------------------------------------
VvAPC7          ------------------------------------------------------------
AtAPC7          ------------------------------------------------------------
MspAPC7         ------------------------------------------------------------
OspAPC7         GRTTSA------------------------------------------------------ 589
```

```
SmAPC7          LKLIGSGDPSAPRNGLLESATSGIASLARLPWGKNVNTQIVSSRTKQPLELFKLYEFEAC 691
PpAPC7          ------------------------------------------------------------
OsAPC7          ------------------------------------------------------------
SbAPC7          ------------------------------------------------------------
PtAPC7          ------------------------------------------------------------
VvAPC7          ------------------------------------------------------------
AtAPC7          ------------------------------------------------------------
MspAPC7         ------------------------------------------------------------
OspAPC7         ------------------------------------------------------------

SmAPC7          PFCRRVREALTELDLSAEVYPCPKGSRVHRAFVKASGGKEQFPFLLDPNTGVSMYESSDI 751
PpAPC7          ------------------------------------------------------------
OsAPC7          ------------------------------------------------------------
SbAPC7          ------------------------------------------------------------
PtAPC7          ------------------------------------------------------------
VvAPC7          ------------------------------------------------------------
AtAPC7          ------------------------------------------------------------
MspAPC7         ------------------------------------------------------------
OspAPC7         ------------------------------------------------------------

SmAPC7          VNYLFQEYGERRRPTFGILESTLVTGWVPTIIRAGRGMSLWNGALPDPPQKLLELYSYEN 811
PpAPC7          ------------------------------------------------------------
OsAPC7          ------------------------------------------------------------
SbAPC7          ------------------------------------------------------------
PtAPC7          ------------------------------------------------------------
VvAPC7          ------------------------------------------------------------
AtAPC7          ------------------------------------------------------------
MspAPC7         ------------------------------------------------------------
OspAPC7         ------------------------------------------------------------

SmAPC7          NQFARLVREALCELELPYILWNTGKGSLNCSKLKQISGSTQVPYLVDPNTGIQMAESLDI 871
PpAPC7          ------------------------------------------------------------
OsAPC7          ------------------------------------------------------------
SbAPC7          ------------------------------------------------------------
PtAPC7          ------------------------------------------------------------
VvAPC7          ------------------------------------------------------------
AtAPC7          ------------------------------------------------------------
MspAPC7         ------------------------------------------------------------
OspAPC7         ------------------------------------------------------------

SmAPC7          IRYLFANYNSKNEAFGASPGRFSATAGNELKVGRFEVLESKDELDLIGQQPPEPLRQFNQ 931
PpAPC7          ------------------------------------------------------------
OsAPC7          ------------------------------------------------------------
SbAPC7          ------------------------------------------------------------
PtAPC7          ------------------------------------------------------------
VvAPC7          ------------------------------------------------------------
AtAPC7          ------------------------------------------------------------
MspAPC7         ------------------------------------------------------------
OspAPC7         ------------------------------------------------------------

SmAPC7          RFQSYIRKFVLCGDLIRLCITLPSSSLLGLVLIRTLSPGISSVPFAMRRQLVEGHDPVLW 991
PpAPC7          ------------------------------------------------------------
OsAPC7          ------------------------------------------------------------
SbAPC7          ------------------------------------------------------------
PtAPC7          ------------------------------------------------------------
VvAPC7          ------------------------------------------------------------
AtAPC7          ------------------------------------------------------------
MspAPC7         ------------------------------------------------------------
OspAPC7         ------------------------------------------------------------

SmAPC7          PVVNNLSLGMAKALGLSGVGKFHGGWFSLTVGQVAWSFRLVNRLPMTGCFKDG 1044
PpAPC7          -----------------------------------------------------
OsAPC7          -----------------------------------------------------
SbAPC7          -----------------------------------------------------
PtAPC7          -----------------------------------------------------
VvAPC7          -----------------------------------------------------
AtAPC7          -----------------------------------------------------
MspAPC7         -----------------------------------------------------
OspAPC7         -----------------------------------------------------
```

```
OspCDC23        -------------------------MAYFDLG------EYRRCAHTLRDARAPLPTFLR- 28
MspCDC23        -------------MAGSRVKHSELRQAVRDLQNRGLLHSARWAAEQLYGLEDEVPGARED 47
VvCDC23_1       -------------MSSKDSCRNELRFAIRQLSDRCLYSAAKWAAEQLVGIEQDPA----- 42
VvCDC23_2       -------------MSSKESCRNELRTAICQLSDRCLYSAAKWAAEQLVGIEQDPA----- 42
PtCDC23         -------------MNSKETCRSELRIALRQLSDRCLYSASKWAGEQLVGIEQDPA----- 42
AtCDC23         -------------MVSKECCRNEIRAAIRQLSDRCLYSAAKWAGEQLVGIEQDPS----- 42
OsCDC23_1       ------------MASSNETYRVELRAAARQLGERGLYSAAKWAAELLVGIEPDATPAPSS 48
OsCDC23_2       ------------MASSKEAYRVELRAAARQLGERGLYSAAKWAAELLVGIDPDATPAPSS 48
SbCDC23         ------------MASAKETYRAELRAAARQLGERCLYSAAKWAAELLVGVEPDAAPVPSA 48
SmCDC23         ------------------------------------------------------------
PpCDC23         ------------------------------------------------------------
CspCDC23        ------------------MQAQELRQAVHDCRSRALYASAKWAATALCGLPEEEV----- 37
CmCDC23         MTSEVDVALAVLRQVHTEQKYRELKEALRESLDRGLVLSARWLGELVASLGRKLEP---- 56
VcCDC23         -MVPERPGATVPSPATPADIAAELTLAVHDLNARGLFQAAQWAAEQLVGLELHSPHQG-- 57

OspCDC23        -EYATFLAG-----------EKSKGQSGAVGGAS---VDGGSGRIDASQPTAIGTRPP-- 71
MspCDC23        EDDATPAAA-----------PATPATPFANDDAS---DDEMDLGTDEKPAKPPATAPPAD 93
VvCDC23_1       -KFTPSHTR-----------FQRGSSSIRRRFRT---NEIAST--PTAGVSYVSTPVL-- 83
VvCDC23_2       -KFTPSHTR-----------FQLGSSSIRRRFRT---NEIAST--PTAGVSSVSTPML-- 83
PtCDC23         -KFTPTNTR-----------FQRGSSSIRRRFRT---NDITST--PVTGMSYVSTPVL-- 83
AtCDC23         -NFTPANTR-----------FQRGSSSIRRRFST---NESISTPLPSVGFSQAATPLP-- 85
OsCDC23_1       VMDTPSSSGSAASGGRLLHLHRSGGSSFRRRLRP---GAA-EAGTPLGGVSYVSTPIP-- 102
OsCDC23_2       AMDTPSSSG---SGGHLLHLHRSGGSSFRRRLRP---GAAGEAGTPLGGVSYVSTPIP-- 100
SbCDC23         VMDTPSSSS-ATSAGRLLHLHRSGGSSFRHRPRPAGGGTSSEAGTPLGGVSYVSTPIP-- 105
SmCDC23         ------------------------------------------------------------
PpCDC23         ------------------------------------------------------------
CspCDC23        ----------------------------------------------MVSSQAAAAAPP-- 49
CmCDC23         -------------------------------------------QLLDNDAETVGTK---- 69
VcCDC23         ----------------------ASGWQHHQQHHPQGHAGFTSRTSSPQTANLLSRNDP-- 93

OspCDC23        --------GGVVEDAGE--------------------SNAELESLNQWLR---------- 93
MspCDC23        DAPEWRGRGGTPEAAGDDFILAKAYFDLGEYRRASHQVTENRSSLGKFLRYYSLYLAGEK 153
VvCDC23_1       ------EEDEAVDG--DFYLLAKSYFDCREYRRTAHVLRDQTGKKAVFLRCYALYLAGEK 135
VvCDC23_2       ------EEDEAIDG--DFYLLAKSYFDCREYRRAAHVLRDQTRKKAVFLRCYALYLAGEK 135
PtCDC23         ------EEDEVIDG--DFYLLAKSYFDCREYKRAAHVLRDQNAKKSVFLRCYALYLAGEK 135
AtCDC23         ------EEDEAIDG--DIYLLAKSYFDCREYRRASHMLRDQVSKKSLFLRYYALYLAGEK 137
OsCDC23_1       ------DDD-AFDVGGDRYLLAKTYFDCREYRRAAHVLRGQTGRKAVFLRCYALYTAGEK 155
OsCDC23_2       ------DDDDAFDVGADR---LETLYSNIEMNGQYKVLTYSSNTIALMQQGGLRIQAGEK 151
SbCDC23         ------DDD-AFDSGGDKYLLAKTYFDCREYRRAAHVLQKQVGRKAVFLRCYALYMAGEK 158
SmCDC23         ---------------------------MREYRRAAHALRGATGKKSFFLRCYATYLAGEK 33
PpCDC23         -------DDIDSEQDDDGLLLARSYFDTREYRRAAHALQSASGSTATFLRFYATYLAGEK 53
CspCDC23        -------------SGSAAFELARSFFDLKEYRSAAHALRDSRDQLSLFLRGYATYLAGEK 96
CmCDC23         ------------RGESDLFFYGKALFDAREYLRAASILEACTSKLGRFLRWYSLFLHGEK 117
VcCDC23         ------------DEQHPQYLLARAYFQSKEYRRTAHALSGLTGPLPTFLRLYATYLAGEK 141
                                                                 :

OspCDC23        -------------------------------AQDDTGA-----KDGFLTFLHGIVCRESG 117
MspCDC23        RKNEEMLELE------------TLSFDLELILKDDAPANAECRNDPFLHYLLGLVLVERE 201
VvCDC23_1       RKEEEMIELE-----GPLGKSDAVNHELVSLERELSTLRKNGTVDPFGLYLYGLVLKEKG 190
VvCDC23_2       RKEEEMIELE-----GPLGKSDAVNHELVYLERELSMLRKNGTVDPFGLYLYGLVLNKKG 190
PtCDC23         RKEEEMIELE-----GPLGKSDAVNRELVSLERELSTLRKNGTIDPFGLYLYGLVLKNRG 190
AtCDC23         RKEEEMIELE-----GPLGKSDAINRELVSLERDLSALRRTGAIDSFGLYLYGVVLKEKG 192
OsCDC23_1       RKEEETVELE-----GSLGKSNAVNQELVALERELATHRRTGAIDSFCLYLYGIVLRDKG 210
OsCDC23_2       RKEEETVELE-----GSLGKSNAVNQELVALERELATHRRTGAIDSFCLYLYGIVLRDKG 206
SbCDC23         RKEAEMIELE-----GSLGKSNAVNQELIALEKELSTHKRTGSIDSFGLYLYGIVLRDKG 213
SmCDC23         RKEEEIIELG-----GPLGRSDAVNPELAGLEQELTSHSEKGTLDAFGNYLYGVVLHERD 88
PpCDC23         RKEEDTVDLA-----GPLGRSDAVNLELVSLEQELSSLYRMGTLDAFGMYLYGVILRERD 108
CspCDC23        RKEEERIESK-----AAGEAAAAANTELDGLEGELQALVAAGQADGFLLYLLGLVLADRE 151
CmCDC23         RKEEMLLEVSPAGCAAPCKPQNTLAASIVQEMDKAVQSDASLPEDAYICWLRGTALKACG 177
VcCDC23         RRDPTAVAAS----------------------------------ATPLLFPAASAPVVQT 167
                                                                 :  .

OspCDC23        QPTQAKTLLAEACRKYPLNWSAWQALIPMLASEE----EEKALDLPRDHWVYTWFIGVFQ 173
MspCDC23        SRDKAKVSLCAACRGYPCNWGAWEALMPLCATVE----EAQALPLP-DHWMRKWFIAALQ 256
VvCDC23_1       SENLARTVLVESVNSYPWNWNAWTELQSLCTTID----ILNSLNLN-NHWMKDFFLASVY 245
VvCDC23_2       SENLARTVLVESVNSYPWNWNAWTELQSLCTTID----ILNSLNLN-YHWMKDFFLANAY 245
PtCDC23         NQNLARTVLVESVNSYPWNWNAWTELQSLCTTIE----MLNSLNLS-NHWMKDFFLASAY 245
AtCDC23         NESLARASLVESVNSYPWNWSAWSELQSLCTSIE----ILNSLNLN-NHWMKEFFLGNAY 247
OsCDC23_1       SEALARTVLVESVNSYPWNWSAWLELQSLCTSSD----ILNNLNIK-NHWMKDFFLASAH 265
OsCDC23_2       SEALARTVLVESVNSYPWNWSAWLELQSLCTSSD----ILNNLNLK-NHWMKDFFLASAH 261
SbCDC23         CEGLARTILVESVNSYPWNWSAWSELQSLCTSSD----ILNKLNLK-NHWMKDFFLASAY 268
SmCDC23         RKSEARAVLCASVNTYPWNWSAWLELQALCTDPE----ILPTLRLE-DHWMRDFFIASVY 143
PpCDC23         KHAEACTVLCASVNSYPWNWSAWLELQALCTDPD----IFHTLDLK-DHWMRDFFVASLF 163
CspCDC23        KKEEARQALAASVTAYPCNWSAWLALQSVCADLA----AVGQLPLP-DHFMRRFFLASLC 206
CmCDC23         CRKEALCAFIEALQRKPHLWAAWTALHELGEDYSSIISALRTLLETGKAWMFAFFLVMQS 237
VcCDC23         NAPPSPTAVAAAATLMLFPAAAAPVVQTNAPPSP---TAVAAAATP--------LLFPAA 216
                    :   .  :         *   :                            ::
```

```
OspCDC23            LEIQKNKESLVTFDSLSNDFPQSKLLLGHVAEAHYNLREFEEAHSIYKDMQEVDPYRVEG 233
MspCDC23            LELQDNRKGLQAYASLVMDIPASAIGVVQMAVGHYNMREFDRAQSIFEDVYKADPYRLEG 316
VvCDC23_1           QELRMHNESLGKYEYLQGTFSFSNYIQAQIAKAQYSLREFEQVEIIFDELLRNDPYRVED 305
VvCDC23_2           QELRMHNESLGKYEYLQGTFSFSNYIQAQIAKAQYSLREFEQVEVIFEELLRNDPYRVED 305
PtCDC23             QELRMHNESLAKYEYLQGTFSFSNYIQAQIAKAQYCLREFDQVEVIFEELLRNDPYRVED 305
AtCDC23             QELRMHTESLAKYEYLQGIFSFSNYIQAQTAKAQYSLREFDQVEIMFEELLRNDPYRVED 307
OsCDC23_1           LELKMHEEALKRYERLMGVFRCSDYIQAQIATVQYSMRDLDEADMIFEELLRTDPFRVDS 325
OsCDC23_2           LELKMHEEALKRYERLMGVFRCSDYIQAQIATVQYSMRDLDEADMIFEELLRTDPFRVDS 321
SbCDC23             LELKMHEEALKRYERLMGVFRCSGYIQAQIATVQYSMRDLDEAEMIFEDLLRTDPFRVDS 328
SmCDC23             LDLQKNSEGLACYRSLHAMFPVSDYVLAQTATAHYNLREFDEAEGLFEELLRTDPYRIEG 203
PpCDC23             LDLQRNSEGLARYQSLNLLFPGSDHILAQTAVAHYNLREFDDAERLFEELLRADPYRIEG 223
CspCDC23            VDMHHNAEALQHLQGLSDEFPRGEAVILLAALAHYNLQNFDEAQELFEGLLLRDPHRIEG 266
CmCDC23             GTFSGETSIIPVLQALSSEFPDSVTLLHLLAHAHFSAHDFETAAELCRRLRELDPYFLDA 297
VcCDC23             SAPVVQTNAPPSPTAIAAAATPLLIPAAAAPVVQTNAPPSPTTVAAAATLLLFP--AAAG 274
                         . .       :              .  :        .      :

OspCDC23            MDNYSNVLYVQELFAELSHLAHHLVSTDKYTPETCCVVGNYYSLKSMHNKAVVYFKRALK 293
MspCDC23            MDTYSNILYVKEATAKLSYLAHCAVLTDKYRPETCCIVGNYYSLKAQHEKAVVYFSRALR 376
VvCDC23_1           MDMYSNVLYAKECFSALSYLAHRVFLTDKYRPESCCIIGNYYSLKGQHEKSVVYFRRALK 365
VvCDC23_2           MDMYSNVLYAKECFSTLSYLAHRVFLTDKYRPESCFIIGNYYSLKGQHEKSVVYFRRALK 365
PtCDC23             MDMYSNVLYAKECFSALSYLAHRVFMTDKYRPESCCIIGNYYSLKGQHEKSVMYFRRALK 365
AtCDC23             MDLYSNVLYAKEACAALSYLAHKVFLTDKYRPESCCIIGNYYSLKGQHEKAVMYFRRALK 367
OsCDC23_1           MDVYSNLLYAKESSTALSFLAHRVFLTDKYRPESCCIIANYYSLKGQHEKSVLYFQRALK 385
OsCDC23_2           MDVYSNLL---------------------------------------------------- 329
SbCDC23             MDIYSNLLYAKESLTALSFLAHRVFLTDKYRPESCCIIANYYSLKGQHEKSVLYFQRALK 388
SmCDC23             MDMYSNILYVKECFAALSHLAHKAVLTDKYRPETCCIIGNYYSLKAQHEKAVLYFKRALK 263
PpCDC23             MDTYSNILYVKECFAALSHLAHKSVLTEKYRSETCCIIGNYYSLKAQHEKAVLYFKRALR 283
CspCDC23            MDIYSNILYVKEEFAALSALAHRCAGADKYRPETCCVIGNYYSLRGMHERAVQYFRRALR 326
CmCDC23             VDLYSNILFVQEDQATLSTLARDCVQIDKYRAETCCVVGNYFALRQNHEKAVQYFRRALT 357
VcCDC23             TDTFSNILFVKEAAAPLSVLAHRVAATDKYRPETCCVLGNYYSLQAASQAGPYREVGCLP 334
                     * :**:*

OspCDC23            LNPRYLSAWTLMGHEYVEMKNPAAAIDAYRHAVDINPRDYRAWYGLGQTYEILQMPYYAL 353
MspCDC23            LNWKYLSAWTLMGHEYVEMKNPAAAIDAYRHAVDINPRDYRAWYGLGQTYEILTMPYYAL 436
VvCDC23_1           LNKNYLSAWTLMGHEYVEMKNTPAAVDAYRRAVDINPCDYRAWYGLGQAYEMMFMPYYAL 425
VvCDC23_2           LNKNYLSAWTLMGHEYVEMKNTPAAVDAYRRAVDINPCDYRAWYGLGQAYEMMGMPYYAL 425
PtCDC23             LDKKYLSAWTLMGHEYVEMKNTPAAVDAYRRAVDINPCDYRAWYGLGQAYEMMGMPFYAL 425
AtCDC23             LNKKYLSAWTLMGHEYVEMKNTPAAIDAYRRAVDINPTDYRAWYGLGQAYEMMGMPFYAL 427
OsCDC23_1           LNRKYLSAWTLMGHEFVELKNTPAAIDAYRRAVDINPRDYRAWYGLGQIYEMMGMPFYAV 445
OsCDC23_2           ------------------------------RAVDINPRDYRAWYGLGQIYEMMGMPFYAV 359
SbCDC23             LNRKYLSAWTLMGHEYVELKNTPAAIDAYRRAVDINPRDFRAWYGLGQIYEMMGMPFYAL 448
SmCDC23             LNRKYLSAWTLMGHEYVEMKNTPAAIDAYRRAVDINPRDYRAWYGLGQTYELLIMPFYAL 323
PpCDC23             LNPKFLSAWTLMGHEFVEMKNTPAAIDAYRRAVDINPRDYRAWYGLGQTYEILAMPYYAL 343
CspCDC23            LNPAYLAAWTLMGHEFVELKNPPAAIEAYRHAVDVNPRDYRAWYGLGQTYELVNMPYYAL 386
CmCDC23             LNRSYTTAWILMGHEFLEMRNTSAAVEAYRRAIDLDPADFRPYYGLGQTYELLHMPHYAL 417
VcCDC23             AKVWCVGR---------------DGRDAYRRAIDVSPQDFRAWYGLGQAYELLKMPYYAL 379
                                                  :*:*:.* *:*.:***** **:: **.**:

OspCDC23            YYYQQAVKLRPDDSRMWCAMGQCYESDQLRMFTSAIRCYQRAVANNEGEGIALAKLAMLH 413
MspCDC23            YYYQRATRLRPKDPRMWCAMGQCYESDQLQMTVAAIRCYQRAHQNGDQEGIALGKLAKLH 496
VvCDC23_1           HYFRKSVFLQPNDSRLWIAMAQCYETDQLQMLEDAIKCYKRAANCNDTEAIALHQLAKLS 485
VvCDC23_2           HYFRKSVFLQPNDSRLWIAMGQCYETDQLQMLEDAIKCYKRAANCNDTEAIALHQIAKLS 485
PtCDC23             HYFKKSVFLQPSDSRLWIAMAQCYETDQLHLLEDAIKCYRRAANCNDKEAIALHQLAKLH 485
AtCDC23             HYFRKSIFFLPNDSRLWIAMAKCYQTEQLYMLEEAIKCYKRAVNCTDTEGIALNQLAKLH 487
OsCDC23_1           YYFRKSSYLQPNDARLWNAMAQCYESDQLQMIEEAIKCYERSANNNDTEGIALHQLAKLH 505
OsCDC23_2           YYFRKSSYLQPNDARLWNAMAQCYESDQLQMIEEAIKCYERSANNNDTEGIALHQLAKLH 419
SbCDC23             HYFRKSSYLQPNDARLWIAMAQCYESDPLQMIEEAIKCYERAADSNDTEGIALHQLAKLH 508
SmCDC23             YYYRRAAQLRPHDARMWCAMGQCYENEQLQMFDAAIRCYRRAVNNNDREGIALNKLAKLH 383
PpCDC23             YYYRRATQLRPHDARMWCAMGQCYESDQLQIYDAAIRCYKRAVNNNDREGIALNKLAKLH 403
CspCDC23            YYFRRAVQLRPHDARMWNAMGHCYQQEQLGLLDAAIRCHRRALP-YDKE----------- 434
CmCDC23             YYFEKAATLRPCDDRMWAAVSQALQD--IGRLDDAVRCLEKALTWNPDNWSYAKRAGDLF 475
VcCDC23             YYYRRAAQLRPTDARMWCALAQCFVHEQIGLQDAAVRAYQRAIAHDDPDGIAVHKLAKLY 439
                    :*:.::  : * * *:* *:.:.     :     *::. .::      :

OspCDC23            REKN-PKAAAHYYILNLKR---LDKEDLESAEK-HEALEFLAHYYMKEQRLQEAEAACAR 468
MspCDC23            HEANNAKAAAHYHRLNLVR---LLEEGADQHEDTVKALSYLADYYKNTKDYGKAEACCMR 553
VvCDC23_1           KELKRSEEAAFYYKKDLER---MEAEERE-GPNMVEALLFLATYYKSQKRFEEAEIYCTR 541
VvCDC23_2           KDLKRSEEAAFYYKKDLER---MEAEERE-GPNLVEALLFLATYYKSQKRFEEAEIYCTR 541
PtCDC23             FELGRPEEAAFYYKKDLDR---MEDEERE-GPNMVEALLFLAQHCRTHKRLEEAEVYCTR 541
AtCDC23             QKLGRNEEAAYYFEKDLER---MDAEGLE-GPNMFEALVFLATHFKNHKKFEEAEVYCTR 543
OsCDC23_1           GMLGQSEEAAFYYKKDLER---MEVEERQ-GQNFVEALLFLAKHCKSIGRFEEAEHYCTR 561
OsCDC23_2           GMLGQSEEAAFYYKKDLER---MEVEERQ-GQNFVEALLFLAKHCKSIGRFEEAEHYCTR 475
SbCDC23             GMLGQSEEAAFYYKKDLER---MEVEERQ-GQNVVEALLFLAKYNKSIGKFEEAEDYCTR 564
SmCDC23             SQLGQADQASYYYKKNLER---LEADQSE-GQDVVDALLFLATHSKNQGFLDDSEMYCMR 439
PpCDC23             VNLGRHEQAAFYFRKNLER---MEADQNE-SQEYVDALLFLGNYSKNNGSLEEAEVYCTR 459
CspCDC23            GDGGDLEAAERYYTRLLDF---GAGSKEAAKSSLREIRTLKAAGVAPRGPAGLTPVRPDS 491
CmCDC23             WETGQYDSAAKHYATYLEIRRALRAPHATIDTDEADVVVRMMTYLRQRGELKLANEYLEY 535
VcCDC23             ESRGEPHAAERLFRDSLRR--LERHSHAFLSLSFAAHNFPTSLPRMCLCPLLLLPGQFDR 497
                          . *   .   *               .
```

```
OspCDC23        LLDVSGPARHAAKALLREIHS---MQSLQ------------ 494
MspCDC23        LLDYAGPEKQLAKALLREIHA---LQEAAAERAEDTSMADV 591
VvCDC23_1       LLDYTGPEKETAKSLLRGMRK---AQSGFPSMDIEHLPP-- 577
VvCDC23_2       LLDYTGPEKETAKSLLRGMRK---AQSGFSSIDIEHLPP-- 577
PtCDC23         LLDYTGPEKEMAKNMLRGMR----SESSFPSMDVEHFPP-- 576
AtCDC23         LLDYSGPEKEKAKSLLRGIRM---AQTGFPSMDLEHFPI-- 579
OsCDC23_1       LLDYTGPERETAKSILQGLKR---AQSVLPLMDIDHFAM-- 597
OsCDC23_2       LLDYTGPERETAKSILQGLKR---SQSVLPLMDIDHFAM-- 511
SbCDC23         LLDYTGPEKETAKNLLQGIKR---LQSGFPSMNTDHFAL-- 600
SmCDC23         LLDYGGPAKEEAKALLREIRSVQQHASVLPSMDLEQFTP-- 478
PpCDC23         VLDFGGPVSSYLLSCPRLSK--------------------- 479
CspCDC23        PPDSPGSDMMAGMSPY------------------------- 507
CmCDC23         MTAVESAEKRETMRKLACDAS----DPAL------------ 560
VcCDC23         STAVAALHRTGGDPVAAVRAYWYPVTGGGGGEGPEHGA--- 535


CspCDC16        ------------------------------------------------------------
VcCDC16         ------------------------------------------------------------
OspCDC16        ------------------------------------------------------------
MspCDC16        ------------------------------------------------------------
SbCDC16         MPLSAASILHAFLLTILLGGGATASFPPGDGDGDVNPSTMFTNDTGTSRIADDTERYICY 60
OsCDC16         MPLSAASINRASYQVLLLLAAAAVSTTGGDGNTAPGNATATATTGG-----DDTEMYICY 55
VvCDC16         ------------------------------------------------------------
PtCDC16         ------------------------------------------------------------
AtCDC16         ------------------------------------------------------------
SmCDC16         ------------------------------------------------------------
PpCDC16         ------------------------------------------------------------
CmCDC16         ------------------------------------------------------------


CspCDC16        ------------------------------------------------------------
VcCDC16         ------------------------------------------------------------
OspCDC16        ------------------------------------------------------------
MspCDC16        ------------------------------------------------------------
SbCDC16         LCTGRNPLLIRYCPIYWDECHLVCYADDAPAATAA--------AIPAAPLPAASANPSGV 112
OsCDC16         LCTGRNPILIRRCPIYWDYCHLNCFDDAPSTAAAADDVAAVPVASPAAPARRVGGVPRET 115
VvCDC16         ------------------------------------------------------------
PtCDC16         ------------------------------------------------------------
AtCDC16         ------------------------------------------------------------
SmCDC16         ------------------------------------------------------------
PpCDC16         ------------------------------------------------------------
CmCDC16         ------------------------------------------------------------


CspCDC16        ------------------------------------------------------------
VcCDC16         ------------------------------------------------------------
OspCDC16        ------------------------------------------------------------
MspCDC16        ------------------------------------------------------------
SbCDC16         HDDDECYVMKLYRNGSYTIVTRLGCARIATCLLSCGGGDMADSDRKALQGTATKTAAPTA 172
OsCDC16         LEDEECYVMKLYENGSYVIVTTLGCSQTASCLLSCGGGDLAADGEEALA---------AA 166
VvCDC16         ------------------------------------------------------------
PtCDC16         ------------------------------------------------------------
AtCDC16         ------------------------------------------------------------
SmCDC16         ------------------------------------------------------------
PpCDC16         ------------------------------------------------------------
CmCDC16         ------------------------------------------------------------


CspCDC16        ------------------------------------------------------------
VcCDC16         -------------------------------------------------------MEALC 5
OspCDC16        ------------------------------------------------MSR-ERALDAAR 11
MspCDC16        ------------------------------------------------MTRCHHDLDATI 12
SbCDC16         TTAAVQGSLTPLLADFQRCGTQVTATPSLPADAAAEPSPKRRRIGEAA-DPPPEMREEAL 231
OsCDC16         HPAGAVGVSPPWRMWDTKFGFPPAAPP--TTAAAAQKNPKRRREAEAEGEVAAEMREEAV 224
VvCDC16         ------------------------------------------------------MREEEI 6
PtCDC16         ------------------------------------------------------MREEQI 6
AtCDC16         ------------------------------------------------------MREEEI 6
SmCDC16         ------------------------------------------------------MEN--- 3
PpCDC16         ------------------------------------------------------MREGEV 6
CmCDC16         ------------------------------------------------------------


CspCDC16        -QLRSVVHDCLAKHMYEAAAFFADKLVTLSGYSPAEVYTL--AQAFFCSRQFRRCLQLLR 57
VcCDC16         SRLRGLAQDCIDKHLTASAIFYADKLVTFSNNSPGDVYLL--AQAYFAARQFHRALSLLR 63
OspCDC16        AARDAYATN----GSIVSAIFCADKARALSGDGDEDALAL--AELLRRDGQHRRASAVAS 65
MspCDC16        AQLRDVVEDRLDKHLASSAIFFADKLVTMSGGALGDVFLH--AKALYLGTHYRRAFATLH 70
SbCDC16         ERLRGVVRDCAGKHLYKSAIFLADKVATVTG-DPGDIYML--AQALFLGRQFRRALHLLN 288
OsCDC16         ERLRGVVRDSVGKHLYASAIFLADKVAAATG-DPADVYML--AQALFLGRHFRRALHILN 281
VvCDC16         EKLRGVVRDCLSKHLYSSAIFFADKVAAFTS-DPADIYMQ--AQALFLGRHYRRAFHLLN 63
PtCDC16         EKLRGVVRDCVSKHLYSSAIFFADKVAAFTD-DPADIYMQ--AQALFLGRHYRRAYHLLN 63
AtCDC16         EKIRGVVRDCVSKHLYSSAIFFADKVAALTN-DPSDIYMQ--AQALFLGRHYRRAFHLLN 63
SmCDC16         -KLRAMVRDCLDKHLHASAIFLADKLVTVGG-TEEDVHLH--AQALFQGRQFRRALHLLR 59
PpCDC16         EKLRAVVRDCLGKHLYSSAIFFADKLATLAGNATQDLYMQAIAQALYLGKQYRRALHLLR 66
CmCDC16         MSDILRAREDLRLGRFESAAYNAERLLAGEPENPAAVEVL--AQALLSAGEARRALHRLE 58
                      . :        :* : *::  :              *:      . :*.
```

```
CspCDC16            STEL--------------------IEKDLR--FRYLAARCLAECKEWEECLSVLGG---- 91
VcCDC16             NAGV--------------------VELGAE--FTYLAGCCLAEAGDWEEVVALLGD---- 97
OspCDC16            VG--------------------EGIRASAR--WRLLRGRCALALRAPEECLRALGEDESG 103
MspCDC16            RGGLIPRKLNPGVENGSLRSRTVDLRAPAENSCRLLAAQCLAAVKDWDGCLAVLGERDDG 130
SbCDC16             NSR---------------------LLRDLR--FRFLAAKCLEELKEWHQCLLMLGD---- 321
OsCDC16             SSK---------------------LLRDLR--FRFLAAKCLEELKEWHQCLIILGD---- 314
VvCDC16             ASQI--------------------VLRDLR--FRYLAAKCLEEQKEWDQCLLMLGD---- 97
PtCDC16             ASKI--------------------VLRDLR--FRYLAAKCLEELKEWDQCLLMLGD---- 97
AtCDC16             ASKI--------------------VLRDLR--FRYLAAKCLEELKEWDQCLLMLGD---- 97
SmCDC16             THGL--------------------LHLHPR--YRYLAAKCLEEIKEWDECLSVLGD---- 93
PpCDC16             RHHL--------------------ITADLR--FRYLAAKCLEEIKEWDECLLMLGD---- 100
CmCDC16             RYRTR-------------------LPLEETVFIRLIEARCLAELRLYEDQVAIFEE---- 95
                                            :          : . *       .  :  :

CspCDC16            -------------------------LDAEEPEQLQLPMPRSTVPL-------------G 112
VcCDC16             -------------------------DEAMQEPQAYEDSVLEPPGG--------------G 118
OspCDC16            -----------------------TEEGVGGAARETEEARATRLA---------------D 125
MspCDC16            DSKSLIEETDTRGEQNPSLPGCLTSARSGVVRQRTKASRVEQLDRTQGHGSRKREYELRD 190
SbCDC16             -----------------------AKVDEHGNVLDQDDGSDFYFDK-----------DAED 347
OsCDC16             -----------------------AKIDEHGNVVDQDDGSDIYFDK-----------DAED 340
VvCDC16             -----------------------AKVDEHGNVNDTKDCNVMYLDK-----------EGED 123
PtCDC16             -----------------------AKVDEHGNVYDTKDCNVMYLDK-----------DSED 123
AtCDC16             -----------------------AKVDDDGIVYDAKDGNVIDFDK-----------DGED 123
SmCDC16             -----------------------YEVDQHGNYPMKDDIDPE---------------SGHQ 115
PpCDC16             -----------------------SEVDEEGNLLVMDDQDTELLDK-----------NIEE 126
CmCDC16             ---------------------------RGLPSTTGERDAARGNANP--------------- 114

CspCDC16            SGINYFSVVCLLRGRVHDALENFPRAVKWYQAALKADPFNYEAFQALVGSHKLSNAEELE 172
VcCDC16             AGVRVQAGMCCLRGRAFEALENRCRAAVWYQVALQLDPYCYDAFAALLEGHLLSNEAEVV 178
OspCDC16            GNRADAAAMCALRGRAYDAMENRARATRAYALALRFDPMCYEAYDAVLSGHALSESEEGA 185
MspCDC16            GSVSIRASLCFMRGKAHGALENWKSAELWCKEALTLDPYCFEAFDLLISSHLLSVGEEDR 250
SbCDC16             REINIKSALCFLRGKAYEALDNRDLARQWYKAAVKADPLCYEALECLVDNYMLTCEEESE 407
OsCDC16             HEINIKAAICFLRGKAYEALDNCDLARQWYKAAVKADPLCYEALECLVDNYMLTCEEESE 400
VvCDC16             REINISSAICFLRGKAYEALENRAQARLWYKAAIKADPLCYEALECLIENHMLTSEEEAS 183
PtCDC16             REINISSAICFLRGRAYEALENRALARHWYKAAIKADPLCYEALECLIENHMLTFEEVX- 182
AtCDC16             REINISSAICFLRGKAYGALQNRSQARQWYKAAIKADPLCYEALECLIESHMLTSEEESS 183
SmCDC16             AGINIAAALCLLRGRACEALENRTRALCWYKASLRVDPYCYEAYEHIVDNHMLSSEKEVA 175
PpCDC16             REISIGAAVCLLRGRAFEALENRARALRWYKAALKADPYCYEAFEHLIDNHMLTSEEESV 186
CmCDC16             ----LIAAYYVLVGDAYEALENPQKASECYIAALQADESCVEALERLFYSNAVVASVPLG 170
                          :    : * .  *::*   *      ::  *    :*   :. .  :

CspCDC16            LVNSLDIPPQQG--WLKLLYT-----------------------------SRCKNKAGV 200
VcCDC16             LVDRLRGALAEGDRWLGLLMR-----------------------------AKCKKVR-- 206
OspCDC16            LVDSLVVNEKNA--WIKEVYAVLSN---------------------VRDVESELLRDS- 220
MspCDC16            FLSSLQIRPEDK--WVPSLYGTICHSGFFSSAEGSSCVASSHGHMNQATDVRCNLPVESD 308
SbCDC16             LLSSLQFGEEDG--WLSTFY--------------------------------SCLITKHE 433
OsCDC16             LLSSLKFGKEDG--WLSAFY--------------------------------SCLIRKHE 426
VvCDC16             LLSSLQFAPEDG--WLSSFY--------------------------------SCLIKKYD 209
PtCDC16             --------------------------------------------------------LSQYD 187
AtCDC16             LLSSLQFSPEDG--WLSSFY--------------------------------SCLIKKYD 209
SmCDC16             FLSSLKFDADDR--WLSLLY--------------------------------SCQAKKYG 201
PpCDC16             LLSSLKFDPEDR--WLSLLY--------------------------------SCRAKKYG 212
CmCDC16             ALPPSTSSGAQHKALSVDQIA-------------------------------LQALWPFE 199

CspCDC16            GLAPMALSPVHEEEKSTPQAAAAAAAGWGLHDNLDVIACRAEWLYHRGAYAECYTLTASA 260
VcCDC16             --QQQQQQGKGEGVQSNGGNGSSGSFGCGLGGNVDVIACRAELLFHRGDYEAAYTLTRPV 264
OspCDC16            --ALGEEDASSQPTTSKSASD-------VLKTSGDVRIARARRLYDRGEFAACYTELRLQ 271
MspCDC16            RDVCNACDVSNIPDTLHSQTSGTQPELFALKYNSEVILARSERYFNRGDYQRCYDTIQEL 368
SbCDC16             KEDLVEAKFKKLEQEACSISSSSG--ERMKNNIDVLACKAEYHHQSGEYQKCFQLTSSL 491
OsCDC16             KEYIVEAKFKEFERESCSISSLSSG--LTLKNNIDVLACKAEYYHQSGEYQKCFELTSAL 484
VvCDC16             KETVVEAKFTELEKQSCNINPSDPSFICTLKNNTDLLACKAEYYHQCGEYQKCFELTAIL 269
PtCDC16             KECVIEAKFREVEKESCNSNPSSPSFMHTLKNNTDLLACKAEYFNQCGEYQKCFELTSDL 247
AtCDC16             KESTVELKFKKLENETSG--SVSGSSMITLANNTDLLACKAEYYHQCCEYQKCFELTAAL 267
SmCDC16             QISALESKLSELEREPQK----HSNVGLSLKDNNDVLACRADYLYHRGEFQLCYDITKTL 257
PpCDC16             QVSVIEEKFQELEKEPDESRPEEERVGCSLKDNNDIIACRADYLYHQGEFQRCYDTTKAL 272
CmCDC16             TPFPKDSSPSKVYLMLANDLRFDDWRKRWSLVPCFFLTMVATRFWRLGFCQEAYRLVSFL 259
                           .                           .    :           .:

CspCDC16            LE--RDPYATECLPVHLASALELRK-----KNELFIQGHKLVEEHSDRAVSWFAVGCYYM 313
VcCDC16             LCCGRDPYALQLLPVHLAAATQLAAYGGGARADLFLLGHRLTEEHPELAVSWYAVGCYYL 324
OspCDC16            YD--VEPSRLDGMPLYFATLVELGK-----KNDLYLLAHSLVDEYPKKALTWFGIGCYYM 324
MspCDC16            LA--NEPTKLAAMPCYLAVTVELRL-----KTKLYLCAHKLVEEYPAKAISWFAVACYYY 421
SbCDC16             LE--RDPFHLKSTLVHLATALELGH-----SNDLYLLACNLVKDYPQKALSWFAVGCYYY 544
OsCDC16             LE--RDPFHLKCTLVHLAAAMELGH-----SNDLYILACNLVKDYPQKALSWFAVGCYYY 537
VvCDC16             LE--KDPFHMKCTLVHLAAAMELGH-----SNELYLMACNLVKDYPQKALSWFAVGCYYY 322
PtCDC16             LE--KDPFHLKCTLVHLAAAVELGN-----SNELYLMASNLVKDYPQKTLSWFAVGCYYY 300
AtCDC16             LE--KDPFHLKCTLVHLAAAMELGN-----SNELYLMACNLVKDYPSKALSWFAVGCYYY 320
SmCDC16             LE--KDPYHLKCMPLHLGAALELGR-----KNELFLRAHNLVQEYSQRPIAWFAVGCYYY 310
PpCDC16             LE--KDPYQLNCMPFHIASALELGR-----KNDLFLRAHNLVQEYPQKAISWFAVGCYYY 325
CmCDC16             VAERKELELKRSFPLFVALLAHHQD-----LITLFRYAHQLVQEFPRAAESWYAVGMYYF 314
                         :         ...   .           *:  .  *..:..  . :*:.:. **
```

```
CspCDC16        CSQQYEAARRYFGKATALDRAFAPAWVAFGHAFAAQDESDQAMAAYRTAHRLFPGLHAPL 373
VcCDC16         AARQPEAARRYLGKATQLQKGFAPAWLAYGHAFSAQDERDQGFFSRTPRTQLQAVSYRPS 384
OspCDC16        ATKQFDQARKYFSKATTMDASFVQAWIGFGHAFAAQDESDQAMAAYRTAARLFSGTHVPV 384
MspCDC16        CTRQFDSARRYFGKATILEATFVPAWLGFGHAFAAQDESDQAMAAYRTATRLYPGCHLSL 481
SbCDC16         CIKKYDQARRYFGKATGLDGTFPPAWIGTGIAYAAQEEGDQAMAAFRTAARLFPGCHLPT 604
OsCDC16         CIKKYDQARRYFGKATGLDGTFPPAWIGTGIAYAAQEEGDQAMAAFRTAARLFPGCHLPT 597
VvCDC16         CIKKYDQSRRYFSKAANLDGTFPPALIGCGNAYAAQEEGDQAMLAYRTAARLFPGCHLPT 382
PtCDC16         CIKKYDQSRRYFSKATSLDGTFAPAWIGFGNAYAAQEEGDQAMSAYRTAARLFPGCHLPT 360
AtCDC16         CIKKYAEARRYFSKATGIDGSFSPARIGYGNSFAAQEEGDQAMSAYRTAARLFPGCHLPT 380
SmCDC16         CIRQFDHARRYFCKATTLDGAFYPAWLGFGNAYAAQDESDQAMAAYRTAARLFSGCHMPA 370
PpCDC16         CIRQFDHARRYFCKATTLESSFAPAWLGFANSYAAQDESDQAMAAYRTSARLFAGCHLPA 385
CmCDC16         ASGKYDASRAYFQKATLLNSNLAYVWVAYGHAFAAVDDSEQALAAYRTAMRLRPNDPTPL 374
                .   :  :* *: **: ::  :  . :. . :::* :: :*.: :  .  :* .    .

CspCDC16        MGMGQEYQRMNNLGLAEQCFSQAARLCP------------------------SDPLVANE 409
VcCDC16         QPAVQILSSSS--CACVTAVRQAYNICP------------------------DDPAVCHE 418
OspCDC16        MSIGIEYQRTNNLSLAYQFFRKSFEISQ------------------------TDPLLYNE 420
MspCDC16        MCIGMEYHRTNNFSLAGQFLSRARHLRP------------------------ADPLVYNE 517
SbCDC16         LYMGMQYVRMHNFKLAEQFFTQAKSICP------------------------SDPLIYNE 640
OsCDC16         LYMGMQYLRMHNFKLAEQFFTQAKSICP------------------------SDPLIYNE 633
VvCDC16         LYIGMEYMRTHSFKLAEQFFMQAKTICP------------------------SDPLVYNE 418
PtCDC16         LYIGMEYMRTHSYKLAEQFFMQAKTICP------------------------SDPLVYNE 396
AtCDC16         LYIGMEYMRTHSYKLADQFFMQAKAICP------------------------SDPLVYNE 416
SmCDC16         LCIGMEYLRTNNLNLAEQFFMQAKGICP------------------------TDPLVYNE 406
PpCDC16         LCIGMEYLRTNNLNLAEQFFLQARSICS------------------------TDPLVYNE 421
CmCDC16         LHVGMEFARQNHLAIARNFFERAAAAAAVVVAPVLTSSADSDADEDALANGIERSRPWNE 434
               .   . ::                               .   :*

CspCDC16        LGVLAYRNRQYEVAAGWLRRALS------------------------------------- 432
VcCDC16         LGVLMYKCGQTAAAAMWLDRALQ------------------------------------- 441
OspCDC16        YGVLLYREGQYESAAQHFERALE------------------------------------- 443
MspCDC16        LGALAFHNGDHVSAISHLEKAIA------------------------------------- 540
SbCDC16         AGVVAYNMKEYRKAVQLFELTLK------------------------------------- 663
OsCDC16         MGVVAYNMKEYQKAVQWFELTLE------------------------------------- 656
VvCDC16         LGVVAYDMKEYNKAVWWFQKTLS------------------------------------- 441
PtCDC16         LGVVAYNMKEYNKAVLWFEKTLK------------------------------------- 419
AtCDC16         LGVVAYHMKEYGKAVRWFEKTLA------------------------------------- 439
SmCDC16         LGVMAYRNREYEEAARWLRKALVLV----------------------------------- 431
PpCDC16         LGVLAYRNRDYDTASRWLRKALQLV----------------------------------- 446
CmCDC16         LGVLCYRDGEYAEAVAYFQKAARPLQAYKKAWSMRLGLDQRYRISDGGGGFAPEKQAEAA 494
                 *.: :   :   *   :  :

CspCDC16        -------------------LVPGGRPTPSWEA-----------------TLVNLGHTLRK 456
VcCDC16         -------------------LLPGGRPTVHWEA-----------------TLVALGHCMRK 465
OspCDC16        --------------------LAPTQMTSRWES-----------------LIVNMAQALRK 466
MspCDC16        --------------------LIPQPVTATWEA-----------------ILVNLAHSNRK 563
SbCDC16         --------------------HTSSSLNEMWEP-----------------TLVNLGHALRK 686
OsCDC16         --------------------HTSSSLNEMWEP-----------------TLVNLGHALRK 679
VvCDC16         --------------------HIPSSLSEMWEP-----------------TIVNLAHAYRK 464
PtCDC16         --------------------HIP-SLSQLWEP-----------------TVINLAHAYRK 441
AtCDC16         --------------------HIPSALTESWEP-----------------TVVNLAHAYRK 462
SmCDC16         -------------------QETRNSLTLCWEP-----------------TVVNLAHTLRK 455
PpCDC16         -------------------PPP---LTEAWES-----------------TVVNLAHSLRK 467
CmCDC16         VQRPVLQQSGIENSQSTSSDTNAPSATSVWCDPTSTFYGCQEARSLLATICSNLGHALIR 554
                          .  *                       :.:   :

CspCDC16        LRQWDAAIECYLQALGLKPGQ--------------PGTYSALGYAHHLKG------DYNA 496
VcCDC16         LCRFPAAAECYSAALALAPAS--------------PGTLAALGYVAQLAG------DPRV 505
OspCDC16        VGRYDDAIAYFEYALSITPRA--------------ASTYAALAFTYQVKSRCTEPRALGL 512
MspCDC16        LNNFDEAIFWYEQALSLAPRN--------------ASTYTALGFTHQLKGNFQSR--MEK 607
SbCDC16         LKEYQKAVSYYEKALTLPTKS--------------LSVFAGLAYTYHLMD------DFEA 726
OsCDC16         LKKYQKAISYYEKALTFQTKS--------------LSAFAGLAYTYHLMD------KFEA 719
VvCDC16         LKMYHEAISFYEKALTLSTRS--------------LSTYAGLAYTYHLQD------NFPA 504
PtCDC16         LKIYHEAISCYERALALSTRS--------------LSTYAGLAYTYHLQD------NFTA 481
AtCDC16         LRKDREAISYYERALTLSTKS--------------LSTYSGLAYTYHLQG------NFSA 502
SmCDC16         LKLYPEAISMYEKALALCPRG--------------ATTYAALGFTHHLQG------STGI 495
PpCDC16         LKSYPEAISMYERALSLFPRG--------------ASTYAALGFTYHLQG------KTGK 507
CmCDC16         LQAIDLAAEALEEALLVGHAVRLAPGCAGSDALARADTLSALGYVEHVRG-----SVQRA 609
                :     *      ** .                    . :.*.:. :: .

CspCDC16        AIENYHKALGLRPEDVFTAEMLAEAM---------------------------------- 522
VcCDC16         AVEHYHAALALRPDDPFTTDMLRLALQVYGG----------------------------- 536
OspCDC16        AIEYYHKALSLRADDVFSQHHLELALIDQSAITMPRHEQVDWNVDFPTSDDMAVTPELGG 572
MspCDC16        AIECYHKALSLKPNDDFAQEMLTLALIDQCAVTMPPYNFVAY------------------ 649
SbCDC16         AINYYHKALWLKPDDQFCTEMLTDALGSICQARRRIV----------------------- 763
OsCDC16         AITYYHKALWLKPDDQFSTDMLTLALESSCQITARTR----------------------- 756
VvCDC16         AITYYHKALWLKPDDQFCTEMLTLALVDEARRGLDPRNESR------------------- 545
PtCDC16         AITCYHKALWLKPDDQFCTEMLSLALVDEGRRGIDPKIE--------------------- 520
AtCDC16         AISYYHKALWLKPDDQFCTEMLNVALMDECQNGVDSKVELC------------------- 543
SmCDC16         AIDFYHKALGLKPDDTFTAEMLTAALTEECLRPSFA--KELTALH--------------- 538
PpCDC16         AVDYYHKALGLNPHDTFTAEMLTSALQEECLRLSSAPDAEFYSLSGMPHIS--------- 558
CmCDC16         AQLYHEAARELALAGINGSTLLLDALLERAVDELAMQSISEIATTE-------------- 655
                *   :. *  *   .      *  *:
```

```
CspCDC16        -----------------------------------------------------------
VcCDC16         -----------------------------------------------------------
OspCDC16        AQRDRDATPDQHGVFPTPSPQSFRGTPTMGRTPFSTRGDASEMDQSVDMDQSVDMDESD 631
MspCDC16        ---------DAHQPLPVKSER---------RS--------------------------- 663
SbCDC16         -----------------------------------------------------------
OsCDC16         -----------------------------------------------------------
VvCDC16         -----------------------------------------------------------
PtCDC16         -----------------------------------------------------------
AtCDC16         -----------------------------------------------------------
SmCDC16         -----------------------------------------------------------
PpCDC16         -----------------------------------------------------------
CmCDC16         -----------------------------------------------------------


OspCDC27        ------------------------------------------------------------
MspCDC27        MTVIVPEQTPAGEAHLEPFLVELVHESLNSYAYSNAAFLCERLHAAAPT----------- 49
CspCDC27        ---MGSPGPPS--AALEEHLVACVQHSLGLYLFDNACFLCERLVAQFPS----------- 44
VcCDC27         ------------------------------------------------------------
CmCDC27         ----MERDLVGRGAALECSLRRALQR---QMPEEHVFFLAERLCAEKST----------- 42
PtCDC27_1       ---------------MEAILVDCVNHSLRHFMHRNAIFMCERLCAEFPS----------- 34
PtCDC27_2       ---------------MEAILIDCVNNSLRHFMYRNSIFMCERLCAEFPS----------- 34
VvCDC27         ---------------MEAILVDSVLGSLRHFLHRNAIFICERLCAEFPS----------- 34
AtCDC27b        ---------------MEAMLVDCVNNSLRHFVYKNAIFMCERLCAEFPS----------- 34
OsCDc27         ---------------METLMVDRVHGSLRLFMHRNAVFLCERLCAQFP------------ 33
SbCDC27         ---------------METLMVDRVHSSLRLFMHRNAVFLCERLCAQFPSEVAPFSPCSST 45
AtCDC27a        --------------MMENLLANCVQKNLNHFMFTNAIFLCELLLAQFPS----------- 35
PpCDC27_1       ---------------MENYLHECVRSSLRAYLYANATFLCERLCAEFPS----------- 34
PpCDC27_2       ---------------MENYLYECVQASLRAFLYANATFLCERLNAEFPS----------- 34
SmCDC27         ---------------MEACLVDSINASLKLYMYRNATFLCERLYAESAT----------- 34


OspCDC27        ------------------------------------------------------------
MspCDC27        -------------EANAHLLATCYYRADQANRAYHTLKG--RTSPKCRYLFALCCVKLRR 94
CspCDC27        -------------EANLFLLATCYHRSNQSFRAYHLLKG--LTGEQSRYLYALCAMQLGK 89
VcCDC27         ------------------------------------------------------------
CmCDC27         -------------AESFALYAQALAQYGRHRQAVDVVAKYWQQDLECRYWYALCCIEANE 89
PtCDC27_1       -------------ETNLQLLAGCYLQNNQAYSAYHILKG--TQMAQSRYLFAISCFQMDL 79
PtCDC27_2       -------------ETNLQLLAGCYLQNSQAYSAYHILKGR-TQMAQSRYLFAISCFQMDL 80
VvCDC27         -------------ETNLQLLASCYLHNNQAYAAYYILKG--TQMAQSRYLFAISCFQMDL 79
AtCDC27b        -------------EVNLQLLATSYLQNNQAYSAYHLLKG--TQMAQSRYLFALSCFQMDL 79
OsCDc27         ------------AETNVQLLATCYLHNNQPYAAYHILKG--KKLPESRYLFAMSCFRMNL 79
SbCDC27         RLVRLDSLWLRVGMTNVQLLATCYLHNNQPYAAYHILKG--KKMPESRYLFATSCFRMNL 103
AtCDC27a        -------------EVNLQLLARCYLSNSQAYSAYYILKG--SKTPQSRYLFAFSCFKLDL 80
PpCDC27_1       -------------ESNVHLLATCYFRSNKAHQAYHVLKG--TKSRQCRYLFALACMEMQN 79
PpCDC27_2       -------------ESNVHLLATCYFRSNKAHLAYHVLKG--TTTRQCRYLFALVCMQMQS 79
SmCDC27         -------------EANLHLLATCYFRSNQAYRTYYLLKG--IKSPQCRYLFALACFEMGN 79


OspCDC27        ------------------------------------------------------------
MspCDC27        LPEAEAAL--CRSPLPAGGRPPATDSSAAERPAANARATSDVPNGAHGLYLLGRVCKET- 151
CspCDC27        LTEAETAL--LPD-----------------------NDASRVPNGGAGFYLLGRIHQLS- 123
VcCDC27         ------------------------------------------------------------
CmCDC27         LAAAYKALSFLLQENANENLTKEVRSSLNNARGLPSERSLSVPWQAEGLYLLGRVFRLSN 149
PtCDC27_1       LNEAEAALCPTNEPG------------------------LEVPNGAPGHYLLGLIYRYT- 114
PtCDC27_2       LNEAEAALCPPNEPG------------------------AEVPNGAPGHFLLGLIYRYT- 115
VvCDC27         LTEAEAALCPVNEPG------------------------AEIPNGAAGHYLLGLIYRYT- 114
AtCDC27b        LNEAESALCPVNEPG------------------------AEIPNGAAGHYLLGLIYKYT- 114
OsCDc27         LREAEEALCPVNEPN------------------------IEVPSGATGHYLLGVIYRYT- 114
SbCDC27         LREAEETLCPVNEPN------------------------MEVPSGATGHYLLGVIYRCT- 138
AtCDC27a        LGEAEAALLPCEDYA------------------------EEVPGGAAGHYLLGLIYRYS- 115
PpCDC27_1       LEEAEAALLSSLEPGA-----------------------ESQPSSAASYYLLGVICKQS- 115
PpCDC27_2       LEEAEATLLNSLEPGA-----------------------ESLPGSATSYYLLCVICKQS- 115
SmCDC27         MAEAEAALQPSDSP-------------------------SEVPNGAAGCYLLGLICRFT- 113


OspCDC27        ------------------------------------------------------------
MspCDC27        GRDKAAAAHFADALALDPFMWCAYEELCALGAEAEAEAATEAALRSADRYPKLADLGKFG 211
CspCDC27        NRHSAAIAYYSTALQLDPMLWSAFEELCGLGADHEAG-----------QYLAAAGAAGTA 172
VcCDC27         ------------------------------------------------------------
CmCDC27         TRVDQAAQCFRRALEIDPFLWCCIEELSSLYR---------------------------- 181
PtCDC27_1       DRRKSAIHHFKQALSIDPLFWAAYEELCILGAAEEAAAVF-------------------- 154
PtCDC27_2       DRRKSAIHHFKQALSIDPLFWAAYEQLCILGAAEEAAAVF-------------------- 155
VvCDC27         DRKKSAVHHFKQALSLDPLLWAAYEELCLLGAAEEATAVF-------------------- 154
AtCDC27b        DRRKNAAQQFKQSLTIDPLLWAAYEELCILGAAEEATAVF-------------------- 154
OsCDc27         GRVEAAAEQFVQALTLDPLLWAAYEELCILGVAEDANECF-------------------- 154
SbCDC27         GRISAAAEQFTQALTLDPLLWAAYEELCILGIAEDTDECF-------------------- 178
AtCDC27a        GRKNCSIQQFRMALSFDPLCWEAYGELCSLGAAEEASTVF-------------------- 155
PpCDC27_1       DRRQGAIGHYTQALSLDPFLWSAYEDLCGLGAEEEAASVF-------------------- 155
PpCDC27_2       GRRQAAIGHYTQALSLDPFLWSAYEDLCGLGADEESVPVF-------------------- 155
SmCDC27         DRRQAAIAHYTQALSVDPFFWSAYEDLCLLGVEEDPLSQS-------------------- 153
```

```
OspCDC27        ------------------------------------------------------------
MspCDC27        ASFGGAAESMSFGTYGGETTSAATNSEGTSGLSGGGGGGTHSGASSNPNHALGGVLGTVA 271
CspCDC27        AAATGAAG--------------PAATAGGAPHSAMGGGGMPSTSTSTFQHGVD------S 212
VcCDC27         ------------------------------------------------------------
CmCDC27         ------------------------------------------------------------
PtCDC27_1       -------------------DEAAALCIQKQHMNHASASQNLSISNEDRNLVSARNFGLED 195
PtCDC27_2       -------------------DEAAALCIQKQYMNCASASHNLSISNEDHNLVSARNFGLED 196
VvCDC27         -------------------GEAAALCIQKQHLHHGLASQNLQTSIEDRNLVSGRNLSSED 195
AtCDC27b        -------------------GETAALSIQKQYMQQLSTSLGLNTYNEERNSTSTKNTSSED 195
OsCDc27         -------------------SEATALRLQQELTSTSNVEK-SNFVNENRFLSSNVSASFGD 194
SbCDC27         -------------------SESTALRLQQEHTSTSTLVK-SNFANENRVLSSRVSASLGD 218
AtCDC27a        -------------------GNVASQRLQKTCVEQRISFSEGATIDQ----ITDSDKALKD 192
PpCDC27_1       -------------------SDAASFQLQKVEFLGQLNAMQNEHDDNDYSLRTPVS----S 192
PpCDC27_2       -------------------SDTVKSQLQKIESVRQSNAVKHESFEHDFSLRAPAS----S 192
SmCDC27         -------------------QWEVKSQYT--------YNTPSPSVSQLVTTKSTVA----P 182

OspCDC27        ------------------------------------------------------------
MspCDC27        EGTPTVEKSLGFAKPPHSGLPSASGPPVPMSTGGESAYYGAYGDEKTPAPDSTRGLMPPP 331
CspCDC27        GGTPSPGS---YVTPSPGGRPSAAPPPPAPKVGGPAAARPSWPATNTPAP---------- 259
VcCDC27         ------------------------------------------------------------
CmCDC27         ------------------------------------------------------------
PtCDC27_1       GSPRQSKHPQGNNLRDIPGNYHGATTLGGSASQPSNGGLPNLSFYNTPSPMATQLSSVAP 255
PtCDC27_2       GSPRQLKHLQGNNLRDIPGNYHGASTLGGPVSQPSNG-------------FILQLSGVAP 243
VvCDC27         VSPRQLKHIHANNLREIPGNYHGAAMSGATASQSLNSGPSSTAFYNTPSPMVAQLSGVAP 255
AtCDC27b        YSPRQSKHTQSHGLKDISGNFHSHGVNGGVSNMSFYN---------TPSPVAAQLSGIAP 246
OsCDc27         -SPKQIKQLHANTTAEVSGYPHVKSTALHMQNGAPSN----LSQFDTPSPTSTQASGIAP 249
SbCDC27         ISPKQIKQLHANNIAEVSGYPHTSS--------------------------------IMP 246
AtCDC27a        TGLSQTEHIPGENQQDLKIMQQPGDIPPNTDRQLSTNG----WDLNTPSPVLLQVMDALP 248
PpCDC27_1       NGSPKHRRLHSGGAVSDAGSAFMTPTSEGMCTPAPVP--------------NLG--LGLP 236
PpCDC27_2       NGSPKHRKLHSGGVISDAGSTVMISTSEGVSTYASVQ--------------NLG--LGPP 236
SmCDC27         VHPQRRKFLDEGKLRKVSGRLFTEPPRRSLRLSAESP--------------STINVCSIP 228

OspCDC27        ------------------------------------------------------------
MspCDC27        PPSTATAAGVPADDLVTPAPIGGEADHGKPPGPPGGPGGGGGGGGGGGGRGDGGRRKFVD 391
CspCDC27        LPVTAG-------------------------GSLAMPGTSGGGGGGGG-----AQRKFVD 289
VcCDC27         ------------------------------------------------------------
CmCDC27         ------------------------------------------------------------
PtCDC27_1       PPLCRN---------------------------------MQPNGSNLSMPGFDNSARSTL 282
PtCDC27_2       PPLCSN---------------------------------LQPNCSNP----------STL 260
VvCDC27         PPLCRN---------------------------------VQQNGLNPSTVGNDSSPRSTV 282
AtCDC27b        PPLFRN---------------------------------FQPAVANPNSLITDSSPKSTV 273
OsCDc27         PPLFRN---------------------------------MHAYQNTAGG---NAPSKPKV 273
SbCDC27         PPLFRN---------------------------------VHAYQNTVSG---DAPTKQKT 270
AtCDC27a        PLLLKN---------------------------------MRRPAVEGSLMSVHGVRVRRR 275
PpCDC27_1       GPGRST---------------------------------NPSASPAGGGAGEGVGRGGGG 263
PpCDC27_2       GPGRSN---------------------------------NPGANATGGGSGDGIGRGGGG 263
SmCDC27         SPVAVS---------------------------------TPTSTASVRVSSNSSVRVSNS 255

OspCDC27        ----------------------------------------------MQHVGASPDDDGAH 14
MspCDC27        EGKLRKVSGQLFQESANAGSGAVRRSSRLAAQTGGGGGAGLDFSTPAMEAGSAEGAEGAD 451
CspCDC27        EGKLRKVSNKLFADPASMLK-------ELRWQEGEAGGG-------ASGSGASGGGGGGA 335
VcCDC27         ------------------------------------------------------------
CmCDC27         --YIDYFVPEMFGISG------------------------------KEEQVFQSGRSSLR 209
PtCDC27_1       NSNMQAPRRKFVDEG-----KLRKISGRLFS---DSGPRRSTRLAA-EAGSNQNTSSTLV 333
PtCDC27_2       ASLIYF-----------------QISGRLFF---DSGPRRSTRLAA-EAGANQNTSATLV 299
VvCDC27         NPTIQAPRRKFVDEG-----KLRKISGRLFS---DSGPRRSTRLAG-EAGANTNPSGTTV 333
AtCDC27b        NSTLQAPRRKFVDEG-----KLRKISGRLFS---DSGPRRSSRLSA-DSGANINSSVATV 324
OsCDc27         NAPNLTLRRKYIDEAGLKK-----VSGRLFNQSSDSVPRRSARLSR-DTTINSNSNISQF 327
SbCDC27         NGVNQPLRRKNIDEARLKKGRRQWVSGRLFN--SDSIPRRSERLK--DTATNSNSNTSQF 326
AtCDC27a        NFFSEELSAEAQEESGRR--RSARIAARKKNPMSQSFGKDSHWLHLSPSESNYAPSLSSM 333
PpCDC27_1       TVGYAQQRRKFVDPD-------------------GKFRKSLRSLSMRPGYTQIPGRLFEQ 304
PpCDC27_2       TAGYAQQRRKFVDPD-------------------VKFRKSLRSLSVRPGQSQASRRVVEQ 304
SmCDC27         RKIISGSAEGPLDDG-------------------RRVYESLETL----GSDEMSNTSSQQ 292

OspCDC27        LSRVP-------EDGEASYD--------------------------TGHYDVHRDQ---- 37
MspCDC27        PTTSPPHHRARGRGGRSTHNPRGSLDGGSRPPLPL------PPTSSSGHGGMNAGMNAGY 505
CspCDC27        AGAAG--------GSLADVA---------------------------ALHGVPRGQ---- 356
VcCDC27         ------------------------------------------------------------
CmCDC27         LDNCILR----------------------------------------------------- 216
PtCDC27_1       AGNGTNNSPKYLGGSKFSSMAIRSVTVRKGQSWVNENYDEGIRNEAFDDSRANNTSSN-C 392
PtCDC27_2       AGYGTNNSSKYLGGSKLSSMAIRSVTVR-------------IRNEAFDDSRANNTSSN-C 345
VvCDC27         AGNGTIHSSKYLGGAKSSSAAFRSVTVRKGQTLANESFDEGTRQEVFDDSRSYISAATST 393
AtCDC27b        SGN-VNNASKYLGGSKLSSLALRSVTLRKGHSWANENMDEGVRGEPFDDSRPN------- 376
OsCDc27         GGNGTDHSS----GNRYH-----------------------VVDEMWTDNVTSTS----- 355
SbCDC27         GGNGAGHSS----GSRYE-----------------------VIDEMWTDNISGIS----- 354
AtCDC27a        IGKCRIQSSKE------------------------------VIPDTVTLNDPATT----- 358
PpCDC27_1       GAPRRSTAT---------------------------------LHYEKLFDSFEVS----- 326
PpCDC27_2       VGPRMSTR----------------------------------LNMIGDCNTAQLQ----- 325
SmCDC27         SSP---------------------------------------------VIDEELR----- 302
```

```
OspCDC27        -GTWSWQPSFAA----------------RTAEGTVLAFKALRPLAEGLRHLAMYRCEDAL 80
MspCDC27        GSNGAGRAGYASNGTALHAGHGGYVGCGRFAEGAAATAALLRPLADGLRTFSMFRCEDAL 565
CspCDC27        ----------------------------RSQEGQQQALPLLQALGEGYRLLCMYRCQEAV 388
VcCDC27         -----------------------------MAESRTALLQLLAPLMEGVRHLAAYRCSEAL 31
CmCDC27         ----------------------------WKTRSSGSPQDNFQALATVRALVEGYHCREAV 248
PtCDC27_1       SLSLTGDSRSLETEVATMPVGGVIASPSCILSGALEILGLLRTLGEGYRLSCMYRCQDAL 452
PtCDC27_2       SSSPPGDSRPLETEVATMPVGGVIISASCILNGALEILGLLRTLGEGYRLFCMYRCQDAL 405
VvCDC27         STSTSGDPKSLEQDEATMTIGGVITNTSKIINGAAEVLNLLRTLGEGYRLSCMYRCQDAL 453
AtCDC27b        TASTTGSMASNDQEDETMSIGGIAMSSQTITIGVSEILNLLRTLGEGCRLSYMYRCQEAL 436
OsCDc27         SSTSIVDGRYPEQEKSER----VLSQDSKLAIGIRELMALLRTLGEGYRLSCLFKCQEAL 411
SbCDC27         SSVSATDGRSFEQDKAER----ILLQDSKLALGIRELLGLFRTLGEGFRLSCLFKCQEAL 410
AtCDC27a        SGQSVSDIGSSVDDEEKSNPSESSPDRFSLISGISEVLSLLKILGDGHRHLHMYKCQEAL 418
PpCDC27_1       GGVDEGLSNSKLEG----AEEEAPLSARCISRGASELFQLLRILGEGYRHLCMLRCQEAV 382
PpCDC27_2       QPCPTSSVGATMVGTVARSDEERSLSARCASRGALELFQLLRILGEGYRHLCMLRCQEAV 385
SmCDC27         RSVSVGFVS----------------RGSKLGEGATELLALLKVLGEGFKHVCMYESQEAL 346
                                                .       :  *          .. :*:

OspCDC27        RSFEQLTRAQYDTAYVLCAVAKAHAEMVDYPNAARY-FEEARAADPHRLEGLDVYSTVLW 139
MspCDC27        AHLRELPRSQYVTGYVLCLVGRAYAEMVNYPEAQRA-FEWARTVCPHGLDGMEVYSTVLW 624
CspCDC27        DALSRLPPHQYQTGWVLCCVGRAFFEMVDYPEAAKA-FSWARQVDPYRLRGLEVYSTVLW 447
VcCDC27         AALSRLPMSQARTAWVMGAMGRAHFESMNYAKAAQVRFESARQLDRTRVEGMEIYSTVLW 91
CmCDC27         ALIATLPLALQQAPVVLKWQGRAYLDAGELSECARTFEKYLSLNRSGSLDGLEYYSTALW 308
PtCDC27_1       DVYMKLPHKHYNTGWVLCQVGKAYVELVDYLEADRA-FSLARRASPYSLEGLDVYSTVLY 511
PtCDC27_2       DVYMKLPHKHYNTGWVLCQVGKAYVELVDYLEADRA-FSLARRASPYSLEGLDVYSTVLY 464
VvCDC27         DVYMKLPHKHYNTGWVLSQIGKAYFELVDYLGADRA-FSSARQASPYSLEGMDIYSTVLY 512
AtCDC27b        DTYMKLPHKHYNTGWVLSQVGKAYFELIDYLEAEKA-FRLARLASPYCLEGMDIYSTVLY 495
OsCDc27         EVYRKLPEAQFNTGWVLCQVGKTYFELVNYLEADHF-FELAHRLSPCTLEGMDIYSTVLY 470
SbCDC27         EVYRKLPESQFNTGWVLCQVGKAYFELVDYLEADRY-FELAHRLSPLQNS---------- 459
AtCDC27a        LAYQKLSQKQYNTHWVLMQVGKAYFELQDYFNADSS-FTLAHQKYPYALEGMDTYSTVLY 477
PpCDC27_1       QSFSKLPQQHFATAWVLCQVGRAYLEMVNYAEAERV-YSWARRVSPHCSEGMDMYSTALY 441
PpCDC27_2       QSFSKLPQQHFATAWVLCQVGRAYVEMVNYPEAERV-YSWARRVSPHCPVGMDMYSTALY 444
SmCDC27         EAFAKLPQNQYETGWVLCQIGRAYFEMVDYAEAERA-FSWARRVSPYRLEGTDIYSTVLY 405
                     *.     :  *:   .::. :  :   .

OspCDC27        HLKEEVKLSNLAQEVQGIDRLAPQTW-CVLGNCFSLQKEHELALKFFQRAIQLDPKYTYA 198
MspCDC27        HLKKEVELSYLAQECVQLDRLAPQTW-CVLGNCFSLQKEHETALRFFQRALQLDPRCTYA 683
CspCDC27        HCKREVELAQLAQAASSLDRHSPYAW-CAMGNCFSLQKEHETALRYFQRALQLDPTLPYA 506
VcCDC27         HTKREYELSHLAQECVATDRLAPQTW-CVLGNLFSSQKEHEAAIEFFLRAAQVDPTFTYA 150
CmCDC27         HMRRDVELNALARYALERDRFSAATW-CIVGNAFSLQRDTDSAIEFFLRAAQIDPRNPYP 367
PtCDC27_1       HLKEDMKLSYLAQELISTDRLAPQSW-CAIGNCYSLQKDHETALKNFQRAVQLDSRFAYA 570
PtCDC27_2       HLKEEMKLSYLAQELISTDRLAPQSWXCAMGNCYSLQKDHETALKNFQRAVQLDSRFAYA 524
VvCDC27         HLREDMKLSYLAQELISTDRLAPQSW-CAMGNCYSLQKDHETALKNFQRAVQLNSRFAYA 571
AtCDC27b        HLKEDMKLSYLAQELISTDRLAPQSW-CAMGNCYSLQKDHETALKNFLRAVQLNPRFAYA 554
OsCDc27         HLNEEMRLSYLAQDLVSIDRLSPQAWCAVGN-CFALRKDHETALKNFQRAVQLDSRVAYA 529
SbCDC27         -------------------------WMCLG-------HNHNRLVWKLGPHVSSFSYVSVW 487
AtCDC27a        HLKEEMRLGYLAQELISVDRLSPESW-CAVGNCYSLRKDHDTALKMFQRAIQLNERFTYA 536
PpCDC27_1       HMKKDVQLSYLAQDAVAMDRLSPQAW-CVMGNCFSLQKDHETALKFFQRALQLDPNFTYA 500
PpCDC27_2       HMKKDVQLSYLAQDAVAMDRLSPQAW-CVMGNCFSLQKDHETALKFFQRALQLDPNFTYA 503
SmCDC27         HMKKDVELSYLAQEVVSMDRLSPQAW-CVIGNCFSLQKDHETALKFFQRALQLDSHFTYA 464
                                         * .         :: :  :  :    .     .

OspCDC27        HTLSGHEYFANEDFEKSMNCYRAALRLDSRHYNAWYGLGTVYYRQEKYVMSEYHFRYALN 258
MspCDC27        HTLCGHEFFANEDFEKAMGCYRNALRLDGRHYNAWYGLGTVYYRQEKYELSEYHFRHALS 743
CspCDC27        YTLAGHEYFANEDFEKGITCYRNAIRIDPRHYNAWFGMGHIYYRQEKYGMAEYHFRRALS 566
VcCDC27         YTLAGHEYFANEDYDKAAACYRSALKLDPRHYKAMYGLGQIAYRQEKYAEALQNFRLAAG 210
CmCDC27         CTLAGHEYLYLDNYDAAMRCYQDALYRNSRHYNAWFGIGQVYQRQEKFRLAEKHYRIALD 427
PtCDC27_1       HTLCGHEYVALEDFENGIKSYQSALRIDARHYNSWHGLGMVYLRQEKNEFSEHHFRMAFQ 630
PtCDC27_2       HTLCGHEYVALDDFENGIKSYQSALRIDARHYKSWHGLGMVYLRQEKNEFSEHHFQMAFQ 584
VvCDC27         HTLCGHEYVALEYFENGIKSYQSALRIDDRHYNSWYGLGMICLRQEKFEFAEHHFRMAFQ 631
AtCDC27b        HTLCGHEYTTLEDFENGMKSYQNALRVDTRHYNAWYGLGMIYLRQEKLEFSEHHFRMAFL 614
OsCDc27         HTLCGHEYSALEDYENSIKLYRSALQVDERHYNAWYGLGVVYLRQEKFEFAEHHFRRAFQ 589
SbCDC27         N----------------------VQQVDERHYNAWYGLGVVYLRQEKFEFAEHHFRRAFQ 525
AtCDC27a        HTLCGHEFAALEEFEDAERCYRKALGIDTRHYNAWYGLGMTYLRQEKFEFAQHQFQLALQ 596
PpCDC27_1       HTLCGHEYVAMEDFEEGLTCYRKAIRLDSRHYNAWYGLGTIYFRQEKYELAEYHFRRALF 560
PpCDC27_2       HTLCGHELVAMEDFEEGLICYREAIRLDSRHYNAWYGLGTIYLRQEKYELAEYHFQKALH 563
SmCDC27         YTLCGHEYVAMEDFEEGLTCYRNAIRMDGRHYNAWYGLGTIYLRQEKYELAEYHFRRALQ 524
                                       .   : ***:: .*:*    ****   :  ::: *

OspCDC27        INSKSSVLFCYAGMAKHALG-ENSDAMTLLSQAIALDEKNPLARYEMAAVLMSEENYDQA 317
MspCDC27        INSRSSVLFCYLGMAQHALR-RNADALTLLQHAIDLDKRNPLAKYEKASVLLSEDRLEDA 802
CspCDC27        INDRSSVLRCYLGMALHKLK-RSGEALETLGQAIAADPRNPLAKFERAAVLMAEDRWRDA 625
VcCDC27         INPRSSVLRCYVGMSAAKLG-QTPLALEKLQEAIDLDPANPLARFERASVLASLERIGEA 269
CmCDC27         LNSNNSMLWYYLGHVIRVGGGREVDALNALEKALEMNPRNPVARFECCKLYMQIGRLQDA 487
PtCDC27_1       INPCSSVIMSYLGTALHALK-RNEEALEMMERAILADKKNPLPMYQKANILVSLESFDEA 689
PtCDC27_2       INPHSSVIMSYLGTALHALK-RNEEALEMMERAILADKKNPLPMYQKANILVSLESFDEA 643
VvCDC27         INPRSSVILCYLGTALHALK-RSGEALYMMEKAILADKKNPLPMYEKANILLGLDNFDEA 690
AtCDC27b        INPSSSVIMSYLGTSLHALK-RSEEALEIMEQAIVADRKNPLPMYQKANILVCLERLDEA 673
OsCDc27         INPCSSVLMCYLGMALHALK-RNEEALEMMENAIFADKKNPLPKYQKALILLGLQKYPDA 648
SbCDC27         INPRSSVLMCYLGMALHSLK-RDEEALEMMEKAIAADKKNPLPKYQKALILLGLMKYEEA 584
AtCDC27a        INPRSSVIMCYYGIALHESK-RNDEALMMMEKAVLTDAKNPLPKYYKAHILTSLGDYHKA 655
PpCDC27_1       VNSRSSVLHCYLGMALHALK-KNGEALALLEQAIVADPKNPLPKFQRANVLMSEGRCHEA 619
PpCDC27_2       VHSRSSVLHCYLGMALHALK-KNDEALALLEQAIVADPKNPLPKFQRANVLMSEGRYREA 622
SmCDC27         INERSSVLHCYLGMALHALK-RSHEALELLGEAIRADPKNPLPKYQKANVLMSEERYNDA 583
                ::  .*::  * *        .   *:  : .*:  :  **:. :  . :        .*
```

```
OspCDC27        LEELQTLQEIAPKEASVFFLMGRIYKKLGLQEKAMINFSIALDLRPSNADVNSIKSAIEK 377
MspCDC27        LEELERLKEVAPREASVFFLIGRIHKKLGAADAAMVAFSTALDLKPASADVNLIKSAIEK 862
CspCDC27        LAELHALKDLAPREASVLFHMGKIYKKLDMLDEAMACFAHALDLQPPSADTNLIKGAIEK 685
VcCDC27         LAELEALQRMAPGEASVAFQMGKLFKRLNNRTVSKSTYVHVCFGGRTSKSVKVLLSTLST 329
CmCDC27         WKELYQLRNMVPREAAIYYQMGVIARELGLKN-AVELFSIALDLDPKQPLYRQALLSLDE 546
PtCDC27_1       LEVLEELKEYAPRESSVYALMGKIYKRRNMHEKAMFHFGLALDLKPSATDVATIKAAIEK 749
PtCDC27_2       LDVLEELKEYAPRESSVYALMGKIYKRRNMYEKAMLHFGLALDFKPSATDVATIKADIEK 703
VvCDC27         LEVLEELKEYAPRESSVYALMGKIYKRRNMYDKAMLHFGIALDLKPSAADVATIKAAIEK 750
AtCDC27b        LEVLEELKEYAPSESSVYALMGRIYKRRNMHDKAMLHFGLALDMKPPATDVAAIKAAMEK 733
OsCDc27         LDELERLKEIAPHESSMYALMGKIYKQLNILDKAVFCFGIALDLKPPAADVAIIKSAMEK 708
SbCDC27         LDELERLKEIAPHESSMFALMGKIYKQLNILDKAVFCFGIALDLKPPAADLAIIKSAMEK 644
AtCDC27a        QKVLEELKECAPQESSVHASLGKIYNQLKQYDKAVLHFGIALDLSPSPSDAVKIKAYMER 715
PpCDC27_1       LAELEELKELAPRESSVFFLMGRIYKRLDMLERAIHHFCIALDLKPSATDVNLIKTAIEK 679
PpCDC27_2       LAELEELKELAPRESSVFFLMGRIYKRLEMLERAVHHFRIALDLKPSSTDVNLIKAAIEK 682
SmCDC27         LGVLEQLKEVAPRESSVYFLIGKVYKRLGQPESAMYHFCVALDLKPSTADVNLIKNAIEK 643
                   *  *:  .* *:::   :* : ..      :   :  .                :.

OspCDC27        LDSDEVSDEEDI------------------------------------------------ 389
MspCDC27        LHVPDDSEEEDL------------------------------------------------ 874
CspCDC27        LRTPDDNEEEEI------------------------------------------------ 697
VcCDC27         LLGVEQFPNCL------------------------------------------------- 340
CmCDC27         TQSAP------------------------------------------------------- 551
PtCDC27_1       LHVPDELEDSL------------------------------------------------- 760
PtCDC27_2       LHVPDEL----------------------------------------------------- 710
VvCDC27         LHVPDEIEDNL------------------------------------------------- 761
AtCDC27b        LHVPDEIDESP------------------------------------------------- 744
OsCDc27         VHLPDELMDDDD---------------------------------------------DDD 723
SbCDC27         VHLPDELMEDDLRFKAEFEPAPSHKFAFEETMPTTILVLDSYKPDISEYSSSNAGDFESD 704
AtCDC27a        LILPDELVTEENL----------------------------------------------- 728
PpCDC27_1       LPIADDLEVENL------------------------------------------------ 691
PpCDC27_2       LPIADALEVENL------------------------------------------------ 694
SmCDC27         LHVPDESEEENL------------------------------------------------ 655


OspCDC27        ------------------------------------------------------------
MspCDC27        ------------------------------------------------------------
CspCDC27        ------------------------------------------------------------
VcCDC27         ------------------------------------------------------------
CmCDC27         ------------------------------------------------------------
PtCDC27_1       ------------------------------------------------------------
PtCDC27_2       ------------------------------------------------------------
VvCDC27         ------------------------------------------------------------
AtCDC27b        ------------------------------------------------------------
OsCDc27         EI---------------------------------------------------------- 725
SbCDC27         ATRNASPLGGHHDNFNSLRQKMCCTSSSQNTCRTQLLTPKARIWIHEFHGTCKQYVPSCG 764
AtCDC27a        ------------------------------------------------------------
PpCDC27_1       ------------------------------------------------------------
PpCDC27_2       ------------------------------------------------------------
SmCDC27         ------------------------------------------------------------


OspCDC27        ------------------
MspCDC27        ------------------
CspCDC27        ------------------
VcCDC27         ------------------
CmCDC27         ------------------
PtCDC27_1       ------------------
PtCDC27_2       ------------------
VvCDC27         ------------------
AtCDC27b        ------------------
OsCDc27         ------------------
SbCDC27         VISRRVTPLDIHGWIFKS 782
AtCDC27a        ------------------
PpCDC27_1       ------------------
PpCDC27_2       ------------------
SmCDC27         ------------------
```
