# Supplementary figures and images for: Genomic evolution and complexity of the Anaphase-promoting Complex (APC) in land plants
Source: BMC Plant Biol. 2010 Nov 18;10:254. doi: 10.1186/1471-2229-10-254 (PMC3095333; doi:10.1186/1471-2229-10-254)

**Additional file 11: Phylogenetic relationships between Plants and algae.**

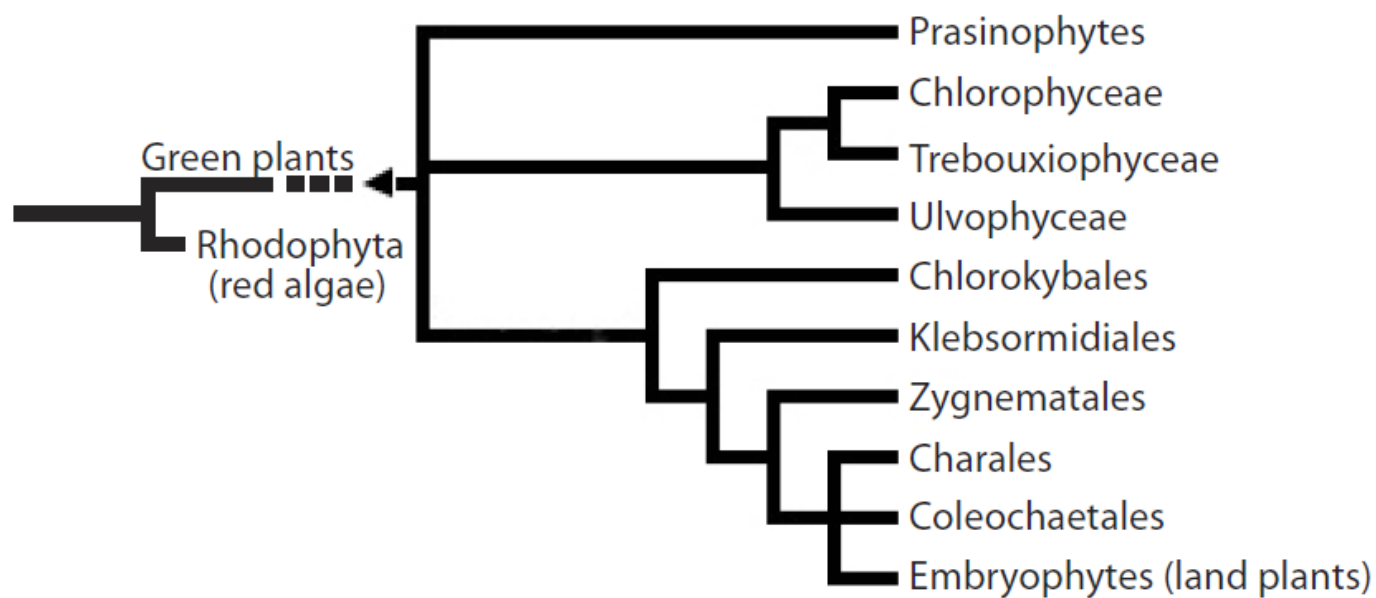

Supplement: Additional file 11 — Phylogenetic relationships between Plants and algae. [file 1471-2229-10-254-S11.PDF]
