# Supplementary material for: Genomic evolution and complexity of the Anaphase-promoting Complex (APC) in land plants
Source: BMC Plant Biol. 2010 Nov 18;10:254. doi: 10.1186/1471-2229-10-254 (PMC3095333; doi:10.1186/1471-2229-10-254)

Additional file 12 – OsCDC23_2 genomic sequence. Exon (yellow boxes), intron and possible exon (gray boxes). Sequencing result - frameshift discarded.

ATGGCTTCCTCCAAGGAAGCCTACCGCGTCGAGCTCCGAGCCGCCGCCCGCCAGCTCGGTGAGCGCGGCCTCTACTCCGCCGCCAAATGGTACGTAGGTACGCCCCCTCCCC
TAAAACCCCTCCTCTAAACCCTAACCCTAACCGCCGCCGCCTCTTCCTCTGTAGGGCGGCCGAGCTGCTCGTCGGCATCGACCCCGACGCGACGCCGGCCCCGTCGTCGGCG
ATGGACACCCCTTCCTCGTCGGGCTCCGGGGGGCACCTGCTGCACCTCCACCGCAGCGGCGGGTCCAGCTTCCGCCGCCGCCTCCGCCCGGGCGCGGCCGGGGAGGCCGGGA
CGCCGCTCGGCGGCGTCTCGTACGTCAGCACTCCCATCCCCGACGACGACGACGCGTTCGATGTCGGGGCTGATAGGTACCTGCTCGCCAAGGCCTAATTCGACTGCCGCGA
GTACCGCCGCGCCGCGCACGTCCTGCGCGGCCAGACCGGCCGCAAGGCCGTGTTCCTTCGCTGCTATGCCCTGTACACGGTGACTTCGGATCCTATTGTTTTTCTTTTTAAT
TATTATTTTGGAATGCGCGGGCGTGTTCCAGTTTGCAGATGTTTGGTATAGCGACTGGTGCTTGTCTGCTTGAGGAAATGTCTCACTGGAGCTGCTGCAAATATTTACGGCT
CCAATTCGCCTATAGAGTGATTCATATTTCTCAACCTGTAGAAACATCTGATTAGTTATCTTGACGTGTCAATGGAGTAATAGTAGTGGGATCTTACTGAAAGAAACAATAG
GAATACATCAAAATTATTGTAAAAATGTAAAAGTAGCTTTATTTGGTATGTTCCCCTTTGTTCATGGTCTGAGCGCTAATCTTTGAAGCCCTAGGGGTGGAGATGCTAGCAT
TTTTGAGTCTTAATTACCCCCCTTGTCCCATAATGATGTACTACTGATTTTTGGCACTTTAAACCTTAATTTGGTAAATGTCTCGACATCTGAAAAAATGTTGAACCCTTTA
TTGTACATAACTGCTTATTTGCTTCGTTCCTAGTTGTTTGGTATCCTTTATGCATTACAGTGATGCTTGATTATTTTTATAGTAAGCGGTAGATGCTGGTTTGCTGCAATGT
TAACATTAGACGAATTTATTGCATGCAAGAATGCATTTTACTAATACAGTGGTTCTGTTGTAATGAAGATTGGAAACATTATATTCTAATATTGAGATGAACGGGCAGTATA
AGGTTTTAACTTATAGTAGTAACACGATTGCCCTTATGCAACAAGGAGGGCTGAGGATCCAGGTCCATAAATGATTTATCTTTTCCTCTTTGCTATTTAATTTGTGATCTGC
TAAAAATAGGAGTATAGGACTCGCCACAGTAATACAGTGTATAATTGTGCGCTTGGGGCTATGTTGCTTTTGGTTGCTAGTGTGCCACTAACGAATAGTGGCTAGTATTGAA
GTATTCAATTTAGAACCATGCTGATTTCCATTTTAGCAGGTCCTTTTATTAGCTGGATTTCTATTTAAGTTGGCAAGCTGCACTGTTAATATGTACAGTTAGTGTCAATCTT
TGCATCAATTTACGTCTCTATACACTAGGCCCTAAACTTATTGGATCGTATAAATGTGGCTAGCAATCTTCCGATCCATGGAAGGATACTATGAATGAGTTATCTTTTCCTC
TACAGGTTATCTAATTTTCTAAATATAGAAATTGTCTCCTGTTTGCTTCGTAGTTTAATTAGCATTTTGAAATTTGATATGTTTGGTGATCATAGTTATTTTCTCTACAATT
TAGGCTGGAGAGAAACGGAAAGAGGAAGAAACAGTCGAGCTTGAGGGATCTTTGGGCAAAAGCAATGCTGTTAATCAGGAACTAGTTGCATTGGAGAGAGAGCTCGCAACAC
ATCGGAGAACTGGTGCTATTGATTCATTTTGTTTGTACTTGTATGGCATTGTTCTACGTGATAAAGGCAGTGAAGCTCTAGCTAGAACAGTTCTGGTGGAATCTGTCAACAG
CTACCCGTGGAACTGGAGTGCTTGGTTAGAATTACAATCTCTCTGCACTAGCAGTGACATTTTGAACAACTTAAATCTCAAGAATCACTGGATGAAAGATTTCTTCCTTGCT
AGTGCACATCTTGAACTAAAAATGCATGAAGAAGCTTTGAAAAGATATGAGCGCTTAATGGGGGTCTTCCGTTGCAGTGACTACATTCAGGCTCAAATAGCTACTGTGCAGT
ATAGTATGAGAGATCTGGATGAAGCTGACATGATTTTTGAAGAACTCCTTAGGACTGATCCTTTTCGTGTGGATTCTATGGACGTTTACTCAAATTTATTGTATGCAAAAGA
AAGCTCGACTGCTTTAAGTTTCCTTGCTCACAGAGTATTTTTGACAGATAAATATCGCCCAGAATCATGCTGCATAATTGCAAATTACTACAGTTTGAAGGGGCAGCATGAA
AAATCAGTTTTGTACTTTCAAAGAGCACTGAAGCTTAATCGAAAGTATCTTTCAGCTTGGACCCTTATGGGACATGAGTTTGTTGAGCTAAAAAATACACCTGCTGCGATTG
ATGCCTACAGGAGAGCTGTTGATATAAATCCCAGAGATTACCGTGCTTGGTATGGTCTTGGTCAGATCTATGAGATGATGGGAATGCCGTTTTATGCAGTTTATTACTTCCG
TAAATCGTCATACCTACAACCTAATGATGCCCGGCTTTGGAATGCTATGGCTCAGTGCTATGAAAGCGATCAACTCCAGATGATTGAAGAAGCCATCAAGTGCTATGAGAGA
TCTGCAAATAATAATGACACTGAAGGAATAGCACTTCATCAGCTAGCAAAGTTACATGGTATGCTTGGACAATCTGAGGAGGCAGCATTTTACTACAAGAAGGATTTAGAGA
GAATGGAAGTTGAGGAAAGGCAGGGCCAGAATTTTGTTGAAGCTCTGCTTTTCCTTGCTAAGCACTGTAAGAGCATAGGCAGGTTTGAGGAGGCAGAGCACTATTGCACAAG
GCTCTTGGATTACACCGGTCCAGTAAGTTCAATTTTACATAAAGTTCCTTTTACCTACTGATGAAAATAACAAAAGATGGAATGGAGCAAATAGACTTCTTATTTATTGGCT
AATGTGTTAATGAACACTTATGTTTGCGTACAGTTCAAATAGACTTATAACTTATTCAATAAGCATTCACTTTGATTTGTTCTTTTAAGAGTGCCTGTTTGCTGTACTCTGG
GTCTAATTGAGGGAACTGGAATATCATGTTTTTCTTTCATAAATAAATCAATATTTGTTTGATCTCTTCTTTTTGATGATTCTGATTCACTACCACTTGTATGTTCTATATT
TACAGGAAAGGGAGACTGCAAAAAGTATTTTGCAAGGGCTAAAAAGATCACAATCGGTACTTCCGTTGATGGATATCGACCATTTTGCAATGTAAATTGGACTTGCAGATAA
GTTATTTAGTTGAAGAACTAAGGTGTTATAAGTGAGATGTACAAGAGAAAACTACCCCTTGCATATTCTTTTCTCCATTTTTTGCATGCATCGAGGAAAGCTACACATTGGT
TTCAAGAGAACTGCTATAATACAGAACAGTCATGAACAAGGGGATTGGTTATCACTCACACCACACCAAGCAAGGAAAAGAAATCTATCAAGGCCACAGACTGGCCCTGTTC
TTTCTCTAATAAAAGTTGGATAAACTTTTGGATACTCGTGGCACACTTTTCAAGCTACTAAACGGTGTGTTTTGTACGAAAACTTTCTATATAAAAGTTGTTCTAAAATATC
AGATTAATCCA

Forward

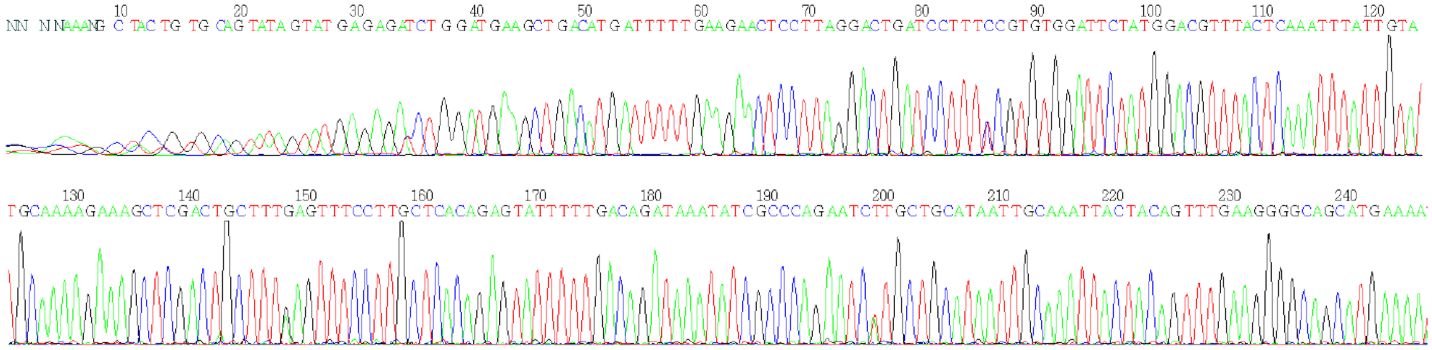

Reverse

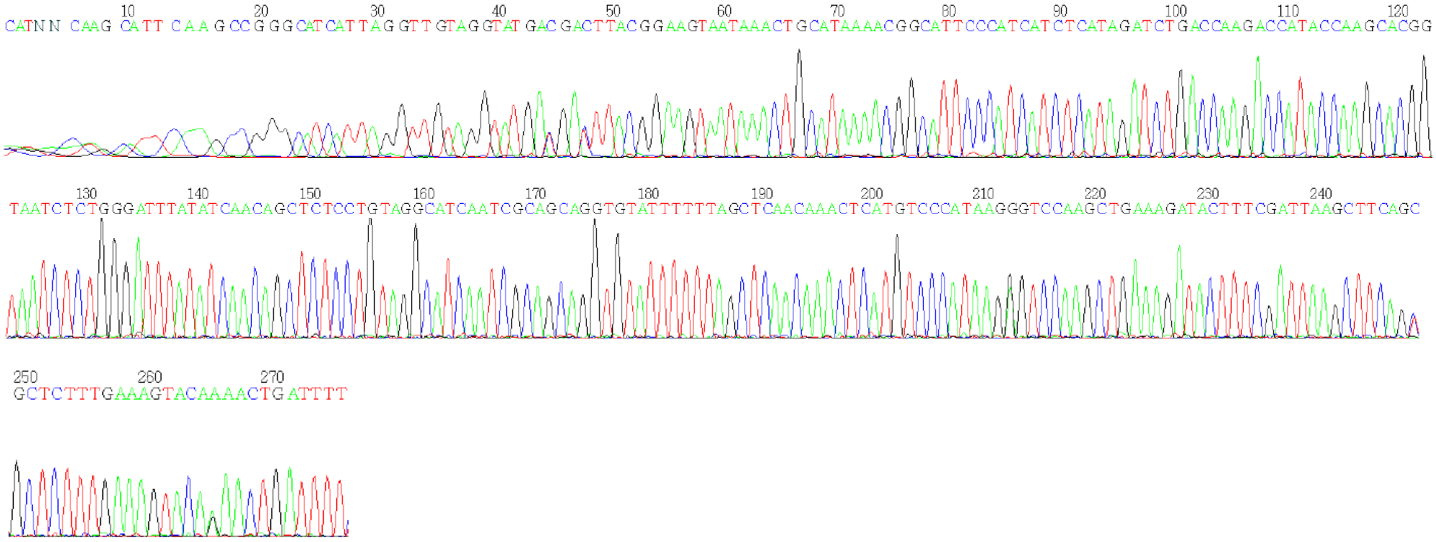

Supplement: Additional file 12 — OsCDC23_2 genomic sequence. Exon (yellow boxes), intron and possible exon (gray boxes). Sequencing result - frameshift discarded. [file 1471-2229-10-254-S12.PDF]
