# Supplementary material for: Genomic evolution and complexity of the Anaphase-promoting Complex (APC) in land plants
Source: BMC Plant Biol. 2010 Nov 18;10:254. doi: 10.1186/1471-2229-10-254 (PMC3095333; doi:10.1186/1471-2229-10-254)
Supplement: Additional file 13 — Pattern of internal domain duplications in Arabidopsis TPR subunits. The intensity of shading reflects the alignment score, with a dark shading for higher scores. The numbers on each axis indicate the domains in N-to-C terminal orientation within the repeat. [file 1471-2229-10-254-S13.PDF]

Additional file 13: Pattern of internal domain duplications in Arabidopsis TPR subunits. The intensity of shading reflects the alignment score, with a dark shading for higher scores. The numbers on each axis indicate the domains in N-to-C terminal orientation within the repeat.

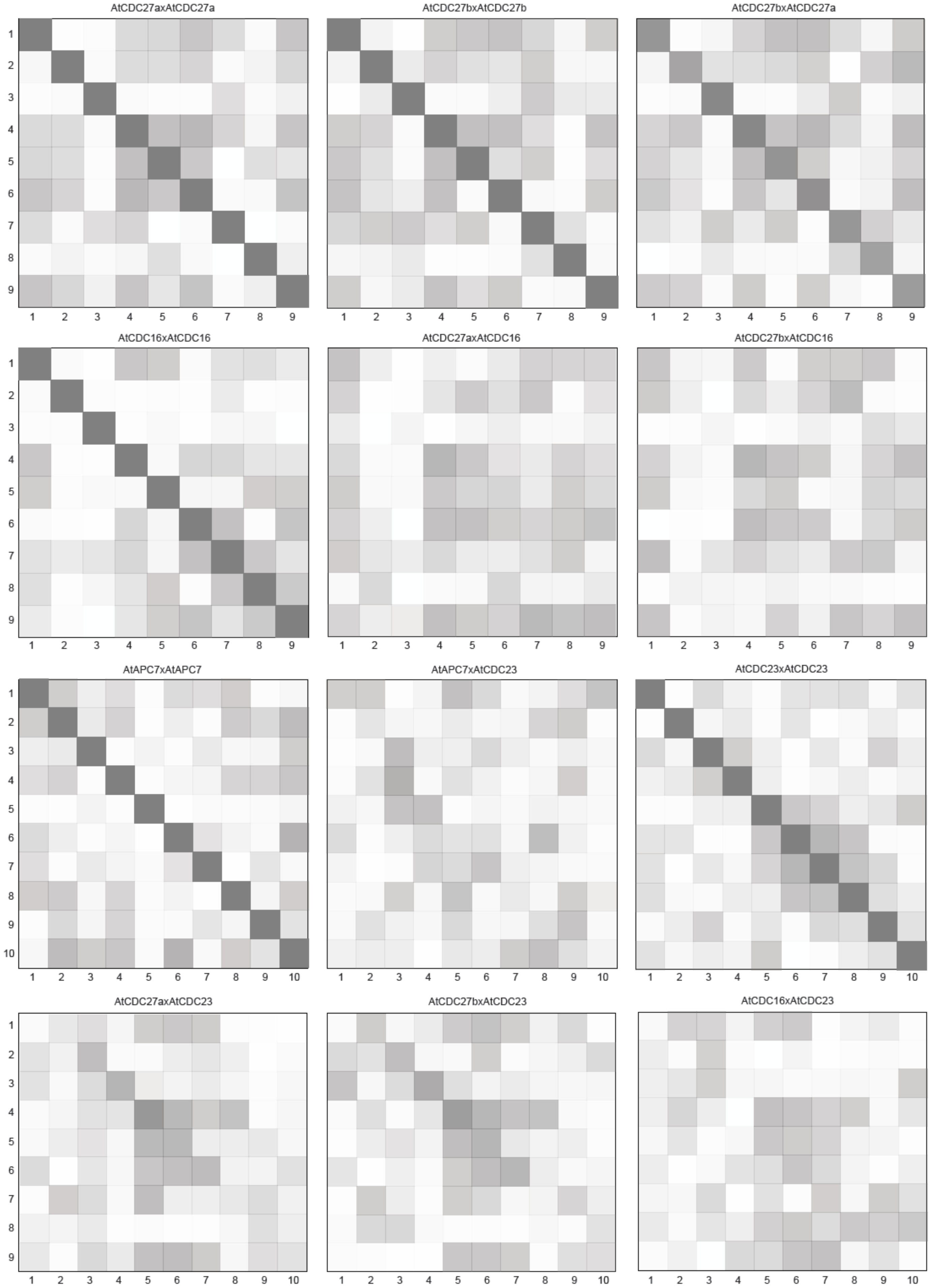

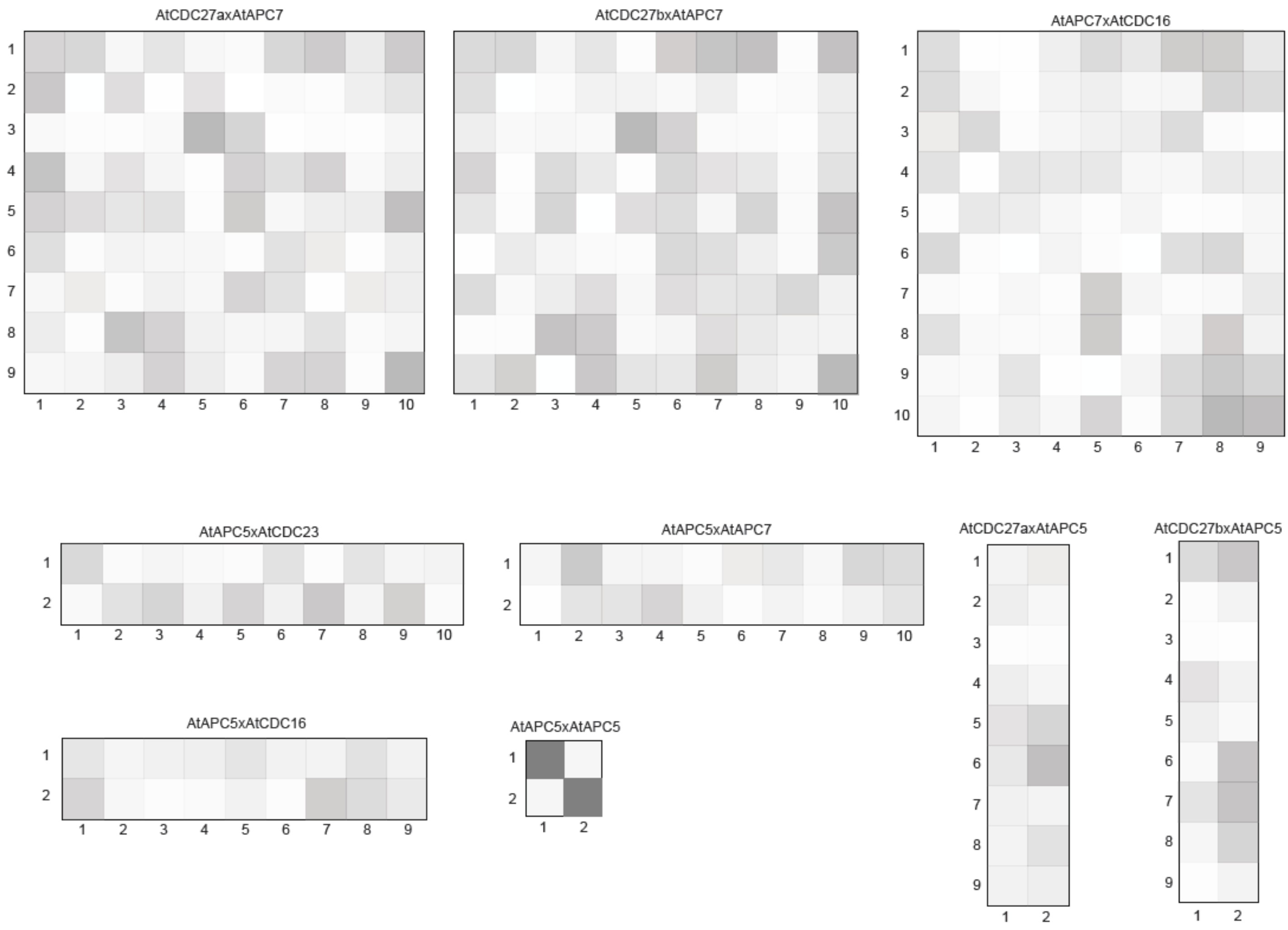
AtCDC27axAtAPC7
AtCDC27bxAtAPC7
AtAPC7xAtCDC16
AtAPC5xAtCDC23
AtAPC5xAtAPC7
AtCDC27axAtAPC5
AtCDC27bxAtAPC5
AtAPC5xAtCDC16
AtAPC5xAtAPC5
