# Supplementary material for: Genomic evolution and complexity of the Anaphase-promoting Complex (APC) in land plants
Source: BMC Plant Biol. 2010 Nov 18;10:254. doi: 10.1186/1471-2229-10-254 (PMC3095333; doi:10.1186/1471-2229-10-254)

Additional file 14: Expression patterns of rice APC genes based on Genevestigator. Expression patterns of Os*APC2*, Os*APC7*, Os*APC10*, Os*APC11_1* and Os*APC13* are shown in different tissues (A) and developmental stages (B).

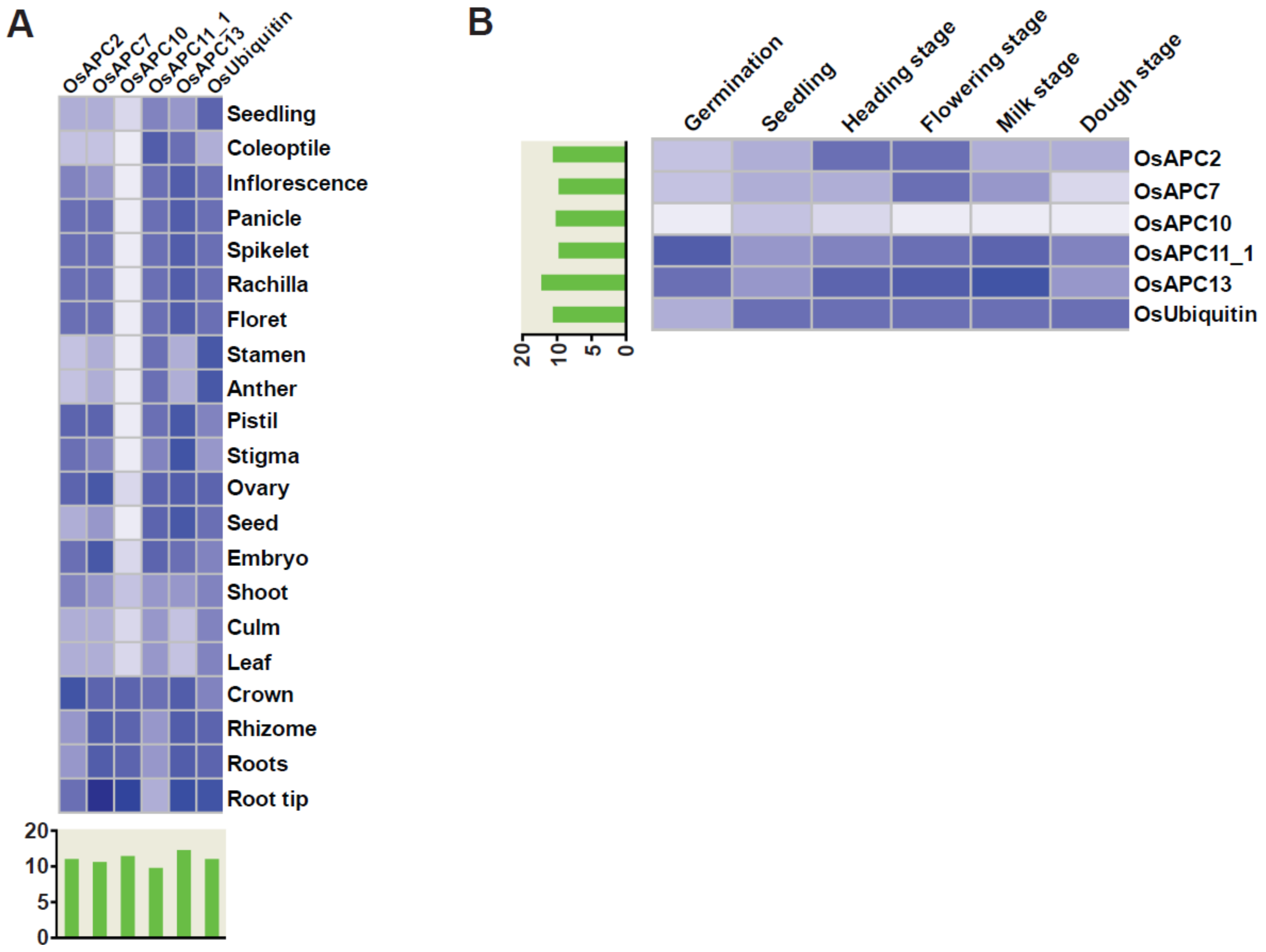

Supplement: Additional file 14 — Expression patterns of rice APC genes based on Genevestigator. Expression patterns of OsAPC2, OsAPC7, OsAPC10, OsAPC11_1 and OsAPC13 are shown in different tissues (A) and developmental stages (B). [file 1471-2229-10-254-S14.PDF]
