# Supplementary material for: Genomic evolution and complexity of the Anaphase-promoting Complex (APC) in land plants
Source: BMC Plant Biol. 2010 Nov 18;10:254. doi: 10.1186/1471-2229-10-254 (PMC3095333; doi:10.1186/1471-2229-10-254)

Additional file 15: Summary statistics for Ka and Ks. (http://services.cbu.uib.no/tools/kaks)

Ka/Ks values for each node in the tree - activators

Node#	Ka/Ks Branch1	Ka Branch1	Ks Branch1	Ka/Ks Branch2	Ka Branch2	Ks Branch2
1	0.2722	0.02281864	0.08382769	0.1852	0.01662576	0.08975730
2	0.2230	0.05842612	0.2620	0.1872	0.04866626	0.2600
3	0.2404	0.07439691	0.3095	0.1746	0.06424535	0.3679
4	0.1185	0.01063779	0.08974799	0.03576771	0.00308808	0.08633697
5	0.1095	0.03713098	0.3392	0.1027	0.04240972	0.4129
6	0.2445	0.1064	0.4352	0.5077	0.1904	0.3751
7	0.2717	0.09561973	0.3520	0.1561	0.07298646	0.4675
8	0.3389	0.1213	0.3578	0.2905	0.1165	0.4010
9	0.5821	0.3345	0.5746	0.3086	0.09245194	0.2996
10	0.1362	0.02710382	0.1991	0.1624	0.03531925	0.2174
11	0.2468	0.08345552	0.3382	0.1412	0.04950378	0.3507
12	0.8186	0.3708	0.4530	0.1789	0.06907513	0.3861
13	0.4433	0.00777269	0.01753242	1.4133	0.01271822	0.00899887
14	0.3463	0.01077193	0.03110965	0.4249	0.02095970	0.04932329
15	0.07003397	0.00237718	0.03394330	0.08949837	0.00359425	0.04015992
16	0.5461	0.07876046	0.1442	0.1592	0.02901613	0.1823
17	0.1343	0.00517381	0.03851349	0.09964731	0.00361460	0.03627390
18	0.1555	0.02778964	0.1787	0.2410	0.02959975	0.1228
19	0.1652	0.09623061	0.5826	0.5577	0.2532	0.4540
20	0.1170	0.03729277	0.3188	0.2283	0.06562150	0.2874
21	0.1554	0.03939388	0.2535	0.06509602	0.02163868	0.3324
22	0.2281	0.08520646	0.3735	0.1591	0.05869326	0.3688

Ka/Ks values for each node in the tree – APC11

Node#	Ka/Ks Branch1	Ka Branch1	Ks Branch1	Ka/Ks Branch2	Ka Branch2	Ks Branch2
1	0.1535	0.01076786	0.07013537	0.03965201	0.00467023	0.1178
2	0.02695868	0.00992007	0.3680	0.01120257	0.00465961	0.4159
3	0.05657378	0.02373612	0.4196	0.05874972	0.02507865	0.4269

Ka/Ks values for each node in the tree – CDC27

Node#	Ka/Ks Branch1	Ka Branch1	Ks Branch1	Ka/Ks Branch2	Ka Branch2	Ks Branch2
1	0.5498	0.2675	0.4865	0.3339	0.1644	0.4924
2	0.4267	0.1366	0.3200	0.2573	0.06389280	0.2483
3	0.2979	0.02423422	0.08134658	0.1802	0.01271027	0.07051774
4	0.2414	0.05176730	0.2145	0.2042	0.03942175	0.1930

Ka/Ks values for each node in the tree – CDC23

Node#	Ka/Ks Branch1	Ka Branch1	Ks Branch1	Ka/Ks Branch2	Ka Branch2	Ks Branch2
1	0.6119	0.03785531	0.06186091	0.1198	0.00801678	0.06690476
2	0.1722	0.05833490	0.3387	0.1462	0.05551671	0.3796
3	0.2049	0.07820839	0.3817	0.1975	0.08139534	0.4122

Ka/Ks annotated evolutionary tree

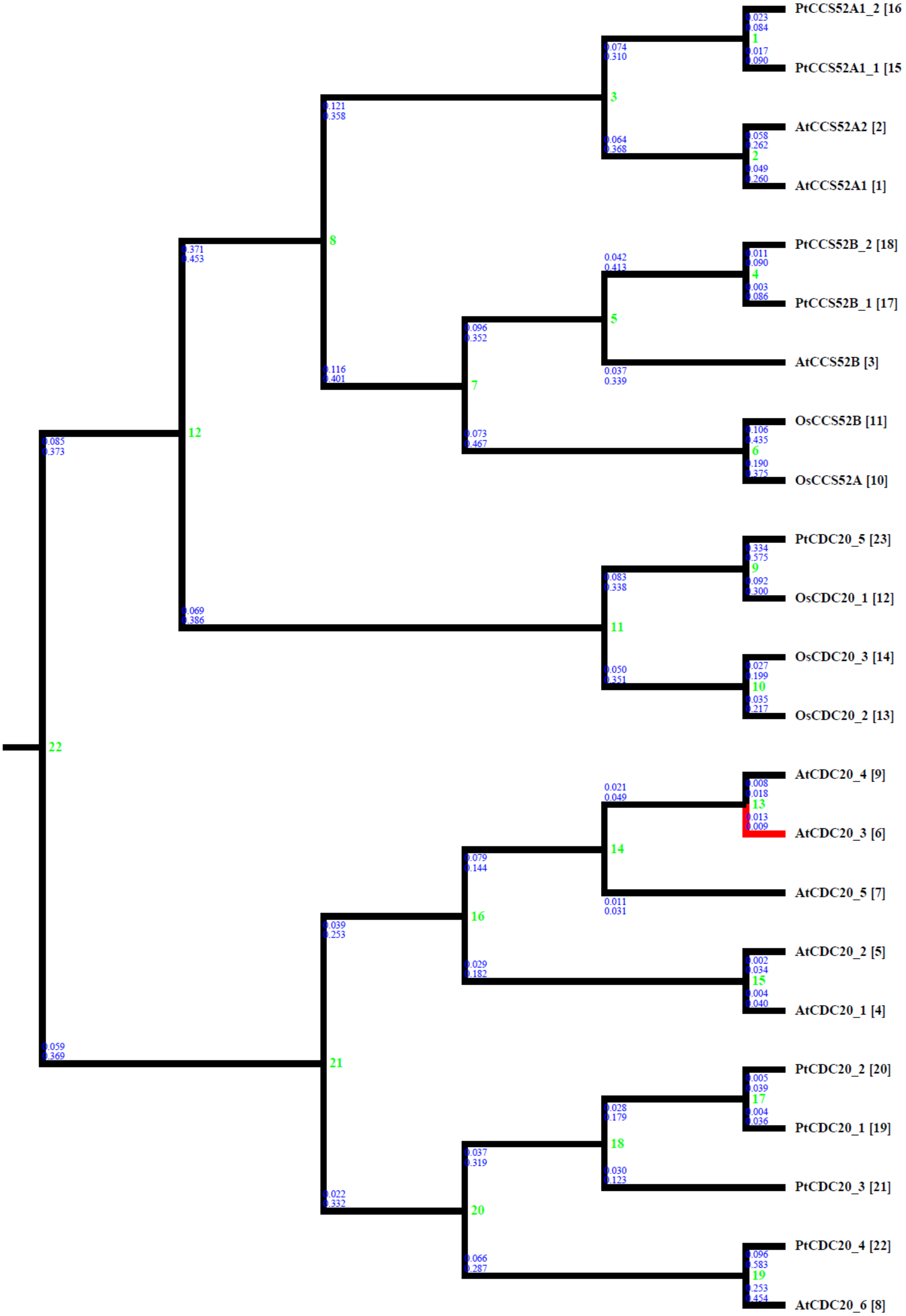

Ka/Ks annotated evolutionary tree

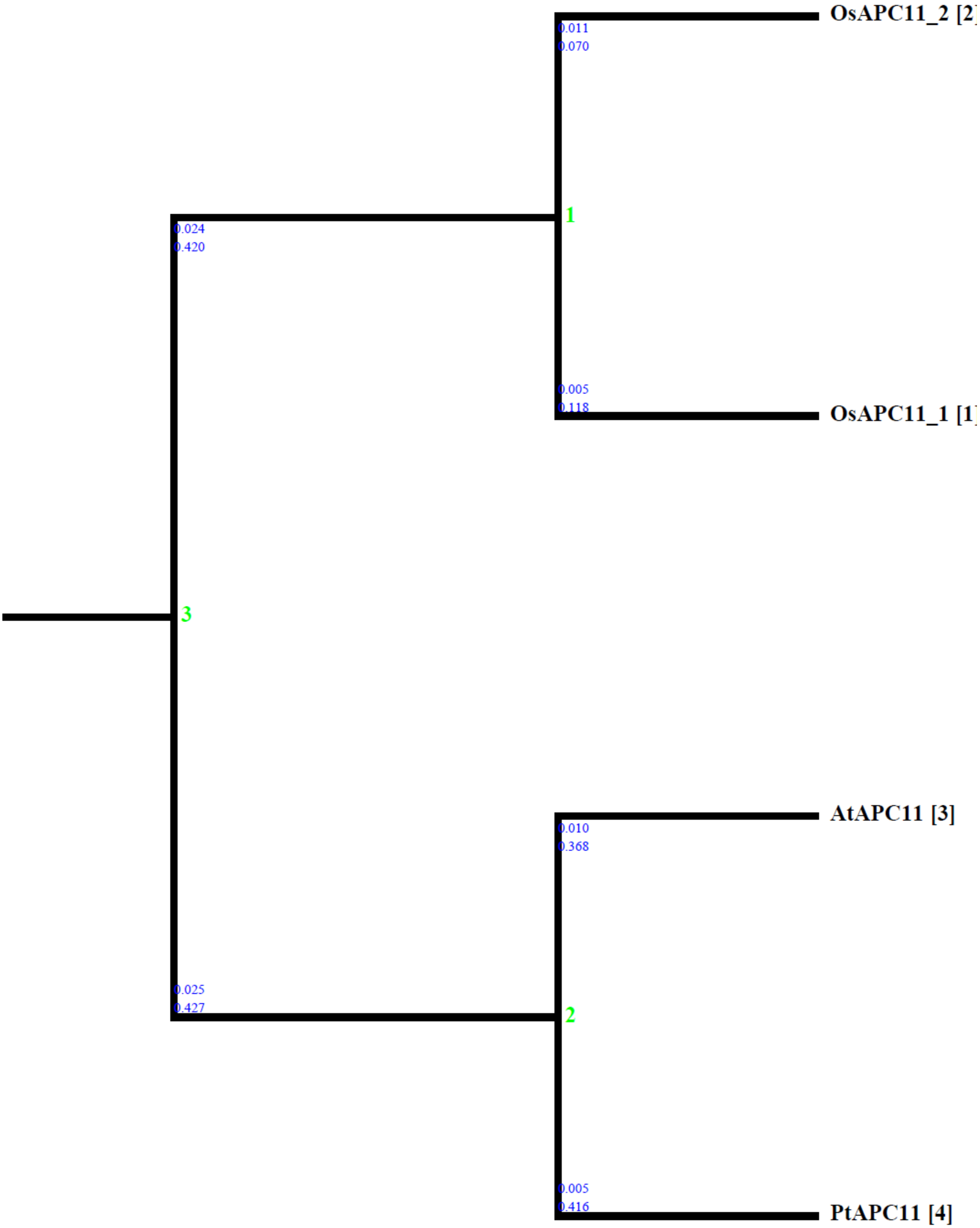

Ka/Ks annotated evolutionary tree

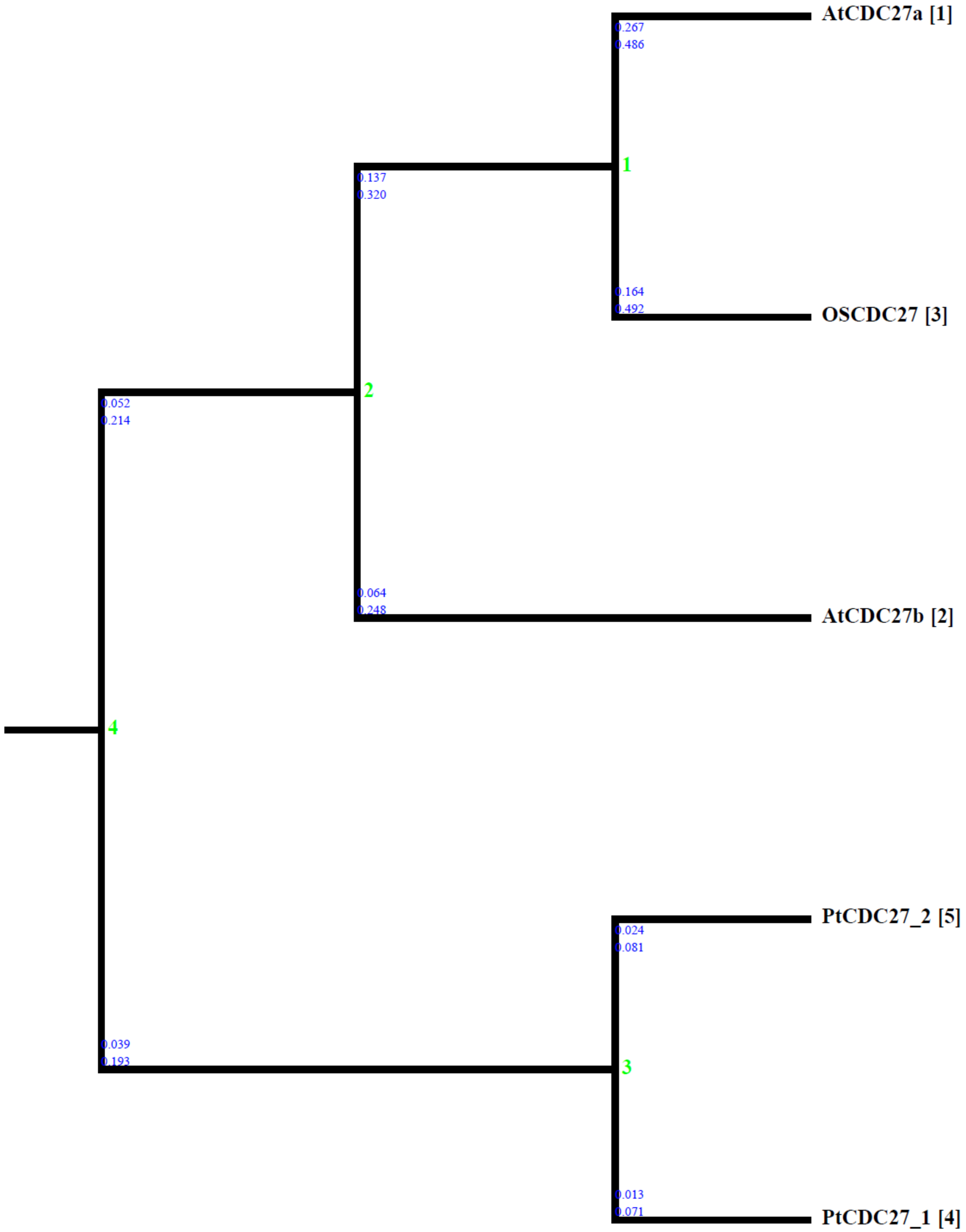

Ka/Ks annotated evolutionary tree

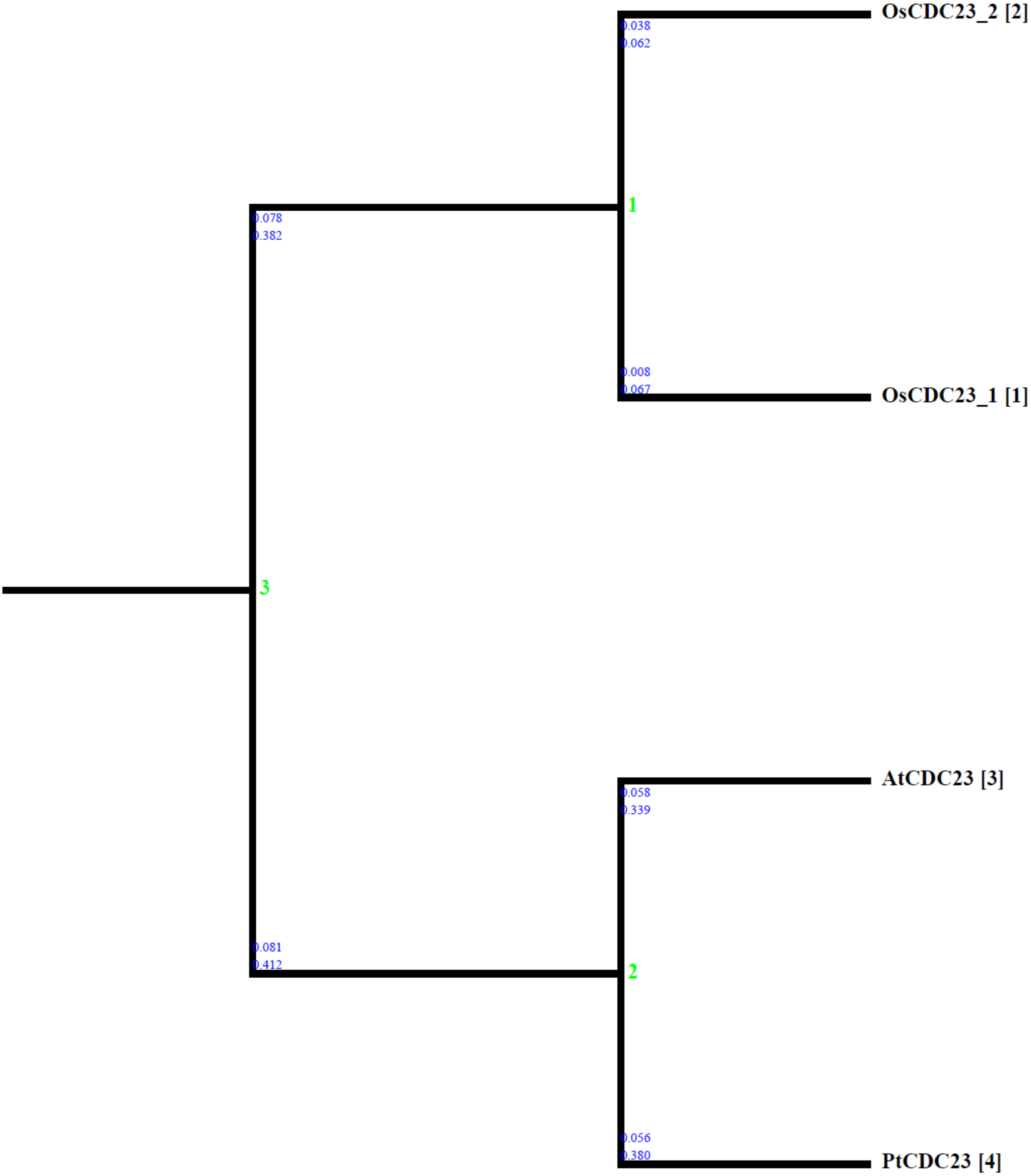

Supplement: Additional file 15 — Summary statistics for Ka and Ks. http://services.cbu.uib.no/tools/kaks. [file 1471-2229-10-254-S15.PDF]
