# Supplementary material for: Genomic evolution and complexity of the Anaphase-promoting Complex (APC) in land plants
Source: BMC Plant Biol. 2010 Nov 18;10:254. doi: 10.1186/1471-2229-10-254 (PMC3095333; doi:10.1186/1471-2229-10-254)
Supplement: Additional file 16 — Promoter analysis. The upstream 1000 bp regions of all rice APC subunits genes were considered to contain the full length promoters. Table 2, motifs found in the promoters and their occurrences. Table 3, motifs found in each duplicated gene (in red, same motifs). [file 1471-2229-10-254-S16.PDF]

Additional file 16: Promoter analysis. The upstream 1000 bp regions of all rice APC subunits genes were considered to contain the full length promoters. Table 2, motifs found in the promoters and their occurrences. Table 3, motifs found in each duplicated gene (in red, same motifs).

Table 2

Cis acting	Sequence	Occurrence	Function
**ARFAT**	TGTCTC	6	Auxin response factor
**HEXMOTIFTAH3H4**	ACGTCA	5	Regulation of replication
**CRTDREHVCBF2**	GTCGAC	4	Sequence for AP2 transcriptional activator
**INTRONLOWER**	TGCAGG	4	Plant intron lower sequence
**LECPLEACS2**	TAAAATAT	4	Regulator of ACC synthase
**PYRIMIDINEBOXOSRAMY1A**	CCTTTT	4	Involved in sugar repression
**QELEMENTZMZM13**	AGGTCA	4	Expression enhancing activity
**REALPHALGLHCB21**	AACCAA	4	Required for phytochrome regulation
**T/GBOXATPIN2**	AACGTG	4	Play a key role in jasmonate signaling
**CACTFTPPCA1**	YACT	3	Component mesophyll expression module1
**ERELEE4**	AWTTCAAA	3	Is related to senescence
**MYB2AT**	TAACTG	3	Regulation genes responsive to water stress
**RYREPEATBNNAPA**	CATGCA	3	Required for seed specific expression
**SEF1MOTIF**	ATATTTAWW	3	Regulation beta-conglycinin genes
**SITEIIATCYTC**	TGGGCY	3	Regulated oxidative phosphorylation
**TGACGTVMAMY**	TGACGT	3	Required for high level of alpha-amylase
**WBOXATNPR1**	TTGAC	3	Recognized specifically by salicylic acid

Table 3

OsCDC23_1	OsCDC23_2	OsAPC11_1	OsAPC11_2
**ACGTABOX**	ARFAT	ARFAT	ERELEE4
**CRTDREHVCBF2**	BIHD1OS	CTRMCAMV35S	GTGANTG10
**CTRMCAMV35S**	INRNTPSADB	DRE2COREZMRAB17	**INTRONLOWER**
**HEXMOTIFTAH3H4**	INTRONLOWER	HEXMOTIFTAH3H4	RYREPEATBNNAPA
**LECPLEACS2**	TGACGTVMAMY	**INTRONLOWER**	TATCCAYMOTIFOSRAMY3D
**PYRIMIDINEBOXOSRAMY1A**		LECPLEACS2	**WBOXATNPR1**
**QELEMENTZMZM13**		LTRE1HVBLT49	WBOXNTERF3
**REALPHALGLHCB21**		MYB2AT	
**SEF1MOTIF**		SEF4MOTIFGM7S	
**SEF4MOTIFGM7S**		SITEIIATCYTC	
**WBOXNTERF3**		SP8BFIBSP8BIB	
		**WBOXATNPR1**	
